# The Selective Interaction between Silica Nanoparticles and Enzymes from Molecular Dynamics Simulations

**DOI:** 10.1371/journal.pone.0107696

**Published:** 2014-09-22

**Authors:** Xiaotian Sun, Zhiwei Feng, Liling Zhang, Tingjun Hou, Youyong Li

**Affiliations:** Institute of Functional Nano & Soft Materials (FUNSOM) and Collaborative Innovation Center of Suzhou Nano Science and Technology, Soochow University, Suzhou, China; Oak Ridge National Laboratory, United States of America

## Abstract

Nanoscale particles have become promising materials in many fields, such as cancer therapeutics, diagnosis, imaging, drug delivery, catalysis, as well as biosensors. In order to stimulate and facilitate these applications, there is an urgent need for the understanding of the interaction mode between the nano-particles and proteins. In this study, we investigate the orientation and adsorption between several enzymes (cytochrome c, RNase A, lysozyme) and 4 nm/11 nm silica nanoparticles (SNPs) by using molecular dynamics (MD) simulation. Our results show that three enzymes are adsorbed onto the surfaces of both 4 nm and 11 nm SNPs during our MD simulations and the small SNPs induce greater structural stabilization. The active site of cytochrome c is far away from the surface of 4 nm SNPs, while it is adsorbed onto the surface of 11 nm SNPs. We also explore the influences of different groups (-OH, -COOH, -NH_2_ and CH_3_) coated onto silica nanoparticles, which show significantly different impacts. Our molecular dynamics results indicate the selective interaction between silicon nanoparticles and enzymes, which is consistent with experimental results. Our study provides useful guides for designing/modifying nanomaterials to interact with proteins for their bio-applications.

## Introduction

Nanoscale particles have become promising materials in many fields, such as cancer therapeutics, diagnosis, imaging, drug delivery, catalysis, as well as biosensors. However, very little is known about exactly how a protein interacts with nano-particles and how its orientation is governed by the size, shape, and chemistry of the surface of the nano-particles. In order to stimulate and facilitate these applications, there is an urgent need for the understanding of the interaction mode between the nano-particles and proteins. In this study, we investigate the selective orientation and adsorption between several important enzymes (cytochrome c, RNase A, lysozyme) and 4 nm/11 nm silica nanoparticles (SNPs) by using molecular dynamics (MD) simulation.

Enzymes [1], [2] regulate almost all chemical reactions involved in numerous biological processes, such as signal transduction, gene expression, immune responses, metastasis, and metabolism. Moreover, enzymes are widely used in pharmaceutical and medical fields, food and environmental industry, biofuel area, as well as life science studies. [3] Cytochrome c [4]–[6] (Cytc) is located in the mitochondrial inter-membrane space where it functions as a mobile electron carrier between complexes III and IV of the electron transport chain (ETC). The role of Cytc in mitochondrial ATP production is essential, as Cytc knockout mice die around midgestation, when metabolism switches from the glycolytic pathway to aerobic energy production. Ribonuclease A [7]–[9] (RNase A) is a pancreatic ribonuclease that cleaves single-stranded RNA. This enzyme has played a crucial role as a model system in studies of protein structure, folding and unfolding pathways and enzyme catalysis. Lysozyme [10], [11] is part of the innate immune system. Reduced lysozyme levels have been associated with bronchopulmonary dysplasia in newborns. Children fed infant formula lacking lysozyme in their diet have three times the rate of diarrheal disease. Since lysozyme is a natural form of protection from gram-positive pathogens like Bacillus and Streptococcus, a deficiency due to infant formula feeding can lead to increased incidence of disease. Whereas the skin is a protective barrier due to its dryness and acidity, the conjunctiva (membrane covering the eye) is, instead, protected by secreted enzymes, mainly lysozyme and defense.

Nano-biotechnology is a promising and interdisciplinary research involving chemistry, physics, biology and medicine. Silicon nanomaterials [12]–[15] are a type of important nanomaterials with attractive properties including excellent electronic/mechanical properties, favorable biocompatibility, huge surface-to-volume ratios, surface tailorability, improved multifunctionality, as well as their compatibility with conventional silicon technology. Consequently, there has been great interest in developing functional silicon nanomaterials for various applications ranging from electronics to biology.

Elucidating protein orientation [12] on nanoscale surfaces has important implications for integrating proteins into micro and nanofabricated devices. Applications include biosensing, actuating of microelectromechanical systems (MEMS), and tissue engineering, as well as screening tools for drug discovery and basic biological research. Although the literature is replete with reports on the structure, stability, and activity of proteins adsorbed onto nanomaterial surfaces, very little is known about exactly how a protein interacts with a surface and how its orientation is governed by the size, shape, and chemistry of the surface. These are major questions that must be addressed to design both nanoscale surfaces and proteins to achieve optimal conjugate functionality.

In the present work, we investigate the orientation and adsorption between several enzymes (cytochrome c, RNase A, lysozyme) and 4 nm/11 nm silica nanoparticles (SNPs) by using molecular dynamics (MD) simulation. Our results show that three enzymes are adsorbed onto the surfaces of both 4 nm and 11 nm SNPs during our MD simulations and the small SNPs induce greater structural stabilization. We also explore the influences of different groups (-OH, -COOH, -NH_2_ and CH_3_) coated onto silica nanoparticles, which show significantly different impacts.

## Materials and Methods

### 1 Prepared Structures

The crystal structures of cytochrome c [5] (PDB entry: 3NWV, resolution 1.90 Å), RNase A [7](PDB entry: 7RSA, resolution 1.26 Å) and lysozyme [10] (PDB entry: 3LN2, resolution 2.04 Å) are used in our studies. The structures are retrieved from the Protein Data Bank (http://www.pdb.org/pdb/). The crystal structures are then prepared by Discovery Studio 2.5 [16] (including residues repair and energy minimization).

Four disulfide bonds are found in the native state of RNase A: Cys26–Cys84, Cys58–110, Cys40–95 and Cys65–72, other four disulfide bonds are found in lysozyme: Cys6–Cys128, Cys30–116, Cys65–81 and Cys77–95. We patch these disulfide bonds before perform molecular dynamics simulations.

Histidine residue is the only one which ionizes within the physiological pH range (∼7.4). To determine the protonation states for histidines and other residues, we use Discovery studio 2.5 to predict protein ionization and residue pK values.

For cytochrome c, the calculated pK values of histidines are: 6.430 for His18, 5.047 for His26, and 6.745 for His33. His18, His26, and His33 in cytochrome c are not protonated.

For RNase A, the calculated pK values of histidines are: 7.389 for His12, 7.668 for His48, 7.170 for His105, and 6.747 for His119. All histidine residues in RNase A are not protonated.

For lysozyme, the calculated pK values of histidines are: 6.983 for His78 and 6.627 for His115. All histidine residues in lysozyme are not protonated.

Side chains of Asp, Glu, Arg, and Lys were charged (Asp^−^, Glu^−^, Arg^+^, and Lys^+^) in all simulations.

In the present work, we simulate the enzymes adsorbed onto the silica nanoparticles (SNPs) with different surface curvatures: 4 nm SNPs and 11 nm SNPs. In order to save computational load, we use the following protocol to obtain different surface curvatures of SNPs.

**Figure 1:**
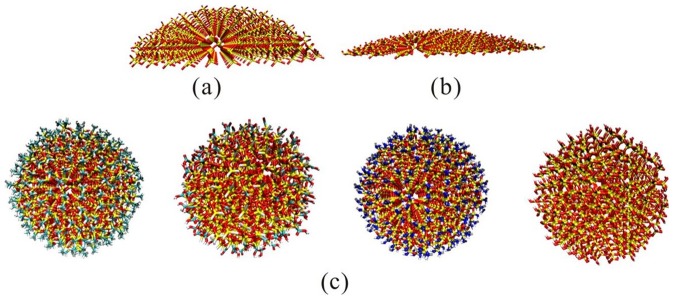
Side view of the energy-minimized molecular structure of the (a) part of 4 nm SNPs, (b) part of 11 nm SNPs and (c) different chemical groups (-CH3, -COOH, -NH2, -OH) coated onto 2 nm SNPs.

We first build two different radiuses of silica nanoparticles (SNPs): 4 nm and 11 nm, which provides a sufficient surface for the enzymes to adsorb. Then we perform geometry optimization on the SNPs by Material Studio 5.5 with Forcite module. [17] Dreiding force field, Gasteiger (maximum iteration is setting 50,000, convergence limit is setting 5.0e^−6^ e) and ultra-fine quality are used for the energy calculation. Then we cut the SNPs by using the central angle of 60 degree; the output structure (Figure 1a and Figure 1b) is regarded as the initial structure of 4 nm/11 nm SNPs for the following molecular dynamics simulations to interact with different enzymes. We use the partial structures in Fig. 1a and Fig. 1b to represent the different surface curvature of SNPs and save the computational load for the simulations.

We also investigate the influences of different chemical groups (-CH_3_, -COOH, -NH_2_ and -OH) coated onto silica nanoparticles. We build 2 nm SNPs by using Material Studio 5.5. [17] Different chemical groups (-CH_3_, -COOH, -NH_2_ and -OH) are attached to the silicon atoms on the surface. Geometry optimizations of the SNPs are performed with Forcite module. The parameters for geometry optimizations include Dreiding force field, Gasteiger (maximum iteration is setting 50,000, convergence limit is setting 5.0e^−6^ e) and ultra-fine quality. The optimized structures are listed in Figure 1c and used for the following simulations.

### 2 MD Simulations

**Figure 2:**
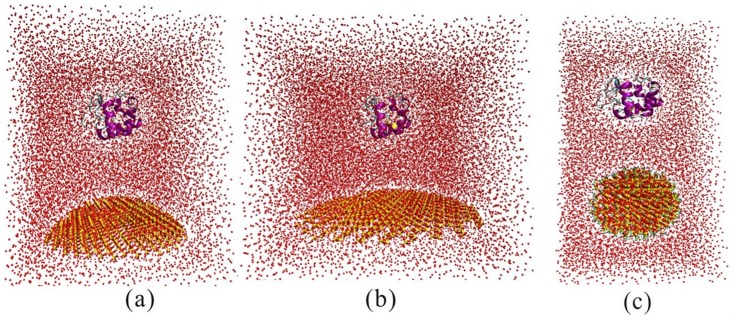
The molecular dynamics simulation box of SNPs and enzymes with water. (a) 4 nm SNP with the enzyme. The box size is 8.0×8.0×8.0 nm^3^; (b) 11 nm SNP with the enzyme. The box size is 11.0×11.0×8.0 nm^3^; (c) 2 nm SNP with the enzyme. The box size is 6.0×6.0×10.0 nm^3^.

Silicon atoms of SNPs are uncharged in accordance with Hummer et al. [18] The SNPs are fixed during the molecular dynamics simulations. The initial minimum (MIN) distance between SNPs surface and enzyme is 2.0 nm in all three systems to make sure that they don't interact with each other in the beginning of the simulations. Importantly, the active pockets of enzymes are far away from the SNPs (Figure 2). Then the enzymes and the SNPs are embedded in periodic boundary conditions in a rectangular water box (TIP3P [19] water model) with a size of 6.0×6.0×10.0 nm ^3^/8.0×8.0×8.0 nm^3^/11.0×11.0×8.0 nm^3^ (for 2 nm/4 nm/11 nm). The waters within 3/2 Å of the enzymes/SNPs are eliminated. VMD [20] software is used for setting up the molecular dynamics simulation systems.

For cytochrome c, the whole system (Figure 2) contains cytochrome c, SNPs (4 nm/11 nm), ∼28,941/∼15,811 water molecules, 10 chloride ions and 0 sodium ion for a total of ∼78,275/∼52,462 atoms per periodic cell. In our simulation, we don't add the ligand-heme in the system. We want to see the effect of SNPs on the cytochrome c without ligands. For comparisons, we also perform the MD simulation of natural structure of cytochrome c with explicit water.

We also perform molecular dynamics simulations of cytochrome c with 2 nm SNPs with different groups(-CH_3_, -COOH, -NH_2_ and -OH), as shown in Figure 2c. Under these conditions, the whole system contains cytochrome c, 2 nm SNPs coated with -CH_3_/-COOH/-NH_2_/-OH, ∼10,299/∼10,396/∼10,502/∼10,200 water molecules, 11/11/11/10 chloride ions and 0/0/0/0 sodium ion for a total of ∼36,774/∼36,277/∼36,945/∼35,612 atoms per periodic cell.

For RNase A, the whole system contains RNase A, SNPs (4 nm/11 nm), ∼28,948/∼19,698 water molecules, 5 chloride ions and 0 sodium ion for a total of ∼77,944/∼52,205 atoms per periodic cell. For comparisons, we also perform the MD simulation of natural structure of RNase A with explicit water.

For lysozyme, the whole system contains lysozyme, SNPs (4 nm/11 nm), ∼24022/∼15,684 water molecules, and 5 chloride ions and 0 sodium ion for a total of ∼78,148/52,432 atoms per periodic cell. For comparisons, we also perform the MD simulation of natural structure of lysozyme with explicit water.

The systems are first equilibrated for 200 ps with the enzymes fixed. Then the enzymes are released and another 200 ps equilibration is performed. The enzymes in the water box are energy minimized.

Starting from the last frame of the equilibration, we perform 100 ns molecular dynamics simulations. Two different orientations of enzymes' conformation are tried in the present work. So, two independent MD simulations of each system are performed using the NAMD package [21] (version 2.7b2) with CHARMM27 [22]–[24] force field for the studied complex with explicit water. In addition, Dreiding force field (K_b_ value of silica is 350) is used for the SNPs. Electrostatics are calculated using the Particle Mesh Ewald [25] (PME) method with a 12 Å non-bonded cutoff and a grid spacing of 1 Å per grid point in each dimension. The van der Waals energies are calculated using a smooth cutoff (switching radius 10 Å, cutoff radius 12 Å). The temperature and pressure are kept constant using a langevin thermostat (310 K) and langevin barostat (1 atm), respectively. The time step of MD simulations is set to 1 fs. The data is saved every 10 ps for analysis. Trajectory analyses are carried out with VMD.

### 3 Evaluation of Active Site of Cytochrome c

In order to examine the change of the active site of cytochrome c before and after SNPs binding, we construct 3D models for heme-cytochrome c by using several docking programs, which include Glide in Schrödinger, [26] GOLD, [27] LigandFit, [28] LibDock, [29] Flexible Docking [30] and CDocker [31] program in Discovery Studio 2.5. However, we cannot construct the 3D models. So we investigate the changes of active site of enzyme. We compare the active site after adsorbed onto SNPs with the active site in the crystal structure to investigate the conformational change.

### 4 Construction for the 3D Model of RNase A -TBU Complex

In order to examine the change of the active site of RNase A before and after SNPs binding, we construct 3D models for RNase A-TBU (tertiary-butyl alcohol) by using CDocker program in Discovery Studio 2.5. The dock program CDocker and DS Catalyst Score are applied to construct receptor-ligand complexes. The binding site of the receptor is set at the active site of RNase A (if it exists) with a radius of 5 Å, large enough to cover the binding pocket. CDocker is a grid-based molecular docking method that employs CHARMM force-field. The receptor is held rigid while the ligands are allowed to flex during the refinement. For pre-docked ligands, prior knowledge of the binding site is not required. It is possible, however, to specify the ligand placement in the active site using a binding site sphere. Random ligand conformations are generated from the initial ligand structure through high temperature molecular dynamics, followed by random rotations. The random conformations are refined by grid-based simulated annealing and a final grid-based or full force field minimization.

## Results and Discussion

After minimization of the systems, we perform two independent molecular dynamics simulations of enzymes and SNPs in explicit water. We compare the molecular dynamics trajectories systematically to identify the differences for enzymes under different diameter of SNPs and study the selective interactions between SNPs and enzymes.

**Figure 3:**
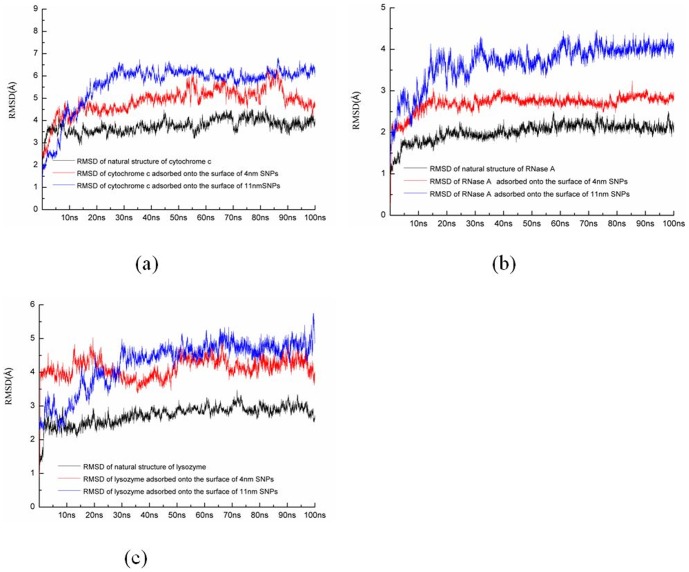
Time evolutions for (a) cytochrome c, (b) RNase A, and (c) lysozyme during 100 ns MD simulations. The values are the average value from two independent MD simulations.

**Figure 4:**
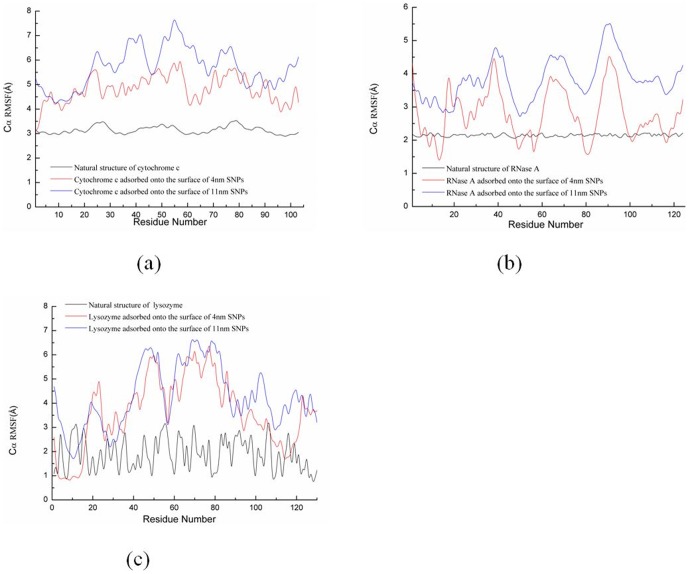
Calculated RMSF of Cα atoms vs protein residue number for (a) cytochrome c (103 residues), (b) RNase A (124 residues), and (c) lysozyme (130 residues) during the MD simulation. A comparison between the RMSF plot for natural structures of proteins, proteins adsorbed onto the surface of 4 nm SNPs, and the proteins adsorbed onto the surface of 11 ns SNPs.

Figure 3 shows the root mean square deviation (RMSD) of the all Cα atoms of enzymes during two independent MD simulations with different orientations. Figure 4 shows the average backbone root mean square fluctuation (RMSF) values for each amino acid residue of cytochrome c, RNase A, and lysozyme.

During 100 ns MD simulation, we find that the RMSD of each system is equilibrated after 20 ns. Moreover, our results show that after the proteins adsorbed onto the surface of SNPs, the positions and the conformations of proteins keep stable. We compare the conformations of enzymes after 35 ns, and find little changes for both positions and structures (See the following discussions). The timescale of 100 ns is reasonable for our purpose to validate the interactions between enzymes and SNPs.

### 1 Adsorption of Enzymes onto SNPs

Our results show that all enzymes are adsorbed onto the surface of SNPs (both for 4 nm SNPs and 11 nm SNPs), although the interaction modes are different.

#### 1.1 The Adsorption of Cytochrome c

**Figure 5:**
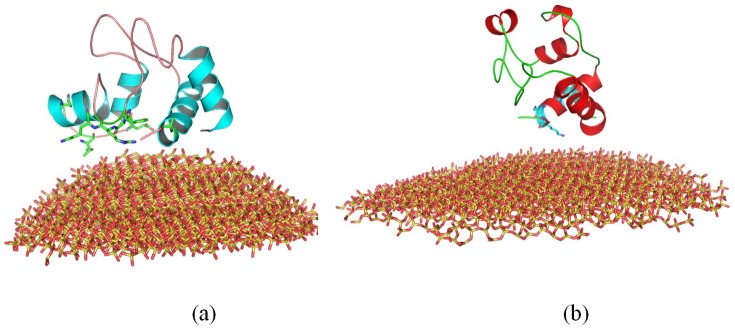
The structure of cytochrome c adsorbed onto the surface of SNPs. (a) with 4 nm SNP, (b) with 11 nm SNP. The structures are the conformations after 100 ns MD simulation.

The initial minimum (MIN) distance between cytochrome c and SNPs is 2.0 nm. Our results show that cytochrome c is gradually adsorbed onto the surface of SNPs (both 4 nm and 11 nm). After 20 ns, cytochrome c is adsorbed onto the surface of SNPs. Our results show that during 35 to 100 ns MD simulation, the positions and the conformations of cytochrome c keep stable. Indeed, one independent simulations of cytochrome c is performed for 167 ns, we compare the conformations between 35 ns to 167 ns, and find little change, as shown in Figure S1c. After 100 ns, the minimum distance between cytochrome c and 4 nm SNP is ∼1.36 Å; while the minimum distance between cytochrome c and 11 nm SNP is ∼0.75 Å, as shown in Figure 5. Figure S1 in File S1 shows the results of another independent simulation.

Our results show different binding modes and different residues involved in the adsorptions, due to the different surface curvature of 4 nm SNP and 11 nm SNP.

Figure 5a and Figure S1a show some key residues contributed to the adsorption of cytochrome c onto 4 nm SNPs, which include Phe36, Gly37, Arg38, Lys39, Pro44, Lys55, Ile58, Glu61, Asp62 and Asn103. These residues are adsorbed onto the surface of 4 nm SNPs through their hydrophobic interactions. The distances between these residues and SNPs surface are less than 0.8 Å. Figure 4a shows that the RMSFs of these residues are about 4.6 Å during the MD simulation. Two α-helixes are completely adsorbed onto the surface of 4 nm SNPs, including “KEERADLIAYLKKA” (residue 88 to 101) and “EDTLMEY” (residue 61 to 67). The residues from these two α-helixes keep stable after adsorption onto the surface, which the RMSFs are lower than other residues, as shown in Figure 4a.

However, it is quite different for the adsorption of cytochrome c onto the surface of 11 nm SNPs, as shown in Figure 5b and Figure S1b. Residues (from two independent simulations) including Ala92, Ile95, Ala96, Lys99 and Lys100, form strong hydrophobic interactions with SNPs. However, the α-helix consisted of residue 1 to 17(“GDVEKGKKIFIMKC”) is close to SNPs. The RMSFs of these residues adsorbed onto 11 nm SNPs are larger than that adsorbed onto 4 nm SNPs, as shown in Figure 4a.

#### 1.2 The Adsorption of RNase A

The initial minimum (MIN) distance between RNase A and SNPs is also 2.0 nm. RNase A is also adsorbed onto the SNPs after 20 ns MD simulation. We compare the conformations between 35 ns to 133 ns (one simulation of RNase A is performed for 133 ns), and also find little change, as shown in figure S2c. After 100 ns, the minimum distance between RNase A and 4 nm SNPs is ∼1.19 Å; while the minimum distance between RNase A and 11 nm SNPs is ∼0.97 Å. Figure S2 in File S1 shows the results of another independent simulation.

**Figure 6:**
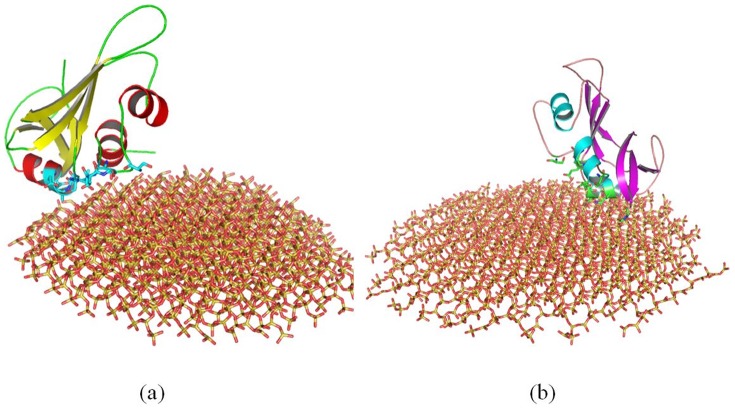
Structure of RNase A adsorbed onto the surface of SNPs. (a) with 4 nm SNP, (b) with 11 nm SNP. The structures are the conformations after 100 ns MD simulation.

Figure 6a and Figure S2a show some key residues contributed to the adsorption of RNase A onto 4 nm SNPs, including Ser15, Ser16, Thr17, Ser18, Ser21, Ser50, Leu51, Ala52, Asp53, Gln55. Three residues from a α-helix (residue 50 to 59, “SLADVQAVCS”) are adsorbed onto the surface of SNPs. Most of the RMSFs of these adsorbed residues (2.0∼3.0 Å) are lower than other residues, as shown in Figure 4b.

However, as shown in Figure 6b and Figure S2b, our results show that the similar residues directly interact with the surface of 11 nm SNPs, including Thr3, Glu9, Met13, Ser15, Ser16, Leu51, Ala52, Gln55 and Pro114. Two α-helixes are adsorbed onto the SNPs surface, including “KETAAAKFERQH”(residue 1 to 12) and “LADVQAV”(residue 51 to 57). The RMSFs of these residues are shown in Figure 4b, which the values are larger for the adsorption onto 11 nm SNPs.

#### 1.3 The Adsorption of Lysozyme

**Figure 7:**
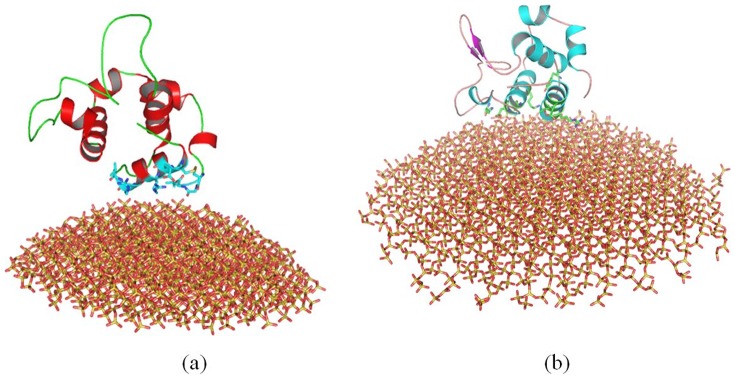
Structure of lysozyme adsorbed onto the surface of SNPs. (a) with 4 nm SNP, (b) with 11 nm SNP. The structures are the conformations after 100 ns MD simulation.

The initial minimum (MIN) distance between lysozyme and SNPs is also 2.0 nm. As shown in Figure 7, after 100 ns molecular dynamics simulation, the minimum distance between lysozyme and 4 nm SNPs is ∼1.44 Å; while the minimum distance between lysozyme and 11 SNPs is ∼1.06 Å. We compare the conformations between 35 ns to 145 ns (one simulation of lysozyme is performed for 145 ns), and also find little change, as shown in figure S3c. Figure S3 in file S1 shows the results of another independent simulation.

Some residues of lysozyme including Cys6, Glu7, Arg10, Arg14, Leu15, Cys128, Gly129 and Val130, are adsorbed onto 4 nm SNP directly. The distances between these residues and 4 nm SNP are less than 1.5 Å. The α-helix consisted of residue 1 to 16 (“KVFERCELARTLKRLG”) interacts strongly with 4 nm SNP. The RMSFs of these residues are lower than 3 Å during the MD simulation, as shown in Figure 4c. However, the distances between other α-helixes/β sheets and SNP are more than 9 Å. All these can be found in Figure 7a and Figure S3a.

However, as shown in Figure 7b and Figure S3b. Our results show that more residues interacted directly with the surface of 11 nm SNP, which include Glu7, Arg10, Thr11, Arg14, Leu15, Tyr20, His78, Gln86, Asn88, Ala90, Asp91, Ala94 and Lys97. Three α-helixes directly interact with 11 nm SNP, which include “KVFERCELARTLKRLG” (residue 1 to 16), “ADAVACAKRVVD” (residue 90 to 101) and “CSAL” (residue 81 to 84). The similar results of the RMSFs of these adsorbed residues can be found in Figure 4c.

Our results show that three enzymes are adsorbed onto the surfaces of 4/11 nm SNPs. This is consistent with other recent simulation reports[15], [32]–[50]: peptides (GAM peptide, dodecamer peptide, α-Helical peptide, RC7 peptide) and proteins (including Frbronection module, Insulin, HP35, WW domains, human serum albumin) are adsorbed onto the surface of graphene, carbon nanotube, C_60_ or gold nanoparticles. Moreover, the conformations of these proteins keep stable after 35 ns (up to 167 ns), which indicates that 100 ns is reasonable for our purpose to validate the interactions between enzymes and SNPs.

### 2 The Conformational Change of the Structures of the Enzymes

The structure of protein determines its function. In this section, we investigate how the interaction with SNPs affects the structures of the enzymes.

#### 2.1 Structural Change of Cytochrome c

**Figure 8:**
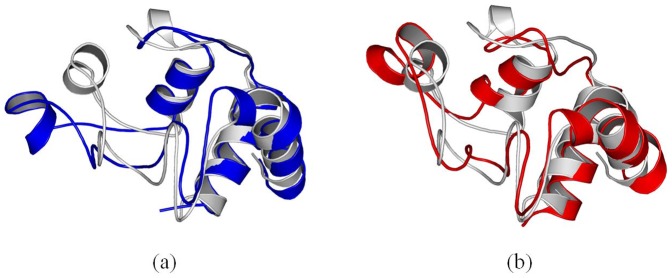
Comparison of the structure of cytochrome c adsorbed onto different diameter of SNPs. (a) align the crystal structure (gray) with the structure adsorbed onto 4 nm SNP (blue), (b) align the crystal structure (gray) with the structure adsorbed onto 11 nm SNP (red). The structures are the conformations after 100 ns MD simulation.

After 100 ns molecular dynamics simulation, we align the crystal structure of cytochrome c with the structure adsorbed onto 4 nm SNP (Figure 8a). We also align the crystal structure with the structure adsorbed onto 11 nm SNP as shown in Figure 8b.

First, our results show that the average RMSD of cytochrome c and the structure adsorbed onto 4 nm SNP is ∼4.56 Å, while the average RMSD of cytochrome c adsorbed onto 11 nm SNP is ∼6.29 Å, as shown in Figure 3a.

Second, our results show that the α-helix consisted of residue 49 to 55(“TAANKNK”) is broken into coil, while the other α-helixes keep stable. The RMSFs of the residues in this α-helix (residue 49 to 55, “TAANKNK”) range from 6.7 to 7.1 Å, as shown in Figure 4a. Moreover, the β sheet consisted of residue 35 to 40 (“LFGRKT”) also breaks into coil in these two simulation, which the RMSFs of the residues are from 6.0 to 6.9 Å, as shown in Figure 4a. Two protein secondary structure prediction servers are used to predict these two structures, including PORTER [51] and PSIPRED [52]. The results show that these structures are not stable for α-helix.

Importantly, our results show that the β sheet (residue 56 to 61, “GIIWGE”) keep stable when it is adsorbed onto 4 nm SNP. However, it breaks into coil when it is absorbs onto 11 nm SNP. The RMSFs of the residues range from 6.4 to 7.3 Å, as shown in Figure 4a.

Indeed, natural proteins undergo conformational changes. For comparisons, we also perform the MD simulations for the crystal structure of cytochrome c with explicit water. Our results show the RMSD of the crystal structure of cytochrome c is ∼3.5 Å (black line), as shown in Figure 3a. The RMSD and the RMSF of the crystal structure of cytochrome c are lower than that adsorbed onto SNPs.

#### 2.2. Structural Change of RNase A

**Figure 9:**
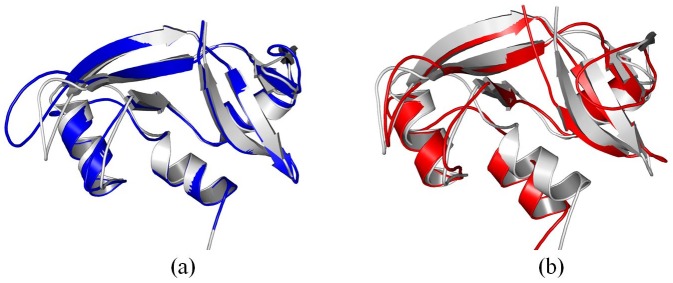
Comparison of the structure of RNase A adsorbed onto different diameter of SNPs. (a) align the crystal structure (gray) with the structure adsorbed onto 4 nm SNP (blue), (b) align the crystal structure (gray) with the structure adsorbed onto 11 nm SNP (red). The structures are the conformations after 100 ns MD simulation.

Figure 9a and 9b show the alignments of different conformations of RNase A. Our results show that the average RMSD of RNase A adsorbed onto the surface of 4 nm SNP is ∼2.66 Å, while the average RMSD of RNase A adsorbed onto 11 nm SNP is 3.97Å, as shown in Figure 3b.

The structure of RNase A adsorbed onto the surface of 4 nm SNPs keeps stable during our 100 ns simulation. Although the α-helixes of RNase A also keep stable when it is adsorbed onto 11 nm SNPs, one β sheet consisted of “QKNVA” (residue 60 to 64) endures large conformational changes, which the RMSFs of these residues range from 4.0 to 4.6 Å (second peak, Figure 4b).

We also perform the MD simulations for the crystal structure of RNase A with explicit water. Our results show the RMSD of the crystal structure of RNase A is ∼2.0 Å (black line), as shown in Figure 3b. The RMSD and RMSF of the crystal structure of RNase A are also lower than that adsorbed onto SNPs.

#### 2.3. Structural Change of Lysozyme

Figure 3c shows that the average RMSD of lysozyme adsorbed onto 4 nm SNP is ∼4.15 Å, while the average RMSD of lysozyme adsorbed onto 11 nm SNP is 5.37Å.

**Figure 10:**
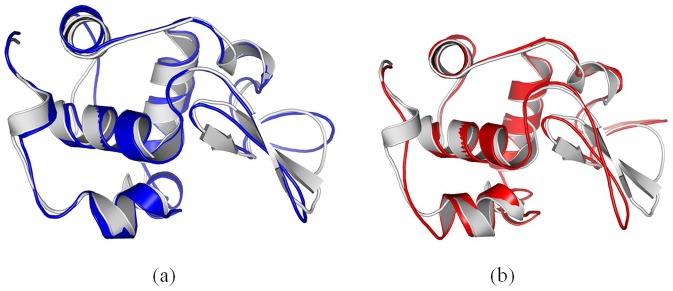
Comparison of the structure of lysozyme adsorbed onto different diameter of SNPs. (a) align the crystal structure (gray) with the structure adsorbed onto 4 nm SNP (blue), (b) align the crystal structure (gray) with the structure adsorbed onto 11 nm SNP (red). The structures are the conformations after 100 ns MD simulation.

Figure 10a shows that the structure of lysozyme adsorbed onto 4 nm SNPs keeps stable during simulation.

However, more conformational changes of lysozyme can be found after it is absorbed onto the surface of 11 nm SNP. Figure 10b shows that a α-helix is not stable, which is consisted of residue 80 to 85 (“SCSALL”). The RMSFs of these residues range from 4.8 to 6.5 Å, as shown in Figure 4c. Two β sheets also endure large change, including “ATNYN” (residue 42 to 46) and “RSTDY” (residue 50 to 54), which the RMSFs of these residues are larger than 4.7 Å, as shown in Figure 4c

The RMSD of the crystal structure of lysozyme is ∼2.7 Å (black line), as shown in Figure 3c. Most values of RMSFS are lower than that of lysozyme adsorbed onto SNPs.

In summary, our results show that all three enzymes adsorbed onto 4 nm SNPs make less structural changes. The three enzymes adsorbed onto 11 nm SNPs make more structural changes. Moreover, the crystal structures of enzymes are more stable than the enzymes adsorbed onto SNPs.

### 3 Conformational Change of Active Site of Cytochrome c

In this section, we investigate the conformational change of active site of cytochrome c, which affects the specificity and efficiency of the enzyme directly.

**Figure 11:**
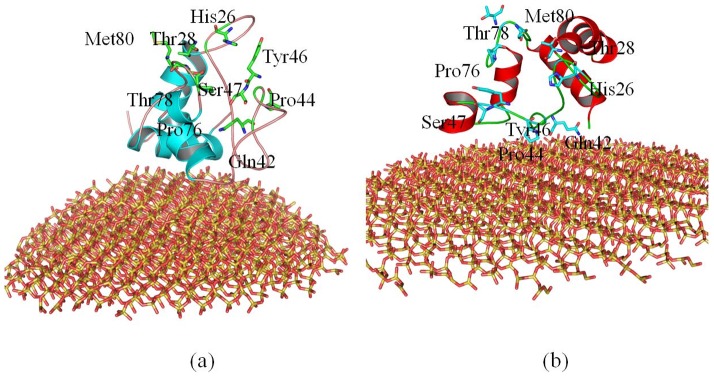
The active site of cytochrome c after it absorbs onto (a) 4 nm SNP, (b) 11 nm SNP. The structures are the conformations after 100 ns MD simulation.

Figure 11 compares the active site of cytochrome c after it absorbs onto 4 nm SNP or 11 nm SNP.

As shown in Figure 11a, our results show that most of the residues from the active site are far away from the surface of 4 nm SNP, which include His26, Thr28, Gln42, Pro44, Tyr46, Ser47, Pro76, Thr78 and Met80. The distances between these residues and the surface of SNPs are more than 10 Å. The average center-of-mass (COM) distance between the active site of cytochrome c and the 4 nm SNPs is 8.7 Å.

However, as shown in Figure 11b, we can find that some important residues of the active site are directly absorbed onto the surface of 11 nm SNP, which include Gln42, Pro44, Tyr46 and Ser47 (also include residue 38 to 41). The average center-of-mass (COM) distance between the active site of cytochrome c and the 11 nm SNP is 4.6 Å.

We try to construct 3D models for cytochrome c - heme by using several docking programs, which include Glide in Schrödinger, GOLD, LigandFit, LibDock, Flexible Docking and CDocker program in Discovery Studio 2.5. We cannot construct the 3D models due to the conformation change of the active site. Thus we investigate the conformation changes and volumes of active site of cytochrome c.

**Figure 12:**
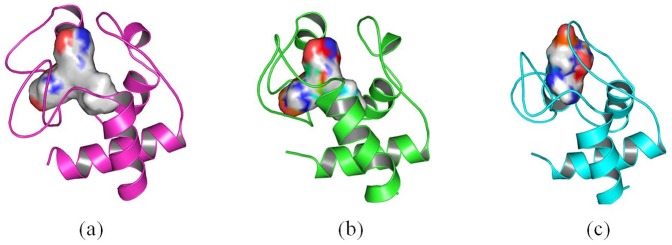
Conformation changes of active site of cytochrome c. (a) active site of cytochrome c in the crystal structure, (b) active site of cytochrome c after it absorbs onto 4 nm SNP (after 100 ns MD simulation), (c) active site of cytochrome c after it absorbs onto 11 nm SNP (after 100 ns MD simulation).

Our results show that the structure of cytochrome c adsorbed onto 4 nm SNP endures conformational change. However, the binding pocket is similar as that of the crystal structure. The volumes of active site of cytochrome c adsorbed onto 4 nm SNP is ∼179 Å^3^ (Figure 12a), while the volumes of active site of crystal structure of cytochrome c is ∼167 Å^3^ (Figure 12b).

However, as shown in Figure 12c, our results show that the structure of cytochrome c adsorbed onto 11 nm SNP endures significant changes. The binding pocket is covered and not suitable for the docking of ligands. The volumes of active site of cytochrome c adsorbed onto 11 nm SNP is only ∼98 Å^3^.

In summary, the active site of cytochrome c endures conformational change after it adsorbs onto the 4 nm SNP or 11 nm SNP. Moreover, the active site makes more conformational change after cytochrome c absorbs onto 11 nm SNP and is not available for the ligands.

### 4 Conformational Change of Active Site of RNase A

**Figure 13:**
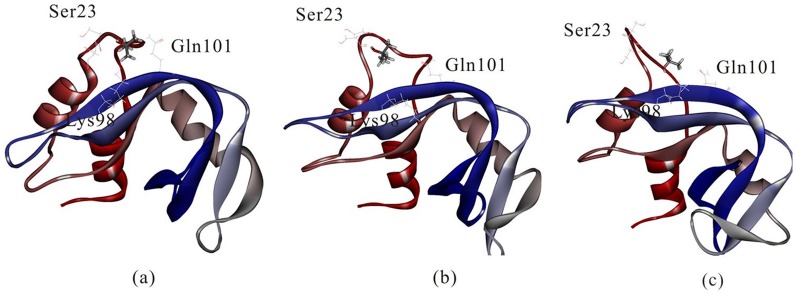
The binding mode of TBU (tertiary-butyl alcohol, highlighted in the stick model) in (a) the crystal structure of RNase A, (b) the structure of RNase A adsorbed onto 4 nm SNP (after 100 ns MD simulation), (c) the structure of RNase A adsorbed onto 11 nm SNP (after 100 ns MD simulation).

We investigate the conformational change of active site of RNase A by docking TBU (tertiary-butyl alcohol), as shown in Figure 13.

Figure 13a shows the docking result of TBU in the crystal structure of RNase A, which is consistent with the crystal structure of RNase A-TBU complex. Figure 13b and 13c show the docking results of TBU after RNase A absorbs onto 4 nm SNP or 11 nm SNP.

Figure 13b shows that RNase A endures some but not significant conformational changes when it is absorbed onto 4 nm SNP, including the changes of α-helixes and the β sheets. Our results show that Lys98 is far away from the ligand-TBU, but the ligand strongly interacts with residue Ser23 and Gln101. The binding mode of TBU is similar as the crystal structure.

However, the situation is very different for RNase A adsorbs onto 11 nm SNP, as shown in Figure 13c. Significant conformational changes are found, especially for the β sheet and coil involved in the active site. Our results show that Lys98 and Ser23 are far away from the ligand-TBU. The binding mode of TBU is very different from the crystal structure.

Based on the results of cytochrome c and RNase A, we find that the small SNPs induce greater structural stabilization, which is in agreement with recent study. [12]

### 5 Influences of Different Chemical Groups Coated onto Silica Nanoparticles

**Figure 14:**
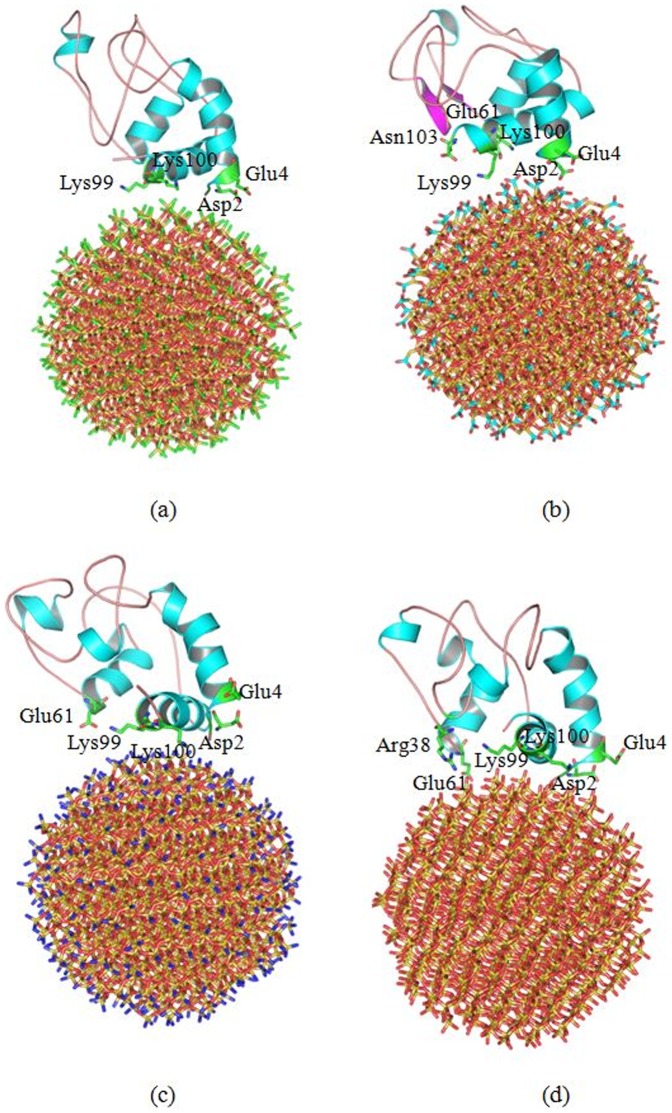
Cytochrome c adsorbed onto 2 nm SNPs coated with (a)-CH_3_, (b)-COOH, (c)-NH_2_ and (d)-OH. The structures are the conformations after 100 ns MD simulation.

In order to investigate the effect of the chemical group coated on SNPs, we perform two independent molecular dynamics simulations of cytochrome c adsorbed onto 2 nm SNP coat with different chemical groups (-CH_3_, -COOH, -NH_2_, -OH), as shown in Figure 14.

**Figure 15:**
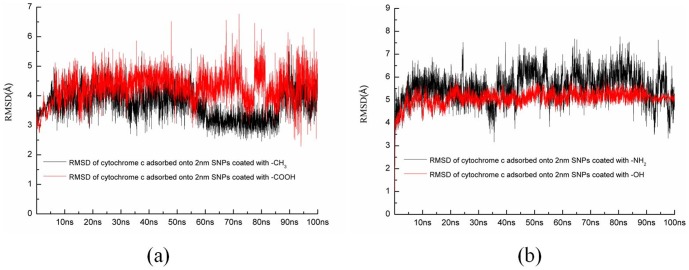
Time evolutions for cytochrome c adsorbed onto different chemical groups coated onto silica nano-particles during 100 ns MD simulations. (a) RMSD of cytochrome c adsorbed onto SNPs coated with -CH_3_ and with -COOH, (b) RMSD of cytochrome c adsorbed onto SNPs coated with -NH_2_ and with -OH.

Coated with -CH_3_(Figure 14a), our results show several residues directly adsorb onto the surface of SNP, which including Gly1, Asp2, Val3, Glu4, Ala96, Lys99 and Lys100. All these residues form hydrophobic interactions with the surface of SNP. Moreover, the active site of cytochrome c is far away from the surface of SNP. The RMSD of cytochrome c adsorbed onto the surface of SNP coated with -CH_3_ is 3.66 Å, as shown in Figure 15a.

Coated with -COOH(Figure 14b), residues interacted with the surface of SNP are all hydrophilic residues, which include Gly1, Asp2, Glu4, Glu61, Lys99, Lys100 and Asn103. These residues form hydrogen bonds with the carboxyl groups. Moreover, our results show that the active site of cytochrome c is far away from the surface of SNP. The RMSD of cytochrome c adsorbed onto the surface of SNP coated with -COOH is 4.57 Å, as shown in Figure 15b.

Coated with -NH_2_(Figure 14c), our results show the similar results with that coat with -COOH. However, the structure of cytochrome c endures large conformational changes. The RMSD of cytochrome c adsorbed onto the surface of SNP coated with -NH_2_ is 5.49 Å, as shown in Figure 15c.

**Figure 16:**
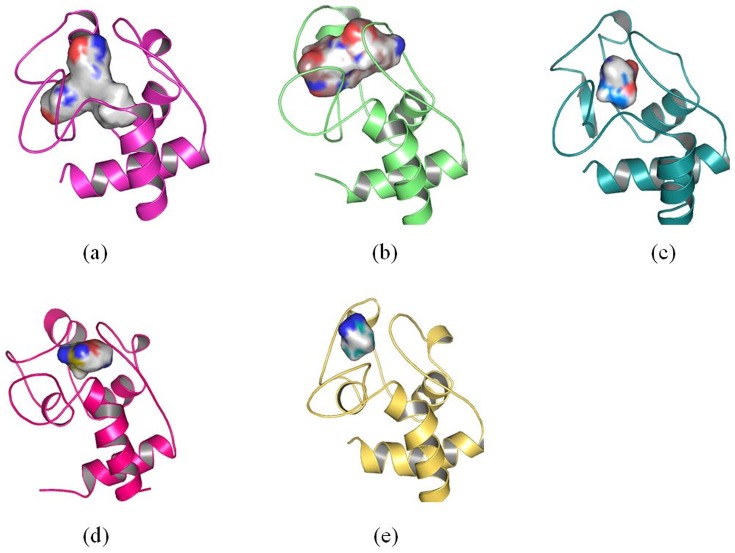
Conformation changes of active site of cytochrome c. (a) active site of cytochrome c of the crystal structure, (b) active site of cytochrome c after it adsorbs onto 2 nm SNP coated with -CH_3_, (c) active site of cytochrome c after it adsorbs onto 2 nm SNP coated with -COOH, (d) active site of cytochrome c after it absorbs onto 2 nm SNP coated with -NH_2_, (e) active site of cytochrome c after it adsorbs onto 2 nm SNP coated with -OH. The structures of (b), (c), (d), (e) are the conformations after 100 ns MD simulation.

Coated with -OH(Figure 14d), Gly1, Asp2, Glu4, Arg38, Glu61, Lys99, Lys100 and Asn103 contribute the hydrophilic interactions with the surface of SNP. The structure also endures large conformational changes. The RMSD of cytochrome c adsorbed onto the surface of SNP coated with -OH is 5.16 Å, as shown in Figure 15d. In order to further study the conformational change of cytochrome c adsorbed onto 2 nm SNPs coated with different chemical groups, we visualize the active site of cytochrome c, as shown in Figure 16.

From Figure 16, we can see clearly that the structure of cytochrome c endure different degrees of conformational changes, especially for the active sites of cytochrome c. The active site of cytochrome c adsorbed onto SNP coated with -COOH endure the least conformational change.

In summary, the active site of cytochrome c adsorbed onto the 2 nm SNPs coated with different groups is far away from the surface of SNPs. but it shows different degree of conformational changes. It endures less conformational changes when it interacts with SNP coated with -COOH.

## Conclusion

Nanoscale particles have become promising materials in many fields, such as cancer therapeutics, diagnosis, imaging, drug delivery, catalysis, as well as biosensors, in order to stimulate and facilitate these applications, there is an urgent need for the understanding of the nanoparticle toxicity and other risks involved with these nanoparticles to human health. Particularly, very little is known about exactly how a protein interacts with nano-particles and how its orientation is governed by the size, shape, and chemistry of the surface of the nano-particles.

In this study, we investigate the orientation and adsorption between several enzymes (cytochrome c/RNase A/lysozyme) and 4 nm/11 nm silica nanoparticles (SNPs) by using molecular dynamics (MD) simulation. Our results show that three enzymes absorb onto the surfaces of both 4 nm and 11 nm SNPs.Moreover, the small SNPs induce greater structural stabilization, which is consistent with experimental results. The active site of cytochrome c is far away from the surface of 4 nm SNP, while it adsorbs onto the surface of 11 nm SNP. Importantly, after it absorbs onto 11 nm SNP, the active site of cytochrome c is not available for ligands. We also explore the influences of different chemical groups (-OH, -COOH, -NH_2_ and CH_3_) coated onto silica nanoparticles. The active site of cytochrome c adsorbed onto the 2 nm SNPs coated with different groups is far away from the surface of SNPs, but it shows different degree of conformational changes. The active site of cytochrome c endures less conformational changes when SNP was coated with -COOH. Our molecular dynamics results indicate the selective interaction between silicon nano-particles and enzymes, which is consistent with experimental results.

## Supporting Information

File S1 **Supplementary Information.**
(DOC)
