# Supplementary material for: The Selective Interaction between Silica Nanoparticles and Enzymes from Molecular Dynamics Simulations
Source: PLoS One. 2014 Sep 22;9(9):e107696. doi: 10.1371/journal.pone.0107696 (PMC4171504; doi:10.1371/journal.pone.0107696)
Supplement: File S1 — Supplementary Information. (DOC) [file pone.0107696.s001.doc]

**Supplementary Information**

**The selective interaction between Silica Nanoparticles and Enzymes from Molecular Dynamics Simulations**

Xiaotian Sun†, Zhiwei Feng†, Liling Zhang, Tingjun Hou, Youyong Li^*^

Institute of Functional Nano & Soft Materials (FUNSOM) and Collaborative Innovation Center of Suzhou Nano Science and Technology, Soochow University, Suzhou 215123, China

† Equal contribution

Email: [yyli@suda.edu.cn](mailto:yyli@suda.edu.cn)

We perform geometry optimization on the SNPs by Material Studio 5.5 with Forcite module. Dreiding force field, Gasteiger (maximum iteration is setting 50,000, convergence limit is setting 5.0e-6 e) and ultra-fine quality are used for the energy calculation. Then we cut the SNPs by using the central angle of 60 degree.

Two independent MD simulations of each system are performed using the NAMD package (version 2.7b2) with CHARMM27 force field for the studied complex with explicit water. In addition, Dreiding force field (K_b_ value of Si is 350) is used for the SNPs. Electrostatics are calculated using the Particle Mesh Ewald (PME) method with a 12 Å non-bonded cutoff and a grid spacing of 1 Å per grid point in each dimension. The van der Waals energies are calculated using a smooth cutoff (switching radius 10 Å, cutoff radius 12 Å). The temperature and pressure are kept constant using a langevin thermostat (310 K) and langevin barostat (1 atm), respectively. The time step of MD simulations is set to 1 fs. The data is saved every 10 ps for analysis.


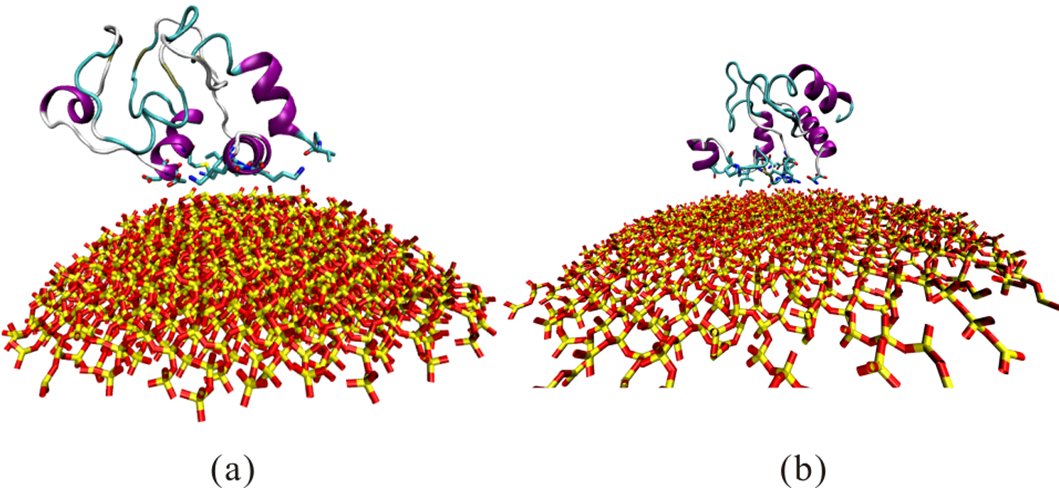


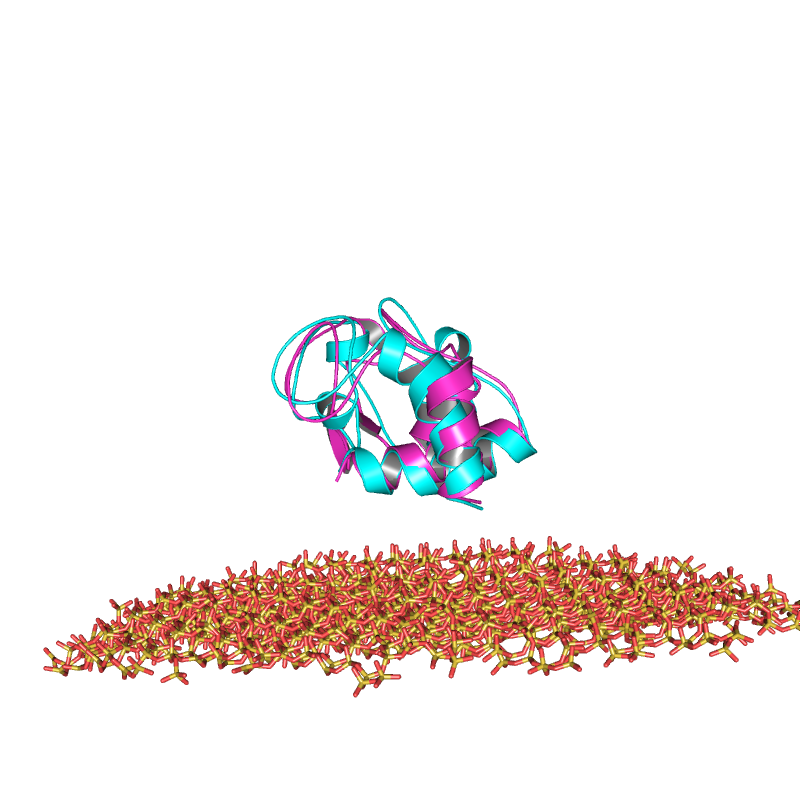


(c)

Figure S1. The structure of cytochrome c adsorbed onto the surface of SNPs. (a) with 4nm SNP, (b) with 11nm SNP. (c) the alignments of conformation of cytochrome c between 35^th^ ns and 167^th^ns.


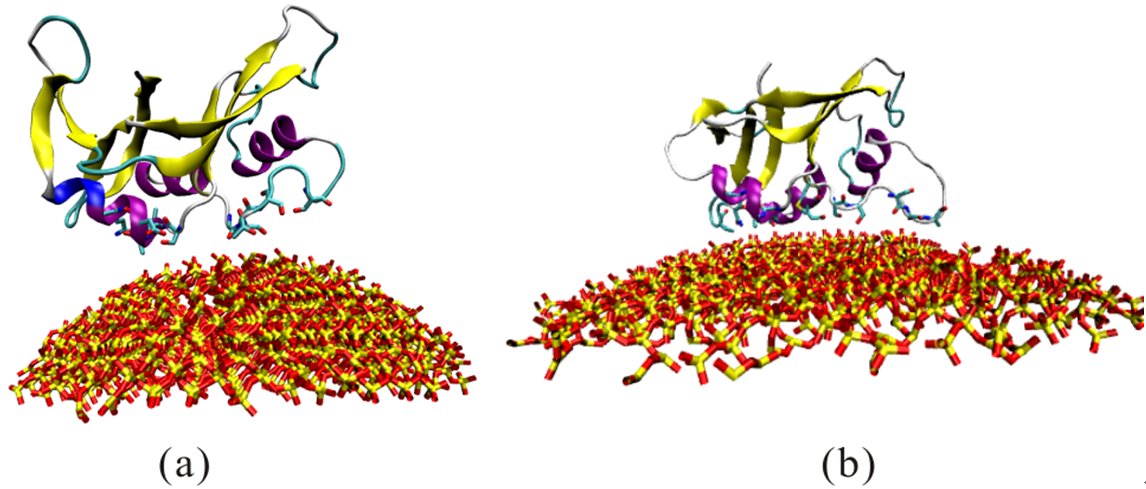


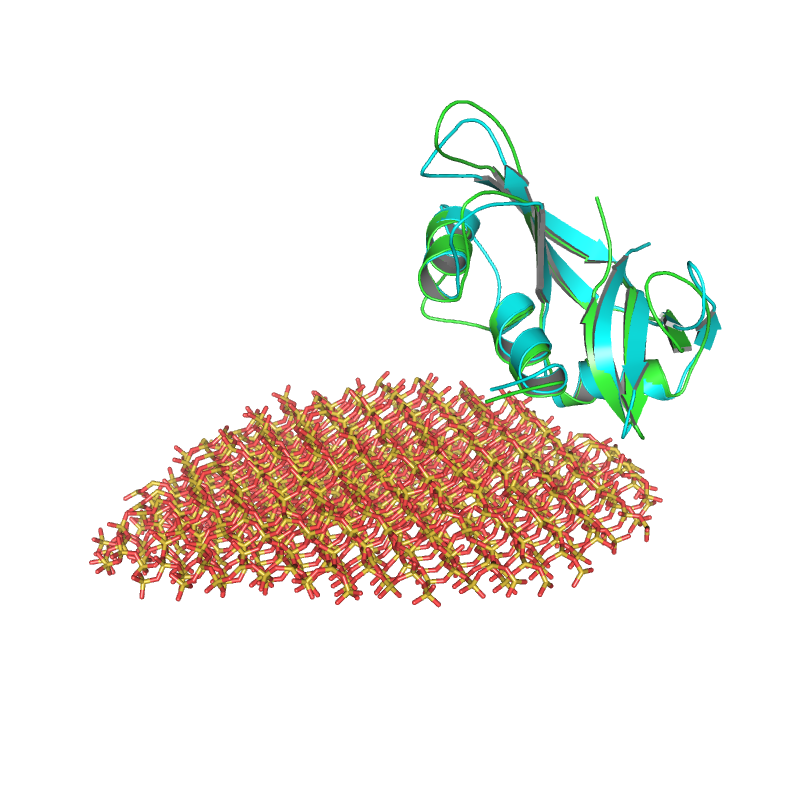


(c)

Figure S2. The structure of of RNase A adsorbed onto the surface of SNPs. (a) with 4nm SNP, (b) with 11nm SNP. (c) the alignments of conformation of RNase A between 35^th^ ns and 133^th^ns.


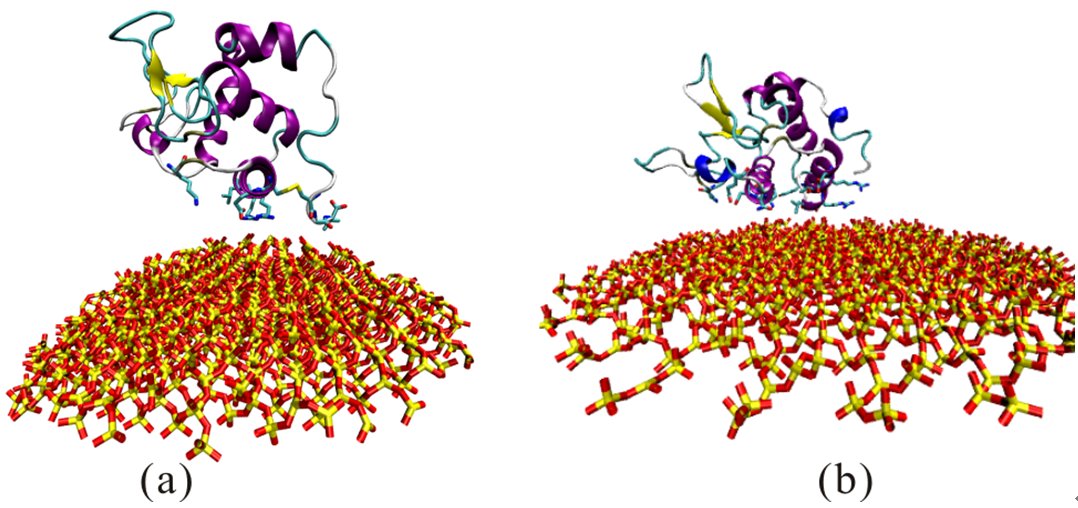


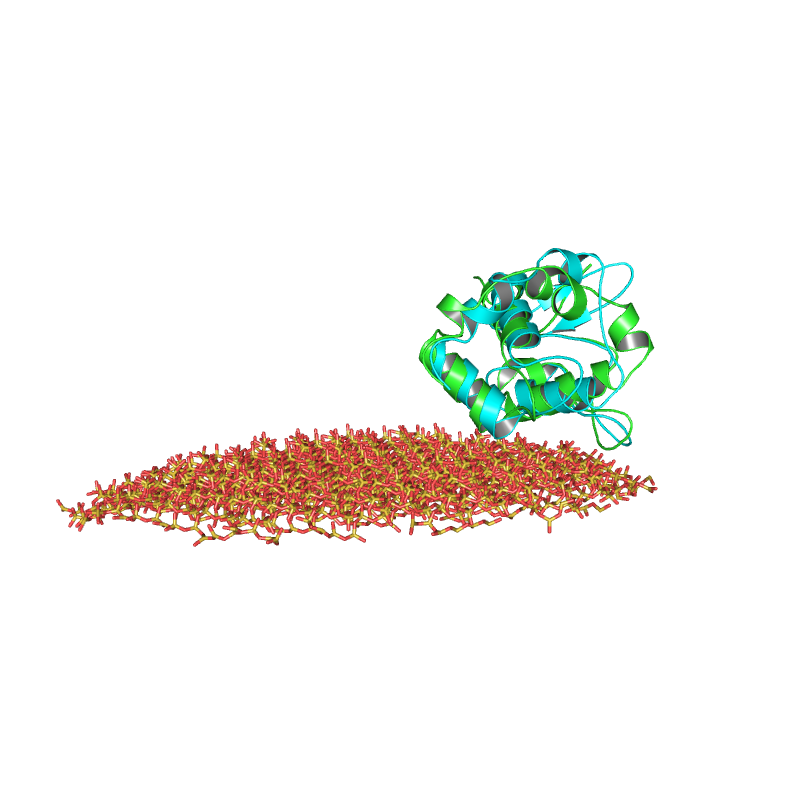


(c)

Figure S3. The structure of lysozyme adsorbed onto the surface of SNPs. (a) with 4nm SNP, (b) with 11nm SNP. (c) the alignments of conformation of lysozyme between 35^th^ ns and 145^th^ns.

***Parameters for enzymes and SNPs:***

**coordinates of cytochrome c**

CRYST1 100.000 100.000 100.000 90.00 90.00 90.00 P1

ATOM 1 N GLY A 1 4.865 -9.580 -4.769 1.00 0.00 A2 N

ATOM 2 HT1 GLY A 1 5.731 -9.087 -4.688 0.00 0.00 A2 H

ATOM 3 HT2 GLY A 1 5.008 -10.442 -5.255 0.00 0.00 A2 H

ATOM 4 HT3 GLY A 1 4.489 -9.760 -3.860 0.00 0.00 A2 H

ATOM 5 CA GLY A 1 3.892 -8.737 -5.543 1.00 0.00 A2 C

ATOM 6 HA1 GLY A 1 4.313 -8.586 -6.528 0.00 0.00 A2 H

ATOM 7 HA2 GLY A 1 3.735 -7.826 -4.982 0.00 0.00 A2 H

ATOM 8 C GLY A 1 2.548 -9.423 -5.707 1.00 0.00 A2 C

ATOM 9 O GLY A 1 1.774 -9.063 -6.588 1.00 0.00 A2 O

ATOM 10 N ASP A 2 2.276 -10.403 -4.841 1.00 0.00 A2 N

ATOM 11 HN ASP A 2 2.784 -10.539 -3.994 0.00 0.00 A2 H

ATOM 12 CA ASP A 2 1.234 -11.362 -5.026 1.00 0.00 A2 C

ATOM 13 HA ASP A 2 0.855 -11.267 -6.037 0.00 0.00 A2 H

ATOM 14 CB ASP A 2 1.763 -12.785 -4.707 1.00 0.00 A2 C

ATOM 15 HB1 ASP A 2 2.544 -13.058 -5.445 0.00 0.00 A2 H

ATOM 16 HB2 ASP A 2 2.213 -12.817 -3.695 0.00 0.00 A2 H

ATOM 17 CG ASP A 2 0.652 -13.886 -4.783 1.00 0.00 A2 C

ATOM 18 OD1 ASP A 2 -0.542 -13.592 -4.545 1.00 0.00 A2 O

ATOM 19 OD2 ASP A 2 0.975 -15.060 -5.075 1.00 0.00 A2 O

ATOM 20 C ASP A 2 0.090 -10.993 -4.092 1.00 0.00 A2 C

ATOM 21 O ASP A 2 0.224 -11.173 -2.851 1.00 0.00 A2 O

ATOM 22 N VAL A 3 -1.030 -10.527 -4.679 1.00 0.00 A2 N

ATOM 23 HN VAL A 3 -1.254 -10.567 -5.649 0.00 0.00 A2 H

ATOM 24 CA VAL A 3 -2.058 -9.885 -3.844 1.00 0.00 A2 C

ATOM 25 HA VAL A 3 -1.538 -9.252 -3.136 0.00 0.00 A2 H

ATOM 26 CB VAL A 3 -2.948 -8.904 -4.610 1.00 0.00 A2 C

ATOM 27 HB VAL A 3 -3.637 -8.426 -3.871 0.00 0.00 A2 H

ATOM 28 CG1 VAL A 3 -2.055 -7.789 -5.299 1.00 0.00 A2 C

ATOM 29 1HG1 VAL A 3 -2.692 -7.095 -5.889 0.00 0.00 A2 H

ATOM 30 2HG1 VAL A 3 -1.510 -7.179 -4.549 0.00 0.00 A2 H

ATOM 31 3HG1 VAL A 3 -1.316 -8.249 -5.989 0.00 0.00 A2 H

ATOM 32 CG2 VAL A 3 -3.838 -9.602 -5.635 1.00 0.00 A2 C

ATOM 33 1HG2 VAL A 3 -4.441 -8.861 -6.201 0.00 0.00 A2 H

ATOM 34 2HG2 VAL A 3 -3.222 -10.178 -6.359 0.00 0.00 A2 H

ATOM 35 3HG2 VAL A 3 -4.544 -10.303 -5.143 0.00 0.00 A2 H

ATOM 36 C VAL A 3 -2.858 -10.888 -3.052 1.00 0.00 A2 C

ATOM 37 O VAL A 3 -3.405 -10.579 -1.996 1.00 0.00 A2 O

ATOM 38 N GLU A 4 -2.897 -12.116 -3.547 1.00 0.00 A2 N

ATOM 39 HN GLU A 4 -2.461 -12.443 -4.380 0.00 0.00 A2 H

ATOM 40 CA GLU A 4 -3.648 -13.138 -2.831 1.00 0.00 A2 C

ATOM 41 HA GLU A 4 -4.609 -12.728 -2.547 0.00 0.00 A2 H

ATOM 42 CB GLU A 4 -3.853 -14.328 -3.742 1.00 0.00 A2 C

ATOM 43 HB1 GLU A 4 -4.199 -13.952 -4.732 0.00 0.00 A2 H

ATOM 44 HB2 GLU A 4 -2.878 -14.831 -3.931 0.00 0.00 A2 H

ATOM 45 CG GLU A 4 -4.865 -15.327 -3.202 1.00 0.00 A2 C

ATOM 46 HG1 GLU A 4 -4.377 -15.980 -2.455 0.00 0.00 A2 H

ATOM 47 HG2 GLU A 4 -5.722 -14.808 -2.728 0.00 0.00 A2 H

ATOM 48 CD GLU A 4 -5.368 -16.231 -4.305 1.00 0.00 A2 C

ATOM 49 OE1 GLU A 4 -5.343 -15.788 -5.482 1.00 0.00 A2 O

ATOM 50 OE2 GLU A 4 -5.795 -17.373 -3.991 1.00 0.00 A2 O

ATOM 51 C GLU A 4 -2.908 -13.520 -1.527 1.00 0.00 A2 C

ATOM 52 O GLU A 4 -3.499 -13.620 -0.441 1.00 0.00 A2 O

ATOM 53 N LYS A 5 -1.601 -13.695 -1.656 1.00 0.00 A2 N

ATOM 54 HN LYS A 5 -1.132 -13.611 -2.534 0.00 0.00 A2 H

ATOM 55 CA LYS A 5 -0.746 -14.023 -0.530 1.00 0.00 A2 C

ATOM 56 HA LYS A 5 -1.194 -14.846 0.013 0.00 0.00 A2 H

ATOM 57 CB LYS A 5 0.646 -14.419 -1.002 1.00 0.00 A2 C

ATOM 58 HB1 LYS A 5 0.919 -13.728 -1.833 0.00 0.00 A2 H

ATOM 59 HB2 LYS A 5 1.375 -14.226 -0.183 0.00 0.00 A2 H

ATOM 60 CG LYS A 5 0.693 -15.892 -1.490 1.00 0.00 A2 C

ATOM 61 HG1 LYS A 5 0.371 -16.561 -0.660 0.00 0.00 A2 H

ATOM 62 HG2 LYS A 5 -0.036 -16.022 -2.323 0.00 0.00 A2 H

ATOM 63 CD LYS A 5 2.072 -16.318 -1.944 1.00 0.00 A2 C

ATOM 64 HD1 LYS A 5 2.457 -15.557 -2.661 0.00 0.00 A2 H

ATOM 65 HD2 LYS A 5 2.747 -16.311 -1.057 0.00 0.00 A2 H

ATOM 66 CE LYS A 5 2.010 -17.708 -2.579 1.00 0.00 A2 C

ATOM 67 HE1 LYS A 5 1.347 -17.699 -3.473 0.00 0.00 A2 H

ATOM 68 HE2 LYS A 5 3.024 -18.038 -2.896 0.00 0.00 A2 H

ATOM 69 NZ LYS A 5 1.473 -18.776 -1.648 1.00 0.00 A2 N

ATOM 70 HZ1 LYS A 5 1.453 -19.695 -2.134 0.00 0.00 A2 H

ATOM 71 HZ2 LYS A 5 2.082 -18.843 -0.808 0.00 0.00 A2 H

ATOM 72 HZ3 LYS A 5 0.509 -18.523 -1.351 0.00 0.00 A2 H

ATOM 73 C LYS A 5 -0.717 -12.833 0.399 1.00 0.00 A2 C

ATOM 74 O LYS A 5 -0.773 -13.013 1.598 1.00 0.00 A2 O

ATOM 75 N GLY A 6 -0.677 -11.609 -0.160 1.00 0.00 A2 N

ATOM 76 HN GLY A 6 -0.715 -11.432 -1.143 0.00 0.00 A2 H

ATOM 77 CA GLY A 6 -0.571 -10.426 0.691 1.00 0.00 A2 C

ATOM 78 HA1 GLY A 6 -0.454 -9.560 0.054 0.00 0.00 A2 H

ATOM 79 HA2 GLY A 6 0.241 -10.577 1.389 0.00 0.00 A2 H

ATOM 80 C GLY A 6 -1.863 -10.249 1.500 1.00 0.00 A2 C

ATOM 81 O GLY A 6 -1.815 -9.866 2.670 1.00 0.00 A2 O

ATOM 82 N LYS A 7 -3.019 -10.550 0.892 1.00 0.00 A2 N

ATOM 83 HN LYS A 7 -3.103 -10.840 -0.060 0.00 0.00 A2 H

ATOM 84 CA LYS A 7 -4.290 -10.464 1.634 1.00 0.00 A2 C

ATOM 85 HA LYS A 7 -4.397 -9.441 1.974 0.00 0.00 A2 H

ATOM 86 CB LYS A 7 -5.509 -10.776 0.758 1.00 0.00 A2 C

ATOM 87 HB1 LYS A 7 -5.543 -10.002 -0.044 0.00 0.00 A2 H

ATOM 88 HB2 LYS A 7 -5.347 -11.751 0.245 0.00 0.00 A2 H

ATOM 89 CG LYS A 7 -6.861 -10.770 1.546 1.00 0.00 A2 C

ATOM 90 HG1 LYS A 7 -6.882 -11.641 2.240 0.00 0.00 A2 H

ATOM 91 HG2 LYS A 7 -6.914 -9.848 2.169 0.00 0.00 A2 H

ATOM 92 CD LYS A 7 -8.070 -10.848 0.631 1.00 0.00 A2 C

ATOM 93 HD1 LYS A 7 -7.996 -10.029 -0.121 0.00 0.00 A2 H

ATOM 94 HD2 LYS A 7 -8.024 -11.814 0.077 0.00 0.00 A2 H

ATOM 95 CE LYS A 7 -9.400 -10.743 1.440 1.00 0.00 A2 C

ATOM 96 HE1 LYS A 7 -9.540 -11.643 2.079 0.00 0.00 A2 H

ATOM 97 HE2 LYS A 7 -9.389 -9.845 2.096 0.00 0.00 A2 H

ATOM 98 NZ LYS A 7 -10.609 -10.634 0.596 1.00 0.00 A2 N

ATOM 99 HZ1 LYS A 7 -11.457 -10.569 1.195 0.00 0.00 A2 H

ATOM 100 HZ2 LYS A 7 -10.541 -9.784 0.000 0.00 0.00 A2 H

ATOM 101 HZ3 LYS A 7 -10.682 -11.474 -0.014 0.00 0.00 A2 H

ATOM 102 C LYS A 7 -4.252 -11.359 2.879 1.00 0.00 A2 C

ATOM 103 O LYS A 7 -4.733 -10.988 3.958 1.00 0.00 A2 O

ATOM 104 N LYS A 8 -3.715 -12.557 2.708 1.00 0.00 A2 N

ATOM 105 HN LYS A 8 -3.335 -12.883 1.844 0.00 0.00 A2 H

ATOM 106 CA LYS A 8 -3.644 -13.510 3.816 1.00 0.00 A2 C

ATOM 107 HA LYS A 8 -4.638 -13.574 4.241 0.00 0.00 A2 H

ATOM 108 CB LYS A 8 -3.332 -14.920 3.229 1.00 0.00 A2 C

ATOM 109 HB1 LYS A 8 -4.146 -15.162 2.507 0.00 0.00 A2 H

ATOM 110 HB2 LYS A 8 -2.393 -14.865 2.634 0.00 0.00 A2 H

ATOM 111 CG LYS A 8 -3.232 -16.066 4.198 1.00 0.00 A2 C

ATOM 112 HG1 LYS A 8 -3.081 -17.012 3.630 0.00 0.00 A2 H

ATOM 113 HG2 LYS A 8 -2.335 -15.919 4.842 0.00 0.00 A2 H

ATOM 114 CD LYS A 8 -4.490 -16.169 5.034 1.00 0.00 A2 C

ATOM 115 HD1 LYS A 8 -4.299 -16.878 5.872 0.00 0.00 A2 H

ATOM 116 HD2 LYS A 8 -4.691 -15.169 5.483 0.00 0.00 A2 H

ATOM 117 CE LYS A 8 -5.733 -16.630 4.236 1.00 0.00 A2 C

ATOM 118 HE1 LYS A 8 -5.671 -16.281 3.181 0.00 0.00 A2 H

ATOM 119 HE2 LYS A 8 -5.807 -17.740 4.235 0.00 0.00 A2 H

ATOM 120 NZ LYS A 8 -6.978 -16.061 4.861 1.00 0.00 A2 N

ATOM 121 HZ1 LYS A 8 -7.817 -16.367 4.327 0.00 0.00 A2 H

ATOM 122 HZ2 LYS A 8 -7.055 -16.391 5.844 0.00 0.00 A2 H

ATOM 123 HZ3 LYS A 8 -6.925 -15.022 4.853 0.00 0.00 A2 H

ATOM 124 C LYS A 8 -2.692 -13.013 4.937 1.00 0.00 A2 C

ATOM 125 O LYS A 8 -3.001 -13.129 6.125 1.00 0.00 A2 O

ATOM 126 N ILE A 9 -1.562 -12.419 4.561 1.00 0.00 A2 N

ATOM 127 HN ILE A 9 -1.265 -12.358 3.611 0.00 0.00 A2 H

ATOM 128 CA ILE A 9 -0.646 -11.796 5.517 1.00 0.00 A2 C

ATOM 129 HA ILE A 9 -0.433 -12.546 6.268 0.00 0.00 A2 H

ATOM 130 CB ILE A 9 0.591 -11.282 4.750 1.00 0.00 A2 C

ATOM 131 HB ILE A 9 0.167 -10.724 3.877 0.00 0.00 A2 H

ATOM 132 CG2 ILE A 9 1.501 -10.455 5.695 1.00 0.00 A2 C

ATOM 133 1HG2 ILE A 9 2.307 -9.953 5.121 0.00 0.00 A2 H

ATOM 134 2HG2 ILE A 9 0.918 -9.667 6.216 0.00 0.00 A2 H

ATOM 135 3HG2 ILE A 9 1.974 -11.105 6.461 0.00 0.00 A2 H

ATOM 136 CG1 ILE A 9 1.362 -12.478 4.197 1.00 0.00 A2 C

ATOM 137 1HG1 ILE A 9 1.897 -12.996 5.024 0.00 0.00 A2 H

ATOM 138 2HG1 ILE A 9 0.637 -13.207 3.767 0.00 0.00 A2 H

ATOM 139 CD ILE A 9 2.374 -12.066 3.094 1.00 0.00 A2 C

ATOM 140 HD1 ILE A 9 2.875 -12.965 2.674 0.00 0.00 A2 H

ATOM 141 HD2 ILE A 9 1.856 -11.537 2.265 0.00 0.00 A2 H

ATOM 142 HD3 ILE A 9 3.159 -11.396 3.504 0.00 0.00 A2 H

ATOM 143 C ILE A 9 -1.324 -10.609 6.248 1.00 0.00 A2 C

ATOM 144 O ILE A 9 -1.280 -10.502 7.493 1.00 0.00 A2 O

ATOM 145 N PHE A 10 -2.009 -9.755 5.481 1.00 0.00 A2 N

ATOM 146 HN PHE A 10 -2.048 -9.782 4.483 0.00 0.00 A2 H

ATOM 147 CA PHE A 10 -2.781 -8.677 6.086 1.00 0.00 A2 C

ATOM 148 HA PHE A 10 -2.072 -8.033 6.592 0.00 0.00 A2 H

ATOM 149 CB PHE A 10 -3.596 -7.872 5.046 1.00 0.00 A2 C

ATOM 150 HB1 PHE A 10 -2.901 -7.449 4.288 0.00 0.00 A2 H

ATOM 151 HB2 PHE A 10 -4.312 -8.527 4.504 0.00 0.00 A2 H

ATOM 152 CG PHE A 10 -4.304 -6.743 5.654 1.00 0.00 A2 C

ATOM 153 CD1 PHE A 10 -3.614 -5.566 5.930 1.00 0.00 A2 C

ATOM 154 HD1 PHE A 10 -2.564 -5.490 5.681 0.00 0.00 A2 H

ATOM 155 CE1 PHE A 10 -4.265 -4.487 6.530 1.00 0.00 A2 C

ATOM 156 HE1 PHE A 10 -3.739 -3.555 6.682 0.00 0.00 A2 H

ATOM 157 CZ PHE A 10 -5.600 -4.619 6.925 1.00 0.00 A2 C

ATOM 158 HZ PHE A 10 -6.097 -3.796 7.418 0.00 0.00 A2 H

ATOM 159 CD2 PHE A 10 -5.644 -6.865 6.078 1.00 0.00 A2 C

ATOM 160 HD2 PHE A 10 -6.166 -7.803 5.955 0.00 0.00 A2 H

ATOM 161 CE2 PHE A 10 -6.288 -5.793 6.678 1.00 0.00 A2 C

ATOM 162 HE2 PHE A 10 -7.330 -5.877 6.954 0.00 0.00 A2 H

ATOM 163 C PHE A 10 -3.723 -9.240 7.159 1.00 0.00 A2 C

ATOM 164 O PHE A 10 -3.732 -8.777 8.279 1.00 0.00 A2 O

ATOM 165 N ILE A 11 -4.529 -10.215 6.806 1.00 0.00 A2 N

ATOM 166 HN ILE A 11 -4.537 -10.644 5.905 0.00 0.00 A2 H

ATOM 167 CA ILE A 11 -5.507 -10.764 7.738 1.00 0.00 A2 C

ATOM 168 HA ILE A 11 -6.136 -9.935 8.036 0.00 0.00 A2 H

ATOM 169 CB ILE A 11 -6.368 -11.895 7.077 1.00 0.00 A2 C

ATOM 170 HB ILE A 11 -5.630 -12.508 6.500 0.00 0.00 A2 H

ATOM 171 CG2 ILE A 11 -7.166 -12.718 8.164 1.00 0.00 A2 C

ATOM 172 1HG2 ILE A 11 -7.646 -13.608 7.706 0.00 0.00 A2 H

ATOM 173 2HG2 ILE A 11 -6.487 -13.077 8.966 0.00 0.00 A2 H

ATOM 174 3HG2 ILE A 11 -7.960 -12.097 8.629 0.00 0.00 A2 H

ATOM 175 CG1 ILE A 11 -7.354 -11.327 6.051 1.00 0.00 A2 C

ATOM 176 1HG1 ILE A 11 -8.096 -10.674 6.563 0.00 0.00 A2 H

ATOM 177 2HG1 ILE A 11 -6.796 -10.687 5.330 0.00 0.00 A2 H

ATOM 178 CD ILE A 11 -8.118 -12.430 5.237 1.00 0.00 A2 C

ATOM 179 HD1 ILE A 11 -8.773 -11.960 4.472 0.00 0.00 A2 H

ATOM 180 HD2 ILE A 11 -7.401 -13.101 4.717 0.00 0.00 A2 H

ATOM 181 HD3 ILE A 11 -8.757 -13.047 5.904 0.00 0.00 A2 H

ATOM 182 C ILE A 11 -4.850 -11.290 8.997 1.00 0.00 A2 C

ATOM 183 O ILE A 11 -5.354 -11.050 10.147 1.00 0.00 A2 O

ATOM 184 N MET A 12 -3.765 -12.049 8.815 1.00 0.00 A2 N

ATOM 185 HN MET A 12 -3.351 -12.243 7.928 0.00 0.00 A2 H

ATOM 186 CA MET A 12 -3.084 -12.672 9.949 1.00 0.00 A2 C

ATOM 187 HA MET A 12 -3.854 -13.111 10.572 0.00 0.00 A2 H

ATOM 188 CB MET A 12 -2.096 -13.740 9.437 1.00 0.00 A2 C

ATOM 189 HB1 MET A 12 -2.705 -14.538 8.950 0.00 0.00 A2 H

ATOM 190 HB2 MET A 12 -1.460 -13.301 8.636 0.00 0.00 A2 H

ATOM 191 CG MET A 12 -1.241 -14.365 10.524 1.00 0.00 A2 C

ATOM 192 HG1 MET A 12 -0.394 -13.695 10.782 0.00 0.00 A2 H

ATOM 193 HG2 MET A 12 -1.850 -14.507 11.443 0.00 0.00 A2 H

ATOM 194 SD MET A 12 -0.582 -15.948 9.986 1.00 0.00 A2 S

ATOM 195 CE MET A 12 -1.537 -17.150 10.930 1.00 0.00 A2 C

ATOM 196 HE1 MET A 12 -1.215 -18.187 10.693 0.00 0.00 A2 H

ATOM 197 HE2 MET A 12 -1.407 -16.993 12.023 0.00 0.00 A2 H

ATOM 198 HE3 MET A 12 -2.621 -17.069 10.699 0.00 0.00 A2 H

ATOM 199 C MET A 12 -2.329 -11.645 10.801 1.00 0.00 A2 C

ATOM 200 O MET A 12 -2.320 -11.757 12.009 1.00 0.00 A2 O

ATOM 201 N LYS A 13 -1.698 -10.650 10.163 1.00 0.00 A2 N

ATOM 202 HN LYS A 13 -1.836 -10.448 9.195 0.00 0.00 A2 H

ATOM 203 CA LYS A 13 -0.736 -9.765 10.858 1.00 0.00 A2 C

ATOM 204 HA LYS A 13 -0.505 -10.283 11.781 0.00 0.00 A2 H

ATOM 205 CB LYS A 13 0.558 -9.589 10.012 1.00 0.00 A2 C

ATOM 206 HB1 LYS A 13 0.258 -9.117 9.048 0.00 0.00 A2 H

ATOM 207 HB2 LYS A 13 1.227 -8.860 10.521 0.00 0.00 A2 H

ATOM 208 CG LYS A 13 1.272 -10.953 9.746 1.00 0.00 A2 C

ATOM 209 HG1 LYS A 13 0.526 -11.686 9.363 0.00 0.00 A2 H

ATOM 210 HG2 LYS A 13 2.039 -10.812 8.950 0.00 0.00 A2 H

ATOM 211 CD LYS A 13 1.914 -11.525 10.975 1.00 0.00 A2 C

ATOM 212 HD1 LYS A 13 2.577 -10.748 11.420 0.00 0.00 A2 H

ATOM 213 HD2 LYS A 13 1.111 -11.743 11.717 0.00 0.00 A2 H

ATOM 214 CE LYS A 13 2.695 -12.802 10.590 1.00 0.00 A2 C

ATOM 215 HE1 LYS A 13 2.047 -13.494 10.007 0.00 0.00 A2 H

ATOM 216 HE2 LYS A 13 3.583 -12.545 9.971 0.00 0.00 A2 H

ATOM 217 NZ LYS A 13 3.171 -13.528 11.782 1.00 0.00 A2 N

ATOM 218 HZ1 LYS A 13 3.691 -14.382 11.495 0.00 0.00 A2 H

ATOM 219 HZ2 LYS A 13 3.799 -12.911 12.336 0.00 0.00 A2 H

ATOM 220 HZ3 LYS A 13 2.356 -13.802 12.368 0.00 0.00 A2 H

ATOM 221 C LYS A 13 -1.240 -8.366 11.232 1.00 0.00 A2 C

ATOM 222 O LYS A 13 -0.660 -7.707 12.106 1.00 0.00 A2 O

ATOM 223 N CYS A 14 -2.285 -7.915 10.562 1.00 0.00 A2 N

ATOM 224 HN CYS A 14 -2.903 -8.480 10.020 0.00 0.00 A2 H

ATOM 225 CA CYS A 14 -2.613 -6.487 10.577 1.00 0.00 A2 C

ATOM 226 HA CYS A 14 -2.033 -6.043 11.378 0.00 0.00 A2 H

ATOM 227 CB CYS A 14 -2.427 -5.862 9.164 1.00 0.00 A2 C

ATOM 228 HB1 CYS A 14 -3.259 -6.237 8.525 0.00 0.00 A2 H

ATOM 229 HB2 CYS A 14 -2.556 -4.759 9.220 0.00 0.00 A2 H

ATOM 230 SG CYS A 14 -0.854 -6.251 8.280 1.00 0.00 A2 S

ATOM 231 HG1 CYS A 14 -1.136 -5.606 7.147 0.00 0.00 A2 H

ATOM 232 C CYS A 14 -4.078 -6.276 10.897 1.00 0.00 A2 C

ATOM 233 O CYS A 14 -4.427 -5.224 11.436 1.00 0.00 A2 O

ATOM 234 N SER A 15 -4.938 -7.225 10.469 1.00 0.00 A2 N

ATOM 235 HN SER A 15 -4.678 -8.152 10.200 0.00 0.00 A2 H

ATOM 236 CA SER A 15 -6.361 -6.952 10.365 1.00 0.00 A2 C

ATOM 237 HA SER A 15 -6.427 -6.006 9.844 0.00 0.00 A2 H

ATOM 238 CB SER A 15 -7.108 -8.022 9.502 1.00 0.00 A2 C

ATOM 239 HB1 SER A 15 -8.067 -7.576 9.151 0.00 0.00 A2 H

ATOM 240 HB2 SER A 15 -6.513 -8.273 8.595 0.00 0.00 A2 H

ATOM 241 OG SER A 15 -7.391 -9.187 10.287 1.00 0.00 A2 O

ATOM 242 HG1 SER A 15 -8.034 -9.712 9.794 0.00 0.00 A2 H

ATOM 243 C SER A 15 -7.055 -6.774 11.727 1.00 0.00 A2 C

ATOM 244 O SER A 15 -8.135 -6.180 11.816 1.00 0.00 A2 O

ATOM 245 N GLN A 16 -6.410 -7.272 12.782 1.00 0.00 A2 N

ATOM 246 HN GLN A 16 -5.569 -7.803 12.693 0.00 0.00 A2 H

ATOM 247 CA GLN A 16 -6.875 -7.087 14.178 1.00 0.00 A2 C

ATOM 248 HA GLN A 16 -7.904 -7.424 14.206 0.00 0.00 A2 H

ATOM 249 CB GLN A 16 -6.016 -7.945 15.140 1.00 0.00 A2 C

ATOM 250 HB1 GLN A 16 -4.954 -7.648 14.976 0.00 0.00 A2 H

ATOM 251 HB2 GLN A 16 -6.256 -7.671 16.193 0.00 0.00 A2 H

ATOM 252 CG GLN A 16 -6.103 -9.538 14.989 1.00 0.00 A2 C

ATOM 253 HG1 GLN A 16 -7.082 -9.910 15.361 0.00 0.00 A2 H

ATOM 254 HG2 GLN A 16 -6.007 -9.838 13.925 0.00 0.00 A2 H

ATOM 255 CD GLN A 16 -5.010 -10.385 15.811 1.00 0.00 A2 C

ATOM 256 OE1 GLN A 16 -4.640 -10.033 16.947 1.00 0.00 A2 O

ATOM 257 NE2 GLN A 16 -4.527 -11.521 15.212 1.00 0.00 A2 N

ATOM 258 1HE2 GLN A 16 -3.854 -12.048 15.723 0.00 0.00 A2 H

ATOM 259 2HE2 GLN A 16 -4.841 -11.772 14.303 0.00 0.00 A2 H

ATOM 260 C GLN A 16 -6.858 -5.572 14.619 1.00 0.00 A2 C

ATOM 261 O GLN A 16 -7.609 -5.147 15.525 1.00 0.00 A2 O

ATOM 262 N CYS A 17 -5.976 -4.810 14.009 1.00 0.00 A2 N

ATOM 263 HN CYS A 17 -5.402 -5.173 13.277 0.00 0.00 A2 H

ATOM 264 CA CYS A 17 -5.748 -3.400 14.324 1.00 0.00 A2 C

ATOM 265 HA CYS A 17 -6.415 -3.184 15.151 0.00 0.00 A2 H

ATOM 266 CB CYS A 17 -4.288 -3.193 14.652 1.00 0.00 A2 C

ATOM 267 HB1 CYS A 17 -3.698 -3.593 13.797 0.00 0.00 A2 H

ATOM 268 HB2 CYS A 17 -4.062 -2.105 14.698 0.00 0.00 A2 H

ATOM 269 SG CYS A 17 -3.827 -4.055 16.158 1.00 0.00 A2 S

ATOM 270 HG1 CYS A 17 -2.529 -3.749 16.134 0.00 0.00 A2 H

ATOM 271 C CYS A 17 -6.090 -2.442 13.191 1.00 0.00 A2 C

ATOM 272 O CYS A 17 -6.255 -1.255 13.442 1.00 0.00 A2 O

ATOM 273 N HSD A 18 -6.275 -2.961 11.978 1.00 0.00 A2 N

ATOM 274 HN HSD A 18 -6.310 -3.935 11.759 0.00 0.00 A2 H

ATOM 275 CA HSD A 18 -6.447 -2.060 10.838 1.00 0.00 A2 C

ATOM 276 HA HSD A 18 -6.715 -1.087 11.231 0.00 0.00 A2 H

ATOM 277 CB HSD A 18 -5.170 -1.988 9.997 1.00 0.00 A2 C

ATOM 278 HB1 HSD A 18 -4.799 -3.020 9.814 0.00 0.00 A2 H

ATOM 279 HB2 HSD A 18 -5.404 -1.550 9.002 0.00 0.00 A2 H

ATOM 280 ND1 HSD A 18 -4.015 0.177 10.630 1.00 0.00 A2 N

ATOM 281 HD1 HSD A 18 -4.756 0.788 10.350 0.00 0.00 A2 H

ATOM 282 CG HSD A 18 -4.038 -1.201 10.598 1.00 0.00 A2 C

ATOM 283 CE1 HSD A 18 -2.892 0.584 11.202 1.00 0.00 A2 C

ATOM 284 HE1 HSD A 18 -2.652 1.633 11.397 0.00 0.00 A2 H

ATOM 285 NE2 HSD A 18 -2.162 -0.476 11.483 1.00 0.00 A2 N

ATOM 286 CD2 HSD A 18 -2.866 -1.609 11.139 1.00 0.00 A2 C

ATOM 287 HD2 HSD A 18 -2.478 -2.612 11.272 0.00 0.00 A2 H

ATOM 288 C HSD A 18 -7.595 -2.518 9.922 1.00 0.00 A2 C

ATOM 289 O HSD A 18 -7.809 -3.710 9.766 1.00 0.00 A2 O

ATOM 290 N THR A 19 -8.318 -1.560 9.312 1.00 0.00 A2 N

ATOM 291 HN THR A 19 -8.300 -0.604 9.603 0.00 0.00 A2 H

ATOM 292 CA THR A 19 -9.186 -1.822 8.182 1.00 0.00 A2 C

ATOM 293 HA THR A 19 -9.149 -2.884 7.980 0.00 0.00 A2 H

ATOM 294 CB THR A 19 -10.642 -1.323 8.401 1.00 0.00 A2 C

ATOM 295 HB THR A 19 -11.206 -1.598 7.477 0.00 0.00 A2 H

ATOM 296 OG1 THR A 19 -10.629 0.104 8.513 1.00 0.00 A2 O

ATOM 297 HG1 THR A 19 -11.547 0.358 8.658 0.00 0.00 A2 H

ATOM 298 CG2 THR A 19 -11.276 -1.959 9.721 1.00 0.00 A2 C

ATOM 299 1HG2 THR A 19 -12.350 -1.691 9.812 0.00 0.00 A2 H

ATOM 300 2HG2 THR A 19 -11.202 -3.067 9.691 0.00 0.00 A2 H

ATOM 301 3HG2 THR A 19 -10.749 -1.600 10.631 0.00 0.00 A2 H

ATOM 302 C THR A 19 -8.575 -1.137 6.987 1.00 0.00 A2 C

ATOM 303 O THR A 19 -7.753 -0.256 7.140 1.00 0.00 A2 O

ATOM 304 N VAL A 20 -8.921 -1.602 5.795 1.00 0.00 A2 N

ATOM 305 HN VAL A 20 -9.558 -2.353 5.639 0.00 0.00 A2 H

ATOM 306 CA VAL A 20 -8.356 -1.007 4.554 1.00 0.00 A2 C

ATOM 307 HA VAL A 20 -7.717 -0.190 4.864 0.00 0.00 A2 H

ATOM 308 CB VAL A 20 -7.584 -2.007 3.745 1.00 0.00 A2 C

ATOM 309 HB VAL A 20 -7.355 -1.537 2.757 0.00 0.00 A2 H

ATOM 310 CG1 VAL A 20 -6.253 -2.367 4.468 1.00 0.00 A2 C

ATOM 311 1HG1 VAL A 20 -5.697 -3.137 3.891 0.00 0.00 A2 H

ATOM 312 2HG1 VAL A 20 -5.588 -1.484 4.567 0.00 0.00 A2 H

ATOM 313 3HG1 VAL A 20 -6.455 -2.776 5.481 0.00 0.00 A2 H

ATOM 314 CG2 VAL A 20 -8.475 -3.266 3.394 1.00 0.00 A2 C

ATOM 315 1HG2 VAL A 20 -7.885 -4.019 2.829 0.00 0.00 A2 H

ATOM 316 2HG2 VAL A 20 -8.857 -3.744 4.322 0.00 0.00 A2 H

ATOM 317 3HG2 VAL A 20 -9.344 -2.981 2.765 0.00 0.00 A2 H

ATOM 318 C VAL A 20 -9.431 -0.426 3.638 1.00 0.00 A2 C

ATOM 319 O VAL A 20 -9.116 0.363 2.730 1.00 0.00 A2 O

ATOM 320 N GLU A 21 -10.681 -0.778 3.900 1.00 0.00 A2 N

ATOM 321 HN GLU A 21 -10.970 -1.308 4.692 0.00 0.00 A2 H

ATOM 322 CA GLU A 21 -11.784 -0.395 3.000 1.00 0.00 A2 C

ATOM 323 HA GLU A 21 -11.454 -0.562 1.982 0.00 0.00 A2 H

ATOM 324 CB GLU A 21 -13.006 -1.303 3.205 1.00 0.00 A2 C

ATOM 325 HB1 GLU A 21 -13.805 -0.975 2.500 0.00 0.00 A2 H

ATOM 326 HB2 GLU A 21 -12.753 -2.347 2.914 0.00 0.00 A2 H

ATOM 327 CG GLU A 21 -13.653 -1.339 4.674 1.00 0.00 A2 C

ATOM 328 HG1 GLU A 21 -13.521 -0.355 5.160 0.00 0.00 A2 H

ATOM 329 HG2 GLU A 21 -14.733 -1.580 4.626 0.00 0.00 A2 H

ATOM 330 CD GLU A 21 -13.029 -2.339 5.694 1.00 0.00 A2 C

ATOM 331 OE1 GLU A 21 -11.823 -2.699 5.545 1.00 0.00 A2 O

ATOM 332 OE2 GLU A 21 -13.763 -2.763 6.689 1.00 0.00 A2 O

ATOM 333 C GLU A 21 -12.138 1.096 3.126 1.00 0.00 A2 C

ATOM 334 O GLU A 21 -11.950 1.703 4.204 1.00 0.00 A2 O

ATOM 335 N LYS A 22 -12.647 1.681 2.034 1.00 0.00 A2 N

ATOM 336 HN LYS A 22 -12.769 1.215 1.159 0.00 0.00 A2 H

ATOM 337 CA LYS A 22 -13.073 3.095 2.056 1.00 0.00 A2 C

ATOM 338 HA LYS A 22 -12.243 3.708 2.385 0.00 0.00 A2 H

ATOM 339 CB LYS A 22 -13.577 3.608 0.694 1.00 0.00 A2 C

ATOM 340 HB1 LYS A 22 -12.712 3.577 -0.008 0.00 0.00 A2 H

ATOM 341 HB2 LYS A 22 -14.330 2.893 0.294 0.00 0.00 A2 H

ATOM 342 CG LYS A 22 -14.143 5.024 0.719 1.00 0.00 A2 C

ATOM 343 HG1 LYS A 22 -15.244 4.974 0.880 0.00 0.00 A2 H

ATOM 344 HG2 LYS A 22 -13.705 5.574 1.584 0.00 0.00 A2 H

ATOM 345 CD LYS A 22 -13.865 5.822 -0.626 1.00 0.00 A2 C

ATOM 346 HD1 LYS A 22 -13.333 6.767 -0.371 0.00 0.00 A2 H

ATOM 347 HD2 LYS A 22 -13.177 5.212 -1.256 0.00 0.00 A2 H

ATOM 348 CE LYS A 22 -15.094 6.166 -1.458 1.00 0.00 A2 C

ATOM 349 HE1 LYS A 22 -15.330 5.340 -2.165 0.00 0.00 A2 H

ATOM 350 HE2 LYS A 22 -15.977 6.330 -0.802 0.00 0.00 A2 H

ATOM 351 NZ LYS A 22 -14.936 7.441 -2.310 1.00 0.00 A2 N

ATOM 352 HZ1 LYS A 22 -15.806 7.617 -2.852 0.00 0.00 A2 H

ATOM 353 HZ2 LYS A 22 -14.746 8.253 -1.688 0.00 0.00 A2 H

ATOM 354 HZ3 LYS A 22 -14.139 7.321 -2.968 0.00 0.00 A2 H

ATOM 355 C LYS A 22 -14.169 3.214 3.105 1.00 0.00 A2 C

ATOM 356 O LYS A 22 -15.108 2.416 3.103 1.00 0.00 A2 O

ATOM 357 N GLY A 23 -14.020 4.179 4.009 1.00 0.00 A2 N

ATOM 358 HN GLY A 23 -13.246 4.811 4.004 0.00 0.00 A2 H

ATOM 359 CA GLY A 23 -14.997 4.380 5.086 1.00 0.00 A2 C

ATOM 360 HA1 GLY A 23 -15.977 4.257 4.645 0.00 0.00 A2 H

ATOM 361 HA2 GLY A 23 -14.816 5.364 5.498 0.00 0.00 A2 H

ATOM 362 C GLY A 23 -14.856 3.363 6.217 1.00 0.00 A2 C

ATOM 363 O GLY A 23 -15.589 3.421 7.177 1.00 0.00 A2 O

ATOM 364 N GLY A 24 -13.868 2.468 6.163 1.00 0.00 A2 N

ATOM 365 HN GLY A 24 -13.207 2.409 5.416 0.00 0.00 A2 H

ATOM 366 CA GLY A 24 -13.695 1.480 7.249 1.00 0.00 A2 C

ATOM 367 HA1 GLY A 24 -12.867 0.841 6.973 0.00 0.00 A2 H

ATOM 368 HA2 GLY A 24 -14.636 0.960 7.369 0.00 0.00 A2 H

ATOM 369 C GLY A 24 -13.351 2.152 8.588 1.00 0.00 A2 C

ATOM 370 O GLY A 24 -12.754 3.237 8.619 1.00 0.00 A2 O

ATOM 371 N LYS A 25 -13.719 1.488 9.674 1.00 0.00 A2 N

ATOM 372 HN LYS A 25 -14.187 0.608 9.613 0.00 0.00 A2 H

ATOM 373 CA LYS A 25 -13.482 1.967 11.021 1.00 0.00 A2 C

ATOM 374 HA LYS A 25 -13.962 2.937 11.062 0.00 0.00 A2 H

ATOM 375 CB LYS A 25 -14.049 0.967 12.039 1.00 0.00 A2 C

ATOM 376 HB1 LYS A 25 -13.554 -0.013 11.847 0.00 0.00 A2 H

ATOM 377 HB2 LYS A 25 -13.741 1.278 13.062 0.00 0.00 A2 H

ATOM 378 CG LYS A 25 -15.540 0.827 11.918 1.00 0.00 A2 C

ATOM 379 HG1 LYS A 25 -15.994 1.839 11.816 0.00 0.00 A2 H

ATOM 380 HG2 LYS A 25 -15.777 0.262 10.987 0.00 0.00 A2 H

ATOM 381 CD LYS A 25 -16.069 0.113 13.185 1.00 0.00 A2 C

ATOM 382 HD1 LYS A 25 -15.702 -0.939 13.175 0.00 0.00 A2 H

ATOM 383 HD2 LYS A 25 -15.624 0.613 14.076 0.00 0.00 A2 H

ATOM 384 CE LYS A 25 -17.595 0.180 13.214 1.00 0.00 A2 C

ATOM 385 HE1 LYS A 25 -17.950 1.160 12.825 0.00 0.00 A2 H

ATOM 386 HE2 LYS A 25 -18.034 -0.627 12.587 0.00 0.00 A2 H

ATOM 387 NZ LYS A 25 -18.096 0.014 14.641 1.00 0.00 A2 N

ATOM 388 HZ1 LYS A 25 -19.135 0.061 14.661 0.00 0.00 A2 H

ATOM 389 HZ2 LYS A 25 -17.784 -0.906 15.014 0.00 0.00 A2 H

ATOM 390 HZ3 LYS A 25 -17.705 0.773 15.235 0.00 0.00 A2 H

ATOM 391 C LYS A 25 -12.023 2.165 11.407 1.00 0.00 A2 C

ATOM 392 O LYS A 25 -11.149 1.346 11.086 1.00 0.00 A2 O

ATOM 393 N HSD A 26 -11.785 3.217 12.175 1.00 0.00 A2 N

ATOM 394 HN HSD A 26 -12.377 4.012 12.298 0.00 0.00 A2 H

ATOM 395 CA HSD A 26 -10.534 3.248 12.934 1.00 0.00 A2 C

ATOM 396 HA HSD A 26 -9.720 2.892 12.315 0.00 0.00 A2 H

ATOM 397 CB HSD A 26 -10.295 4.622 13.517 1.00 0.00 A2 C

ATOM 398 HB1 HSD A 26 -11.169 4.907 14.142 0.00 0.00 A2 H

ATOM 399 HB2 HSD A 26 -9.414 4.590 14.194 0.00 0.00 A2 H

ATOM 400 ND1 HSD A 26 -9.249 5.488 11.389 1.00 0.00 A2 N

ATOM 401 HD1 HSD A 26 -8.764 4.647 11.149 0.00 0.00 A2 H

ATOM 402 CG HSD A 26 -10.085 5.667 12.471 1.00 0.00 A2 C

ATOM 403 CE1 HSD A 26 -9.256 6.564 10.625 1.00 0.00 A2 C

ATOM 404 HE1 HSD A 26 -8.699 6.650 9.688 0.00 0.00 A2 H

ATOM 405 NE2 HSD A 26 -10.043 7.454 11.205 1.00 0.00 A2 N

ATOM 406 CD2 HSD A 26 -10.606 6.902 12.336 1.00 0.00 A2 C

ATOM 407 HD2 HSD A 26 -11.304 7.431 12.974 0.00 0.00 A2 H

ATOM 408 C HSD A 26 -10.703 2.257 14.077 1.00 0.00 A2 C

ATOM 409 O HSD A 26 -11.813 2.086 14.595 1.00 0.00 A2 O

ATOM 410 N LYS A 27 -9.641 1.507 14.358 1.00 0.00 A2 N

ATOM 411 HN LYS A 27 -8.785 1.538 13.844 0.00 0.00 A2 H

ATOM 412 CA LYS A 27 -9.661 0.557 15.476 1.00 0.00 A2 C

ATOM 413 HA LYS A 27 -10.542 0.736 16.081 0.00 0.00 A2 H

ATOM 414 CB LYS A 27 -9.655 -0.886 14.979 1.00 0.00 A2 C

ATOM 415 HB1 LYS A 27 -8.660 -1.067 14.510 0.00 0.00 A2 H

ATOM 416 HB2 LYS A 27 -9.717 -1.571 15.854 0.00 0.00 A2 H

ATOM 417 CG LYS A 27 -10.717 -1.167 14.004 1.00 0.00 A2 C

ATOM 418 HG1 LYS A 27 -11.641 -0.621 14.304 0.00 0.00 A2 H

ATOM 419 HG2 LYS A 27 -10.408 -0.775 13.008 0.00 0.00 A2 H

ATOM 420 CD LYS A 27 -10.992 -2.630 13.944 1.00 0.00 A2 C

ATOM 421 HD1 LYS A 27 -10.880 -3.051 14.969 0.00 0.00 A2 H

ATOM 422 HD2 LYS A 27 -12.055 -2.771 13.640 0.00 0.00 A2 H

ATOM 423 CE LYS A 27 -10.088 -3.317 12.992 1.00 0.00 A2 C

ATOM 424 HE1 LYS A 27 -10.323 -3.018 11.946 0.00 0.00 A2 H

ATOM 425 HE2 LYS A 27 -9.027 -3.057 13.203 0.00 0.00 A2 H

ATOM 426 NZ LYS A 27 -10.311 -4.725 13.177 1.00 0.00 A2 N

ATOM 427 HZ1 LYS A 27 -9.703 -5.270 12.532 0.00 0.00 A2 H

ATOM 428 HZ2 LYS A 27 -10.092 -4.985 14.160 0.00 0.00 A2 H

ATOM 429 HZ3 LYS A 27 -11.308 -4.946 12.979 0.00 0.00 A2 H

ATOM 430 C LYS A 27 -8.454 0.877 16.330 1.00 0.00 A2 C

ATOM 431 O LYS A 27 -8.199 2.032 16.554 1.00 0.00 A2 O

ATOM 432 N THR A 28 -7.714 -0.117 16.799 1.00 0.00 A2 N

ATOM 433 HN THR A 28 -7.911 -1.094 16.720 0.00 0.00 A2 H

ATOM 434 CA THR A 28 -6.481 0.188 17.508 1.00 0.00 A2 C

ATOM 435 HA THR A 28 -6.762 0.764 18.379 0.00 0.00 A2 H

ATOM 436 CB THR A 28 -5.730 -1.097 17.929 1.00 0.00 A2 C

ATOM 437 HB THR A 28 -5.604 -1.702 16.998 0.00 0.00 A2 H

ATOM 438 OG1 THR A 28 -6.578 -1.826 18.845 1.00 0.00 A2 O

ATOM 439 HG1 THR A 28 -6.075 -2.609 19.093 0.00 0.00 A2 H

ATOM 440 CG2 THR A 28 -4.450 -0.732 18.739 1.00 0.00 A2 C

ATOM 441 1HG2 THR A 28 -3.843 -1.638 18.949 0.00 0.00 A2 H

ATOM 442 2HG2 THR A 28 -3.817 -0.023 18.164 0.00 0.00 A2 H

ATOM 443 3HG2 THR A 28 -4.712 -0.257 19.709 0.00 0.00 A2 H

ATOM 444 C THR A 28 -5.585 1.118 16.631 1.00 0.00 A2 C

ATOM 445 O THR A 28 -5.007 2.069 17.118 1.00 0.00 A2 O

ATOM 446 N GLY A 29 -5.474 0.799 15.365 1.00 0.00 A2 N

ATOM 447 HN GLY A 29 -5.872 -0.043 15.002 0.00 0.00 A2 H

ATOM 448 CA GLY A 29 -4.764 1.642 14.386 1.00 0.00 A2 C

ATOM 449 HA1 GLY A 29 -4.098 0.999 13.827 0.00 0.00 A2 H

ATOM 450 HA2 GLY A 29 -4.266 2.432 14.932 0.00 0.00 A2 H

ATOM 451 C GLY A 29 -5.722 2.283 13.411 1.00 0.00 A2 C

ATOM 452 O GLY A 29 -6.915 1.914 13.356 1.00 0.00 A2 O

ATOM 453 N PRO A 30 -5.178 3.153 12.538 1.00 0.00 A2 N

ATOM 454 CD PRO A 30 -3.804 3.683 12.636 1.00 0.00 A2 C

ATOM 455 HD1 PRO A 30 -3.490 4.311 13.501 0.00 0.00 A2 H

ATOM 456 HD2 PRO A 30 -3.194 2.752 12.588 0.00 0.00 A2 H

ATOM 457 CA PRO A 30 -6.001 3.910 11.641 1.00 0.00 A2 C

ATOM 458 HA PRO A 30 -6.852 4.319 12.173 0.00 0.00 A2 H

ATOM 459 CB PRO A 30 -5.068 5.076 11.150 1.00 0.00 A2 C

ATOM 460 HB1 PRO A 30 -5.107 5.908 11.889 0.00 0.00 A2 H

ATOM 461 HB2 PRO A 30 -5.358 5.466 10.153 0.00 0.00 A2 H

ATOM 462 CG PRO A 30 -3.651 4.498 11.293 1.00 0.00 A2 C

ATOM 463 HG1 PRO A 30 -2.873 5.281 11.387 0.00 0.00 A2 H

ATOM 464 HG2 PRO A 30 -3.426 3.856 10.411 0.00 0.00 A2 H

ATOM 465 C PRO A 30 -6.507 3.069 10.452 1.00 0.00 A2 C

ATOM 466 O PRO A 30 -5.840 2.127 9.998 1.00 0.00 A2 O

ATOM 467 N ASN A 31 -7.690 3.411 9.951 1.00 0.00 A2 N

ATOM 468 HN ASN A 31 -8.382 3.975 10.400 0.00 0.00 A2 H

ATOM 469 CA ASN A 31 -8.061 2.944 8.625 1.00 0.00 A2 C

ATOM 470 HA ASN A 31 -8.082 1.860 8.662 0.00 0.00 A2 H

ATOM 471 CB ASN A 31 -9.392 3.555 8.210 1.00 0.00 A2 C

ATOM 472 HB1 ASN A 31 -10.183 3.122 8.861 0.00 0.00 A2 H

ATOM 473 HB2 ASN A 31 -9.383 4.651 8.381 0.00 0.00 A2 H

ATOM 474 CG ASN A 31 -9.737 3.257 6.809 1.00 0.00 A2 C

ATOM 475 OD1 ASN A 31 -9.617 4.113 5.943 1.00 0.00 A2 O

ATOM 476 ND2 ASN A 31 -10.196 2.025 6.557 1.00 0.00 A2 N

ATOM 477 1HD2 ASN A 31 -10.423 1.805 5.612 0.00 0.00 A2 H

ATOM 478 2HD2 ASN A 31 -10.271 1.361 7.294 0.00 0.00 A2 H

ATOM 479 C ASN A 31 -6.958 3.353 7.645 1.00 0.00 A2 C

ATOM 480 O ASN A 31 -6.448 4.471 7.714 1.00 0.00 A2 O

ATOM 481 N LEU A 32 -6.641 2.471 6.699 1.00 0.00 A2 N

ATOM 482 HN LEU A 32 -7.136 1.620 6.536 0.00 0.00 A2 H

ATOM 483 CA LEU A 32 -5.505 2.690 5.796 1.00 0.00 A2 C

ATOM 484 HA LEU A 32 -4.937 3.499 6.237 0.00 0.00 A2 H

ATOM 485 CB LEU A 32 -4.652 1.409 5.699 1.00 0.00 A2 C

ATOM 486 HB1 LEU A 32 -5.321 0.595 5.336 0.00 0.00 A2 H

ATOM 487 HB2 LEU A 32 -3.880 1.547 4.910 0.00 0.00 A2 H

ATOM 488 CG LEU A 32 -4.003 0.970 7.031 1.00 0.00 A2 C

ATOM 489 HG LEU A 32 -4.710 0.888 7.734 0.00 0.00 A2 H

ATOM 490 CD1 LEU A 32 -3.286 -0.419 6.820 1.00 0.00 A2 C

ATOM 491 1HD1 LEU A 32 -2.769 -0.736 7.751 0.00 0.00 A2 H

ATOM 492 2HD1 LEU A 32 -4.024 -1.203 6.546 0.00 0.00 A2 H

ATOM 493 3HD1 LEU A 32 -2.530 -0.351 6.009 0.00 0.00 A2 H

ATOM 494 CD2 LEU A 32 -2.963 2.015 7.477 1.00 0.00 A2 C

ATOM 495 1HD2 LEU A 32 -2.505 1.715 8.445 0.00 0.00 A2 H

ATOM 496 2HD2 LEU A 32 -2.145 2.121 6.736 0.00 0.00 A2 H

ATOM 497 3HD2 LEU A 32 -3.443 3.008 7.613 0.00 0.00 A2 H

ATOM 498 C LEU A 32 -5.907 3.122 4.397 1.00 0.00 A2 C

ATOM 499 O LEU A 32 -5.049 3.189 3.524 1.00 0.00 A2 O

ATOM 500 N HSD A 33 -7.205 3.289 4.134 1.00 0.00 A2 N

ATOM 501 HN HSD A 33 -7.945 3.211 4.800 0.00 0.00 A2 H

ATOM 502 CA HSD A 33 -7.635 3.622 2.737 1.00 0.00 A2 C

ATOM 503 HA HSD A 33 -7.285 2.834 2.082 0.00 0.00 A2 H

ATOM 504 CB HSD A 33 -9.169 3.735 2.570 1.00 0.00 A2 C

ATOM 505 HB1 HSD A 33 -9.650 2.843 3.027 0.00 0.00 A2 H

ATOM 506 HB2 HSD A 33 -9.540 4.621 3.131 0.00 0.00 A2 H

ATOM 507 ND1 HSD A 33 -9.924 2.729 0.381 1.00 0.00 A2 N

ATOM 508 HD1 HSD A 33 -10.017 1.790 0.714 0.00 0.00 A2 H

ATOM 509 CG HSD A 33 -9.592 3.835 1.138 1.00 0.00 A2 C

ATOM 510 CE1 HSD A 33 -10.218 3.114 -0.850 1.00 0.00 A2 C

ATOM 511 HE1 HSD A 33 -10.531 2.431 -1.644 0.00 0.00 A2 H

ATOM 512 NE2 HSD A 33 -10.072 4.428 -0.918 1.00 0.00 A2 N

ATOM 513 CD2 HSD A 33 -9.661 4.897 0.302 1.00 0.00 A2 C

ATOM 514 HD2 HSD A 33 -9.418 5.937 0.484 0.00 0.00 A2 H

ATOM 515 C HSD A 33 -6.985 4.911 2.281 1.00 0.00 A2 C

ATOM 516 O HSD A 33 -6.882 5.888 3.051 1.00 0.00 A2 O

ATOM 517 N GLY A 34 -6.544 4.921 1.038 1.00 0.00 A2 N

ATOM 518 HN GLY A 34 -6.598 4.131 0.428 0.00 0.00 A2 H

ATOM 519 CA GLY A 34 -5.935 6.121 0.466 1.00 0.00 A2 C

ATOM 520 HA1 GLY A 34 -6.509 6.947 0.863 0.00 0.00 A2 H

ATOM 521 HA2 GLY A 34 -6.013 6.000 -0.606 0.00 0.00 A2 H

ATOM 522 C GLY A 34 -4.471 6.359 0.806 1.00 0.00 A2 C

ATOM 523 O GLY A 34 -3.886 7.338 0.291 1.00 0.00 A2 O

ATOM 524 N LEU A 35 -3.834 5.478 1.583 1.00 0.00 A2 N

ATOM 525 HN LEU A 35 -4.162 4.582 1.875 0.00 0.00 A2 H

ATOM 526 CA LEU A 35 -2.470 5.833 2.098 1.00 0.00 A2 C

ATOM 527 HA LEU A 35 -2.632 6.766 2.623 0.00 0.00 A2 H

ATOM 528 CB LEU A 35 -1.936 4.869 3.138 1.00 0.00 A2 C

ATOM 529 HB1 LEU A 35 -1.270 5.452 3.815 0.00 0.00 A2 H

ATOM 530 HB2 LEU A 35 -2.780 4.513 3.769 0.00 0.00 A2 H

ATOM 531 CG LEU A 35 -1.124 3.601 2.941 1.00 0.00 A2 C

ATOM 532 HG LEU A 35 -0.461 3.759 2.209 0.00 0.00 A2 H

ATOM 533 CD1 LEU A 35 -0.373 3.241 4.230 1.00 0.00 A2 C

ATOM 534 1HD1 LEU A 35 0.184 2.288 4.104 0.00 0.00 A2 H

ATOM 535 2HD1 LEU A 35 0.355 4.038 4.494 0.00 0.00 A2 H

ATOM 536 3HD1 LEU A 35 -1.082 3.119 5.076 0.00 0.00 A2 H

ATOM 537 CD2 LEU A 35 -2.020 2.483 2.569 1.00 0.00 A2 C

ATOM 538 1HD2 LEU A 35 -1.431 1.555 2.405 0.00 0.00 A2 H

ATOM 539 2HD2 LEU A 35 -2.764 2.273 3.363 0.00 0.00 A2 H

ATOM 540 3HD2 LEU A 35 -2.567 2.718 1.630 0.00 0.00 A2 H

ATOM 541 C LEU A 35 -1.358 6.065 1.077 1.00 0.00 A2 C

ATOM 542 O LEU A 35 -0.457 6.850 1.350 1.00 0.00 A2 O

ATOM 543 N PHE A 36 -1.393 5.345 -0.049 1.00 0.00 A2 N

ATOM 544 HN PHE A 36 -2.144 4.745 -0.320 0.00 0.00 A2 H

ATOM 545 CA PHE A 36 -0.283 5.392 -0.998 1.00 0.00 A2 C

ATOM 546 HA PHE A 36 0.626 5.246 -0.427 0.00 0.00 A2 H

ATOM 547 CB PHE A 36 -0.410 4.344 -2.089 1.00 0.00 A2 C

ATOM 548 HB1 PHE A 36 -1.479 4.250 -2.381 0.00 0.00 A2 H

ATOM 549 HB2 PHE A 36 0.153 4.644 -2.999 0.00 0.00 A2 H

ATOM 550 CG PHE A 36 0.025 2.991 -1.663 1.00 0.00 A2 C

ATOM 551 CD1 PHE A 36 -0.773 2.242 -0.836 1.00 0.00 A2 C

ATOM 552 HD1 PHE A 36 -1.726 2.636 -0.512 0.00 0.00 A2 H

ATOM 553 CE1 PHE A 36 -0.357 0.968 -0.402 1.00 0.00 A2 C

ATOM 554 HE1 PHE A 36 -0.965 0.409 0.295 0.00 0.00 A2 H

ATOM 555 CZ PHE A 36 0.828 0.435 -0.877 1.00 0.00 A2 C

ATOM 556 HZ PHE A 36 1.108 -0.574 -0.611 0.00 0.00 A2 H

ATOM 557 CD2 PHE A 36 1.230 2.457 -2.093 1.00 0.00 A2 C

ATOM 558 HD2 PHE A 36 1.880 3.037 -2.733 0.00 0.00 A2 H

ATOM 559 CE2 PHE A 36 1.626 1.165 -1.675 1.00 0.00 A2 C

ATOM 560 HE2 PHE A 36 2.576 0.762 -1.998 0.00 0.00 A2 H

ATOM 561 C PHE A 36 -0.200 6.793 -1.627 1.00 0.00 A2 C

ATOM 562 O PHE A 36 -1.184 7.297 -2.255 1.00 0.00 A2 O

ATOM 563 N GLY A 37 0.990 7.379 -1.460 1.00 0.00 A2 N

ATOM 564 HN GLY A 37 1.738 6.906 -0.996 0.00 0.00 A2 H

ATOM 565 CA GLY A 37 1.296 8.745 -1.936 1.00 0.00 A2 C

ATOM 566 HA1 GLY A 37 0.728 8.882 -2.846 0.00 0.00 A2 H

ATOM 567 HA2 GLY A 37 2.368 8.789 -2.070 0.00 0.00 A2 H

ATOM 568 C GLY A 37 0.899 9.847 -0.963 1.00 0.00 A2 C

ATOM 569 O GLY A 37 0.962 11.042 -1.307 1.00 0.00 A2 O

ATOM 570 N ARG A 38 0.472 9.435 0.240 1.00 0.00 A2 N

ATOM 571 HN ARG A 38 0.319 8.464 0.408 0.00 0.00 A2 H

ATOM 572 CA ARG A 38 0.202 10.366 1.383 1.00 0.00 A2 C

ATOM 573 HA ARG A 38 0.101 11.366 0.977 0.00 0.00 A2 H

ATOM 574 CB ARG A 38 -1.084 9.974 2.200 1.00 0.00 A2 C

ATOM 575 HB1 ARG A 38 -0.915 8.936 2.574 0.00 0.00 A2 H

ATOM 576 HB2 ARG A 38 -1.161 10.617 3.105 0.00 0.00 A2 H

ATOM 577 CG ARG A 38 -2.382 9.994 1.406 1.00 0.00 A2 C

ATOM 578 HG1 ARG A 38 -2.324 10.811 0.653 0.00 0.00 A2 H

ATOM 579 HG2 ARG A 38 -2.461 9.051 0.818 0.00 0.00 A2 H

ATOM 580 CD ARG A 38 -3.636 10.116 2.377 1.00 0.00 A2 C

ATOM 581 HD1 ARG A 38 -3.675 9.225 3.045 0.00 0.00 A2 H

ATOM 582 HD2 ARG A 38 -3.577 11.034 3.008 0.00 0.00 A2 H

ATOM 583 NE ARG A 38 -4.939 10.209 1.653 1.00 0.00 A2 N

ATOM 584 HE ARG A 38 -4.936 10.900 0.921 0.00 0.00 A2 H

ATOM 585 CZ ARG A 38 -6.009 9.453 1.942 1.00 0.00 A2 C

ATOM 586 NH1 ARG A 38 -5.955 8.538 2.967 1.00 0.00 A2 N

ATOM 587 1HH1 ARG A 38 -6.733 7.960 3.168 0.00 0.00 A2 H

ATOM 588 2HH1 ARG A 38 -5.058 8.401 3.393 0.00 0.00 A2 H

ATOM 589 NH2 ARG A 38 -7.132 9.621 1.242 1.00 0.00 A2 N

ATOM 590 1HH2 ARG A 38 -7.953 9.132 1.501 0.00 0.00 A2 H

ATOM 591 2HH2 ARG A 38 -7.159 10.389 0.615 0.00 0.00 A2 H

ATOM 592 C ARG A 38 1.365 10.417 2.396 1.00 0.00 A2 C

ATOM 593 O ARG A 38 2.080 9.422 2.612 1.00 0.00 A2 O

ATOM 594 N LYS A 39 1.4

**coordinates of RNase A**

CRYST1 100.000 100.000 100.000 90.00 90.00 90.00 P1

ATOM 1 N LYS A 1 0.328 -15.014 -7.279 1.00 0.00 A2 N

ATOM 2 HT1 LYS A 1 -0.223 -15.808 -7.536 0.00 0.00 A2 H

ATOM 3 HT2 LYS A 1 1.222 -15.313 -6.946 0.00 0.00 A2 H

ATOM 4 HT3 LYS A 1 0.443 -14.409 -8.067 0.00 0.00 A2 H

ATOM 5 CA LYS A 1 -0.383 -14.255 -6.173 1.00 0.00 A2 C

ATOM 6 HA LYS A 1 -1.317 -13.854 -6.547 0.00 0.00 A2 H

ATOM 7 CB LYS A 1 -0.660 -15.235 -5.107 1.00 0.00 A2 C

ATOM 8 HB1 LYS A 1 -1.525 -15.851 -5.445 0.00 0.00 A2 H

ATOM 9 HB2 LYS A 1 0.205 -15.931 -5.021 0.00 0.00 A2 H

ATOM 10 CG LYS A 1 -1.010 -15.032 -3.692 1.00 0.00 A2 C

ATOM 11 HG1 LYS A 1 -0.090 -14.767 -3.123 0.00 0.00 A2 H

ATOM 12 HG2 LYS A 1 -1.710 -14.169 -3.612 0.00 0.00 A2 H

ATOM 13 CD LYS A 1 -1.644 -16.287 -3.049 1.00 0.00 A2 C

ATOM 14 HD1 LYS A 1 -2.416 -16.688 -3.746 0.00 0.00 A2 H

ATOM 15 HD2 LYS A 1 -0.851 -17.063 -2.948 0.00 0.00 A2 H

ATOM 16 CE LYS A 1 -2.279 -16.071 -1.690 1.00 0.00 A2 C

ATOM 17 HE1 LYS A 1 -2.348 -17.033 -1.134 0.00 0.00 A2 H

ATOM 18 HE2 LYS A 1 -1.676 -15.358 -1.086 0.00 0.00 A2 H

ATOM 19 NZ LYS A 1 -3.653 -15.521 -1.678 1.00 0.00 A2 N

ATOM 20 HZ1 LYS A 1 -3.986 -15.417 -0.698 0.00 0.00 A2 H

ATOM 21 HZ2 LYS A 1 -3.659 -14.592 -2.146 0.00 0.00 A2 H

ATOM 22 HZ3 LYS A 1 -4.288 -16.166 -2.191 0.00 0.00 A2 H

ATOM 23 C LYS A 1 0.530 -13.063 -5.847 1.00 0.00 A2 C

ATOM 24 O LYS A 1 1.678 -13.036 -6.237 1.00 0.00 A2 O

ATOM 25 N GLU A 2 -0.076 -12.118 -5.175 1.00 0.00 A2 N

ATOM 26 HN GLU A 2 -1.028 -12.099 -4.883 0.00 0.00 A2 H

ATOM 27 CA GLU A 2 0.706 -10.910 -4.776 1.00 0.00 A2 C

ATOM 28 HA GLU A 2 1.060 -10.429 -5.679 0.00 0.00 A2 H

ATOM 29 CB GLU A 2 -0.216 -10.073 -3.942 1.00 0.00 A2 C

ATOM 30 HB1 GLU A 2 -1.179 -9.964 -4.492 0.00 0.00 A2 H

ATOM 31 HB2 GLU A 2 -0.462 -10.609 -2.998 0.00 0.00 A2 H

ATOM 32 CG GLU A 2 0.318 -8.668 -3.608 1.00 0.00 A2 C

ATOM 33 HG1 GLU A 2 1.258 -8.758 -3.033 0.00 0.00 A2 H

ATOM 34 HG2 GLU A 2 0.509 -8.086 -4.531 0.00 0.00 A2 H

ATOM 35 CD GLU A 2 -0.655 -7.939 -2.741 1.00 0.00 A2 C

ATOM 36 OE1 GLU A 2 -1.364 -8.482 -1.915 1.00 0.00 A2 O

ATOM 37 OE2 GLU A 2 -0.724 -6.644 -2.889 1.00 0.00 A2 O

ATOM 38 C GLU A 2 1.935 -11.281 -3.948 1.00 0.00 A2 C

ATOM 39 O GLU A 2 1.840 -12.086 -3.037 1.00 0.00 A2 O

ATOM 40 N THR A 3 3.030 -10.652 -4.314 1.00 0.00 A2 N

ATOM 41 HN THR A 3 3.104 -9.996 -5.065 0.00 0.00 A2 H

ATOM 42 CA THR A 3 4.309 -10.895 -3.584 1.00 0.00 A2 C

ATOM 43 HA THR A 3 4.333 -11.942 -3.313 0.00 0.00 A2 H

ATOM 44 CB THR A 3 5.529 -10.518 -4.383 1.00 0.00 A2 C

ATOM 45 HB THR A 3 6.410 -10.836 -3.773 0.00 0.00 A2 H

ATOM 46 OG1 THR A 3 5.514 -9.083 -4.489 1.00 0.00 A2 O

ATOM 47 HG1 THR A 3 6.291 -8.856 -5.011 0.00 0.00 A2 H

ATOM 48 CG2 THR A 3 5.642 -11.171 -5.769 1.00 0.00 A2 C

ATOM 49 1HG2 THR A 3 6.618 -10.931 -6.241 0.00 0.00 A2 H

ATOM 50 2HG2 THR A 3 5.566 -12.276 -5.684 0.00 0.00 A2 H

ATOM 51 3HG2 THR A 3 4.833 -10.818 -6.445 0.00 0.00 A2 H

ATOM 52 C THR A 3 4.248 -10.080 -2.229 1.00 0.00 A2 C

ATOM 53 O THR A 3 3.500 -9.183 -2.058 1.00 0.00 A2 O

ATOM 54 N ALA A 4 5.107 -10.559 -1.341 1.00 0.00 A2 N

ATOM 55 HN ALA A 4 5.722 -11.334 -1.483 0.00 0.00 A2 H

ATOM 56 CA ALA A 4 5.192 -9.919 -0.016 1.00 0.00 A2 C

ATOM 57 HA ALA A 4 4.223 -9.855 0.466 0.00 0.00 A2 H

ATOM 58 CB ALA A 4 6.058 -10.770 0.933 1.00 0.00 A2 C

ATOM 59 HB1 ALA A 4 5.604 -11.777 1.049 0.00 0.00 A2 H

ATOM 60 HB2 ALA A 4 7.084 -10.903 0.526 0.00 0.00 A2 H

ATOM 61 HB3 ALA A 4 6.134 -10.309 1.941 0.00 0.00 A2 H

ATOM 62 C ALA A 4 5.677 -8.468 -0.213 1.00 0.00 A2 C

ATOM 63 O ALA A 4 5.171 -7.593 0.492 1.00 0.00 A2 O

ATOM 64 N ALA A 5 6.558 -8.213 -1.128 1.00 0.00 A2 N

ATOM 65 HN ALA A 5 6.938 -8.935 -1.706 0.00 0.00 A2 H

ATOM 66 CA ALA A 5 7.059 -6.881 -1.388 1.00 0.00 A2 C

ATOM 67 HA ALA A 5 7.461 -6.437 -0.485 0.00 0.00 A2 H

ATOM 68 CB ALA A 5 8.214 -6.819 -2.367 1.00 0.00 A2 C

ATOM 69 HB1 ALA A 5 9.051 -7.449 -1.997 0.00 0.00 A2 H

ATOM 70 HB2 ALA A 5 7.912 -7.204 -3.366 0.00 0.00 A2 H

ATOM 71 HB3 ALA A 5 8.593 -5.781 -2.488 0.00 0.00 A2 H

ATOM 72 C ALA A 5 5.932 -5.966 -1.853 1.00 0.00 A2 C

ATOM 73 O ALA A 5 5.825 -4.801 -1.432 1.00 0.00 A2 O

ATOM 74 N ALA A 6 5.110 -6.508 -2.769 1.00 0.00 A2 N

ATOM 75 HN ALA A 6 5.218 -7.435 -3.126 0.00 0.00 A2 H

ATOM 76 CA ALA A 6 3.979 -5.753 -3.312 1.00 0.00 A2 C

ATOM 77 HA ALA A 6 4.300 -4.779 -3.665 0.00 0.00 A2 H

ATOM 78 CB ALA A 6 3.354 -6.542 -4.476 1.00 0.00 A2 C

ATOM 79 HB1 ALA A 6 4.108 -6.681 -5.280 0.00 0.00 A2 H

ATOM 80 HB2 ALA A 6 3.021 -7.549 -4.142 0.00 0.00 A2 H

ATOM 81 HB3 ALA A 6 2.483 -6.007 -4.912 0.00 0.00 A2 H

ATOM 82 C ALA A 6 2.946 -5.499 -2.242 1.00 0.00 A2 C

ATOM 83 O ALA A 6 2.336 -4.415 -2.197 1.00 0.00 A2 O

ATOM 84 N LYS A 7 2.681 -6.464 -1.397 1.00 0.00 A2 N

ATOM 85 HN LYS A 7 3.135 -7.350 -1.475 0.00 0.00 A2 H

ATOM 86 CA LYS A 7 1.742 -6.351 -0.307 1.00 0.00 A2 C

ATOM 87 HA LYS A 7 0.782 -6.053 -0.710 0.00 0.00 A2 H

ATOM 88 CB LYS A 7 1.561 -7.653 0.413 1.00 0.00 A2 C

ATOM 89 HB1 LYS A 7 1.177 -8.389 -0.331 0.00 0.00 A2 H

ATOM 90 HB2 LYS A 7 2.557 -8.029 0.738 0.00 0.00 A2 H

ATOM 91 CG LYS A 7 0.583 -7.577 1.605 1.00 0.00 A2 C

ATOM 92 HG1 LYS A 7 0.951 -6.816 2.330 0.00 0.00 A2 H

ATOM 93 HG2 LYS A 7 -0.411 -7.232 1.237 0.00 0.00 A2 H

ATOM 94 CD LYS A 7 0.502 -8.929 2.255 1.00 0.00 A2 C

ATOM 95 HD1 LYS A 7 -0.052 -9.614 1.573 0.00 0.00 A2 H

ATOM 96 HD2 LYS A 7 1.538 -9.327 2.358 0.00 0.00 A2 H

ATOM 97 CE LYS A 7 -0.178 -8.867 3.623 1.00 0.00 A2 C

ATOM 98 HE1 LYS A 7 0.368 -8.171 4.299 0.00 0.00 A2 H

ATOM 99 HE2 LYS A 7 -1.226 -8.509 3.522 0.00 0.00 A2 H

ATOM 100 NZ LYS A 7 -0.160 -10.244 4.187 1.00 0.00 A2 N

ATOM 101 HZ1 LYS A 7 -0.613 -10.251 5.123 0.00 0.00 A2 H

ATOM 102 HZ2 LYS A 7 -0.673 -10.887 3.550 0.00 0.00 A2 H

ATOM 103 HZ3 LYS A 7 0.824 -10.567 4.278 0.00 0.00 A2 H

ATOM 104 C LYS A 7 2.221 -5.218 0.625 1.00 0.00 A2 C

ATOM 105 O LYS A 7 1.436 -4.406 1.111 1.00 0.00 A2 O

ATOM 106 N PHE A 8 3.509 -5.163 0.854 1.00 0.00 A2 N

ATOM 107 HN PHE A 8 4.203 -5.786 0.496 0.00 0.00 A2 H

ATOM 108 CA PHE A 8 4.050 -4.067 1.742 1.00 0.00 A2 C

ATOM 109 HA PHE A 8 3.520 -4.143 2.684 0.00 0.00 A2 H

ATOM 110 CB PHE A 8 5.545 -4.306 1.997 1.00 0.00 A2 C

ATOM 111 HB1 PHE A 8 5.669 -5.240 2.588 0.00 0.00 A2 H

ATOM 112 HB2 PHE A 8 6.096 -4.447 1.042 0.00 0.00 A2 H

ATOM 113 CG PHE A 8 6.187 -3.197 2.801 1.00 0.00 A2 C

ATOM 114 CD1 PHE A 8 6.301 -3.308 4.185 1.00 0.00 A2 C

ATOM 115 HD1 PHE A 8 5.963 -4.209 4.679 0.00 0.00 A2 H

ATOM 116 CE1 PHE A 8 6.834 -2.288 4.964 1.00 0.00 A2 C

ATOM 117 HE1 PHE A 8 6.949 -2.425 6.030 0.00 0.00 A2 H

ATOM 118 CZ PHE A 8 7.207 -1.122 4.325 1.00 0.00 A2 C

ATOM 119 HZ PHE A 8 7.573 -0.311 4.939 0.00 0.00 A2 H

ATOM 120 CD2 PHE A 8 6.624 -2.022 2.195 1.00 0.00 A2 C

ATOM 121 HD2 PHE A 8 6.549 -1.907 1.123 0.00 0.00 A2 H

ATOM 122 CE2 PHE A 8 7.135 -0.970 2.938 1.00 0.00 A2 C

ATOM 123 HE2 PHE A 8 7.461 -0.068 2.440 0.00 0.00 A2 H

ATOM 124 C PHE A 8 3.731 -2.717 1.164 1.00 0.00 A2 C

ATOM 125 O PHE A 8 3.331 -1.755 1.859 1.00 0.00 A2 O

ATOM 126 N GLU A 9 3.963 -2.525 -0.133 1.00 0.00 A2 N

ATOM 127 HN GLU A 9 4.344 -3.222 -0.735 0.00 0.00 A2 H

ATOM 128 CA GLU A 9 3.681 -1.280 -0.787 1.00 0.00 A2 C

ATOM 129 HA GLU A 9 4.258 -0.505 -0.299 0.00 0.00 A2 H

ATOM 130 CB GLU A 9 4.043 -1.183 -2.237 1.00 0.00 A2 C

ATOM 131 HB1 GLU A 9 3.473 -1.965 -2.790 0.00 0.00 A2 H

ATOM 132 HB2 GLU A 9 3.699 -0.207 -2.647 0.00 0.00 A2 H

ATOM 133 CG GLU A 9 5.486 -1.358 -2.532 1.00 0.00 A2 C

ATOM 134 HG1 GLU A 9 6.065 -0.562 -2.029 0.00 0.00 A2 H

ATOM 135 HG2 GLU A 9 5.846 -2.346 -2.183 0.00 0.00 A2 H

ATOM 136 CD GLU A 9 5.925 -1.246 -3.986 1.00 0.00 A2 C

ATOM 137 OE1 GLU A 9 6.977 -1.882 -4.269 1.00 0.00 A2 O

ATOM 138 OE2 GLU A 9 5.149 -0.434 -4.668 1.00 0.00 A2 O

ATOM 139 C GLU A 9 2.208 -0.903 -0.649 1.00 0.00 A2 C

ATOM 140 O GLU A 9 1.839 0.235 -0.359 1.00 0.00 A2 O

ATOM 141 N ARG A 10 1.335 -1.898 -0.930 1.00 0.00 A2 N

ATOM 142 HN ARG A 10 1.629 -2.810 -1.205 0.00 0.00 A2 H

ATOM 143 CA ARG A 10 -0.096 -1.674 -0.841 1.00 0.00 A2 C

ATOM 144 HA ARG A 10 -0.325 -0.824 -1.473 0.00 0.00 A2 H

ATOM 145 CB ARG A 10 -0.874 -2.921 -1.386 1.00 0.00 A2 C

ATOM 146 HB1 ARG A 10 -0.557 -3.051 -2.448 0.00 0.00 A2 H

ATOM 147 HB2 ARG A 10 -0.534 -3.835 -0.851 0.00 0.00 A2 H

ATOM 148 CG ARG A 10 -2.402 -2.789 -1.344 1.00 0.00 A2 C

ATOM 149 HG1 ARG A 10 -2.720 -2.694 -0.283 0.00 0.00 A2 H

ATOM 150 HG2 ARG A 10 -2.696 -1.831 -1.831 0.00 0.00 A2 H

ATOM 151 CD ARG A 10 -3.067 -3.938 -2.067 1.00 0.00 A2 C

ATOM 152 HD1 ARG A 10 -4.173 -3.815 -2.018 0.00 0.00 A2 H

ATOM 153 HD2 ARG A 10 -2.759 -3.981 -3.138 0.00 0.00 A2 H

ATOM 154 NE ARG A 10 -2.731 -5.237 -1.510 1.00 0.00 A2 N

ATOM 155 HE ARG A 10 -1.962 -5.683 -1.982 0.00 0.00 A2 H

ATOM 156 CZ ARG A 10 -3.314 -5.822 -0.514 1.00 0.00 A2 C

ATOM 157 NH1 ARG A 10 -4.292 -5.227 0.213 1.00 0.00 A2 N

ATOM 158 1HH1 ARG A 10 -4.700 -5.693 0.986 0.00 0.00 A2 H

ATOM 159 2HH1 ARG A 10 -4.470 -4.262 0.011 0.00 0.00 A2 H

ATOM 160 NH2 ARG A 10 -2.939 -6.988 -0.076 1.00 0.00 A2 N

ATOM 161 1HH2 ARG A 10 -3.463 -7.442 0.630 0.00 0.00 A2 H

ATOM 162 2HH2 ARG A 10 -2.261 -7.472 -0.614 0.00 0.00 A2 H

ATOM 163 C ARG A 10 -0.581 -1.320 0.558 1.00 0.00 A2 C

ATOM 164 O ARG A 10 -1.426 -0.458 0.741 1.00 0.00 A2 O

ATOM 165 N GLN A 11 -0.094 -2.016 1.532 1.00 0.00 A2 N

ATOM 166 HN GLN A 11 0.602 -2.726 1.438 0.00 0.00 A2 H

ATOM 167 CA GLN A 11 -0.550 -1.799 2.892 1.00 0.00 A2 C

ATOM 168 HA GLN A 11 -1.617 -1.626 2.822 0.00 0.00 A2 H

ATOM 169 CB GLN A 11 -0.257 -3.003 3.783 1.00 0.00 A2 C

ATOM 170 HB1 GLN A 11 0.851 -3.122 3.810 0.00 0.00 A2 H

ATOM 171 HB2 GLN A 11 -0.570 -2.774 4.827 0.00 0.00 A2 H

ATOM 172 CG GLN A 11 -0.838 -4.308 3.388 1.00 0.00 A2 C

ATOM 173 HG1 GLN A 11 -1.922 -4.199 3.169 0.00 0.00 A2 H

ATOM 174 HG2 GLN A 11 -0.344 -4.698 2.474 0.00 0.00 A2 H

ATOM 175 CD GLN A 11 -0.709 -5.346 4.521 1.00 0.00 A2 C

ATOM 176 OE1 GLN A 11 -1.573 -6.138 4.735 1.00 0.00 A2 O

ATOM 177 NE2 GLN A 11 0.434 -5.238 5.172 1.00 0.00 A2 N

ATOM 178 1HE2 GLN A 11 0.590 -5.880 5.917 0.00 0.00 A2 H

ATOM 179 2HE2 GLN A 11 1.082 -4.528 4.919 0.00 0.00 A2 H

ATOM 180 C GLN A 11 0.072 -0.573 3.561 1.00 0.00 A2 C

ATOM 181 O GLN A 11 -0.577 -0.016 4.425 1.00 0.00 A2 O

ATOM 182 N HSD A 12 1.306 -0.206 3.195 1.00 0.00 A2 N

ATOM 183 HN HSD A 12 1.784 -0.564 2.394 0.00 0.00 A2 H

ATOM 184 CA HSD A 12 2.048 0.772 3.973 1.00 0.00 A2 C

ATOM 185 HA HSD A 12 1.359 1.089 4.746 0.00 0.00 A2 H

ATOM 186 CB HSD A 12 3.208 0.104 4.716 1.00 0.00 A2 C

ATOM 187 HB1 HSD A 12 3.949 -0.264 3.974 0.00 0.00 A2 H

ATOM 188 HB2 HSD A 12 3.731 0.858 5.344 0.00 0.00 A2 H

ATOM 189 ND1 HSD A 12 2.072 -0.834 6.735 1.00 0.00 A2 N

ATOM 190 HD1 HSD A 12 1.701 0.046 7.034 0.00 0.00 A2 H

ATOM 191 CG HSD A 12 2.834 -1.059 5.597 1.00 0.00 A2 C

ATOM 192 CE1 HSD A 12 1.857 -2.048 7.250 1.00 0.00 A2 C

ATOM 193 HE1 HSD A 12 1.259 -2.233 8.146 0.00 0.00 A2 H

ATOM 194 NE2 HSD A 12 2.452 -2.994 6.535 1.00 0.00 A2 N

ATOM 195 CD2 HSD A 12 3.068 -2.389 5.474 1.00 0.00 A2 C

ATOM 196 HD2 HSD A 12 3.648 -2.923 4.731 0.00 0.00 A2 H

ATOM 197 C HSD A 12 2.518 1.999 3.264 1.00 0.00 A2 C

ATOM 198 O HSD A 12 2.943 2.925 3.995 1.00 0.00 A2 O

ATOM 199 N MET A 13 2.594 2.035 1.965 1.00 0.00 A2 N

ATOM 200 HN MET A 13 2.289 1.332 1.326 0.00 0.00 A2 H

ATOM 201 CA MET A 13 3.184 3.215 1.330 1.00 0.00 A2 C

ATOM 202 HA MET A 13 3.791 3.686 2.094 0.00 0.00 A2 H

ATOM 203 CB MET A 13 4.077 2.763 0.140 1.00 0.00 A2 C

ATOM 204 HB1 MET A 13 3.453 2.095 -0.499 0.00 0.00 A2 H

ATOM 205 HB2 MET A 13 4.330 3.646 -0.488 0.00 0.00 A2 H

ATOM 206 CG MET A 13 5.340 2.016 0.493 1.00 0.00 A2 C

ATOM 207 HG1 MET A 13 5.094 1.067 1.015 0.00 0.00 A2 H

ATOM 208 HG2 MET A 13 5.892 1.755 -0.436 0.00 0.00 A2 H

ATOM 209 SD MET A 13 6.475 2.928 1.571 1.00 0.00 A2 S

ATOM 210 CE MET A 13 6.692 4.446 0.585 1.00 0.00 A2 C

ATOM 211 HE1 MET A 13 7.386 5.151 1.090 0.00 0.00 A2 H

ATOM 212 HE2 MET A 13 7.112 4.214 -0.418 0.00 0.00 A2 H

ATOM 213 HE3 MET A 13 5.723 4.969 0.437 0.00 0.00 A2 H

ATOM 214 C MET A 13 2.178 4.232 0.858 1.00 0.00 A2 C

ATOM 215 O MET A 13 1.232 3.864 0.176 1.00 0.00 A2 O

ATOM 216 N ASP A 14 2.410 5.511 1.147 1.00 0.00 A2 N

ATOM 217 HN ASP A 14 3.120 5.855 1.755 0.00 0.00 A2 H

ATOM 218 CA ASP A 14 1.612 6.554 0.574 1.00 0.00 A2 C

ATOM 219 HA ASP A 14 1.219 6.208 -0.375 0.00 0.00 A2 H

ATOM 220 CB ASP A 14 0.401 6.981 1.375 1.00 0.00 A2 C

ATOM 221 HB1 ASP A 14 -0.243 6.098 1.562 0.00 0.00 A2 H

ATOM 222 HB2 ASP A 14 0.708 7.402 2.353 0.00 0.00 A2 H

ATOM 223 CG ASP A 14 -0.382 8.024 0.551 1.00 0.00 A2 C

ATOM 224 OD1 ASP A 14 -0.175 8.197 -0.629 1.00 0.00 A2 O

ATOM 225 OD2 ASP A 14 -1.244 8.655 1.278 1.00 0.00 A2 O

ATOM 226 C ASP A 14 2.561 7.750 0.259 1.00 0.00 A2 C

ATOM 227 O ASP A 14 2.575 8.740 0.936 1.00 0.00 A2 O

ATOM 228 N SER A 15 3.242 7.596 -0.881 1.00 0.00 A2 N

ATOM 229 HN SER A 15 3.180 6.794 -1.474 0.00 0.00 A2 H

ATOM 230 CA SER A 15 4.171 8.637 -1.367 1.00 0.00 A2 C

ATOM 231 HA SER A 15 4.690 8.995 -0.488 0.00 0.00 A2 H

ATOM 232 CB SER A 15 5.098 8.009 -2.436 1.00 0.00 A2 C

ATOM 233 HB1 SER A 15 4.470 7.712 -3.307 0.00 0.00 A2 H

ATOM 234 HB2 SER A 15 5.832 8.762 -2.803 0.00 0.00 A2 H

ATOM 235 OG SER A 15 5.736 6.864 -1.903 1.00 0.00 A2 O

ATOM 236 HG1 SER A 15 6.119 6.381 -2.646 0.00 0.00 A2 H

ATOM 237 C SER A 15 3.446 9.818 -1.974 1.00 0.00 A2 C

ATOM 238 O SER A 15 4.144 10.783 -2.333 1.00 0.00 A2 O

ATOM 239 N SER A 16 2.185 9.765 -2.165 1.00 0.00 A2 N

ATOM 240 HN SER A 16 1.677 8.961 -1.857 0.00 0.00 A2 H

ATOM 241 CA SER A 16 1.352 10.793 -2.814 1.00 0.00 A2 C

ATOM 242 HA SER A 16 1.975 11.213 -3.593 0.00 0.00 A2 H

ATOM 243 CB SER A 16 0.064 10.219 -3.459 1.00 0.00 A2 C

ATOM 244 HB1 SER A 16 -0.278 10.933 -4.243 0.00 0.00 A2 H

ATOM 245 HB2 SER A 16 0.283 9.253 -3.969 0.00 0.00 A2 H

ATOM 246 OG SER A 16 -1.004 10.068 -2.440 1.00 0.00 A2 O

ATOM 247 HG1 SER A 16 -1.832 9.927 -2.916 0.00 0.00 A2 H

ATOM 248 C SER A 16 0.978 11.907 -1.897 1.00 0.00 A2 C

ATOM 249 O SER A 16 0.447 12.974 -2.420 1.00 0.00 A2 O

ATOM 250 N THR A 17 1.158 11.775 -0.616 1.00 0.00 A2 N

ATOM 251 HN THR A 17 1.535 10.949 -0.198 0.00 0.00 A2 H

ATOM 252 CA THR A 17 0.825 12.823 0.330 1.00 0.00 A2 C

ATOM 253 HA THR A 17 0.802 13.758 -0.213 0.00 0.00 A2 H

ATOM 254 CB THR A 17 -0.527 12.644 1.066 1.00 0.00 A2 C

ATOM 255 HB THR A 17 -0.657 13.554 1.701 0.00 0.00 A2 H

ATOM 256 OG1 THR A 17 -0.395 11.491 1.918 1.00 0.00 A2 O

ATOM 257 HG1 THR A 17 -1.249 11.395 2.354 0.00 0.00 A2 H

ATOM 258 CG2 THR A 17 -1.647 12.354 0.118 1.00 0.00 A2 C

ATOM 259 1HG2 THR A 17 -2.622 12.342 0.650 0.00 0.00 A2 H

ATOM 260 2HG2 THR A 17 -1.698 13.134 -0.671 0.00 0.00 A2 H

ATOM 261 3HG2 THR A 17 -1.508 11.368 -0.376 0.00 0.00 A2 H

ATOM 262 C THR A 17 1.985 12.924 1.319 1.00 0.00 A2 C

ATOM 263 O THR A 17 2.654 11.947 1.561 1.00 0.00 A2 O

ATOM 264 N SER A 18 2.208 14.116 1.860 1.00 0.00 A2 N

ATOM 265 HN SER A 18 1.660 14.919 1.625 0.00 0.00 A2 H

ATOM 266 CA SER A 18 3.245 14.372 2.824 1.00 0.00 A2 C

ATOM 267 HA SER A 18 4.058 13.721 2.529 0.00 0.00 A2 H

ATOM 268 CB SER A 18 3.739 15.850 2.822 1.00 0.00 A2 C

ATOM 269 HB1 SER A 18 2.845 16.514 2.860 0.00 0.00 A2 H

ATOM 270 HB2 SER A 18 4.341 16.059 3.735 0.00 0.00 A2 H

ATOM 271 OG SER A 18 4.443 15.922 1.568 1.00 0.00 A2 O

ATOM 272 HG1 SER A 18 4.592 16.857 1.380 0.00 0.00 A2 H

ATOM 273 C SER A 18 2.874 14.013 4.264 1.00 0.00 A2 C

ATOM 274 O SER A 18 3.727 13.805 5.108 1.00 0.00 A2 O

ATOM 275 N ALA A 19 1.552 13.938 4.521 1.00 0.00 A2 N

ATOM 276 HN ALA A 19 0.919 14.078 3.760 0.00 0.00 A2 H

ATOM 277 CA ALA A 19 0.940 13.675 5.788 1.00 0.00 A2 C

ATOM 278 HA ALA A 19 1.256 12.728 6.211 0.00 0.00 A2 H

ATOM 279 CB ALA A 19 1.216 14.775 6.812 1.00 0.00 A2 C

ATOM 280 HB1 ALA A 19 2.306 14.824 7.022 0.00 0.00 A2 H

ATOM 281 HB2 ALA A 19 0.897 15.768 6.427 0.00 0.00 A2 H

ATOM 282 HB3 ALA A 19 0.690 14.582 7.772 0.00 0.00 A2 H

ATOM 283 C ALA A 19 -0.607 13.556 5.542 1.00 0.00 A2 C

ATOM 284 O ALA A 19 -1.128 13.963 4.555 1.00 0.00 A2 O

ATOM 285 N ALA A 20 -1.223 12.998 6.549 1.00 0.00 A2 N

ATOM 286 HN ALA A 20 -0.775 12.652 7.373 0.00 0.00 A2 H

ATOM 287 CA ALA A 20 -2.712 12.842 6.514 1.00 0.00 A2 C

ATOM 288 HA ALA A 20 -3.065 12.410 5.585 0.00 0.00 A2 H

ATOM 289 CB ALA A 20 -3.071 12.058 7.752 1.00 0.00 A2 C

ATOM 290 HB1 ALA A 20 -2.680 11.022 7.664 0.00 0.00 A2 H

ATOM 291 HB2 ALA A 20 -2.622 12.517 8.660 0.00 0.00 A2 H

ATOM 292 HB3 ALA A 20 -4.172 11.998 7.894 0.00 0.00 A2 H

ATOM 293 C ALA A 20 -3.319 14.298 6.587 1.00 0.00 A2 C

ATOM 294 O ALA A 20 -2.862 15.006 7.479 1.00 0.00 A2 O

ATOM 295 N SER A 21 -4.257 14.565 5.765 1.00 0.00 A2 N

ATOM 296 HN SER A 21 -4.685 13.947 5.106 0.00 0.00 A2 H

ATOM 297 CA SER A 21 -4.819 15.975 5.752 1.00 0.00 A2 C

ATOM 298 HA SER A 21 -4.059 16.555 6.259 0.00 0.00 A2 H

ATOM 299 CB SER A 21 -5.020 16.315 4.261 1.00 0.00 A2 C

ATOM 300 HB1 SER A 21 -5.598 17.265 4.198 0.00 0.00 A2 H

ATOM 301 HB2 SER A 21 -4.038 16.496 3.769 0.00 0.00 A2 H

ATOM 302 OG SER A 21 -5.755 15.285 3.580 1.00 0.00 A2 O

ATOM 303 HG1 SER A 21 -6.037 15.651 2.732 0.00 0.00 A2 H

ATOM 304 C SER A 21 -6.125 16.196 6.492 1.00 0.00 A2 C

ATOM 305 O SER A 21 -6.610 17.339 6.668 1.00 0.00 A2 O

ATOM 306 N SER A 22 -6.745 15.084 6.934 1.00 0.00 A2 N

ATOM 307 HN SER A 22 -6.409 14.149 6.818 0.00 0.00 A2 H

ATOM 308 CA SER A 22 -8.021 15.180 7.652 1.00 0.00 A2 C

ATOM 309 HA SER A 22 -7.927 16.010 8.340 0.00 0.00 A2 H

ATOM 310 CB SER A 22 -9.205 15.377 6.723 1.00 0.00 A2 C

ATOM 311 HB1 SER A 22 -10.044 15.805 7.318 0.00 0.00 A2 H

ATOM 312 HB2 SER A 22 -8.956 16.117 5.929 0.00 0.00 A2 H

ATOM 313 OG SER A 22 -9.626 14.151 6.163 1.00 0.00 A2 O

ATOM 314 HG1 SER A 22 -10.496 14.301 5.773 0.00 0.00 A2 H

ATOM 315 C SER A 22 -8.243 13.923 8.485 1.00 0.00 A2 C

ATOM 316 O SER A 22 -7.570 12.886 8.332 1.00 0.00 A2 O

ATOM 317 N SER A 23 -9.254 14.047 9.363 1.00 0.00 A2 N

ATOM 318 HN SER A 23 -9.824 14.853 9.522 0.00 0.00 A2 H

ATOM 319 CA SER A 23 -9.597 12.885 10.211 1.00 0.00 A2 C

ATOM 320 HA SER A 23 -8.671 12.582 10.681 0.00 0.00 A2 H

ATOM 321 CB SER A 23 -10.631 13.287 11.275 1.00 0.00 A2 C

ATOM 322 HB1 SER A 23 -10.845 12.393 11.905 0.00 0.00 A2 H

ATOM 323 HB2 SER A 23 -10.211 14.069 11.948 0.00 0.00 A2 H

ATOM 324 OG SER A 23 -11.740 13.685 10.639 1.00 0.00 A2 O

ATOM 325 HG1 SER A 23 -12.447 13.720 11.296 0.00 0.00 A2 H

ATOM 326 C SER A 23 -10.092 11.709 9.391 1.00 0.00 A2 C

ATOM 327 O SER A 23 -10.067 10.609 10.012 1.00 0.00 A2 O

ATOM 328 N ASN A 24 -10.477 11.827 8.169 1.00 0.00 A2 N

ATOM 329 HN ASN A 24 -10.456 12.708 7.699 0.00 0.00 A2 H

ATOM 330 CA ASN A 24 -10.972 10.722 7.359 1.00 0.00 A2 C

ATOM 331 HA ASN A 24 -11.426 10.048 8.078 0.00 0.00 A2 H

ATOM 332 CB ASN A 24 -12.117 11.101 6.427 1.00 0.00 A2 C

ATOM 333 HB1 ASN A 24 -11.922 12.128 6.048 0.00 0.00 A2 H

ATOM 334 HB2 ASN A 24 -12.142 10.422 5.550 0.00 0.00 A2 H

ATOM 335 CG ASN A 24 -13.437 11.074 7.241 1.00 0.00 A2 C

ATOM 336 OD1 ASN A 24 -13.835 9.988 7.723 1.00 0.00 A2 O

ATOM 337 ND2 ASN A 24 -14.047 12.205 7.367 1.00 0.00 A2 N

ATOM 338 1HD2 ASN A 24 -14.891 12.213 7.896 0.00 0.00 A2 H

ATOM 339 2HD2 ASN A 24 -13.656 13.029 6.969 0.00 0.00 A2 H

ATOM 340 C ASN A 24 -9.884 9.932 6.600 1.00 0.00 A2 C

ATOM 341 O ASN A 24 -10.250 9.016 5.862 1.00 0.00 A2 O

ATOM 342 N TYR A 25 -8.662 10.384 6.717 1.00 0.00 A2 N

ATOM 343 HN TYR A 25 -8.361 11.150 7.282 0.00 0.00 A2 H

ATOM 344 CA TYR A 25 -7.582 9.723 5.952 1.00 0.00 A2 C

ATOM 345 HA TYR A 25 -7.792 9.920 4.908 0.00 0.00 A2 H

ATOM 346 CB TYR A 25 -6.230 10.385 6.291 1.00 0.00 A2 C

ATOM 347 HB1 TYR A 25 -6.252 11.447 5.964 0.00 0.00 A2 H

ATOM 348 HB2 TYR A 25 -6.056 10.383 7.389 0.00 0.00 A2 H

ATOM 349 CG TYR A 25 -5.064 9.736 5.595 1.00 0.00 A2 C

ATOM 350 CD1 TYR A 25 -4.655 10.156 4.316 1.00 0.00 A2 C

ATOM 351 HD1 TYR A 25 -5.185 10.956 3.818 0.00 0.00 A2 H

ATOM 352 CE1 TYR A 25 -3.566 9.518 3.673 1.00 0.00 A2 C

ATOM 353 HE1 TYR A 25 -3.213 9.867 2.714 0.00 0.00 A2 H

ATOM 354 CZ TYR A 25 -2.970 8.438 4.291 1.00 0.00 A2 C

ATOM 355 OH TYR A 25 -1.919 7.758 3.673 1.00 0.00 A2 O

ATOM 356 HH TYR A 25 -1.602 7.095 4.290 0.00 0.00 A2 H

ATOM 357 CD2 TYR A 25 -4.416 8.628 6.164 1.00 0.00 A2 C

ATOM 358 HD2 TYR A 25 -4.763 8.223 7.104 0.00 0.00 A2 H

ATOM 359 CE2 TYR A 25 -3.352 7.994 5.518 1.00 0.00 A2 C

ATOM 360 HE2 TYR A 25 -2.864 7.153 5.988 0.00 0.00 A2 H

ATOM 361 C TYR A 25 -7.586 8.214 6.133 1.00 0.00 A2 C

ATOM 362 O TYR A 25 -7.575 7.450 5.145 1.00 0.00 A2 O

ATOM 363 CB CYS A 26 -7.261 6.006 9.097 1.00 0.00 A2 C

ATOM 364 SG CYS A 26 -5.577 6.395 9.629 1.00 0.00 A2 S

ATOM 365 N CYS A 26 -7.538 7.787 7.368 1.00 0.00 A2 N

ATOM 366 HN CYS A 26 -7.535 8.373 8.176 0.00 0.00 A2 H

ATOM 367 CA CYS A 26 -7.483 6.339 7.653 1.00 0.00 A2 C

ATOM 368 HA CYS A 26 -6.599 5.967 7.148 0.00 0.00 A2 H

ATOM 369 HB1 CYS A 26 -7.984 6.611 9.690 0.00 0.00 A2 H

ATOM 370 HB2 CYS A 26 -7.525 4.942 9.284 0.00 0.00 A2 H

ATOM 371 C CYS A 26 -8.680 5.602 7.090 1.00 0.00 A2 C

ATOM 372 O CYS A 26 -8.593 4.541 6.438 1.00 0.00 A2 O

ATOM 373 N ASN A 27 -9.869 6.165 7.327 1.00 0.00 A2 N

ATOM 374 HN ASN A 27 -10.047 7.007 7.835 0.00 0.00 A2 H

ATOM 375 CA ASN A 27 -11.096 5.492 6.790 1.00 0.00 A2 C

ATOM 376 HA ASN A 27 -11.127 4.523 7.277 0.00 0.00 A2 H

ATOM 377 CB ASN A 27 -12.329 6.314 7.148 1.00 0.00 A2 C

ATOM 378 HB1 ASN A 27 -12.107 7.379 6.917 0.00 0.00 A2 H

ATOM 379 HB2 ASN A 27 -13.191 6.009 6.520 0.00 0.00 A2 H

ATOM 380 CG ASN A 27 -12.650 6.182 8.643 1.00 0.00 A2 C

ATOM 381 OD1 ASN A 27 -12.232 5.312 9.342 1.00 0.00 A2 O

ATOM 382 ND2 ASN A 27 -13.387 7.201 9.094 1.00 0.00 A2 N

ATOM 383 1HD2 ASN A 27 -13.630 7.198 10.060 0.00 0.00 A2 H

ATOM 384 2HD2 ASN A 27 -13.646 7.939 8.479 0.00 0.00 A2 H

ATOM 385 C ASN A 27 -11.019 5.252 5.295 1.00 0.00 A2 C

ATOM 386 O ASN A 27 -11.373 4.186 4.795 1.00 0.00 A2 O

ATOM 387 N GLN A 28 -10.607 6.308 4.579 1.00 0.00 A2 N

ATOM 388 HN GLN A 28 -10.345 7.171 5.008 0.00 0.00 A2 H

ATOM 389 CA GLN A 28 -10.502 6.301 3.127 1.00 0.00 A2 C

ATOM 390 HA GLN A 28 -11.460 5.956 2.758 0.00 0.00 A2 H

ATOM 391 CB GLN A 28 -10.223 7.721 2.599 1.00 0.00 A2 C

ATOM 392 HB1 GLN A 28 -9.265 8.054 3.062 0.00 0.00 A2 H

ATOM 393 HB2 GLN A 28 -10.039 7.677 1.501 0.00 0.00 A2 H

ATOM 394 CG GLN A 28 -11.312 8.709 2.924 1.00 0.00 A2 C

ATOM 395 HG1 GLN A 28 -12.154 8.608 2.206 0.00 0.00 A2 H

ATOM 396 HG2 GLN A 28 -11.711 8.532 3.944 0.00 0.00 A2 H

ATOM 397 CD GLN A 28 -10.894 10.155 2.844 1.00 0.00 A2 C

ATOM 398 OE1 GLN A 28 -11.674 11.143 2.765 1.00 0.00 A2 O

ATOM 399 NE2 GLN A 28 -9.539 10.379 2.839 1.00 0.00 A2 N

ATOM 400 1HE2 GLN A 28 -9.240 11.328 2.791 0.00 0.00 A2 H

ATOM 401 2HE2 GLN A 28 -8.910 9.609 2.866 0.00 0.00 A2 H

ATOM 402 C GLN A 28 -9.432 5.336 2.607 1.00 0.00 A2 C

ATOM 403 O GLN A 28 -9.708 4.492 1.726 1.00 0.00 A2 O

ATOM 404 N MET A 29 -8.267 5.434 3.216 1.00 0.00 A2 N

ATOM 405 HN MET A 29 -8.063 6.047 3.976 0.00 0.00 A2 H

ATOM 406 CA MET A 29 -7.140 4.603 2.790 1.00 0.00 A2 C

ATOM 407 HA MET A 29 -7.133 4.680 1.710 0.00 0.00 A2 H

ATOM 408 CB MET A 29 -5.838 5.178 3.330 1.00 0.00 A2 C

ATOM 409 HB1 MET A 29 -5.973 5.310 4.429 0.00 0.00 A2 H

ATOM 410 HB2 MET A 29 -5.024 4.430 3.203 0.00 0.00 A2 H

ATOM 411 CG MET A 29 -5.490 6.465 2.718 1.00 0.00 A2 C

ATOM 412 HG1 MET A 29 -6.338 7.177 2.808 0.00 0.00 A2 H

ATOM 413 HG2 MET A 29 -4.620 6.909 3.250 0.00 0.00 A2 H

ATOM 414 SD MET A 29 -5.069 6.415 0.982 1.00 0.00 A2 S

ATOM 415 CE MET A 29 -3.563 5.537 0.991 1.00 0.00 A2 C

ATOM 416 HE1 MET A 29 -3.160 5.430 -0.039 0.00 0.00 A2 H

ATOM 417 HE2 MET A 29 -2.799 6.062 1.604 0.00 0.00 A2 H

ATOM 418 HE3 MET A 29 -3.697 4.517 1.412 0.00 0.00 A2 H

ATOM 419 C MET A 29 -7.222 3.147 3.139 1.00 0.00 A2 C

ATOM 420 O MET A 29 -6.823 2.250 2.357 1.00 0.00 A2 O

ATOM 421 N MET A 30 -7.734 2.822 4.293 1.00 0.00 A2 N

ATOM 422 HN MET A 30 -8.060 3.484 4.965 0.00 0.00 A2 H

ATOM 423 CA MET A 30 -7.873 1.440 4.700 1.00 0.00 A2 C

ATOM 424 HA MET A 30 -6.896 0.977 4.639 0.00 0.00 A2 H

ATOM 425 CB MET A 30 -8.374 1.375 6.125 1.00 0.00 A2 C

ATOM 426 HB1 MET A 30 -9.263 2.046 6.187 0.00 0.00 A2 H

ATOM 427 HB2 MET A 30 -8.744 0.348 6.340 0.00 0.00 A2 H

ATOM 428 CG MET A 30 -7.354 1.810 7.166 1.00 0.00 A2 C

ATOM 429 HG1 MET A 30 -6.996 2.839 6.949 0.00 0.00 A2 H

ATOM 430 HG2 MET A 30 -7.829 1.824 8.171 0.00 0.00 A2 H

ATOM 431 SD MET A 30 -5.882 0.794 7.300 1.00 0.00 A2 S

ATOM 432 CE MET A 30 -6.427 -0.691 8.121 1.00 0.00 A2 C

ATOM 433 HE1 MET A 30 -5.581 -1.397 8.267 0.00 0.00 A2 H

ATOM 434 HE2 MET A 30 -6.855 -0.460 9.120 0.00 0.00 A2 H

ATOM 435 HE3 MET A 30 -7.208 -1.213 7.527 0.00 0.00 A2 H

ATOM 436 C MET A 30 -8.801 0.700 3.716 1.00 0.00 A2 C

ATOM 437 O MET A 30 -8.643 -0.470 3.343 1.00 0.00 A2 O

ATOM 438 N LYS A 31 -9.840 1.455 3.295 1.00 0.00 A2 N

ATOM 439 HN LYS A 31 -10.027 2.396 3.572 0.00 0.00 A2 H

ATOM 440 CA LYS A 31 -10.806 0.880 2.344 1.00 0.00 A2 C

ATOM 441 HA LYS A 31 -11.027 -0.124 2.686 0.00 0.00 A2 H

ATOM 442 CB LYS A 31 -12.086 1.715 2.303 1.00 0.00 A2 C

ATOM 443 HB1 LYS A 31 -12.350 1.962 3.357 0.00 0.00 A2 H

ATOM 444 HB2 LYS A 31 -11.870 2.684 1.799 0.00 0.00 A2 H

ATOM 445 CG LYS A 31 -13.143 0.828 1.604 1.00 0.00 A2 C

ATOM 446 HG1 LYS A 31 -12.785 0.573 0.581 0.00 0.00 A2 H

ATOM 447 HG2 LYS A 31 -13.245 -0.127 2.168 0.00 0.00 A2 H

ATOM 448 CD LYS A 31 -14.483 1.472 1.477 1.00 0.00 A2 C

ATOM 449 HD1 LYS A 31 -14.798 1.831 2.484 0.00 0.00 A2 H

ATOM 450 HD2 LYS A 31 -14.377 2.367 0.822 0.00 0.00 A2 H

ATOM 451 CE LYS A 31 -15.472 0.418 0.880 1.00 0.00 A2 C

ATOM 452 HE1 LYS A 31 -14.945 -0.541 0.677 0.00 0.00 A2 H

ATOM 453 HE2 LYS A 31 -16.305 0.218 1.589 0.00 0.00 A2 H

ATOM 454 NZ LYS A 31 -16.030 0.946 -0.393 1.00 0.00 A2 N

ATOM 455 HZ1 LYS A 31 -16.690 0.256 -0.806 0.00 0.00 A2 H

ATOM 456 HZ2 LYS A 31 -16.533 1.838 -0.208 0.00 0.00 A2 H

ATOM 457 HZ3 LYS A 31 -15.254 1.124 -1.063 0.00 0.00 A2 H

ATOM 458 C LYS A 31 -10.221 0.765 0.967 1.00 0.00 A2 C

ATOM 459 O LYS A 31 -10.319 -0.299 0.309 1.00 0.00 A2 O

ATOM 460 N SER A 32 -9.615 1.875 0.450 1.00 0.00 A2 N

ATOM 461 HN SER A 32 -9.513 2.730 0.958 0.00 0.00 A2 H

ATOM 462 CA SER A 32 -9.057 1.911 -0.904 1.00 0.00 A2 C

ATOM 463 HA SER A 32 -9.881 1.634 -1.548 0.00 0.00 A2 H

ATOM 464 CB SER A 32 -8.688 3.320 -1.399 1.00 0.00 A2 C

ATOM 465 HB1 SER A 32 -8.488 3.261 -2.493 0.00 0.00 A2 H

ATOM 466 HB2 SER A 32 -9.545 4.017 -1.260 0.00 0.00 A2 H

ATOM 467 OG SER A 32 -7.554 3.697 -0.712 1.00 0.00 A2 O

ATOM 468 HG1 SER A 32 -7.190 4.467 -1.166 0.00 0.00 A2 H

ATOM 469 C SER A 32 -7.949 0.919 -1.143 1.00 0.00 A2 C

ATOM 470 O SER A 32 -7.823 0.402 -2.319 1.00 0.00 A2 O

ATOM 471 N ARG A 33 -7.173 0.634 -0.132 1.00 0.00 A2 N

ATOM 472 HN ARG A 33 -7.295 1.080 0.752 0.00 0.00 A2 H

ATOM 473 CA ARG A 33 -6.093 -0.341 -0.229 1.00 0.00 A2 C

ATOM 474 HA ARG A 33 -5.717 -0.282 -1.243 0.00 0.00 A2 H

ATOM 475 CB ARG A 33 -4.949 0.132 0.657 1.00 0.00 A2 C

ATOM 476 HB1 ARG A 33 -5.371 0.242 1.685 0.00 0.00 A2 H

ATOM 477 HB2 ARG A 33 -4.177 -0.666 0.726 0.00 0.00 A2 H

ATOM 478 CG ARG A 33 -4.305 1.464 0.268 1.00 0.00 A2 C

ATOM 479 HG1 ARG A 33 -5.083 2.258 0.307 0.00 0.00 A2 H

ATOM 480 HG2 ARG A 33 -3.554 1.743 1.043 0.00 0.00 A2 H

ATOM 481 CD ARG A 33 -3.619 1.406 -1.049 1.00 0.00 A2 C

ATOM 482 HD1 ARG A 33 -2.861 0.590 -1.036 0.00 0.00 A2 H

ATOM 483 HD2 ARG A 33 -4.340 1.211 -1.877 0.00 0.00 A2 H

ATOM 484 NE ARG A 33 -2.941 2.626 -1.418 1.00 0.00 A2 N

ATOM 485 HE ARG A 33 -3.472 3.196 -2.055 0.00 0.00 A2 H

ATOM 486 CZ ARG A 33 -1.720 2.983 -0.966 1.00 0.00 A2 C

ATOM 487 NH1 ARG A 33 -0.985 2.161 -0.279 1.00 0.00 A2 N

ATOM 488 1HH1 ARG A 33 -0.102 2.444 0.068 0.00 0.00 A2 H

ATOM 489 2HH1 ARG A 33 -1.416 1.297 -0.012 0.00 0.00 A2 H

ATOM 490 NH2 ARG A 33 -1.254 4.179 -1.315 1.00 0.00 A2 N

ATOM 491 1HH2 ARG A 33 -0.329 4.438 -1.075 0.00 0.00 A2 H

ATOM 492 2HH2 ARG A 33 -1.779 4.687 -1.985 0.00 0.00 A2 H

ATOM 493 C ARG A 33 -6.497 -1.778 -0.001 1.00 0.00 A2 C

ATOM 494 O ARG A 33 -5.677 -2.676 0.077 1.00 0.00 A2 O

ATOM 495 N ASN A 34 -7.819 -1.974 0.166 1.00 0.00 A2 N

ATOM 496 HN ASN A 34 -8.508 -1.251 0.192 0.00 0.00 A2 H

ATOM 497 CA ASN A 34 -8.366 -3.359 0.332 1.00 0.00 A2 C

ATOM 498 HA ASN A 34 -9.442 -3.220 0.350 0.00 0.00 A2 H

ATOM 499 CB ASN A 34 -8.021 -4.117 -0.969 1.00 0.00 A2 C

ATOM 500 HB1 ASN A 34 -7.925 -3.367 -1.785 0.00 0.00 A2 H

ATOM 501 HB2 ASN A 34 -7.042 -4.628 -0.869 0.00 0.00 A2 H

ATOM 502 CG ASN A 34 -9.034 -5.120 -1.431 1.00 0.00 A2 C

ATOM 503 OD1 ASN A 34 -8.632 -6.216 -1.847 1.00 0.00 A2 O

ATOM 504 ND2 ASN A 34 -10.304 -4.757 -1.315 1.00 0.00 A2 N

ATOM 505 1HD2 ASN A 34 -10.996 -5.413 -1.605 0.00 0.00 A2 H

ATOM 506 2HD2 ASN A 34 -10.536 -3.872 -0.922 0.00 0.00 A2 H

ATOM 507 C ASN A 34 -7.984 -4.028 1.581 1.00 0.00 A2 C

ATOM 508 O ASN A 34 -7.942 -5.279 1.711 1.00 0.00 A2 O

ATOM 509 N LEU A 35 -7.763 -3.184 2.616 1.00 0.00 A2 N

ATOM 510 HN LEU A 35 -7.829 -2.190 2.566 0.00 0.00 A2 H

ATOM 511 CA LEU A 35 -7.390 -3.705 3.957 1.00 0.00 A2 C

ATOM 512 HA LEU A 35 -6.774 -4.574 3.766 0.00 0.00 A2 H

ATOM 513 CB LEU A 35 -6.456 -2.727 4.680 1.00 0.00 A2 C

ATOM 514 HB1 LEU A 35 -7.056 -1.820 4.926 0.00 0.00 A2 H

ATOM 515 HB2 LEU A 35 -6.154 -3.169 5.655 0.00 0.00 A2 H

ATOM 516 CG LEU A 35 -5.227 -2.345 3.792 1.00 0.00 A2 C

ATOM 517 HG LEU A 35 -5.598 -1.949 2.952 0.00 0.00 A2 H

ATOM 518 CD1 LEU A 35 -4.396 -1.328 4.515 1.00 0.00 A2 C

ATOM 519 1HD1 LEU A 35 -3.489 -1.075 3.925 0.00 0.00 A2 H

ATOM 520 2HD1 LEU A 35 -4.976 -0.395 4.681 0.00 0.00 A2 H

ATOM 521 3HD1 LEU A 35 -4.069 -1.718 5.502 0.00 0.00 A2 H

ATOM 522 CD2 LEU A 35 -4.422 -3.482 3.273 1.00 0.00 A2 C

ATOM 523 1HD2 LEU A 35 -3.587 -3.108 2.642 0.00 0.00 A2 H

ATOM 524 2HD2 LEU A 35 -3.980 -4.080 4.095 0.00 0.00 A2 H

ATOM 525 3HD2 LEU A 35 -5.053 -4.154 2.652 0.00 0.00 A2 H

ATOM 526 C LEU A 35 -8.565 -4.154 4.792 1.00 0.00 A2 C

ATOM 527 O LEU A 35 -8.321 -4.926 5.764 1.00 0.00 A2 O

ATOM 528 N THR A 36 -9.722 -3.749 4.393 1.00 0.00 A2 N

ATOM 529 HN THR A 36 -9.870 -3.199 3.571 0.00 0.00 A2 H

ATOM 530 CA THR A 36 -10.970 -4.076 5.145 1.00 0.00 A2 C

ATOM 531 HA THR A 36 -10.627 -4.610 6.021 0.00 0.00 A2 H

ATOM 532 CB THR A 36 -11.652 -2.730 5.451 1.00 0.00 A2 C

ATOM 533 HB THR A 36 -12.522 -2.966 6.111 0.00 0.00 A2 H

ATOM 534 OG1 THR A 36 -12.174 -2.094 4.361 1.00 0.00 A2 O

ATOM 535 HG1 THR A 36 -12.551 -1.274 4.698 0.00 0.00 A2 H

ATOM 536 CG2 THR A 36 -10.707 -1.815 6.247 1.00 0.00 A2 C

ATOM 537 1HG2 THR A 36 -11.230 -0.890 6.569 0.00 0.00 A2 H

ATOM 538 2HG2 THR A 36 -10.341 -2.335 7.158 0.00 0.00 A2 H

ATOM 539 3HG2 THR A 36 -9.826 -1.522 5.637 0.00 0.00 A2 H

ATOM 540 C THR A 36 -11.918 -5.015 4.408 1.00 0.00 A2 C

ATOM 541 O THR A 36 -13.111 -5.048 4.734 1.00 0.00 A2 O

ATOM 542 N LYS A 37 -11.347 -5.697 3.432 1.00 0.00 A2 N

ATOM 543 HN LYS A 37 -10.371 -5.701 3.220 0.00 0.00 A2 H

ATOM 544 CA LYS A 37 -12.192 -6.548 2.544 1.00 0.00 A2 C

ATOM 545 HA LYS A 37 -13.014 -5.929 2.205 0.00 0.00 A2 H

ATOM 546 CB LYS A 37 -11.353 -6.939 1.316 1.00 0.00 A2 C

ATOM 547 HB1 LYS A 37 -11.063 -5.993 0.803 0.00 0.00 A2 H

ATOM 548 HB2 LYS A 37 -10.406 -7.411 1.660 0.00 0.00 A2 H

ATOM 549 CG LYS A 37 -12.116 -7.845 0.345 1.00 0.00 A2 C

ATOM 550 HG1 LYS A 37 -12.421 -8.775 0.877 0.00 0.00 A2 H

ATOM 551 HG2 LYS A 37 -13.048 -7.327 0.021 0.00 0.00 A2 H

ATOM 552 CD LYS A 37 -11.288 -8.228 -0.866 1.00 0.00 A2 C

ATOM 553 HD1 LYS A 37 -11.969 -8.655 -1.637 0.00 0.00 A2 H

ATOM 554 HD2 LYS A 37 -10.844 -7.298 -1.291 0.00 0.00 A2 H

ATOM 555 CE LYS A 37 -10.176 -9.215 -0.585 1.00 0.00 A2 C

ATOM 556 HE1 LYS A 37 -9.324 -9.051 -1.282 0.00 0.00 A2 H

ATOM 557 HE2 LYS A 37 -9.805 -9.099 0.457 0.00 0.00 A2 H

ATOM 558 NZ LYS A 37 -10.698 -10.619 -0.769 1.00 0.00 A2 N

ATOM 559 HZ1 LYS A 37 -9.942 -11.308 -0.580 0.00 0.00 A2 H

ATOM 560 HZ2 LYS A 37 -11.488 -10.784 -0.113 0.00 0.00 A2 H

ATOM 561 HZ3 LYS A 37 -11.035 -10.737 -1.746 0.00 0.00 A2 H

ATOM 562 C LYS A 37 -12.787 -7.717 3.290 1.00 0.00 A2 C

ATOM 563 O LYS A 37 -14.055 -7.925 3.098 1.00 0.00 A2 O

ATOM 564 N ASP A 38 -12.008 -8.436 3.993 1.00 0.00 A2 N

ATOM 565 HN ASP A 38 -11.038 -8.239 4.110 0.00 0.00 A2 H

ATOM 566 CA ASP A 38 -12.441 -9.646 4.719 1.00 0.00 A2 C

ATOM 567 HA ASP A 38 -13.331 -9.987 4.203 0.00 0.00 A2 H

ATOM 568 CB ASP A 38 -11.458 -10.752 4.682 1.00 0.00 A2 C

ATOM 569 HB1 ASP A 38 -10.492 -10.395 5.092 0.00 0.00 A2 H

ATOM 570 HB2 ASP A 38 -11.805 -11.604 5.300 0.00 0.00 A2 H

ATOM 571 CG ASP A 38 -11.195 -11.254 3.281 1.00 0.00 A2 C

ATOM 572 OD1 ASP A 38 -9.989 -11.349 2.957 1.00 0.00 A2 O

ATOM 573 OD2 ASP A 38 -12.235 -11.495 2.500 1.00 0.00 A2 O

ATOM 574 C ASP A 38 -12.832 -9.418 6.156 1.00 0.00 A2 C

ATOM 575 O ASP A 38 -13.689 -10.158 6.718 1.00 0.00 A2 O

ATOM 576 N ARG A 39 -12.195 -8.411 6.757 1.00 0.00 A2 N

ATOM 577 HN ARG A 39 -11.544 -7.863 6.237 0.00 0.00 A2 H

ATOM 578 CA ARG A 39 -12.389 -8.043 8.164 1.00 0.00 A2 C

ATOM 579 HA ARG A 39 -13.448 -7.935 8.369 0.00 0.00 A2 H

ATOM 580 CB ARG A 39 -11.644 -9.083 9.053 1.00 0.00 A2 C

ATOM 581 HB1 ARG A 39 -11.865 -8.808 10.112 0.00 0.00 A2 H

ATOM 582 HB2 ARG A 39 -12.089 -10.091 8.899 0.00 0.00 A2 H

ATOM 583 CG ARG A 39 -10.125 -9.071 8.823 1.00 0.00 A2 C

ATOM 584 HG1 ARG A 39 -9.931 -8.820 7.757 0.00 0.00 A2 H

ATOM 585 HG2 ARG A 39 -9.676 -8.239 9.413 0.00 0.00 A2 H

ATOM 586 CD ARG A 39 -9.368 -10.273 9.192 1.00 0.00 A2 C

ATOM 587 HD1 ARG A 39 -9.706 -10.631 10.191 0.00 0.00 A2 H

ATOM 588 HD2 ARG A 39 -9.515 -11.092 8.450 0.00 0.00 A2 H

ATOM 589 NE ARG A 39 -7.910 -9.972 9.235 1.00 0.00 A2 N

ATOM 590 HE ARG A 39 -7.421 -10.264 8.405 0.00 0.00 A2 H

ATOM 591 CZ ARG A 39 -7.252 -9.380 10.236 1.00 0.00 A2 C

ATOM 592 NH1 ARG A 39 -7.838 -8.864 11.316 1.00 0.00 A2 N

ATOM 593 1HH1 ARG A 39 -7.300 -8.414 12.015 0.00 0.00 A2 H

ATOM 594 2HH1 ARG A 39 -8.840 -8.837 11.307 0.00 0.00 A2 H

ATOM 595 NH2 ARG A 39 -5.910 -9.342 10.174 1.00 0.00 A2 N

ATOM 596 1HH2 ARG A 39 -5.390 -8.989 10.939 0.00 0.00 A2 H

ATOM 597 2HH2 ARG A 39 -5.480 -9.863 9.448 0.00 0.00 A2 H

ATOM 598 C ARG A 39 -11.753 -6.662 8.399 1.00 0.00 A2 C

ATOM 599 O ARG A 39 -10.993 -6.209 7.581 1.00 0.00 A2 O

ATOM 600 CB CYS A 40 -12.230 -3.943 10.777 1.00 0.00 A2 C

ATOM 601 SG CYS A 40 -13.973 -3.676 10.365 1.00 0.00 A2 S

ATOM 602 N CYS A 40 -12.103 -6.046 9.493 1.00 0.00 A2 N

ATOM 603 HN CYS A 40 -12.775 -6.333 10.173 0.00 0.00 A2 H

ATOM 604 CA CYS A 40 -11.431 -4.751 9.789 1.00 0.00 A2 C

ATOM 605 HA CYS A 40 -11.268 -4.223 8.857 0.00 0.00 A2 H

ATOM 606 HB1 CYS A 40 -12.199 -4.486 11.749 0.00 0.00 A2 H

ATOM 607 HB2 CYS A 40 -11.731 -2.965 10.956 0.00 0.00 A2 H

ATOM 608 C CYS A 40 -10.052 -5.106 10.373 1.00 0.00 A2 C

ATOM 609 O CYS A 40 -9.980 -5.690 11.457 1.00 0.00 A2 O

ATOM 610 N LYS A 41 -9.044 -4.709 9.675 1.00 0.00 A2 N

ATOM 611 HN LYS A 41 -9.104 -4.297 8.767 0.00 0.00 A2 H

ATOM 612 CA LYS A 41 -7.634 -4.855 10.226 1.00 0.00 A2 C

ATOM 613 HA LYS A 41 -7.502 -5.851 10.632 0.00 0.00 A2 H

ATOM 614 CB LYS A 41 -6.707 -4.448 9.089 1.00 0.00 A2 C

ATOM 615 HB1 LYS A 41 -6.945 -5.105 8.221 0.00 0.00 A2 H

ATOM 616 HB2 LYS A 41 -6.956 -3.410 8.772 0.00 0.00 A2 H

ATOM 617 CG LYS A 41 -5.263 -4.568 9.406 1.00 0.00 A2 C

ATOM 618 HG1 LYS A 41 -5.024 -3.909 10.271 0.00 0.00 A2 H

ATOM 619 HG2 LYS A 41 -5.046 -5.616 9.717 0.00 0.00 A2 H

ATOM 620 CD LYS A 41 -4.223 -4.216 8.345 1.00 0.00 A2 C

ATOM 621 HD1 LYS A 41 -4.580 -4.599 7.362 0.00 0.00 A2 H

ATOM 622 HD2 LYS A 41 -4.167 -3.105 8.270 0.00 0.00 A2 H

ATOM 623 CE LYS A 41 -2.861 -4.815 8.722 1.00 0.00 A2 C

ATOM 624 HE1 LYS A 41 -2.863 -5.152 9.783 0.00 0.00 A2 H

ATOM 625 HE2 LYS A 41 -2.626 -5.690 8.076 0.00 0.00 A2 H

ATOM 626 NZ LYS A 41 -1.699 -3.908 8.598 1.00 0.00 A2 N

ATOM 627 HZ1 LYS A 41 -0.828 -4.405 8.876 0.00 0.00 A2 H

ATOM 628 HZ2 LYS A 41 -1.613 -3.586 7.613 0.00 0.00 A2 H

ATOM 629 HZ3 LYS A 41 -1.837 -3.083 9.216 0.00 0.00 A2 H

ATOM 630 C LYS A 41 -7.551 -3.884 11.378 1.00 0.00 A2 C

ATOM 631 O LYS A 41 -7.909 -2.682 11.165 1.00 0.00 A2 O

ATOM 632 N PRO A 42 -7.179 -4.190 12.565 1.00 0.00 A2 N

ATOM 633 CD PRO A 42 -6.816 -5.623 12.987 1.00 0.00 A2 C

ATOM 634 HD1 PRO A 42 -7.656 -6.354 12.994 0.00 0.00 A2 H

ATOM 635 HD2 PRO A 42 -6.001 -5.973 12.312 0.00 0.00 A2 H

ATOM 636 CA PRO A 42 -7.194 -3.363 13.699 1.00 0.00 A2 C

ATOM 637 HA PRO A 42 -8.220 -3.033 13.807 0.00 0.00 A2 H

ATOM 638 CB PRO A 42 -7.023 -4.249 14.923 1.00 0.00 A2 C

ATOM 639 HB1 PRO A 42 -8.018 -4.645 15.226 0.00 0.00 A2 H

ATOM 640 HB2 PRO A 42 -6.572 -3.711 15.782 0.00 0.00 A2 H

ATOM 641 CG PRO A 42 -6.285 -5.435 14.380 1.00 0.00 A2 C

ATOM 642 HG1 PRO A 42 -6.422 -6.347 14.994 0.00 0.00 A2 H

ATOM 643 HG2 PRO A 42 -5.200 -5.192 14.313 0.00 0.00 A2 H

ATOM 644 C PRO A 42 -6.267 -2.151 13.787 1.00 0.00 A2 C

ATOM 645 O PRO A 42 -6.630 -1.111 14.384 1.00 0.00 A2 O

ATOM 646 N VAL A 43 -5.108 -2.323 13.194 1.00 0.00 A2 N

ATOM 647 HN VAL A 43 -4.852 -3.153 12.706 0.00 0.00 A2 H

ATOM 648 CA VAL A 43 -4.080 -1.287 13.204 1.00 0.00 A2 C

ATOM 649 HA VAL A 43 -4.569 -0.324 13.276 0.00 0.00 A2 H

ATOM 650 CB VAL A 43 -2.999 -1.508 14.299 1.00 0.00 A2 C

ATOM 651 HB VAL A 43 -2.346 -2.351 13.963 0.00 0.00 A2 H

ATOM 652 CG1 VAL A 43 -2.102 -0.264 14.522 1.00 0.00 A2 C

ATOM 653 1HG1 VAL A 43 -1.373 -0.456 15.338 0.00 0.00 A2 H

ATOM 654 2HG1 VAL A 43 -1.520 -0.010 13.612 0.00 0.00 A2 H

ATOM 655 3HG1 VAL A 43 -2.716 0.616 14.810 0.00 0.00 A2 H

ATOM 656 CG2 VAL A 43 -3.458 -2.088 15.608 1.00 0.00 A2 C

ATOM 657 1HG2 VAL A 43 -2.611 -2.163 16.323 0.00 0.00 A2 H

ATOM 658 2HG2 VAL A 43 -4.245 -1.449 16.064 0.00 0.00 A2 H

ATOM 659 3HG2 VAL A 43 -3.873 -3.108 15.472 0.00 0.00 A2 H

ATOM 660 C VAL A 43 -3.322 -1.319 11.874 1.00 0.00 A2 C

ATOM 661 O VAL A 43 -3.120 -2.407 11.335 1.00 0.00 A2 O

ATOM 662 N ASN A 44 -2.894 -0.127 11.476 1.00 0.00 A2 N

ATOM 663 HN ASN A 44 -3.071 0.758 11.904 0.00 0.00 A2 H

ATOM 664 CA ASN A 44 -2.067 -0.064 10.280 1.00 0.00 A2 C

ATOM 665 HA ASN A 44 -1.348 -0.874 10.343 0.00 0.00 A2 H

ATOM 666 CB ASN A 44 -2.882 -0.267 9.033 1.00 0.00 A2 C

ATOM 667 HB1 ASN A 44 -3.510 -1.173 9.180 0.00 0.00 A2 H

ATOM 668 HB2 ASN A 44 -3.567 0.591 8.876 0.00 0.00 A2 H

ATOM 669 CG ASN A 44 -2.024 -0.486 7.797 1.00 0.00 A2 C

ATOM 670 OD1 ASN A 44 -1.158 -1.373 7.836 1.00 0.00 A2 O

ATOM 671 ND2 ASN A 44 -2.155 0.347 6.761 1.00 0.00 A2 N

ATOM 672 1HD2 ASN A 44 -1.589 0.174 5.960 0.00 0.00 A2 H

ATOM 673 2HD2 ASN A 44 -2.820 1.087 6.797 0.00 0.00 A2 H

ATOM 674 C ASN A 44 -1.273 1.275 10.312 1.00 0.00 A2 C

ATOM 675 O ASN A 44 -1.746 2.230 10.905 1.00 0.00 A2 O

ATOM 676 N THR A 45 -0.147 1.278 9.666 1.00 0.00 A2 N

ATOM 677 HN THR A 45 0.214 0.458 9.224 0.00 0.00 A2 H

ATOM 678 CA THR A 45 0.705 2.471 9.518 1.00 0.00 A2 C

ATOM 679 HA THR A 45 0.128 3.314 9.874 0.00 0.00 A2 H

ATOM 680 CB THR A 45 2.080 2.319 10.211 1.00 0.00 A2 C

ATOM 681 HB THR A 45 2.584 1.454 9.714 0.00 0.00 A2 H

ATOM 682 OG1 THR A 45 1.808 1.991 11.569 1.00 0.00 A2 O

ATOM 683 HG1 THR A 45 2.670 1.911 11.991 0.00 0.00 A2 H

ATOM 684 CG2 THR A 45 2.928 3.590 10.183 1.00 0.00 A2 C

ATOM 685 1HG2 THR A 45 3.943 3.397 10.589 0.00 0.00 A2 H

ATOM 686 2HG2 THR A 45 3.042 3.957 9.141 0.00 0.00 A2 H

ATOM 687 3HG2 THR A 45 2.460 4.397 10.787 0.00 0.00 A2 H

ATOM 688 C THR A 45 0.965 2.720 8.021 1.00 0.00 A2 C

ATOM 689 O THR A 45 1.311 1.781 7.300 1.00 0.00 A2 O

ATOM 690 N PHE A 46 0.773 3.936 7.616 1.00 0.00 A2 N

ATOM 691 HN PHE A 46 0.365 4.624 8.214 0.00 0.00 A2 H

ATOM 692 CA PHE A 46 1.125 4.430 6.264 1.00 0.00 A2 C

ATOM 693 HA PHE A 46 1.405 3.572 5.665 0.00 0.00 A2 H

ATOM 694 CB PHE A 46 0.006 5.218 5.598 1.00 0.00 A2 C

ATOM 695 HB1 PHE A 46 -0.372 5.985 6.309 0.00 0.00 A2 H

ATOM 696 HB2 PHE A 46 0.376 5.758 4.700 0.00 0.00 A2 H

ATOM 697 CG PHE A 46 -1.157 4.319 5.241 1.00 0.00 A2 C

ATOM 698 CD1 PHE A 46 -1.124 3.594 4.066 1.00 0.00 A2 C

ATOM 699 HD1 PHE A 46 -0.282 3.700 3.396 0.00 0.00 A2 H

ATOM 700 CE1 PHE A 46 -2.202 2.725 3.755 1.00 0.00 A2 C

ATOM 701 HE1 PHE A 46 -2.153 2.095 2.878 0.00 0.00 A2 H

ATOM 702 CZ PHE A 46 -3.301 2.698 4.592 1.00 0.00 A2 C

ATOM 703 HZ PHE A 46 -4.130 2.053 4.336 0.00 0.00 A2 H

ATOM 704 CD2 PHE A 46 -2.244 4.254 6.073 1.00 0.00 A2 C

ATOM 705 HD2 PHE A 46 -2.260 4.832 6.987 0.00 0.00 A2 H

ATOM 706 CE2 PHE A 46 -3.353 3.481 5.766 1.00 0.00 A2 C

ATOM 707 HE2 PHE A 46 -4.216 3.495 6.417 0.00 0.00 A2 H

ATOM 708 C PHE A 46 2.406 5.342 6.393 1.00 0.00 A2 C

ATOM 709 O PHE A 46 2.542 6.108 7.333 1.00 0.00 A2 O

ATOM 710 N VAL A 47 3.285 5.184 5.407 1.00 0.00 A2 N

ATOM 711 HN VAL A 47 3.165 4.553 4.645 0.00 0.00 A2 H

ATOM 712 CA VAL A 47 4.539 5.954 5.367 1.00 0.00 A2 C

ATOM 713 HA VAL A 47 4.615 6.527 6.283 0.00 0.00 A2 H

ATOM 714 CB VAL A 47 5.721 5.018 5.314 1.00 0.00 A2 C

ATOM 715 HB VAL A 47 5.643 4.429 4.367 0.00 0.00 A2 H

ATOM 716 CG1 VAL A 47 7.074 5.758 5.307 1.00 0.00 A2 C

ATOM 717 1HG1 VAL A 47 7.912 5.029 5.307 0.00 0.00 A2 H

ATOM 718 2HG1 VAL A 47 7.189 6.393 4.405 0.00 0.00 A2 H

ATOM 719 3HG1 VAL A 47 7.176 6.399 6.209 0.00 0.00 A2 H

ATOM 720 CG2 VAL A 47 5.674 3.893 6.357 1.00 0.00 A2 C

ATOM 721 1HG2 VAL A 47 6.586 3.262 6.296 0.00 0.00 A2 H

ATOM 722 2HG2 VAL A 47 5.608 4.315 7.383 0.00 0.00 A2 H

ATOM 723 3HG2 VAL A 47 4.798 3.231 6.195 0.00 0.00 A2 H

ATOM 724 C VAL A 47 4.481 6.959 4.195 1.00 0.00 A2 C

ATOM 725 O VAL A 47 4.300 6.564 3.040 1.00 0.00 A2 O

ATOM 726 N HSP A 48 4.704 8.212 4.514 1.00 0.00 A2 N

ATOM 727 HN HSP A 48 4.918 8.440 5.468 0.00 0.00 A2 H

ATOM 728 CA HSP A 48 4.652 9.285 3.531 1.00 0.00 A2 C

ATOM 729 HA HSP A 48 4.095 8.924 2.675 0.00 0.00 A2 H

ATOM 730 CB HSP A 48 3.899 10.469 4.148 1.00 0.00 A2 C

ATOM 731 HB1 HSP A 48 4.499 10.881 4.988 0.00 0.00 A2 H

ATOM 732 HB2 HSP A 48 3.814 11.279 3.389 0.00 0.00 A2 H

ATOM 733 CD2 HSP A 48 2.100 9.987 5.935 1.00 0.00 A2 C

ATOM 734 HD2 HSP A 48 2.660 10.127 6.856 0.00 0.00 A2 H

ATOM 735 CG HSP A 48 2.525 10.163 4.648 1.00 0.00 A2 C

ATOM 736 NE2 HSP A 48 0.767 9.711 5.856 1.00 0.00 A2 N

ATOM 737 HE2 HSP A 48 0.151 9.585 6.636 0.00 0.00 A2 H

ATOM 738 ND1 HSP A 48 1.491 10.070 3.806 1.00 0.00 A2 N

ATOM 739 HD1 HSP A 48 1.502 10.299 2.831 0.00 0.00 A2 H

ATOM 740 CE1 HSP A 48 0.427 9.797 4.584 1.00 0.00 A2 C

ATOM 741 HE1 HSP A 48 -0.592 9.684 4.243 0.00 0.00 A2 H

ATOM 742 C HSP A 48 6.008 9.731 3.013 1.00 0.00 A2 C

ATOM 743 O HSP A 48 6.465 10.879 3.298 1.00 0.00 A2 O

ATOM 744 N GLU A 49 6.691 8.830 2.409 1.00 0.00 A2 N

ATOM 745 HN GLU A 49 6.291 7.932 2.248 0.00 0.00 A2 H

ATOM 746 CA GLU A 49 8.036 8.970 1.911 1.00 0.00 A2 C

ATOM 747 HA GLU A 49 8.244 10.020 1.748 0.00 0.00 A2 H

ATOM 748 CB GLU A 49 9.051 8.326 2.899 1.00 0.00 A2 C

ATOM 749 HB1 GLU A 49 8.813 7.240 2.983 0.00 0.00 A2 H

ATOM 750 HB2 GLU A 49 10.078 8.381 2.474 0.00 0.00 A2 H

ATOM 751 CG GLU A 49 9.076 8.910 4.314 1.00 0.00 A2 C

ATOM 752 HG1 GLU A 49 8.053 8.905 4.733 0.00 0.00 A2 H

ATOM 753 HG2 GLU A 49 9.744 8.322 4.974 0.00 0.00 A2 H

ATOM 754 CD GLU A 49 9.501 10.350 4.525 1.00 0.00 A2 C

ATOM 755 OE1 GLU A 49 9.247 10.977 5.540 1.00 0.00 A2 O

ATOM 756 OE2 GLU A 49 10.127 10.883 3.506 1.00 0.00 A2 O

ATOM 757 C GLU A 49 8.146 8.285 0.588 1.00 0.00 A2 C

ATOM 758 O GLU A 49 7.305 7.488 0.151 1.00 0.00 A2 O

ATOM 759 N SER A 50 9.229 8.592 -0.112 1.00 0.00 A2 N

ATOM 760 HN SER A 50 9.923 9.264 0.144 0.00 0.00 A2 H

ATOM 761 CA SER A 50 9.491 7.910 -1.402 1.00 0.00 A2 C

ATOM 762 HA SER A 50 8.595 7.984 -2.005 0.00 0.00 A2 H

ATOM 763 CB SER A 50 10.682 8.502 -2.169 1.00 0.00 A2 C

ATOM 764 HB1 SER A 50 10.688 8.062 -3.192 0.00 0.00 A2 H

ATOM 765 HB2 SER A 50 10.559 9.602 -2.287 0.00 0.00 A2 H

ATOM 766 OG SER A 50 11.832 8.173 -1.515 1.00 0.00 A2 O

ATOM 767 HG1 SER A 50 12.563 8.355 -2.118 0.00 0.00 A2 H

ATOM 768 C SER A 50 9.712 6.395 -1.081 1.00 0.00 A2 C

ATOM 769 O SER A 50 10.248 6.005 -0.049 1.00 0.00 A2 O

ATOM 770 N LEU A 51 9.311 5.572 -2.052 1.00 0.00 A2 N

ATOM 771 HN LEU A 51 8.846 5.852 -2.889 0.00 0.00 A2 H

ATOM 772 CA LEU A 51 9.541 4.127 -1.945 1.00 0.00 A2 C

ATOM 773 HA LEU A 51 9.090 3.796 -1.018 0.00 0.00 A2 H

ATOM 774 CB LEU A 51 8.873 3.352 -3.093 1.00 0.00 A2 C

ATOM 775 HB1 LEU A 51 7.781 3.569 -3.045 0.00 0.00 A2 H

ATOM 776 HB2 LEU A 51 9.226 3.767 -4.063 0.00 0.00 A2 H

ATOM 777 CG LEU A 51 9.047 1.855 -3.092 1.00 0.00 A2 C

ATOM 778 HG LEU A 51 10.030 1.684 -3.032 0.00 0.00 A2 H

ATOM 779 CD1 LEU A 51 8.427 1.192 -1.909 1.00 0.00 A2 C

ATOM 780 1HD1 LEU A 51 8.539 0.089 -1.977 0.00 0.00 A2 H

ATOM 781 2HD1 LEU A 51 8.912 1.535 -0.970 0.00 0.00 A2 H

ATOM 782 3HD1 LEU A 51 7.343 1.427 -1.851 0.00 0.00 A2 H

ATOM 783 CD2 LEU A 51 8.503 1.256 -4.380 1.00 0.00 A2 C

ATOM 784 1HD2 LEU A 51 8.649 0.154 -4.385 0.00 0.00 A2 H

ATOM 785 2HD2 LEU A 51 7.418 1.452 -4.497 0.00 0.00 A2 H

ATOM 786 3HD2 LEU A 51 9.032 1.681 -5.260 0.00 0.00 A2 H

ATOM 787 C LEU A 51 11.021 3.850 -1.855 1.00 0.00 A2 C

ATOM 788 O LEU A 51 11.453 2.994 -1.078 1.00 0.00 A2 O

ATOM 789 N ALA A 52 11.865 4.528 -2.634 1.00 0.00 A2 N

ATOM 790 HN ALA A 52 11.567 5.195 -3.317 0.00 0.00 A2 H

ATOM 791 CA ALA A 52 13.309 4.334 -2.535 1.00 0.00 A2 C

ATOM 792 HA ALA A 52 13.551 3.304 -2.770 0.00 0.00 A2 H

ATOM 793 CB ALA A 52 14.011 5.186 -3.590 1.00 0.00 A2 C

ATOM 794 HB1 ALA A 52 13.616 4.933 -4.597 0.00 0.00 A2 H

ATOM 795 HB2 ALA A 52 13.830 6.269 -3.414 0.00 0.00 A2 H

ATOM 796 HB3 ALA A 52 15.108 5.007 -3.597 0.00 0.00 A2 H

ATOM 797 C ALA A 52 13.862 4.597 -1.158 1.00 0.00 A2 C

ATOM 798 O ALA A 52 14.731 3.820 -0.681 1.00 0.00 A2 O

ATOM 799 N ASP A 53 13.414 5.640 -0.450 1.00 0.00 A2 N

ATOM 800 HN ASP A 53 12.743 6.290 -0.795 0.00 0.00 A2 H

ATOM 801 CA ASP A 53 13.844 5.937 0.871 1.00 0.00 A2 C

ATOM 802 HA ASP A 53 14.927 5.981 0.844 0.00 0.00 A2 H

ATOM 803 CB ASP A 53 13.421 7.286 1.422 1.00 0.00 A2 C

ATOM 804 HB1 ASP A 53 12.336 7.429 1.245 0.00 0.00 A2 H

ATOM 805 HB2 ASP A 53 13.606 7.337 2.513 0.00 0.00 A2 H

ATOM 806 CG ASP A 53 14.128 8.451 0.775 1.00 0.00 A2 C

ATOM 807 OD1 ASP A 53 15.144 8.315 0.118 1.00 0.00 A2 O

ATOM 808 OD2 ASP A 53 13.635 9.592 0.996 1.00 0.00 A2 O

ATOM 809 C ASP A 53 13.464 4.803 1.876 1.00 0.00 A2 C

ATOM 810 O ASP A 53 14.213 4.583 2.799 1.00 0.00 A2 O

ATOM 811 N VAL A 54 12.289 4.239 1.664 1.00 0.00 A2 N

ATOM 812 HN VAL A 54 11.635 4.470 0.949 0.00 0.00 A2 H

ATOM 813 CA VAL A 54 11.858 3.149 2.553 1.00 0.00 A2 C

ATOM 814 HA VAL A 54 12.115 3.429 3.567 0.00 0.00 A2 H

ATOM 815 CB VAL A 54 10.336 2.981 2.569 1.00 0.00 A2 C

ATOM 816 HB VAL A 54 9.995 2.890 1.508 0.00 0.00 A2 H

ATOM 817 CG1 VAL A 54 9.861 1.736 3.317 1.00 0.00 A2 C

ATOM 818 1HG1 VAL A 54 8.751 1.692 3.326 0.00 0.00 A2 H

ATOM 819 2HG1 VAL A 54 10.225 0.805 2.835 0.00 0.00 A2 H

ATOM 820 3HG1 VAL A 54 10.213 1.753 4.371 0.00 0.00 A2 H

ATOM 821 CG2 VAL A 54 9.660 4.245 3.084 1.00 0.00 A2 C

ATOM 822 1HG2 VAL A 54 8.559 4.109 3.133 0.00 0.00 A2 H

ATOM 823 2HG2 VAL A 54 10.027 4.497 4.102 0.00 0.00 A2 H

ATOM 824 3HG2 VAL A 54 9.862 5.109 2.417 0.00 0.00 A2 H

ATOM 825 C VAL A 54 12.628 1.844 2.221 1.00 0.00 A2 C

ATOM 826 O VAL A 54 13.012 1.145 3.154 1.00 0.00 A2 O

ATOM 827 N GLN A 55 12.792 1.591 0.951 1.00 0.00 A2 N

ATOM 828 HN GLN A 55 12.436 2.121 0.183 0.00 0.00 A2 H

ATOM 829 CA GLN A 55 13.591 0.391 0.557 1.00 0.00 A2 C

ATOM 830 HA GLN A 55 13.107 -0.469 1.004 0.00 0.00 A2 H

ATOM 831 CB GLN A 55 13.642 0.225 -0.946 1.00 0.00 A2 C

ATOM 832 HB1 GLN A 55 13.957 1.207 -1.368 0.00 0.00 A2 H

ATOM 833 HB2 GLN A 55 14.447 -0.499 -1.210 0.00 0.00 A2 H

ATOM 834 CG GLN A 55 12.312 -0.189 -1.545 1.00 0.00 A2 C

ATOM 835 HG1 GLN A 55 12.091 -1.251 -1.302 0.00 0.00 A2 H

ATOM 836 HG2 GLN A 55 11.488 0.432 -1.138 0.00 0.00 A2 H

ATOM 837 CD GLN A 55 12.337 -0.070 -3.072 1.00 0.00 A2 C

ATOM 838 OE1 GLN A 55 13.054 0.706 -3.703 1.00 0.00 A2 O

ATOM 839 NE2 GLN A 55 11.482 -0.820 -3.640 1.00 0.00 A2 N

ATOM 840 1HE2 GLN A 55 11.450 -0.793 -4.635 0.00 0.00 A2 H

ATOM 841 2HE2 GLN A 55 10.876 -1.384 -3.090 0.00 0.00 A2 H

ATOM 842 C GLN A 55 14.993 0.457 1.103 1.00 0.00 A2 C

ATOM 843 O GLN A 55 15.607 -0.548 1.519 1.00 0.00 A2 O

ATOM 844 N ALA A 56 15.569 1.676 1.155 1.00 0.00 A2 N

ATOM 845 HN ALA A 56 15.098 2.492 0.821 0.00 0.00 A2 H

ATOM 846 CA ALA A 56 16.898 1.899 1.691 1.00 0.00 A2 C

ATOM 847 HA ALA A 56 17.599 1.306 1.115 0.00 0.00 A2 H

ATOM 848 CB ALA A 56 17.339 3.363 1.516 1.00 0.00 A2 C

ATOM 849 HB1 ALA A 56 17.244 3.656 0.449 0.00 0.00 A2 H

ATOM 850 HB2 ALA A 56 16.700 4.047 2.116 0.00 0.00 A2 H

ATOM 851 HB3 ALA A 56 18.398 3.511 1.820 0.00 0.00 A2 H

ATOM 852 C ALA A 56 17.106 1.515 3.147 1.00 0.00 A2 C

ATOM 853 O ALA A 56 18.208 1.274 3.615 1.00 0.00 A2 O

ATOM 854 N VAL A 57 15.997 1.456 3.931 1.00 0.00 A2 N

ATOM 855 HN VAL A 57 15.079 1.678 3.612 0.00 0.00 A2 H

ATOM 856 CA VAL A 57 16.068 1.053 5.334 1.00 0.00 A2 C

ATOM 857 HA VAL A 57 16.736 1.744 5.833 0.00 0.00 A2 H

ATOM 858 CB VAL A 57 14.712 1.273 6.021 1.00 0.00 A2 C

ATOM 859 HB VAL A 57 13.946 0.692 5.451 0.00 0.00 A2 H

ATOM 860 CG1 VAL A 57 14.699 0.788 7.464 1.00 0.00 A2 C

ATOM 861 1HG1 VAL A 57 13.715 1.001 7.934 0.00 0.00 A2 H

ATOM 862 2HG1 VAL A 57 14.866 -0.307 7.530 0.00 0.00 A2 H

ATOM 863 3HG1 VAL A 57 15.483 1.304 8.059 0.00 0.00 A2 H

ATOM 864 CG2 VAL A 57 14.285 2.687 5.957 1.00 0.00 A2 C

ATOM 865 1HG2 VAL A 57 13.323 2.832 6.493 0.00 0.00 A2 H

ATOM 866 2HG2 VAL A 57 15.048 3.347 6.424 0.00 0.00 A2 H

ATOM 867 3HG2 VAL A 57 14.136 3.015 4.907 0.00 0.00 A2 H

ATOM 868 C VAL A 57 16.670 -0.368 5.486 1.00 0.00 A2 C

ATOM 869 O VAL A 57 17.274 -0.639 6.519 1.00 0.00 A2 O

ATOM 870 CB CYS A 58 16.519 -3.383 3.323 1.00 0.00 A2 C

ATOM 871 SG CYS A 58 14.774 -3.692 3.407 1.00 0.00 A2 S

ATOM 872 N CYS A 58 16.498 -1.170 4.470 1.00 0.00 A2 N

ATOM 873 HN CYS A 58 15.996 -0.926 3.643 0.00 0.00 A2 H

ATOM 874 CA CYS A 58 17.053 -2.538 4.476 1.00 0.00 A2 C

ATOM 875 HA CYS A 58 16.663 -3.001 5.375 0.00 0.00 A2 H

ATOM 876 HB1 CYS A 58 16.733 -2.829 2.381 0.00 0.00 A2 H

ATOM 877 HB2 CYS A 58 17.091 -4.334 3.256 0.00 0.00 A2 H

ATOM 878 C CYS A 58 18.574 -2.584 4.554 1.00 0.00 A2 C

ATOM 879 O CYS A 58 19.142 -3.670 4.793 1.00 0.00 A2 O

ATOM 880 N SER A 59 19.187 -1.485 4.357 1.00 0.00 A2 N

ATOM 881 HN SER A 59 18.705 -0.640 4.125 0.00 0.00 A2 H

ATOM 882 CA SER A 59 20.672 -1.337 4.449 1.00 0.00 A2 C

ATOM 883 HA SER A 59 21.072 -2.316 4.681 0.00 0.00 A2 H

ATOM 884 CB SER A 59 21.210 -0.828 3.105 1.00 0.00 A2 C

ATOM 885 HB1 SER A 59 22.321 -0.901 3.127 0.00 0.00 A2 H

ATOM 886 HB2 SER A 59 20.856 -1.479 2.274 0.00 0.00 A2 H

ATOM 887 OG SER A 59 20.814 0.522 2.947 1.00 0.00 A2 O

ATOM 888 HG1 SER A 59 21.337 0.888 2.223 0.00 0.00 A2 H

ATOM 889 C SER A 59 21.017 -0.416 5.620 1.00 0.00 A2 C

ATOM 890 O SER A 59 22.206 0.070 5.675 1.00 0.00 A2 O

ATOM 891 N GLN A 60 20.174 -0.197 6.565 1.00 0.00 A2 N

ATOM 892 HN GLN A 60 19.294 -0.668 6.554 0.00 0.00 A2 H

ATOM 893 CA GLN A 60 20.365 0.703 7.705 1.00 0.00 A2 C

ATOM 894 HA GLN A 60 21.242 1.298 7.481 0.00 0.00 A2 H

ATOM 895 CB GLN A 60 19.346 1.810 7.715 1.00 0.00 A2 C

ATOM 896 HB1 GLN A 60 18.344 1.325 7.764 0.00 0.00 A2 H

ATOM 897 HB2 GLN A 60 19.451 2.400 8.654 0.00 0.00 A2 H

ATOM 898 CG GLN A 60 19.495 2.650 6.465 1.00 0.00 A2 C

ATOM 899 HG1 GLN A 60 20.496 3.131 6.439 0.00 0.00 A2 H

ATOM 900 HG2 GLN A 60 19.395 2.022 5.556 0.00 0.00 A2 H

ATOM 901 CD GLN A 60 18.498 3.772 6.371 1.00 0.00 A2 C

ATOM 902 OE1 GLN A 60 17.818 4.175 7.289 1.00 0.00 A2 O

ATOM 903 NE2 GLN A 60 18.379 4.255 5.163 1.00 0.00 A2 N

ATOM 904 1HE2 GLN A 60 17.735 5.005 5.040 0.00 0.00 A2 H

ATOM 905 2HE2 GLN A 60 18.907 3.861 4.419 0.00 0.00 A2 H

ATOM 906 C GLN A 60 20.640 -0.051 9.008 1.00 0.00 A2 C

ATOM 907 O GLN A 60 21.398 -1.084 8.907 1.00 0.00 A2 O

ATOM 908 N LYS A 61 20.159 0.309 10.116 1.00 0.00 A2 N

ATOM 909 HN LYS A 61 19.505 1.061 10.184 0.00 0.00 A2 H

ATOM 910 CA LYS A 61 20.513 -0.340 11.409 1.00 0.00 A2 C

ATOM 911 HA LYS A 61 21.580 -0.520 11.367 0.00 0.00 A2 H

ATOM 912 CB LYS A 61 20.183 0.634 12.533 1.00 0.00 A2 C

ATOM 913 HB1 LYS A 61 20.612 1.622 12.247 0.00 0.00 A2 H

ATOM 914 HB2 LYS A 61 19.080 0.773 12.579 0.00 0.00 A2 H

ATOM 915 CG LYS A 61 20.710 0.278 13.923 1.00 0.00 A2 C

ATOM 916 HG1 LYS A 61 20.176 -0.626 14.295 0.00 0.00 A2 H

ATOM 917 HG2 LYS A 61 21.790 0.017 13.845 0.00 0.00 A2 H

ATOM 918 CD LYS A 61 20.530 1.389 14.947 1.00 0.00 A2 C

ATOM 919 HD1 LYS A 61 20.574 2.367 14.416 0.00 0.00 A2 H

ATOM 920 HD2 LYS A 61 19.510 1.293 15.387 0.00 0.00 A2 H

ATOM 921 CE LYS A 61 21.574 1.349 16.056 1.00 0.00 A2 C

ATOM 922 HE1 LYS A 61 21.338 0.541 16.784 0.00 0.00 A2 H

ATOM 923 HE2 LYS A 61 22.585 1.158 15.634 0.00 0.00 A2 H

ATOM 924 NZ LYS A 61 21.687 2.599 16.850 1.00 0.00 A2 N

ATOM 925 HZ1 LYS A 61 22.417 2.490 17.584 0.00 0.00 A2 H

ATOM 926 HZ2 LYS A 61 21.944 3.387 16.222 0.00 0.00 A2 H

ATOM 927 HZ3 LYS A 61 20.773 2.806 17.301 0.00 0.00 A2 H

ATOM 928 C LYS A 61 19.844 -1.663 11.628 1.00 0.00 A2 C

ATOM 929 O LYS A 61 18.642 -1.661 11.830 1.00 0.00 A2 O

ATOM 930 N ASN A 62 20.596 -2.719 11.670 1.00 0.00 A2 N

ATOM 931 HN ASN A 62 21.584 -2.707 11.518 0.00 0.00 A2 H

ATOM 932 CA ASN A 62 20.039 -4.048 11.951 1.00 0.00 A2 C

ATOM 933 HA ASN A 62 19.140 -4.151 11.352 0.00 0.00 A2 H

ATOM 934 CB ASN A 62 20.989 -5.167 11.486 1.00 0.00 A2 C

ATOM 935 HB1 ASN A 62 21.102 -5.081 10.383 0.00 0.00 A2 H

ATOM 936 HB2 ASN A 62 21.994 -5.031 11.935 0.00 0.00 A2 H

ATOM 937 CG ASN A 62 20.461 -6.571 11.796 1.00 0.00 A2 C

ATOM 938 OD1 ASN A 62 21.049 -7.334 12.598 1.00 0.00 A2 O

ATOM 939 ND2 ASN A 62 19.309 -6.964 11.236 1.00 0.00 A2 N

ATOM 940 1HD2 ASN A 62 18.979 -7.877 11.461 0.00 0.00 A2 H

ATOM 941 2HD2 ASN A 62 18.804 -6.342 10.646 0.00 0.00 A2 H

ATOM 942 C ASN A 62 19.632 -4.093 13.390 1.00 0.00 A2 C

ATOM 943 O ASN A 62 20.367 -3.684 14.338 1.00 0.00 A2 O

ATOM 944 N VAL A 63 18.395 -4.598 13.680 1.00 0.00 A2 N

ATOM 945 HN VAL A 63 17.837 -4.929 12.923 0.00 0.00 A2 H

ATOM 946 CA VAL A 63 17.776 -4.718 14.969 1.00 0.00 A2 C

ATOM 947 HA VAL A 63 18.553 -4.887 15.704 0.00 0.00 A2 H

ATOM 948 CB VAL A 63 17.048 -3.449 15.424 1.00 0.00 A2 C

ATOM 949 HB VAL A 63 16.693 -3.620 16.470 0.00 0.00 A2 H

ATOM 950 CG1 VAL A 63 17.948 -2.225 15.423 1.00 0.00 A2 C

ATOM 951 1HG1 VAL A 63 17.372 -1.323 15.721 0.00 0.00 A2 H

ATOM 952 2HG1 VAL A 63 18.787 -2.333 16.141 0.00 0.00 A2 H

ATOM 953 3HG1 VAL A 63 18.367 -2.046 14.410 0.00 0.00 A2 H

ATOM 954 CG2 VAL A 63 15.809 -3.100 14.644 1.00 0.00 A2 C

ATOM 955 1HG2 VAL A 63 15.368 -2.149 15.012 0.00 0.00 A2 H

ATOM 956 2HG2 VAL A 63 16.048 -2.980 13.565 0.00 0.00 A2 H

ATOM 957 3HG2 VAL A 63 15.035 -3.889 14.744 0.00 0.00 A2 H

ATOM 958 C VAL A 63 16.848 -5.962 14.977 1.00 0.00 A2 C

ATOM 959 O VAL A 63 16.406 -6.471 13.951 1.00 0.00 A2 O

ATOM 960 N ALA A 64 16.664 -6.482 16.172 1.00 0.00 A2 N

ATOM 961 HN ALA A 64 17.095 -6.163 17.016 0.00 0.00 A2 H

ATOM 962 CA ALA A 64 15.750 -7.638 16.322 1.00 0.00 A2 C

ATOM 963 HA ALA A 64 15.887 -8.386 15.549 0.00 0.00 A2 H

ATOM 964 CB ALA A 64 15.961 -8.415 17.600 1.00 0.00 A2 C

ATOM 965 HB1 ALA A 64 16.996 -8.818 17.624 0.00 0.00 A2 H

ATOM 966 HB2 ALA A 64 15.827 -7.763 18.491 0.00 0.00 A2 H

ATOM 967 HB3 ALA A 64 15.257 -9.271 17.679 0.00 0.00 A2 H

ATOM 968 C ALA A 64 14.330 -7.086 16.144 1.00 0.00 A2 C

ATOM 969 O ALA A 64 13.942 -5.977 16.615 1.00 0.00 A2 O

ATOM 970 CB CYS A 65 11.427 -8.481 14.252 1.00 0.00 A2 C

ATOM 971 SG CYS A 65 12.263 -8.514 12.636 1.00 0.00 A2 S

ATOM 972 N CYS A 65 13.503 -7.812 15.412 1.00 0.00 A2 N

ATOM 973 HN CYS A 65 13.824 -8.614 14.912 0.00 0.00 A2 H

ATOM 974 CA CYS A 65 12.068 -7.525 15.261 1.00 0.00 A2 C

ATOM 975 HA CYS A 65 11.946 -6.468 15.056 0.00 0.00 A2 H

ATOM 976 HB1 CYS A 65 11.471 -9.503 14.692 0.00 0.00 A2 H

ATOM 977 HB2 CYS A 65 10.346 -8.242 14.140 0.00 0.00 A2 H

ATOM 978 C CYS A 65 11.409 -7.819 16.648 1.00 0.00 A2 C

ATOM 979 O CYS A 65 11.985 -8.518 17.494 1.00 0.00 A2 O

ATOM 980 N LYS A 66 10.180 -7.343 16.815 1.00 0.00 A2 N

ATOM 981 HN LYS A 66 9.695 -6.826 16.111 0.00 0.00 A2 H

ATOM 982 CA LYS A 66 9.445 -7.542 18.047 1.00 0.00 A2 C

ATOM 983 HA LYS A 66 10.018 -7.074 18.839 0.00 0.00 A2 H

ATOM 984 CB LYS A 66 8.009 -7.056 18.062 1.00 0.00 A2 C

ATOM 985 HB1 LYS A 66 7.509 -7.484 17.163 0.00 0.00 A2 H

ATOM 986 HB2 LYS A 66 7.489 -7.490 18.945 0.00 0.00 A2 H

ATOM 987 CG LYS A 66 7.896 -5.548 18.039 1.00 0.00 A2 C

ATOM 988 HG1 LYS A 66 8.306 -5.137 18.990 0.00 0.00 A2 H

ATOM 989 HG2 LYS A 66 8.522 -5.150 17.208 0.00 0.00 A2 H

ATOM 990 CD LYS A 66 6.357 -5.193 17.872 1.00 0.00 A2 C

ATOM 991 HD1 LYS A 66 6.030 -5.514 16.857 0.00 0.00 A2 H

ATOM 992 HD2 LYS A 66 5.780 -5.793 18.614 0.00 0.00 A2 H

ATOM 993 CE LYS A 66 6.124 -3.727 18.077 1.00 0.00 A2 C

ATOM 994 HE1 LYS A 66 5.859 -3.519 19.137 0.00 0.00 A2 H

ATOM 995 HE2 LYS A 66 7.038 -3.147 17.823 0.00 0.00 A2 H

ATOM 996 NZ LYS A 66 4.983 -3.212 17.200 1.00 0.00 A2 N

ATOM 997 HZ1 LYS A 66 4.841 -2.194 17.362 0.00 0.00 A2 H

ATOM 998 HZ2 LYS A 66 5.214 -3.374 16.199 0.00 0.00 A2 H

ATOM 999 HZ3 LYS A 66 4.108 -3.724 17.435 0.00 0.00 A2 H

ATOM 1000 C LYS A 66 9.324 -9.039 18.390 1.00 0.00 A2 C

ATOM 1001 O LYS A 66 9.360 -9.346 19.574 1.00 0.00 A2 O

ATOM 1002 N ASN A 67 9.192 -9.899 17.355 1.00 0.00 A2 N

ATOM 1003 HN ASN A 67 9.185 -9.616 16.397 0.00 0.00 A2 H

ATOM 1004 CA ASN A 67 9.046 -11.348 17.564 1.00 0.00 A2 C

ATOM 1005 HA ASN A 67 8.512 -11.431 18.505 0.00 0.00 A2 H

ATOM 1006 CB ASN A 67 8.215 -11.957 16.432 1.00 0.00 A2 C

ATOM 1007 HB1 ASN A 67 7.849 -12.950 16.774 0.00 0.00 A2 H

ATOM 1008 HB2 ASN A 67 7.325 -11.328 16.225 0.00 0.00 A2 H

ATOM 1009 CG ASN A 67 9.000 -12.180 15.130 1.00 0.00 A2 C

ATOM 1010 OD1 ASN A 67 10.063 -11.598 14.805 1.00 0.00 A2 O

ATOM 1011 ND2 ASN A 67 8.515 -13.170 14.343 1.00 0.00 A2 N

ATOM 1012 1HD2 ASN A 67 9.000 -13.358 13.493 0.00 0.00 A2 H

ATOM 1013 2HD2 ASN A 67 7.723 -13.697 14.636 0.00 0.00 A2 H

ATOM 1014 C ASN A 67 10.368 -12.106 17.712 1.00 0.00 A2 C

ATOM 1015 O ASN A 67 10.393 -13.375 17.843 1.00 0.00 A2 O

ATOM 1016 N GLY A 68 11.479 -11.373 17.671 1.00 0.00 A2 N

ATOM 1017 HN GLY A 68 11.485 -10.384 17.527 0.00 0.00 A2 H

ATOM 1018 CA GLY A 68 12.777 -11.988 17.838 1.00 0.00 A2 C

ATOM 1019 HA1 GLY A 68 12.581 -12.941 18.310 0.00 0.00 A2 H

ATOM 1020 HA2 GLY A 68 13.346 -11.299 18.448 0.00 0.00 A2 H

ATOM 1021 C GLY A 68 13.551 -12.242 16.588 1.00 0.00 A2 C

ATOM 1022 O GLY A 68 14.774 -12.481 16.660 1.00 0.00 A2 O

ATOM 1023 N GLN A 69 12.902 -12.181 15.437 1.00 0.00 A2 N

ATOM 1024 HN GLN A 69 11.930 -11.999 15.300 0.00 0.00 A2 H

ATOM 1025 CA GLN A 69 13.676 -12.404 14.171 1.00 0.00 A2 C

ATOM 1026 HA GLN A 69 14.173 -13.362 14.258 0.00 0.00 A2 H

ATOM 1027 CB GLN A 69 12.759 -12.284 12.956 1.00 0.00 A2 C

ATOM 1028 HB1 GLN A 69 12.176 -11.343 13.084 0.00 0.00 A2 H

ATOM 1029 HB2 GLN A 69 13.377 -12.144 12.040 0.00 0.00 A2 H

ATOM 1030 CG GLN A 69 11.832 -13.413 12.792 1.00 0.00 A2 C

ATOM 1031 HG1 GLN A 69 12.392 -14.343 12.556 0.00 0.00 A2 H

ATOM 1032 HG2 GLN A 69 11.258 -13.589 13.725 0.00 0.00 A2 H

ATOM 1033 CD GLN A 69 10.850 -13.200 11.646 1.00 0.00 A2 C

ATOM 1034 OE1 GLN A 69 9.765 -13.793 11.794 1.00 0.00 A2 O

ATOM 1035 NE2 GLN A 69 11.066 -12.329 10.616 1.00 0.00 A2 N

ATOM 1036 1HE2 GLN A 69 10.354 -12.267 9.922 0.00 0.00 A2 H

ATOM 1037 2HE2 GLN A 69 11.912 -11.807 10.578 0.00 0.00 A2 H

ATOM 1038 C GLN A 69 14.773 -11.322 14.019 1.00 0.00 A2 C

ATOM 1039 O GLN A 69 14.595 -10.214 14.523 1.00 0.00 A2 O

ATOM 1040 N THR A 70 15.816 -11.636 13.311 1.00 0.00 A2 N

ATOM 1041 HN THR A 70 15.933 -12.505 12.832 0.00 0.00 A2 H

ATOM 1042 CA THR A 70 16.919 -10.724 13.159 1.00 0.00 A2 C

ATOM 1043 HA THR A 70 16.690 -9.930 13.857 0.00 0.00 A2 H

ATOM 1044 CB THR A 70 18.165 -11.444 13.696 1.00 0.00 A2 C

ATOM 1045 HB THR A 70 19.029 -10.769 13.482 0.00 0.00 A2 H

ATOM 1046 OG1 THR A 70 18.323 -12.621 12.967 1.00 0.00 A2 O

ATOM 1047 HG1 THR A 70 19.103 -13.049 13.337 0.00 0.00 A2 H

ATOM 1048 CG2 THR A 70 18.169 -11.752 15.174 1.00 0.00 A2 C

ATOM 1049 1HG2 THR A 70 19.150 -12.167 15.489 0.00 0.00 A2 H

ATOM 1050 2HG2 THR A 70 17.984 -10.828 15.763 0.00 0.00 A2 H

ATOM 1051 3HG2 THR A 70 17.380 -12.492 15.431 0.00 0.00 A2 H

ATOM 1052 C THR A 70 17.072 -10.081 11.819 1.00 0.00 A2 C

ATOM 1053 O THR A 70 18.127 -9.492 11.523 1.00 0.00 A2 O

ATOM 1054 N ASN A 71 16.026 -10.115 11.003 1.00 0.00 A2 N

ATOM 1055 HN ASN A 71 15.186 -10.598 11.246 0.00 0.00 A2 H

ATOM 1056 CA ASN A 71 15.994 -9.463 9.690 1.00 0.00 A2 C

ATOM 1057 HA ASN A 71 17.034 -9.274 9.447 0.00 0.00 A2 H

ATOM 1058 CB ASN A 71 15.515 -10.415 8.593 1.00 0.00 A2 C

ATOM 1059 HB1 ASN A 71 15.614 -9.889 7.618 0.00 0.00 A2 H

ATOM 1060 HB2 ASN A 71 16.163 -11.314 8.553 0.00 0.00 A2 H

ATOM 1061 CG ASN A 71 14.081 -10.820 8.754 1.00 0.00 A2 C

ATOM 1062 OD1 ASN A 71 13.610 -11.048 9.877 1.00 0.00 A2 O

ATOM 1063 ND2 ASN A 71 13.355 -10.873 7.634 1.00 0.00 A2 N

ATOM 1064 1HD2 ASN A 71 12.396 -11.129 7.722 0.00 0.00 A2 H

ATOM 1065 2HD2 ASN A 71 13.767 -10.632 6.761 0.00 0.00 A2 H

ATOM 1066 C ASN A 71 15.262 -8.104 9.700 1.00 0.00 A2 C

ATOM 1067 O ASN A 71 14.782 -7.725 8.653 1.00 0.00 A2 O

ATOM 1068 SG CYS A 72 12.053 -6.660 12.004 1.00 0.00 A2 S

ATOM 1069 CB CYS A 72 13.792 -6.007 12.156 1.00 0.00 A2 C

ATOM 1070 N CYS A 72 15.233 -7.460 10.870 1.00 0.00 A2 N

ATOM 1071 HN CYS A 72 15.614 -7.802 11.727 0.00 0.00 A2 H

ATOM 1072 CA CYS A 72 14.613 -6.162 10.960 1.00 0.00 A2 C

ATOM 1073 HA CYS A 72 13.979 -6.036 10.090 0.00 0.00 A2 H

ATOM 1074 HB1 CYS A 72 14.296 -6.565 12.977 0.00 0.00 A2 H

ATOM 1075 HB2 CYS A 72 13.782 -4.940 12.471 0.00 0.00 A2 H

ATOM 1076 C CYS A 72 15.747 -5.066 10.890 1.00 0.00 A2 C

ATOM 1077 O CYS A 72 16.917 -5.352 11.209 1.00 0.00 A2 O

ATOM 1078 N TYR A 73 15.267 -3.927 10.437 1.00 0.00 A2 N

ATOM 1079 HN TYR A 73 14.305 -3.795 10.205 0.00 0.00 A2 H

ATOM 1080 CA TYR A 73 16.112 -2.744 10.233 1.00 0.00 A2 C

ATOM 1081 HA TYR A 73 16.968 -2.852 10.888 0.00 0.00 A2 H

ATOM 1082 CB TYR A 73 16.588 -2.575 8.776 1.00 0.00 A2 C

ATOM 1083 HB1 TYR A 73 15.702 -2.533 8.106 0.00 0.00 A2 H

ATOM 1084 HB2 TYR A 73 17.140 -1.618 8.652 0.00 0.00 A2 H

ATOM 1085 CG TYR A 73 17.438 -3.746 8.359 1.00 0.00 A2 C

ATOM 1086 CD1 TYR A 73 18.833 -3.674 8.464 1.00 0.00 A2 C

ATOM 1087 HD1 TYR A 73 19.301 -2.754 8.785 0.00 0.00 A2 H

ATOM 1088 CE1 TYR A 73 19.591 -4.810 8.122 1.00 0.00 A2 C

ATOM 1089 HE1 TYR A 73 20.670 -4.779 8.149 0.00 0.00 A2 H

ATOM 1090 CZ TYR A 73 18.961 -5.972 7.742 1.00 0.00 A2 C

ATOM 1091 OH TYR A 73 19.666 -7.123 7.365 1.00 0.00 A2 O

ATOM 1092 HH TYR A 73 19.022 -7.818 7.214 0.00 0.00 A2 H

ATOM 1093 CD2 TYR A 73 16.847 -4.913 7.897 1.00 0.00 A2 C

ATOM 1094 HD2 TYR A 73 15.781 -4.944 7.716 0.00 0.00 A2 H

ATOM 1095 CE2 TYR A 73 17.583 -6.055 7.604 1.00 0.00 A2 C

ATOM 1096 HE2 TYR A 73 17.077 -6.946 7.265 0.00 0.00 A2 H

ATOM 1097 C TYR A 73 15.379 -1.494 10.660 1.00 0.00 A2 C

ATOM 1098 O TYR A 73 14.208 -1.330 10.313 1.00 0.00 A2 O

ATOM 1099 N GLN A 74 16.082 -0.625 11.359 1.00 0.00 A2 N

ATOM 1100 HN GLN A 74 17.029 -0.815 11.610 0.00 0.00 A2 H

ATOM 1101 CA GLN A 74 15.568 0.693 11.839 1.00 0.00 A2 C

ATOM 1102 HA GLN A 74 14.495 0.694 11.692 0.00 0.00 A2 H

ATOM 1103 CB GLN A 74 15.897 0.941 13.289 1.00 0.00 A2 C

ATOM 1104 HB1 GLN A 74 15.478 0.084 13.866 0.00 0.00 A2 H

ATOM 1105 HB2 GLN A 74 17.001 0.899 13.429 0.00 0.00 A2 H

ATOM 1106 CG GLN A 74 15.341 2.233 13.844 1.00 0.00 A2 C

ATOM 1107 HG1 GLN A 74 15.719 3.100 13.260 0.00 0.00 A2 H

ATOM 1108 HG2 GLN A 74 14.233 2.241 13.787 0.00 0.00 A2 H

ATOM 1109 CD GLN A 74 15.798 2.396 15.273 1.00 0.00 A2 C

ATOM 1110 OE1 GLN A 74 15.603 1.532 16.085 1.00 0.00 A2 O

ATOM 1111 NE2 GLN A 74 16.484 3.472 15.502 1.00 0.00 A2 N

ATOM 1112 1HE2 GLN A 74 16.806 3.610 16.434 0.00 0.00 A2 H

ATOM 1113 2HE2 GLN A 74 16.650 4.119 14.765 0.00 0.00 A2 H

ATOM 1114 C GLN A 74 16.146 1.823 11.002 1.00 0.00 A2 C

ATOM 1115 O GLN A 74 17.372 1.877 10.750 1.00 0.00 A2 O

ATOM 1116 N SER A 75 15.250 2.722 10.603 1.00 0.00 A2 N

ATOM 1117 HN SER A 75 14.271 2.747 10.803 0.00 0.00 A2 H

ATOM 1118 CA SER A 75 15.718 3.823 9.767 1.00 0.00 A2 C

ATOM 1119 HA SER A 75 16.347 3.394 8.998 0.00 0.00 A2 H

ATOM 1120 CB SER A 75 14.533 4.604 9.203 1.00 0.00 A2 C

ATOM 1121 HB1 SER A 75 14.932 5.451 8.600 0.00 0.00 A2 H

ATOM 1122 HB2 SER A 75 13.939 3.963 8.513 0.00 0.00 A2 H

ATOM 1123 OG SER A 75 13.722 5.126 10.251 1.00 0.00 A2 O

ATOM 1124 HG1 SER A 75 13.134 5.782 9.855 0.00 0.00 A2 H

ATOM 1125 C SER A 75 16.616 4.760 10.631 1.00 0.00 A2 C

ATOM 1126 O SER A 75 16.360 5.017 11.803 1.00 0.00 A2 O

ATOM 1127 N TYR A 76 17.618 5.283 9.928 1.00 0.00 A2 N

ATOM 1128 HN TYR A 76 17.855 5.081 8.979 0.00 0.00 A2 H

ATOM 1129 CA TYR A 76 18.506 6.280 10.588 1.00 0.00 A2 C

ATOM 1130 HA TYR A 76 18.782 5.865 11.550 0.00 0.00 A2 H

ATOM 1131 CB TYR A 76 19.767 6.500 9.750 1.00 0.00 A2 C

ATOM 1132 HB1 TYR A 76 19.473 6.723 8.701 0.00 0.00 A2 H

ATOM 1133 HB2 TYR A 76 20.339 7.376 10.124 0.00 0.00 A2 H

ATOM 1134 CG TYR A 76 20.733 5.347 9.645 1.00 0.00 A2 C

ATOM 1135 CD1 TYR A 76 21.118 4.636 10.772 1.00 0.00 A2 C

ATOM 1136 HD1 TYR A 76 20.741 4.924 11.744 0.00 0.00 A2 H

ATOM 1137 CE1 TYR A 76 21.996 3.569 10.706 1.00 0.00 A2 C

ATOM 1138 HE1 TYR A 76 22.241 3.003 11.593 0.00 0.00 A2 H

ATOM 1139 CZ TYR A 76 22.557 3.247 9.489 1.00 0.00 A2 C

ATOM 1140 OH TYR A 76 23.456 2.189 9.422 1.00 0.00 A2 O

ATOM 1141 HH TYR A 76 23.686 2.064 8.499 0.00 0.00 A2 H

ATOM 1142 CD2 TYR A 76 21.340 5.020 8.444 1.00 0.00 A2 C

ATOM 1143 HD2 TYR A 76 21.141 5.610 7.560 0.00 0.00 A2 H

ATOM 1144 CE2 TYR A 76 22.255 3.964 8.348 1.00 0.00 A2 C

ATOM 1145 HE2 TYR A 76 22.721 3.743 7.399 0.00 0.00 A2 H

ATOM 1146 C TYR A 76 17.748 7.577 10.858 1.00 0.00 A2 C

ATOM 1147 O TYR A 76 18.047 8.194 11.897 1.00 0.00 A2 O

ATOM 1148 N SER A 77 16.885 7.972 9.995 1.00 0.00 A2 N

ATOM 1149 HN SER A 77 16.710 7.435 9.170 0.00 0.00 A2 H

ATOM 1150 CA SER A 77 16.089 9.188 10.095 1.00 0.00 A2 C

ATOM 1151 HA SER A 77 16.536 9.769 10.891 0.00 0.00 A2 H

ATOM 1152 CB SER A 77 16.179 9.962 8.790 1.00 0.00 A2 C

ATOM 1153 HB1 SER A 77 15.979 9.254 7.953 0.00 0.00 A2 H

ATOM 1154 HB2 SER A 77 15.391 10.748 8.750 0.00 0.00 A2 H

ATOM 1155 OG SER A 77 17.541 10.483 8.728 1.00 0.00 A2 O

ATOM 1156 HG1 SER A 77 17.689 10.781 7.822 0.00 0.00 A2 H

ATOM 1157 C SER A 77 14.629 8.882 10.512 1.00 0.00 A2 C

ATOM 1158 O SER A 77 14.151 7.815 10.279 1.00 0.00 A2 O

ATOM 1159 N THR A 78 14.012 9.918 11.012 1.00 0.00 A2 N

ATOM 1160 HN THR A 78 14.446 10.795 11.218 0.00 0.00 A2 H

ATOM 1161 CA THR A 78 12.545 9.866 11.328 1.00 0.00 A2 C

ATOM 1162 HA THR A 78 12.280 8.867 11.647 0.00 0.00 A2 H

ATOM 1163 CB THR A 78 12.027 10.877 12.336 1.00 0.00 A2 C

ATOM 1164 HB THR A 78 10.923 10.715 12.399 0.00 0.00 A2 H

ATOM 1165 OG1 THR A 78 12.283 12.140 11.786 1.00 0.00 A2 O

ATOM 1166 HG1 THR A 78 11.962 12.773 12.437 0.00 0.00 A2 H

ATOM 1167 CG2 THR A 78 12.637 10.804 13.716 1.00 0.00 A2 C

ATOM 1168 1HG2 THR A 78 12.122 11.498 14.413 0.00 0.00 A2 H

ATOM 1169 2HG2 THR A 78 12.544 9.776 14.127 0.00 0.00 A2 H

ATOM 1170 3HG2 THR A 78 13.715 11.072 13.692 0.00 0.00 A2 H

ATOM 1171 C THR A 78 11.897 10.071 9.935 1.00 0.00 A2 C

ATOM 1172 O THR A 78 12.397 10.651 8.964 1.00 0.00 A2 O

ATOM 1173 N MET A 79 10.665 9.522 9.848 1.00 0.00 A2 N

ATOM 1174 HN MET A 79 10.241 9.034 10.608 0.00 0.00 A2 H

ATOM 1175 CA MET A 79 9.848 9.590 8.648 1.00 0.00 A2 C

ATOM 1176 HA MET A 79 10.303 10.322 7.992 0.00 0.00 A2 H

ATOM 1177 CB MET A 79 9.766 8.165 7.989 1.00 0.00 A2 C

ATOM 1178 HB1 MET A 79 9.437 7.458 8.786 0.00 0.00 A2 H

ATOM 1179 HB2 MET A 79 8.963 8.160 7.218 0.00 0.00 A2 H

ATOM 1180 CG MET A 79 11.149 7.809 7.450 1.00 0.00 A2 C

ATOM 1181 HG1 MET A 79 11.544 8.637 6.824 0.00 0.00 A2 H

ATOM 1182 HG2 MET A 79 11.854 7.662 8.297 0.00 0.00 A2 H

ATOM 1183 SD MET A 79 11.039 6.304 6.456 1.00 0.00 A2 S

ATOM 1184 CE MET A 79 12.637 6.319 5.722 1.00 0.00 A2 C

ATOM 1185 HE1 MET A 79 12.770 5.450 5.043 0.00 0.00 A2 H

ATOM 1186 HE2 MET A 79 12.794 7.244 5.127 0.00 0.00 A2 H

ATOM 1187 HE3 MET A 79 13.433 6.271 6.496 0.00 0.00 A2 H

ATOM 1188 C MET A 79 8.458 10.069 8.969 1.00 0.00 A2 C

ATOM 1189 O MET A 79 8.053 9.856 10.113 1.00 0.00 A2 O

ATOM 1190 N SER A 80 7.784 10.673 8.028 1.00 0.00 A2 N

ATOM 1191 HN SER A 80 8.161 10.881 7.125 0.00 0.00 A2 H

ATOM 1192 CA SER A 80 6.394 11.106 8.214 1.00 0.00 A2 C

ATOM 1193 HA SER A 80 6.293 11.465 9.230 0.00 0.00 A2 H

ATOM 1194 CB SER A 80 6.050 12.093 7.112 1.00 0.00 A2 C

ATOM 1195 HB1 SER A 80 6.693 12.994 7.241 0.00 0.00 A2 H

ATOM 1196 HB2 SER A 80 6.290 11.660 6.115 0.00 0.00 A2 H

ATOM 1197 OG SER A 80 4.689 12.394 7.304 1.00 0.00 A2 O

ATOM 1198 HG1 SER A 80 4.494 13.176 6.772 0.00 0.00 A2 H

ATOM 1199 C SER A 80 5.490 9.835 8.077 1.00 0.00 A2 C

ATOM 1200 O SER A 80 5.552 9.152 7.040 1.00 0.00 A2 O

ATOM 1201 N ILE A 81 4.782 9.588 9.133 1.00 0.00 A2 N

ATOM 1202 HN ILE A 81 4.765 10.129 9.971 0.00 0.00 A2 H

ATOM 1203 CA ILE A 81 3.885 8.391 9.158 1.00 0.00 A2 C

ATOM 1204 HA ILE A 81 3.769 8.061 8.134 0.00 0.00 A2 H

ATOM 1205 CB ILE A 81 4.421 7.264 10.015 1.00 0.00 A2 C

ATOM 1206 HB ILE A 81 3.637 6.467 9.952 0.00 0.00 A2 H

ATOM 1207 CG2 ILE A 81 5.771 6.714 9.461 1.00 0.00 A2 C

ATOM 1208 1HG2 ILE A 81 6.070 5.793 10.004 0.00 0.00 A2 H

ATOM 1209 2HG2 ILE A 81 5.682 6.456 8.385 0.00 0.00 A2 H

ATOM 1210 3HG2 ILE A 81 6.583 7.463 9.576 0.00 0.00 A2 H

ATOM 1211 CG1 ILE A 81 4.520 7.627 11.478 1.00 0.00 A2 C

ATOM 1212 1HG1 ILE A 81 5.328 8.378 11.626 0.00 0.00 A2 H

ATOM 1213 2HG1 ILE A 81 3.565 8.104 11.797 0.00 0.00 A2 H

ATOM 1214 CD ILE A 81 4.783 6.411 12.384 1.00 0.00 A2 C

ATOM 1215 HD1 ILE A 81 4.790 6.720 13.452 0.00 0.00 A2 H

ATOM 1216 HD2 ILE A 81 3.991 5.643 12.249 0.00 0.00 A2 H

ATOM 1217 HD3 ILE A 81 5.766 5.948 12.154 0.00 0.00 A2 H

ATOM 1218 C ILE A 81 2.494 8.799 9.652 1.00 0.00 A2 C

ATOM 1219 O ILE A 81 2.247 9.805 10.341 1.00 0.00 A2 O

ATOM 1220 N THR A 82 1.524 7.932 9.293 1.00 0.00 A2 N

ATOM 1221 HN THR A 82 1.685 7.154 8.687 0.00 0.00 A2 H

ATOM 1222 CA THR A 82 0.126 8.055 9.767 1.00 0.00 A2 C

ATOM 1223 HA THR A 82 0.099 8.821 10.531 0.00 0.00 A2 H

ATOM 1224 CB THR A 82 -0.898 8.357 8.718 1.00 0.00 A2 C

ATOM 1225 HB THR A 82 -0.787 7.564 7.939 0.00 0.00 A2 H

ATOM 1226 OG1 THR A 82 -0.581 9.642 8.114 1.00 0.00 A2 O

ATOM 1227 HG1 THR A 82 -1.271 9.795 7.459 0.00 0.00 A2 H

ATOM 1228 CG2 THR A 82 -2.293 8.464 9.254 1.00 0.00 A2 C

ATOM 1229 1HG2 THR A 82 -3.026 8.575 8.428 0.00 0.00 A2 H

ATOM 1230 2HG2 THR A 82 -2.559 7.550 9.827 0.00 0.00 A2 H

ATOM 1231 3HG2 THR A 82 -2.396 9.340 9.930 0.00 0.00 A2 H

ATOM 1232 C THR A 82 -0.215 6.704 10.479 1.00 0.00 A2 C

ATOM 1233 O THR A 82 -0.073 5.659 9.818 1.00 0.00 A2 O

ATOM 1234 N ASP A 83 -0.658 6.791 11.681 1.00 0.00 A2 N

ATOM 1235 HN ASP A 83 -0.731 7.635 12.206 0.00 0.00 A2 H

ATOM 1236 CA ASP A 83 -1.111 5.628 12.417 1.00 0.00 A2 C

ATOM 1237 HA ASP A 83 -0.737 4.742 11.916 0.00 0.00 A2 H

ATOM 1238 CB ASP A 83 -0.687 5.725 13.892 1.00 0.00 A2 C

ATOM 1239 HB1 ASP A 83 -0.277 6.736 14.088 0.00 0.00 A2 H

ATOM 1240 HB2 ASP A 83 -1.557 5.566 14.560 0.00 0.00 A2 H

ATOM 1241 CG ASP A 83 0.379 4.699 14.169 1.00 0.00 A2 C

ATOM 1242 OD1 ASP A 83 1.381 4.520 13.534 1.00 0.00 A2 O

ATOM 1243 OD2 ASP A 83 0.028 3.950 15.183 1.00 0.00 A2 O

ATOM 1244 C ASP A 83 -2.637 5.570 12.336 1.00 0.00 A2 C

ATOM 1245 O ASP A 83 -3.295 6.596 12.531 1.00 0.00 A2 O

ATOM 1246 SG CYS A 84 -4.548 4.743 9.165 1.00 0.00 A2 S

ATOM 1247 CB CYS A 84 -4.907 3.579 10.511 1.00 0.00 A2 C

ATOM 1248 N CYS A 84 -3.176 4.392 12.004 1.00 0.00 A2 N

ATOM 1249 HN CYS A 84 -2.587 3.612 11.803 0.00 0.00 A2 H

ATOM 1250 CA CYS A 84 -4.564 4.128 11.905 1.00 0.00 A2 C

ATOM 1251 HA CYS A 84 -5.088 5.049 12.132 0.00 0.00 A2 H

ATOM 1252 HB1 CYS A 84 -4.296 2.661 10.358 0.00 0.00 A2 H

ATOM 1253 HB2 CYS A 84 -5.969 3.248 10.487 0.00 0.00 A2 H

ATOM 1254 C CYS A 84 -4.950 3.101 12.964 1.00 0.00 A2 C

ATOM 1255 O CYS A 84 -4.357 2.030 12.922 1.00 0.00 A2 O

ATOM 1256 N ARG A 85 -5.897 3.459 13.779 1.00 0.00 A2 N

ATOM 1257 HN ARG A 85 -6.358 4.342 13.730 0.00 0.00 A2 H

ATOM 1258 CA ARG A 85 -6.350 2.519 14.868 1.00 0.00 A2 C

ATOM 1259 HA ARG A 85 -5.994 1.519 14.650 0.00 0.00 A2 H

ATOM 1260 CB ARG A 85 -5.964 3.001 16.242 1.00 0.00 A2 C

ATOM 1261 HB1 ARG A 85 -5.033 3.603 16.115 0.00 0.00 A2 H

ATOM 1262 HB2 ARG A 85 -6.736 3.707 16.619 0.00 0.00 A2 H

ATOM 1263 CG ARG A 85 -5.681 1.936 17.287 1.00 0.00 A2 C

ATOM 1264 HG1 ARG A 85 -6.104 2.273 18.259 0.00 0.00 A2 H

ATOM 1265 HG2 ARG A 85 -6.241 1.010 17.021 0.00 0.00 A2 H

ATOM 1266 CD ARG A 85 -4.215 1.630 17.347 1.00 0.00 A2 C

ATOM 1267 HD1 ARG A 85 -4.031 0.850 18.121 0.00 0.00 A2 H

ATOM 1268 HD2 ARG A 85 -3.834 1.258 16.367 0.00 0.00 A2 H

ATOM 1269 NE ARG A 85 -3.451 2.826 17.674 1.00 0.00 A2 N

ATOM 1270 HE ARG A 85 -4.028 3.596 17.969 0.00 0.00 A2 H

ATOM 1271 CZ ARG A 85 -2.129 2.919 17.600 1.00 0.00 A2 C

ATOM 1272 NH1 ARG A 85 -1.338 1.908 17.254 1.00 0.00 A2 N

ATOM 1273 1HH1 ARG A 85 -0.354 2.020 17.238 0.00 0.00 A2 H

ATOM 1274 2HH1 ARG A 85 -1.780 1.014 17.150 0.00 0.00 A2 H

ATOM 1275 NH2 ARG A 85 -1.576 4.108 17.888 1.00 0.00 A2 N

ATOM 1276 1HH2 ARG A 85 -0.604 4.244 17.761 0.00 0.00 A2 H

ATOM 1277 2HH2 ARG A 85 -2.196 4.871 18.019 0.00 0.00 A2 H

ATOM 1278 C ARG A 85 -7.875 2.451 14.862 1.00 0.00 A2 C

ATOM 1279 O ARG A 85 -8.541 3.464 14.786 1.00 0.00 A2 O

ATOM 1280 N GLU A 86 -8.369 1.202 14.903 1.00 0.00 A2 N

ATOM 1281 HN GLU A 86 -7.833 0.363 14.940 0.00 0.00 A2 H

ATOM 1282 CA GLU A 86 -9.802 0.985 14.896 1.00 0.00 A2 C

ATOM 1283 HA GLU A 86 -10.214 1.464 14.016 0.00 0.00 A2 H

ATOM 1284 CB GLU A 86 -10.013 -0.520 14.862 1.00 0.00 A2 C

ATOM 1285 HB1 GLU A 86 -9.250 -0.962 14.181 0.00 0.00 A2 H

ATOM 1286 HB2 GLU A 86 -9.811 -0.952 15.868 0.00 0.00 A2 H

ATOM 1287 CG GLU A 86 -11.312 -0.934 14.419 1.00 0.00 A2 C

ATOM 1288 HG1 GLU A 86 -12.079 -0.548 15.114 0.00 0.00 A2 H

ATOM 1289 HG2 GLU A 86 -11.522 -0.557 13.398 0.00 0.00 A2 H

ATOM 1290 CD GLU A 86 -11.651 -2.451 14.359 1.00 0.00 A2 C

ATOM 1291 OE1 GLU A 86 -12.623 -2.705 13.782 1.00 0.00 A2 O

ATOM 1292 OE2 GLU A 86 -10.813 -3.167 14.937 1.00 0.00 A2 O

ATOM 1293 C GLU A 86 -10.466 1.641 16.128 1.00 0.00 A2 C

ATOM 1294 O GLU A 86 -9.927 1.627 17.235 1.00 0.00 A2 O

ATOM 1295 N THR A 87 -11.577 2.289 15.859 1.00 0.00 A2 N

ATOM 1296 HN THR A 87 -11.957 2.334 14.936 0.00 0.00 A2 H

ATOM 1297 CA THR A 87 -12.375 3.013 16.885 1.00 0.00 A2 C

ATOM 1298 HA THR A 87 -11.701 3.268 17.692 0.00 0.00 A2 H

ATOM 1299 CB THR A 87 -13.072 4.264 16.366 1.00 0.00 A2 C

ATOM 1300 HB THR A 87 -13.438 4.812 17.268 0.00 0.00 A2 H

ATOM 1301 OG1 THR A 87 -14.182 3.863 15.605 1.00 0.00 A2 O

ATOM 1302 HG1 THR A 87 -14.584 4.679 15.289 0.00 0.00 A2 H

ATOM 1303 CG2 THR A 87 -12.150 5.160 15.599 1.00 0.00 A2 C

ATOM 1304 1HG2 THR A 87 -12.655 6.112 15.333 0.00 0.00 A2 H

ATOM 1305 2HG2 THR A 87 -11.255 5.407 16.208 0.00 0.00 A2 H

ATOM 1306 3HG2 THR A 87 -11.808 4.673 14.660 0.00 0.00 A2 H

ATOM 1307 C THR A 87 -13.400 2.039 17.466 1.00 0.00 A2 C

ATOM 1308 O THR A 87 -13.727 1.002 16.891 1.00 0.00 A2 O

ATOM 1309 N GLY A 88 -13.968 2.513 18.609 1.00 0.00 A2 N

ATOM 1310 HN GLY A 88 -13.743 3.394 19.024 0.00 0.00 A2 H

ATOM 1311 CA GLY A 88 -14.976 1.722 19.312 1.00 0.00 A2 C

ATOM 1312 HA1 GLY A 88 -15.254 2.277 20.198 0.00 0.00 A2 H

ATOM 1313 HA2 GLY A 88 -14.542 0.752 19.513 0.00 0.00 A2 H

ATOM 1314 C GLY A 88 -16.195 1.516 18.500 1.00 0.00 A2 C

ATOM 1315 O GLY A 88 -16.930 0.526 18.772 1.00 0.00 A2 O

ATOM 1316 N SER A 89 -16.516 2.375 17.532 1.00 0.00 A2 N

ATOM 1317 HN SER A 89 -15.933 3.167 17.353 0.00 0.00 A2 H

ATOM 1318 CA SER A 89 -17.651 2.285 16.684 1.00 0.00 A2 C

ATOM 1319 HA SER A 89 -18.389 1.779 17.292 0.00 0.00 A2 H

ATOM 1320 CB SER A 89 -18.176 3.638 16.301 1.00 0.00 A2 C

ATOM 1321 HB1 SER A 89 -17.320 4.251 15.936 0.00 0.00 A2 H

ATOM 1322 HB2 SER A 89 -18.898 3.549 15.458 0.00 0.00 A2 H

ATOM 1323 OG SER A 89 -18.731 4.175 17.495 1.00 0.00 A2 O

ATOM 1324 HG1 SER A 89 -18.873 5.118 17.343 0.00 0.00 A2 H

ATOM 1325 C SER A 89 -17.471 1.451 15.387 1.00 0.00 A2 C

ATOM 1326 O SER A 89 -18.409 1.258 14.626 1.00 0.00 A2 O

ATOM 1327 N SER A 90 -16.294 0.975 15.192 1.00 0.00 A2 N

ATOM 1328 HN SER A 90 -15.530 1.110 15.822 0.00 0.00 A2 H

ATOM 1329 CA SER A 90 -15.954 0.159 13.980 1.00 0.00 A2 C

ATOM 1330 HA SER A 90 -16.280 0.722 13.115 0.00 0.00 A2 H

ATOM 1331 CB SER A 90 -14.518 -0.097 14.041 1.00 0.00 A2 C

ATOM 1332 HB1 SER A 90 -13.997 0.880 14.166 0.00 0.00 A2 H

ATOM 1333 HB2 SER A 90 -14.272 -0.716 14.934 0.00 0.00 A2 H

ATOM 1334 OG SER A 90 -14.109 -0.717 12.811 1.00 0.00 A2 O

ATOM 1335 HG1 SER A 90 -13.145 -0.673 12.778 0.00 0.00 A2 H

ATOM 1336 C SER A 90 -16.781 -1.152 14.015 1.00 0.00 A2 C

ATOM 1337 O SER A 90 -16.715 -1.813 15.049 1.00 0.00 A2 O

ATOM 1338 N LYS A 91 -17.404 -1.416 12.908 1.00 0.00 A2 N

ATOM 1339 HN LYS A 91 -17.398 -0.843 12.090 0.00 0.00 A2 H

ATOM 1340 CA LYS A 91 -18.198 -2.650 12.800 1.00 0.00 A2 C

ATOM 1341 HA LYS A 91 -17.674 -3.428 13.341 0.00 0.00 A2 H

ATOM 1342 CB LYS A 91 -19.584 -2.401 13.355 1.00 0.00 A2 C

ATOM 1343 HB1 LYS A 91 -19.467 -2.175 14.440 0.00 0.00 A2 H

ATOM 1344 HB2 LYS A 91 -20.002 -1.481 12.887 0.00 0.00 A2 H

ATOM 1345 CG LYS A 91 -20.575 -3.569 13.204 1.00 0.00 A2 C

ATOM 1346 HG1 LYS A 91 -21.041 -3.523 12.193 0.00 0.00 A2 H

ATOM 1347 HG2 LYS A 91 -20.015 -4.530 13.269 0.00 0.00 A2 H

ATOM 1348 CD LYS A 91 -21.669 -3.526 14.253 1.00 0.00 A2 C

ATOM 1349 HD1 LYS A 91 -21.191 -3.477 15.258 0.00 0.00 A2 H

ATOM 1350 HD2 LYS A 91 -22.248 -2.584 14.112 0.00 0.00 A2 H

ATOM 1351 CE LYS A 91 -22.600 -4.721 14.172 1.00 0.00 A2 C

ATOM 1352 HE1 LYS A 91 -22.017 -5.663 14.071 0.00 0.00 A2 H

ATOM 1353 HE2 LYS A 91 -23.226 -4.792 15.089 0.00 0.00 A2 H

ATOM 1354 NZ LYS A 91 -23.500 -4.610 13.007 1.00 0.00 A2 N

ATOM 1355 HZ1 LYS A 91 -24.131 -5.436 12.964 0.00 0.00 A2 H

ATOM 1356 HZ2 LYS A 91 -24.070 -3.744 13.090 0.00 0.00 A2 H

ATOM 1357 HZ3 LYS A 91 -22.933 -4.564 12.136 0.00 0.00 A2 H

ATOM 1358 C LYS A 91 -18.281 -3.090 11.329 1.00 0.00 A2 C

ATOM 1359 O LYS A 91 -18.858 -2.428 10.525 1.00 0.00 A2 O

ATOM 1360 N TYR A 92 -17.627 -4.192 11.101 1.00 0.00 A2 N

ATOM 1361 HN TYR A 92 -17.149 -4.733 11.791 0.00 0.00 A2 H

ATOM 1362 CA TYR A 92 -17.548 -4.744 9.715 1.00 0.00 A2 C

ATOM 1363 HA TYR A 92 -16.979 -4.032 9.130 0.00 0.00 A2 H

ATOM 1364 CB TYR A 92 -16.930 -6.150 9.787 1.00 0.00 A2 C

ATOM 1365 HB1 TYR A 92 -15.945 -6.089 10.300 0.00 0.00 A2 H

ATOM 1366 HB2 TYR A 92 -17.572 -6.831 10.387 0.00 0.00 A2 H

ATOM 1367 CG TYR A 92 -16.707 -6.698 8.422 1.00 0.00 A2 C

ATOM 1368 CD1 TYR A 92 -15.554 -6.380 7.667 1.00 0.00 A2 C

ATOM 1369 HD1 TYR A 92 -14.804 -5.721 8.083 0.00 0.00 A2 H

ATOM 1370 CE1 TYR A 92 -15.391 -6.886 6.400 1.00 0.00 A2 C

ATOM 1371 HE1 TYR A 92 -14.507 -6.654 5.825 0.00 0.00 A2 H

ATOM 1372 CZ TYR A 92 -16.400 -7.712 5.848 1.00 0.00 A2 C

ATOM 1373 OH TYR A 92 -16.249 -8.249 4.609 1.00 0.00 A2 O

ATOM 1374 HH TYR A 92 -16.999 -8.827 4.452 0.00 0.00 A2 H

ATOM 1375 CD2 TYR A 92 -17.686 -7.518 7.821 1.00 0.00 A2 C

ATOM 1376 HD2 TYR A 92 -18.606 -7.734 8.346 0.00 0.00 A2 H

ATOM 1377 CE2 TYR A 92 -17.513 -8.006 6.563 1.00 0.00 A2 C

ATOM 1378 HE2 TYR A 92 -18.281 -8.620 6.116 0.00 0.00 A2 H

ATOM 1379 C TYR A 92 -18.950 -4.840 9.078 1.00 0.00 A2 C

ATOM 1380 O TYR A 92 -19.803 -5.349 9.783 1.00 0.00 A2 O

ATOM 1381 N PRO A 93 -19.087 -4.419 7.831 1.00 0.00 A2 N

ATOM 1382 CD PRO A 93 -20.443 -4.575 7.119 1.00 0.00 A2 C

ATOM 1383 HD1 PRO A 93 -20.731 -5.650 7.160 0.00 0.00 A2 H

ATOM 1384 HD2 PRO A 93 -21.253 -3.938 7.543 0.00 0.00 A2 H

ATOM 1385 CA PRO A 93 -18.132 -3.924 6.902 1.00 0.00 A2 C

ATOM 1386 HA PRO A 93 -17.224 -4.506 7.007 0.00 0.00 A2 H

ATOM 1387 CB PRO A 93 -18.694 -4.338 5.502 1.00 0.00 A2 C

ATOM 1388 HB1 PRO A 93 -18.373 -5.379 5.276 0.00 0.00 A2 H

ATOM 1389 HB2 PRO A 93 -18.350 -3.670 4.687 0.00 0.00 A2 H

ATOM 1390 CG PRO A 93 -20.160 -4.168 5.687 1.00 0.00 A2 C

ATOM 1391 HG1 PRO A 93 -20.758 -4.778 4.982 0.00 0.00 A2 H

ATOM 1392 HG2 PRO A 93 -20.423 -3.092 5.562 0.00 0.00 A2 H

ATOM 1393 C PRO A 93 -17.794 -2.453 7.021 1.00 0.00 A2 C

ATOM 1394 O PRO A 93 -16.967 -1.988 6.237 1.00 0.00 A2 O

ATOM 1395 N ASN A 94 -18.408 -1.824 8.001 1.00 0.00 A2 N

ATOM 1396 HN ASN A 94 -19.045 -2.226 8.657 0.00 0.00 A2 H

ATOM 1397 CA ASN A 94 -18.159 -0.353 8.195 1.00 0.00 A2 C

ATOM 1398 HA ASN A 94 -17.815 0.018 7.235 0.00 0.00 A2 H

ATOM 1399 CB ASN A 94 -19.493 0.328 8.542 1.00 0.00 A2 C

ATOM 1400 HB1 ASN A 94 -19.899 -0.163 9.453 0.00 0.00 A2 H

ATOM 1401 HB2 ASN A 94 -19.328 1.397 8.786 0.00 0.00 A2 H

ATOM 1402 CG ASN A 94 -20.547 0.196 7.422 1.00 0.00 A2 C

ATOM 1403 OD1 ASN A 94 -21.703 -0.215 7.639 1.00 0.00 A2 O

ATOM 1404 ND2 ASN A 94 -20.156 0.623 6.230 1.00 0.00 A2 N

ATOM 1405 1HD2 ASN A 94 -20.814 0.564 5.484 0.00 0.00 A2 H

ATOM 1406 2HD2 ASN A 94 -19.247 1.012 6.113 0.00 0.00 A2 H

ATOM 1407 C ASN A 94 -17.039 -0.107 9.217 1.00 0.00 A2 C

ATOM 1408 O ASN A 94 -17.257 0.291 10.338 1.00 0.00 A2 O

ATOM 1409 SG CYS A 95 -13.809 -2.782 8.569 1.00 0.00 A2 S

ATOM 1410 CB CYS A 95 -13.479 -1.030 8.901 1.00 0.00 A2 C

ATOM 1411 N CYS A 95 -15.838 -0.416 8.722 1.00 0.00 A2 N

ATOM 1412 HN CYS A 95 -15.670 -0.733 7.791 0.00 0.00 A2 H

ATOM 1413 CA CYS A 95 -14.634 -0.301 9.561 1.00 0.00 A2 C

ATOM 1414 HA CYS A 95 -14.866 -0.710 10.538 0.00 0.00 A2 H

ATOM 1415 HB1 CYS A 95 -13.278 -0.521 7.931 0.00 0.00 A2 H

ATOM 1416 HB2 CYS A 95 -12.557 -0.905 9.510 0.00 0.00 A2 H

ATOM 1417 C CYS A 95 -14.330 1.182 9.739 1.00 0.00 A2 C

ATOM 1418 O CYS A 95 -14.403 1.926 8.748 1.00 0.00 A2 O

ATOM 1419 N ALA A 96 -14.004 1.527 10.957 1.00 0.00 A2 N

ATOM 1420 HN ALA A 96 -13.905 0.876 11.709 0.00 0.00 A2 H

ATOM 1421 CA ALA A 96 -13.756 2.937 11.304 1.00 0.00 A2 C

ATOM 1422 HA ALA A 96 -13.609 3.549 10.421 0.00 0.00 A2 H

ATOM 1423 CB ALA A 96 -14.928 3.491 12.126 1.00 0.00 A2 C

ATOM 1424 HB1 ALA A 96 -15.855 3.471 11.514 0.00 0.00 A2 H

ATOM 1425 HB2 ALA A 96 -15.105 2.875 13.034 0.00 0.00 A2 H

ATOM 1426 HB3 ALA A 96 -14.747 4.541 12.441 0.00 0.00 A2 H

ATOM 1427 C ALA A 96 -12.463 3.019 12.103 1.00 0.00 A2 C

ATOM 1428 O ALA A 96 -12.174 2.222 12.935 1.00 0.00 A2 O

ATOM 1429 N TYR A 97 -11.713 4.067 11.763 1.00 0.00 A2 N

ATOM 1430 HN TYR A 97 -12.024 4.737 11.091 0.00 0.00 A2 H

ATOM 1431 CA TYR A 97 -10.417 4.333 12.312 1.00 0.00 A2 C

ATOM 1432 HA TYR A 97 -10.369 3.691 13.183 0.00 0.00 A2 H

ATOM 1433 CB TYR A 97 -9.296 4.110 11.138 1.00 0.00 A2 C

ATOM 1434 HB1 TYR A 97 -9.539 4.765 10.273 0.00 0.00 A2 H

ATOM 1435 HB2 TYR A 97 -8.286 4.403 11.498 0.00 0.00 A2 H

ATOM 1436 CG TYR A 97 -9.349 2.627 10.689 1.00 0.00 A2 C

ATOM 1437 CD1 TYR A 97 -8.603 1.700 11.359 1.00 0.00 A2 C

ATOM 1438 HD1 TYR A 97 -7.912 2.020 12.127 0.00 0.00 A2 H

ATOM 1439 CE1 TYR A 97 -8.694 0.345 11.062 1.00 0.00 A2 C

ATOM 1440 HE1 TYR A 97 -8.097 -0.377 11.600 0.00 0.00 A2 H

ATOM 1441 CZ TYR A 97 -9.561 -0.051 10.057 1.00 0.00 A2 C

ATOM 1442 OH TYR A 97 -9.661 -1.366 9.695 1.00 0.00 A2 O

ATOM 1443 HH TYR A 97 -10.360 -1.432 9.041 0.00 0.00 A2 H

ATOM 1444 CD2 TYR A 97 -10.204 2.229 9.698 1.00 0.00 A2 C

ATOM 1445 HD2 TYR A 97 -10.781 2.964 9.155 0.00 0.00 A2 H

ATOM 1446 CE2 TYR A 97 -10.292 0.868 9.354 1.00 0.00 A2 C

ATOM 1447 HE2 TYR A 97 -10.917 0.563 8.528 0.00 0.00 A2 H

ATOM 1448 C TYR A 97 -10.136 5.772 12.792 1.00 0.00 A2 C

ATOM 1449 O TYR A 97 -10.704 6.719 12.180 1.00 0.00 A2 O

ATOM 1450 N LYS A 98 -9.296 5.829 13.762 1.00 0.00 A2 N

ATOM 1451 HN LYS A 98 -8.956 5.035 14.263 0.00 0.00 A2 H

ATOM 1452 CA LYS A 98 -8.761 7.129 14.211 1.00 0.00 A2 C

ATOM 1453 HA LYS A 98 -9.419 7.926 13.885 0.00 0.00 A2 H

ATOM 1454 CB LYS A 98 -8.451 7.056 15.687 1.00 0.00 A2 C

ATOM 1455 HB1 LYS A 98 -9.411 6.851 16.214 0.00 0.00 A2 H

ATOM 1456 HB2 LYS A 98 -7.796 6.176 15.877 0.00 0.00 A2 H

ATOM 1457 CG LYS A 98 -7.823 8.296 16.338 1.00 0.00 A2 C

ATOM 1458 HG1 LYS A 98 -7.405 8.014 17.331 0.00 0.00 A2 H

ATOM 1459 HG2 LYS A 98 -6.974 8.646 15.707 0.00 0.00 A2 H

ATOM 1460 CD LYS A 98 -8.857 9.372 16.505 1.00 0.00 A2 C

ATOM 1461 HD1 LYS A 98 -9.445 9.444 15.562 0.00 0.00 A2 H

ATOM 1462 HD2 LYS A 98 -9.558 9.057 17.312 0.00 0.00 A2 H

ATOM 1463 CE LYS A 98 -8.366 10.769 16.851 1.00 0.00 A2 C

ATOM 1464 HE1 LYS A 98 -7.993 10.803 17.899 0.00 0.00 A2 H

ATOM 1465 HE2 LYS A 98 -7.538 11.071 16.173 0.00 0.00 A2 H

ATOM 1466 NZ LYS A 98 -9.541 11.699 16.687 1.00 0.00 A2 N

ATOM 1467 HZ1 LYS A 98 -9.258 12.674 16.914 0.00 0.00 A2 H

ATOM 1468 HZ2 LYS A 98 -9.882 11.658 15.705 0.00 0.00 A2 H

ATOM 1469 HZ3 LYS A 98 -10.307 11.405 17.327 0.00 0.00 A2 H

ATOM 1470 C LYS A 98 -7.383 7.309 13.443 1.00 0.00 A2 C

ATOM 1471 O LYS A 98 -6.594 6.375 13.443 1.00 0.00 A2 O

ATOM 1472 N THR A 99 -7.225 8.510 12.924 1.00 0.00 A2 N

ATOM 1473 HN THR A 99 -7.922 9.223 12.989 0.00 0.00 A2 H

ATOM 1474 CA THR A 99 -6.025 8.911 12.210 1.00 0.00 A2 C

ATOM 1475 HA THR A 99 -5.486 8.002 11.980 0.00 0.00 A2 H

ATOM 1476 CB THR A 99 -6.476 9.816 11.029 1.00 0.00 A2 C

ATOM 1477 HB THR A 99 -7.131 10.604 11.475 0.00 0.00 A2 H

ATOM 1478 OG1 THR A 99 -7.246 9.048 10.145 1.00 0.00 A2 O

ATOM 1479 HG1 THR A 99 -7.490 9.646 9.430 0.00 0.00 A2 H

ATOM 1480 CG2 THR A 99 -5.298 10.374 10.239 1.00 0.00 A2 C

ATOM 1481 1HG2 THR A 99 -5.644 11.104 9.477 0.00 0.00 A2 H

ATOM 1482 2HG2 THR A 99 -4.590 10.898 10.917 0.00 0.00 A2 H

ATOM 1483 3HG2 THR A 99 -4.745 9.563 9.718 0.00 0.00 A2 H

ATOM 1484 C THR A 99 -5.103 9.748 13.155 1.00 0.00 A2 C

ATOM 1485 O THR A 99 -5.586 10.783 13.708 1.00 0.00 A2 O

ATOM 1486 N THR A 100 -3.866 9.394 13.253 1.00 0.00 A2 N

ATOM 1487 HN THR A 100 -3.511 8.597 12.765 0.00 0.00 A2 H

ATOM 1488 CA THR A 100 -2.871 10.094 14.063 1.00 0.00 A2 C

ATOM 1489 HA THR A 100 -3.275 11.072 14.288 0.00 0.00 A2 H

ATOM 1490 CB THR A 100 -2.450 9.268 15.318 1.00 0.00 A2 C

ATOM 1491 HB THR A 100 -2.052 8.298 14.932 0.00 0.00 A2 H

ATOM 1492 OG1 THR A 100 -3.574 9.009 16.069 1.00 0.00 A2 O

ATOM 1493 HG1 THR A 100 -3.263 8.511 16.833 0.00 0.00 A2 H

ATOM 1494 CG2 THR A 100 -1.486 10.142 16.191 1.00 0.00 A2 C

ATOM 1495 1HG2 THR A 100 -1.073 9.551 17.035 0.00 0.00 A2 H

ATOM 1496 2HG2 THR A 100 -0.633 10.506 15.580 0.00 0.00 A2 H

ATOM 1497 3HG2 THR A 100 -2.015 11.025 16.610 0.00 0.00 A2 H

ATOM 1498 C THR A 100 -1.634 10.323 13.206 1.00 0.00 A2 C

ATOM 1499 O THR A 100 -1.083 9.366 12.649 1.00 0.00 A2 O

ATOM 1500 N GLN A 101 -1.188 11.547 13.157 1.00 0.00 A2 N

ATOM 1501 HN GLN A 101 -1.634 12.317 13.609 0.00 0.00 A2 H

ATOM 1502 CA GLN A 101 0.056 11.907 12.403 1.00 0.00 A2 C

ATOM 1503 HA GLN A 101 0.195 11.173 11.618 0.00 0.00 A2 H

ATOM 1504 CB GLN A 101 0.067 13.369 11.962 1.00 0.00 A2 C

ATOM 1505 HB1 GLN A 101 0.146 13.984 12.888 0.00 0.00 A2 H

ATOM 1506 HB2 GLN A 101 0.993 13.571 11.376 0.00 0.00 A2 H

ATOM 1507 CG GLN A 101 -1.028 13.997 11.191 1.00 0.00 A2 C

ATOM 1508 HG1 GLN A 101 -1.094 13.549 10.176 0.00 0.00 A2 H

ATOM 1509 HG2 GLN A 101 -2.003 13.841 11.696 0.00 0.00 A2 H

ATOM 1510 CD GLN A 101 -0.805 15.514 10.989 1.00 0.00 A2 C

ATOM 1511 OE1 GLN A 101 -0.204 16.282 11.793 1.00 0.00 A2 O

ATOM 1512 NE2 GLN A 101 -1.309 15.955 9.842 1.00 0.00 A2 N

ATOM 1513 1HE2 GLN A 101 -1.197 16.925 9.645 0.00 0.00 A2 H

ATOM 1514 2HE2 GLN A 101 -1.785 15.326 9.237 0.00 0.00 A2 H

ATOM 1515 C GLN A 101 1.251 11.808 13.340 1.00 0.00 A2 C

ATOM 1516 O GLN A 101 1.148 12.204 14.551 1.00 0.00 A2 O

ATOM 1517 N ALA A 102 2.388 11.361 12.828 1.00 0.00 A2 N

ATOM 1518 HN ALA A 102 2.506 11.055 11.884 0.00 0.00 A2 H

ATOM 1519 CA ALA A 102 3.596 11.290 13.673 1.00 0.00 A2 C

ATOM 1520 HA ALA A 102 3.668 12.129 14.355 0.00 0.00 A2 H

ATOM 1521 CB ALA A 102 3.535 10.008 14.483 1.00 0.00 A2 C

ATOM 1522 HB1 ALA A 102 2.656 10.038 15.162 0.00 0.00 A2 H

ATOM 1523 HB2 ALA A 102 3.426 9.122 13.820 0.00 0.00 A2 H

ATOM 1524 HB3 ALA A 102 4.446 9.874 15.106 0.00 0.00 A2 H

ATOM 1525 C ALA A 102 4.799 11.376 12.762 1.00 0.00 A2 C

ATOM 1526 O ALA A 102 4.752 11.240 11.563 1.00 0.00 A2 O

ATOM 1527 N ASN A 103 5.933 11.581 13.417 1.00 0.00 A2 N

ATOM 1528 HN ASN A 103 5.979 11.719 14.406 0.00 0.00 A2 H

ATOM 1529 CA ASN A 103 7.271 11.624 12.716 1.00 0.00 A2 C

ATOM 1530 HA ASN A 103 7.172 11.170 11.736 0.00 0.00 A2 H

ATOM 1531 CB ASN A 103 7.872 13.004 12.525 1.00 0.00 A2 C

ATOM 1532 HB1 ASN A 103 7.713 13.577 13.465 0.00 0.00 A2 H

ATOM 1533 HB2 ASN A 103 8.966 12.929 12.362 0.00 0.00 A2 H

ATOM 1534 CG ASN A 103 7.281 13.839 11.411 1.00 0.00 A2 C

ATOM 1535 OD1 ASN A 103 6.239 14.525 11.561 1.00 0.00 A2 O

ATOM 1536 ND2 ASN A 103 7.927 13.835 10.244 1.00 0.00 A2 N

ATOM 1537 1HD2 ASN A 103 7.549 14.392 9.510 0.00 0.00 A2 H

ATOM 1538 2HD2 ASN A 103 8.768 13.313 10.142 0.00 0.00 A2 H

ATOM 1539 C ASN A 103 8.157 10.708 13.563 1.00 0.00 A2 C

ATOM 1540 O ASN A 103 8.532 11.146 14.651 1.00 0.00 A2 O

ATOM 1541 N LYS A 104 8.463 9.548 13.100 1.00 0.00 A2 N

ATOM 1542 HN LYS A 104 8.226 9.264 12.172 0.00 0.00 A2 H

ATOM 1543 CA LYS A 104 9.178 8.556 13.885 1.00 0.00 A2 C

ATOM 1544 HA LYS A 104 9.796 9.093 14.595 0.00 0.00 A2 H

ATOM 1545 CB LYS A 104 8.132 7.634 14.532 1.00 0.00 A2 C

ATOM 1546 HB1 LYS A 104 7.405 7.354 13.735 0.00 0.00 A2 H

ATOM 1547 HB2 LYS A 104 8.628 6.689 14.849 0.00 0.00 A2 H

ATOM 1548 CG LYS A 104 7.415 8.318 15.684 1.00 0.00 A2 C

ATOM 1549 HG1 LYS A 104 8.170 8.766 16.369 0.00 0.00 A2 H

ATOM 1550 HG2 LYS A 104 6.795 9.152 15.281 0.00 0.00 A2 H

ATOM 1551 CD LYS A 104 6.549 7.357 16.472 1.00 0.00 A2 C

ATOM 1552 HD1 LYS A 104 5.724 7.004 15.811 0.00 0.00 A2 H

ATOM 1553 HD2 LYS A 104 7.168 6.469 16.736 0.00 0.00 A2 H

ATOM 1554 CE LYS A 104 5.986 7.996 17.732 1.00 0.00 A2 C

ATOM 1555 HE1 LYS A 104 5.416 8.918 17.480 0.00 0.00 A2 H

ATOM 1556 HE2 LYS A 104 5.303 7.292 18.256 0.00 0.00 A2 H

ATOM 1557 NZ LYS A 104 7.021 8.397 18.723 1.00 0.00 A2 N

ATOM 1558 HZ1 LYS A 104 6.569 8.826 19.556 0.00 0.00 A2 H

ATOM 1559 HZ2 LYS A 104 7.563 7.559 19.017 0.00 0.00 A2 H

ATOM 1560 HZ3 LYS A 104 7.667 9.086 18.288 0.00 0.00 A2 H

ATOM 1561 C LYS A 104 10.094 7.745 13.019 1.00 0.00 A2 C

ATOM 1562 O LYS A 104 9.959 7.748 11.811 1.00 0.00 A2 O

ATOM 1563 N HSD A 105 10.971 6.990 13.681 1.00 0.00 A2 N

ATOM 1564 HN HSD A 105 11.089 6.984 14.673 0.00 0.00 A2 H

ATOM 1565 CA HSD A 105 11.842 6.093 12.951 1.00 0.00 A2 C

ATOM 1566 HA HSD A 105 12.113 6.504 11.986 0.00 0.00 A2 H

ATOM 1567 CB HSD A 105 13.038 5.577 13.751 1.00 0.00 A2 C

ATOM 1568 HB1 HSD A 105 12.667 5.017 14.637 0.00 0.00 A2 H

ATOM 1569 HB2 HSD A 105 13.615 4.854 13.133 0.00 0.00 A2 H

ATOM 1570 ND1 HSD A 105 15.008 7.090 13.494 1.00 0.00 A2 N

ATOM 1571 HD1 HSD A 105 15.276 6.732 12.599 0.00 0.00 A2 H

ATOM 1572 CG HSD A 105 13.942 6.712 14.211 1.00 0.00 A2 C

ATOM 1573 CE1 HSD A 105 15.556 8.118 14.169 1.00 0.00 A2 C

ATOM 1574 HE1 HSD A 105 16.464 8.640 13.854 0.00 0.00 A2 H

ATOM 1575 NE2 HSD A 105 14.841 8.394 15.236 1.00 0.00 A2 N

ATOM 1576 CD2 HSD A 105 13.808 7.495 15.343 1.00 0.00 A2 C

ATOM 1577 HD2 HSD A 105 13.095 7.419 16.155 0.00 0.00 A2 H

ATOM 1578 C HSD A 105 10.931 4.871 12.693 1.00 0.00 A2 C

ATOM 1579 O HSD A 105 10.068 4.581 13.497 1.00 0.00 A2 O

ATOM 1580 N ILE A 106 11.211 4.178 11.613 1.00 0.00 A2 N

ATOM 1581 HN ILE A 106 11.934 4.393 10.959 0.00 0.00 A2 H

ATOM 1582 CA ILE A 106 10.439 2.981 11.281 1.00 0.00 A2 C

ATOM 1583 HA ILE A 106 9.674 2.865 12.039 0.00 0.00 A2 H

ATOM 1584 CB ILE A 106 9.767 3.066 9.925 1.00 0.00 A2 C

ATOM 1585 HB ILE A 106 9.249 2.080 9.807 0.00 0.00 A2 H

ATOM 1586 CG2 ILE A 106 8.814 4.247 9.939 1.00 0.00 A2 C

ATOM 1587 1HG2 ILE A 106 8.187 4.254 9.023 0.00 0.00 A2 H

ATOM 1588 2HG2 ILE A 106 8.132 4.195 10.813 0.00 0.00 A2 H

ATOM 1589 3HG2 ILE A 106 9.371 5.207 9.985 0.00 0.00 A2 H

ATOM 1590 CG1 ILE A 106 10.726 3.153 8.788 1.00 0.00 A2 C

ATOM 1591 1HG1 ILE A 106 11.189 4.165 8.762 0.00 0.00 A2 H

ATOM 1592 2HG1 ILE A 106 11.548 2.419 8.952 0.00 0.00 A2 H

ATOM 1593 CD ILE A 106 10.109 2.860 7.388 1.00 0.00 A2 C

ATOM 1594 HD1 ILE A 106 10.898 2.888 6.605 0.00 0.00 A2 H

ATOM 1595 HD2 ILE A 106 9.637 1.854 7.370 0.00 0.00 A2 H

ATOM 1596 HD3 ILE A 106 9.338 3.615 7.126 0.00 0.00 A2 H

ATOM 1597 C ILE A 106 11.382 1.739 11.396 1.00 0.00 A2 C

ATOM 1598 O ILE A 106 12.566 1.845 11.173 1.00 0.00 A2 O

ATOM 1599 N ILE A 107 10.739 0.629 11.701 1.00 0.00 A2 N

ATOM 1600 HN ILE A 107 9.763 0.595 11.906 0.00 0.00 A2 H

ATOM 1601 CA ILE A 107 11.402 -0.651 11.766 1.00 0.00 A2 C

ATOM 1602 HA ILE A 107 12.406 -0.538 11.377 0.00 0.00 A2 H

ATOM 1603 CB ILE A 107 11.457 -1.277 13.151 1.00 0.00 A2 C

ATOM 1604 HB ILE A 107 10.386 -1.350 13.468 0.00 0.00 A2 H

ATOM 1605 CG2 ILE A 107 12.209 -2.588 13.140 1.00 0.00 A2 C

ATOM 1606 1HG2 ILE A 107 12.107 -3.105 14.117 0.00 0.00 A2 H

ATOM 1607 2HG2 ILE A 107 11.811 -3.267 12.357 0.00 0.00 A2 H

ATOM 1608 3HG2 ILE A 107 13.290 -2.424 12.946 0.00 0.00 A2 H

ATOM 1609 CG1 ILE A 107 12.164 -0.300 14.143 1.00 0.00 A2 C

ATOM 1610 1HG1 ILE A 107 13.210 -0.117 13.810 0.00 0.00 A2 H

ATOM 1611 2HG1 ILE A 107 11.635 0.681 14.122 0.00 0.00 A2 H

ATOM 1612 CD ILE A 107 12.244 -0.676 15.573 1.00 0.00 A2 C

ATOM 1613 HD1 ILE A 107 12.720 0.139 16.160 0.00 0.00 A2 H

ATOM 1614 HD2 ILE A 107 11.229 -0.857 15.988 0.00 0.00 A2 H

ATOM 1615 HD3 ILE A 107 12.850 -1.596 15.710 0.00 0.00 A2 H

ATOM 1616 C ILE A 107 10.634 -1.591 10.773 1.00 0.00 A2 C

ATOM 1617 O ILE A 107 9.427 -1.819 10.981 1.00 0.00 A2 O

ATOM 1618 N VAL A 108 11.371 -2.120 9.835 1.00 0.00 A2 N

ATOM 1619 HN VAL A 108 12.340 -1.916 9.721 0.00 0.00 A2 H

ATOM 1620 CA VAL A 108 10.824 -3.086 8.840 1.00 0.00 A2 C

ATOM 1621 HA VAL A 108 9.820 -3.339 9.156 0.00 0.00 A2 H

ATOM 1622 CB VAL A 108 10.814 -2.436 7.422 1.00 0.00 A2 C

ATOM 1623 HB VAL A 108 10.401 -3.190 6.708 0.00 0.00 A2 H

ATOM 1624 CG1 VAL A 108 9.921 -1.179 7.396 1.00 0.00 A2 C

ATOM 1625 1HG1 VAL A 108 9.953 -0.704 6.392 0.00 0.00 A2 H

ATOM 1626 2HG1 VAL A 108 8.861 -1.425 7.611 0.00 0.00 A2 H

ATOM 1627 3HG1 VAL A 108 10.271 -0.433 8.141 0.00 0.00 A2 H

ATOM 1628 CG2 VAL A 108 12.176 -2.129 6.883 1.00 0.00 A2 C

ATOM 1629 1HG2 VAL A 108 12.104 -1.633 5.892 0.00 0.00 A2 H

ATOM 1630 2HG2 VAL A 108 12.726 -1.453 7.573 0.00 0.00 A2 H

ATOM 1631 3HG2 VAL A 108 12.774 -3.054 6.752 0.00 0.00 A2 H

ATOM 1632 C VAL A 108 11.640 -4.385 8.814 1.00 0.00 A2 C

ATOM 1633 O VAL A 108 12.806 -4.349 9.109 1.00 0.00 A2 O

ATOM 1634 N ALA A 109 10.963 -5.445 8.378 1.00 0.00 A2 N

ATOM 1635 HN ALA A 109 9.982 -5.415 8.189 0.00 0.00 A2 H

ATOM 1636 CA ALA A 109 11.613 -6.744 8.142 1.00 0.00 A2 C

ATOM 1637 HA ALA A 109 12.587 -6.835 8.610 0.00 0.00 A2 H

ATOM 1638 CB ALA A 109 10.867 -7.954 8.542 1.00 0.00 A2 C

ATOM 1639 HB1 ALA A 109 10.731 -7.961 9.645 0.00 0.00 A2 H

ATOM 1640 HB2 ALA A 109 9.859 -7.975 8.073 0.00 0.00 A2 H

ATOM 1641 HB3 ALA A 109 11.409 -8.882 8.257 0.00 0.00 A2 H

ATOM 1642 C ALA A 109 11.860 -6.778 6.600 1.00 0.00 A2 C

ATOM 1643 O ALA A 109 11.010 -6.380 5.844 1.00 0.00 A2 O

ATOM 1644 SG CYS A 110 14.390 -4.719 5.055 1.00 0.00 A2 S

ATOM 1645 CB CYS A 110 14.731 -6.460 4.610 1.00 0.00 A2 C

ATOM 1646 N CYS A 110 13.066 -7.260 6.226 1.00 0.00 A2 N

ATOM 1647 HN CYS A 110 13.743 -7.598 6.877 0.00 0.00 A2 H

ATOM 1648 CA CYS A 110 13.471 -7.324 4.845 1.00 0.00 A2 C

ATOM 1649 HA CYS A 110 12.631 -6.956 4.268 0.00 0.00 A2 H

ATOM 1650 HB1 CYS A 110 15.539 -6.871 5.257 0.00 0.00 A2 H

ATOM 1651 HB2 CYS A 110 15.084 -6.584 3.562 0.00 0.00 A2 H

ATOM 1652 C CYS A 110 13.752 -8.761 4.395 1.00 0.00 A2 C

ATOM 1653 O CYS A 110 14.245 -9.563 5.192 1.00 0.00 A2 O

ATOM 1654 N GLU A 111 13.475 -8.994 3.119 1.00 0.00 A2 N

ATOM 1655 HN GLU A 111 13.075 -8.358 2.465 0.00 0.00 A2 H

ATOM 1656 CA GLU A 111 13.776 -10.342 2.550 1.00 0.00 A2 C

ATOM 1657 HA GLU A 111 14.698 -10.712 2.981 0.00 0.00 A2 H

ATOM 1658 CB GLU A 111 12.596 -11.291 2.653 1.00 0.00 A2 C

ATOM 1659 HB1 GLU A 111 11.745 -10.849 2.085 0.00 0.00 A2 H

ATOM 1660 HB2 GLU A 111 12.838 -12.250 2.143 0.00 0.00 A2 H

ATOM 1661 CG GLU A 111 12.088 -11.616 4.024 1.00 0.00 A2 C

ATOM 1662 HG1 GLU A 111 12.876 -12.140 4.595 0.00 0.00 A2 H

ATOM 1663 HG2 GLU A 111 11.795 -10.696 4.567 0.00 0.00 A2 H

ATOM 1664 CD GLU A 111 10.901 -12.552 4.147 1.00 0.00 A2 C

ATOM 1665 OE1 GLU A 111 10.306 -12.638 5.226 1.00 0.00 A2 O

ATOM 1666 OE2 GLU A 111 10.603 -13.176 3.043 1.00 0.00 A2 O

ATOM 1667 C GLU A 111 14.002 -10.206 1.036 1.00 0.00 A2 C

ATOM 1668 O GLU A 111 13.543 -9.267 0.468 1.00 0.00 A2 O

ATOM 1669 N GLY A 112 14.619 -11.269 0.503 1.00 0.00 A2 N

ATOM 1670 HN GLY A 112 15.000 -11.975 1.098 0.00 0.00 A2 H

ATOM 1671 CA GLY A 112 14.785 -11.488 -0.904 1.00 0.00 A2 C

ATOM 1672 HA1 GLY A 112 13.878 -11.094 -1.342 0.00 0.00 A2 H

ATOM 1673 HA2 GLY A 112 14.895 -12.560 -0.998 0.00 0.00 A2 H

ATOM 1674 C GLY A 112 15.945 -10.845 -1.588 1.00 0.00 A2 C

ATOM 1675 O GLY A 112 16.829 -10.307 -0.994 1.00 0.00 A2 O

ATOM 1676 N ASN A 113 15.911 -11.003 -2.937 1.00 0.00 A2 N

ATOM 1677 HN ASN A 113 15.222 -11.524 -3.439 0.00 0.00 A2 H

ATOM 1678 CA ASN A 113 16.941 -10.384 -3.781 1.00 0.00 A2 C

ATOM 1679 HA ASN A 113 17.398 -9.575 -3.222 0.00 0.00 A2 H

ATOM 1680 CB ASN A 113 18.111 -11.326 -4.097 1.00 0.00 A2 C

ATOM 1681 HB1 ASN A 113 18.645 -11.537 -3.145 0.00 0.00 A2 H

ATOM 1682 HB2 ASN A 113 17.733 -12.293 -4.488 0.00 0.00 A2 H

ATOM 1683 CG ASN A 113 19.077 -10.680 -5.070 1.00 0.00 A2 C

ATOM 1684 OD1 ASN A 113 19.810 -9.730 -4.656 1.00 0.00 A2 O

ATOM 1685 ND2 ASN A 113 19.141 -11.186 -6.318 1.00 0.00 A2 N

ATOM 1686 1HD2 ASN A 113 19.786 -10.765 -6.950 0.00 0.00 A2 H

ATOM 1687 2HD2 ASN A 113 18.580 -11.969 -6.568 0.00 0.00 A2 H

ATOM 1688 C ASN A 113 16.182 -9.758 -4.974 1.00 0.00 A2 C

ATOM 1689 O ASN A 113 15.605 -10.584 -5.758 1.00 0.00 A2 O

ATOM 1690 N PRO A 114 16.097 -8.436 -5.115 1.00 0.00 A2 N

ATOM 1691 CD PRO A 114 15.361 -7.869 -6.266 1.00 0.00 A2 C

ATOM 1692 HD1 PRO A 114 16.166 -8.168 -6.975 0.00 0.00 A2 H

ATOM 1693 HD2 PRO A 114 14.369 -8.221 -6.632 0.00 0.00 A2 H

ATOM 1694 CA PRO A 114 16.654 -7.417 -4.274 1.00 0.00 A2 C

ATOM 1695 HA PRO A 114 17.732 -7.501 -4.199 0.00 0.00 A2 H

ATOM 1696 CB PRO A 114 16.189 -6.071 -4.918 1.00 0.00 A2 C

ATOM 1697 HB1 PRO A 114 16.902 -5.790 -5.726 0.00 0.00 A2 H

ATOM 1698 HB2 PRO A 114 16.132 -5.242 -4.184 0.00 0.00 A2 H

ATOM 1699 CG PRO A 114 15.348 -6.365 -6.102 1.00 0.00 A2 C

ATOM 1700 HG1 PRO A 114 15.320 -5.531 -6.830 0.00 0.00 A2 H

ATOM 1701 HG2 PRO A 114 14.311 -6.599 -5.767 0.00 0.00 A2 H

ATOM 1702 C PRO A 114 16.023 -7.539 -2.825 1.00 0.00 A2 C

ATOM 1703 O PRO A 114 14.879 -8.025 -2.646 1.00 0.00 A2 O

ATOM 1704 N TYR A 115 16.801 -7.087 -1.910 1.00 0.00 A2 N

ATOM 1705 HN TYR A 115 17.689 -6.676 -2.109 0.00 0.00 A2 H

ATOM 1706 CA TYR A 115 16.450 -7.133 -0.462 1.00 0.00 A2 C

ATOM 1707 HA TYR A 115 15.863 -8.030 -0.303 0.00 0.00 A2 H

ATOM 1708 CB TYR A 115 17.767 -7.157 0.283 1.00 0.00 A2 C

ATOM 1709 HB1 TYR A 115 18.450 -7.885 -0.207 0.00 0.00 A2 H

ATOM 1710 HB2 TYR A 115 18.261 -6.162 0.239 0.00 0.00 A2 H

ATOM 1711 CG TYR A 115 17.741 -7.570 1.719 1.00 0.00 A2 C

ATOM 1712 CD1 TYR A 115 17.257 -8.850 2.086 1.00 0.00 A2 C

ATOM 1713 HD1 TYR A 115 16.855 -9.510 1.329 0.00 0.00 A2 H

ATOM 1714 CE1 TYR A 115 17.255 -9.297 3.392 1.00 0.00 A2 C

ATOM 1715 HE1 TYR A 115 16.858 -10.270 3.639 0.00 0.00 A2 H

ATOM 1716 CZ TYR A 115 17.775 -8.450 4.385 1.00 0.00 A2 C

ATOM 1717 OH TYR A 115 17.829 -8.849 5.650 1.00 0.00 A2 O

ATOM 1718 HH TYR A 115 18.277 -8.163 6.149 0.00 0.00 A2 H

ATOM 1719 CD2 TYR A 115 18.259 -6.743 2.710 1.00 0.00 A2 C

ATOM 1720 HD2 TYR A 115 18.611 -5.752 2.457 0.00 0.00 A2 H

ATOM 1721 CE2 TYR A 115 18.274 -7.171 4.015 1.00 0.00 A2 C

ATOM 1722 HE2 TYR A 115 18.653 -6.520 4.788 0.00 0.00 A2 H

ATOM 1723 C TYR A 115 15.580 -5.971 -0.118 1.00 0.00 A2 C

ATOM 1724 O TYR A 115 16.125 -4.870 -0.023 1.00 0.00 A2 O

ATOM 1725 N VAL A 116 14.308 -6.254 0.087 1.00 0.00 A2 N

ATOM 1726 HN VAL A 116 13.923 -7.173 0.120 0.00 0.00 A2 H

ATOM 1727 CA VAL A 116 13.330 -5.179 0.289 1.00 0.00 A2 C

ATOM 1728 HA VAL A 116 13.889 -4.274 0.491 0.00 0.00 A2 H

ATOM 1729 CB VAL A 116 12.516 -5.120 -1.060 1.00 0.00 A2 C

ATOM 1730 HB VAL A 116 11.685 -4.386 -0.921 0.00 0.00 A2 H

ATOM 1731 CG1 VAL A 116 13.414 -4.650 -2.224 1.00 0.00 A2 C

ATOM 1732 1HG1 VAL A 116 12.842 -4.649 -3.177 0.00 0.00 A2 H

ATOM 1733 2HG1 VAL A 116 13.788 -3.618 -2.062 0.00 0.00 A2 H

ATOM 1734 3HG1 VAL A 116 14.285 -5.329 -2.347 0.00 0.00 A2 H

ATOM 1735 CG2 VAL A 116 11.812 -6.430 -1.404 1.00 0.00 A2 C

ATOM 1736 1HG2 VAL A 116 11.287 -6.349 -2.380 0.00 0.00 A2 H

ATOM 1737 2HG2 VAL A 116 12.546 -7.262 -1.469 0.00 0.00 A2 H

ATOM 1738 3HG2 VAL A 116 11.054 -6.693 -0.637 0.00 0.00 A2 H

ATOM 1739 C VAL A 116 12.444 -5.443 1.487 1.00 0.00 A2 C

ATOM 1740 O VAL A 116 12.333 -6.543 1.978 1.00 0.00 A2 O

ATOM 1741 N PRO A 117 11.746 -4.381 1.911 1.00 0.00 A2 N

ATOM 1742 CD PRO A 117 11.853 -2.944 1.451 1.00 0.00 A2 C

ATOM 1743 HD1 PRO A 117 12.808 -3.439 1.738 0.00 0.00 A2 H

ATOM 1744 HD2 PRO A 117 12.014 -2.256 0.589 0.00 0.00 A2 H

ATOM 1745 CA PRO A 117 10.821 -4.512 3.038 1.00 0.00 A2 C

ATOM 1746 HA PRO A 117 11.310 -4.972 3.889 0.00 0.00 A2 H

ATOM 1747 CB PRO A 117 10.328 -3.087 3.343 1.00 0.00 A2 C

ATOM 1748 HB1 PRO A 117 11.060 -2.586 4.015 0.00 0.00 A2 H

ATOM 1749 HB2 PRO A 117 9.329 -3.073 3.825 0.00 0.00 A2 H

ATOM 1750 CG PRO A 117 11.288 -2.170 2.638 1.00 0.00 A2 C

ATOM 1751 HG1 PRO A 117 11.319 -1.155 3.079 0.00 0.00 A2 H

ATOM 1752 HG2 PRO A 117 11.004 -2.098 1.563 0.00 0.00 A2 H

ATOM 1753 C PRO A 117 9.595 -5.357 2.645 1.00 0.00 A2 C

ATOM 1754 O PRO A 117 8.973 -5.182 1.622 1.00 0.00 A2 O

ATOM 1755 N VAL A 118 9.299 -6.257 3.582 1.00 0.00 A2 N

ATOM 1756 HN VAL A 118 9.834 -6.380 4.414 0.00 0.00 A2 H

ATOM 1757 CA VAL A 118 8.153 -7.149 3.466 1.00 0.00 A2 C

ATOM 1758 HA VAL A 118 7.626 -6.841 2.572 0.00 0.00 A2 H

ATOM 1759 CB VAL A 118 8.558 -8.606 3.127 1.00 0.00 A2 C

ATOM 1760 HB VAL A 118 7.619 -9.205 3.037 0.00 0.00 A2 H

ATOM 1761 CG1 VAL A 118 9.313 -8.656 1.807 1.00 0.00 A2 C

ATOM 1762 1HG1 VAL A 118 9.636 -9.696 1.587 0.00 0.00 A2 H

ATOM 1763 2HG1 VAL A 118 8.680 -8.320 0.960 0.00 0.00 A2 H

ATOM 1764 3HG1 VAL A 118 10.220 -8.016 1.849 0.00 0.00 A2 H

ATOM 1765 CG2 VAL A 118 9.300 -9.193 4.298 1.00 0.00 A2 C

ATOM 1766 1HG2 VAL A 118 9.639 -10.226 4.070 0.00 0.00 A2 H

ATOM 1767 2HG2 VAL A 118 10.193 -8.578 4.543 0.00 0.00 A2 H

ATOM 1768 3HG2 VAL A 118 8.654 -9.244 5.199 0.00 0.00 A2 H

ATOM 1769 C VAL A 118 7.178 -7.062 4.621 1.00 0.00 A2 C

ATOM 1770 O VAL A 118 6.071 -7.610 4.561 1.00 0.00 A2 O

ATOM 1771 N HSD A 119 7.564 -6.426 5.742 1.00 0.00 A2 N

ATOM 1772 HN HSD A 119 8.464 -6.000 5.825 0.00 0.00 A2 H

ATOM 1773 CA HSD A 119 6.710 -6.307 6.916 1.00 0.00 A2 C

ATOM 1774 HA HSD A 119 5.678 -6.227 6.598 0.00 0.00 A2 H

ATOM 1775 CB HSD A 119 6.962 -7.539 7.788 1.00 0.00 A2 C

ATOM 1776 HB1 HSD A 119 6.389 -8.398 7.376 0.00 0.00 A2 H

ATOM 1777 HB2 HSD A 119 8.038 -7.817 7.737 0.00 0.00 A2 H

ATOM 1778 ND1 HSD A 119 5.350 -7.688 9.665 1.00 0.00 A2 N

ATOM 1779 HD1 HSD A 119 4.564 -7.974 9.116 0.00 0.00 A2 H

ATOM 1780 CG HSD A 119 6.620 -7.478 9.209 1.00 0.00 A2 C

ATOM 1781 CE1 HSD A 119 5.390 -7.554 10.989 1.00 0.00 A2 C

ATOM 1782 HE1 HSD A 119 4.518 -7.664 11.639 0.00 0.00 A2 H

ATOM 1783 NE2 HSD A 119 6.666 -7.273 11.393 1.00 0.00 A2 N

ATOM 1784 CD2 HSD A 119 7.455 -7.222 10.283 1.00 0.00 A2 C

ATOM 1785 HD2 HSD A 119 8.513 -6.987 10.295 0.00 0.00 A2 H

ATOM 1786 C HSD A 119 7.068 -5.011 7.622 1.00 0.00 A2 C

ATOM 1787 O HSD A 119 8.214 -4.663 7.740 1.00 0.00 A2 O

ATOM 1788 N PHE A 120 6.069 -4.401 8.161 1.00 0.00 A2 N

ATOM 1789 HN PHE A 120 5.130 -4.721 8.049 0.00 0.00 A2 H

ATOM 1790 CA PHE A 120 6.210 -3.147 9.013 1.00 0.00 A2 C

ATOM 1791 HA PHE A 120 7.196 -2.723 8.866 0.00 0.00 A2 H

ATOM 1792 CB PHE A 120 5.079 -2.174 8.701 1.00 0.00 A2 C

ATOM 1793 HB1 PHE A 120 5.028 -2.016 7.602 0.00 0.00 A2 H

ATOM 1794 HB2 PHE A 120 4.097 -2.586 9.019 0.00 0.00 A2 H

ATOM 1795 CG PHE A 120 5.344 -0.825 9.351 1.00 0.00 A2 C

ATOM 1796 CD1 PHE A 120 4.973 -0.617 10.656 1.00 0.00 A2 C

ATOM 1797 HD1 PHE A 120 4.460 -1.400 11.198 0.00 0.00 A2 H

ATOM 1798 CE1 PHE A 120 5.257 0.612 11.273 1.00 0.00 A2 C

ATOM 1799 HE1 PHE A 120 4.972 0.779 12.302 0.00 0.00 A2 H

ATOM 1800 CZ PHE A 120 5.903 1.565 10.528 1.00 0.00 A2 C

ATOM 1801 HZ PHE A 120 6.159 2.480 11.042 0.00 0.00 A2 H

ATOM 1802 CD2 PHE A 120 5.971 0.147 8.598 1.00 0.00 A2 C

ATOM 1803 HD2 PHE A 120 6.229 -0.051 7.567 0.00 0.00 A2 H

ATOM 1804 CE2 PHE A 120 6.234 1.406 9.209 1.00 0.00 A2 C

ATOM 1805 HE2 PHE A 120 6.682 2.202 8.631 0.00 0.00 A2 H

ATOM 1806 C PHE A 120 6.157 -3.660 10.422 1.00 0.00 A2 C

ATOM 1807 O PHE A 120 5.100 -4.166 10.891 1.00 0.00 A2 O

ATOM 1808 N ASP A 121 7.252 -3.488 11.173 1.00 0.00 A2 N

ATOM 1809 HN ASP A 121 8.080 -3.010 10.891 0.00 0.00 A2 H

ATOM 1810 CA ASP A 121 7.331 -4.009 12.531 1.00 0.00 A2 C

ATOM 1811 HA ASP A 121 6.640 -4.844 12.573 0.00 0.00 A2 H

ATOM 1812 CB ASP A 121 8.743 -4.516 12.791 1.00 0.00 A2 C

ATOM 1813 HB1 ASP A 121 9.134 -4.996 11.871 0.00 0.00 A2 H

ATOM 1814 HB2 ASP A 121 9.416 -3.678 13.060 0.00 0.00 A2 H

ATOM 1815 CG ASP A 121 8.758 -5.545 13.902 1.00 0.00 A2 C

ATOM 1816 OD1 ASP A 121 8.020 -6.531 13.856 1.00 0.00 A2 O

ATOM 1817 OD2 ASP A 121 9.472 -5.411 14.919 1.00 0.00 A2 O

ATOM 1818 C ASP A 121 6.892 -3.042 13.553 1.00 0.00 A2 C

ATOM 1819 O ASP A 121 6.126 -3.390 14.444 1.00 0.00 A2 O

ATOM 1820 N ALA A 122 7.330 -1.797 13.516 1.00 0.00 A2 N

ATOM 1821 HN ALA A 122 7.913 -1.508 12.758 0.00 0.00 A2 H

ATOM 1822 CA ALA A 122 7.039 -0.780 14.496 1.00 0.00 A2 C

ATOM 1823 HA ALA A 122 5.966 -0.687 14.622 0.00 0.00 A2 H

ATOM 1824 CB ALA A 122 7.847 -1.156 15.766 1.00 0.00 A2 C

ATOM 1825 HB1 ALA A 122 7.495 -2.135 16.156 0.00 0.00 A2 H

ATOM 1826 HB2 ALA A 122 8.931 -1.249 15.537 0.00 0.00 A2 H

ATOM 1827 HB3 ALA A 122 7.719 -0.400 16.571 0.00 0.00 A2 H

ATOM 1828 C ALA A 122 7.546 0.599 14.084 1.00 0.00 A2 C

ATOM 1829 O ALA A 122 8.345 0.758 13.165 1.00 0.00 A2 O

ATOM 1830 N SER A 123 7.072 1.585 14.827 1.00 0.00 A2 N

ATOM 1831 HN SER A 123 6.363 1.459 15.521 0.00 0.00 A2 H

ATOM 1832 CA SER A 123 7.556 2.987 14.697 1.00 0.00 A2 C

ATOM 1833 HA SER A 123 8.404 3.014 14.025 0.00 0.00 A2 H

ATOM 1834 CB SER A 123 6.573 3.963 14.165 1.00 0.00 A2 C

ATOM 1835 HB1 SER A 123 7.106 4.922 13.973 0.00 0.00 A2 H

ATOM 1836 HB2 SER A 123 6.168 3.613 13.188 0.00 0.00 A2 H

ATOM 1837 OG SER A 123 5.567 4.160 15.121 1.00 0.00 A2 O

ATOM 1838 HG1 SER A 123 5.074 4.947 14.856 0.00 0.00 A2 H

ATOM 1839 C SER A 123 8.087 3.319 16.128 1.00 0.00 A2 C

ATOM 1840 O SER A 123 7.413 2.951 17.142 1.00 0.00 A2 O

ATOM 1841 C VAL A 124 10.327 5.703 17.430 1.00 0.00 A2 C

ATOM 1842 OT1 VAL A 124 10.090 6.076 16.533 0.00 0.00 A2 O

ATOM 1843 OT2 VAL A 124 10.757 5.951 18.298 0.00 0.00 A2 O

ATOM 1844 N VAL A 124 9.239 3.900 16.197 1.00 0.00 A2 N

ATOM 1845 HN VAL A 124 9.775 4.150 15.395 0.00 0.00 A2 H

ATOM 1846 CA VAL A 124 9.877 4.252 17.498 1.00 0.00 A2 C

ATOM 1847 HA VAL A 124 9.133 4.179 18.281 0.00 0.00 A2 H

ATOM 1848 CB VAL A 124 11.001 3.250 17.854 1.00 0.00 A2 C

ATOM 1849 HB VAL A 124 11.496 3.616 18.787 0.00 0.00 A2 H

ATOM 1850 CG1 VAL A 124 10.460 1.843 18.113 1.00 0.00 A2 C

ATOM 1851 1HG1 VAL A 124 11.295 1.142 18.327 0.00 0.00 A2 H

ATOM 1852 2HG1 VAL A 124 9.778 1.821 18.988 0.00 0.00 A2 H

ATOM 1853 3HG1 VAL A 124 9.912 1.463 17.224 0.00 0.00 A2 H

ATOM 1854 CG2 VAL A 124 12.106 3.186 16.868 1.00 0.00 A2 C

ATOM 1855 1HG2 VAL A 124 12.854 2.421 17.166 0.00 0.00 A2 H

ATOM 1856 2HG2 VAL A 124 11.716 2.921 15.861 0.00 0.00 A2 H

ATOM 1857 3HG2 VAL A 124 12.634 4.159 16.792 0.00 0.00 A2 H

TER

**coordinates of lysozyme**

CRYST1 100.000 100.000 100.000 90.00 90.00 90.00 P1

ATOM 1 N LYS A 1 -6.732 -4.048 -3.048 1.00 0.00 A2 N

ATOM 2 HT1 LYS A 1 -7.436 -3.875 -3.737 0.00 0.00 A2 H

ATOM 3 HT2 LYS A 1 -6.736 -3.317 -2.366 0.00 0.00 A2 H

ATOM 4 HT3 LYS A 1 -6.898 -4.929 -2.605 0.00 0.00 A2 H

ATOM 5 CA LYS A 1 -5.438 -4.082 -3.695 1.00 0.00 A2 C

ATOM 6 HA LYS A 1 -5.433 -4.917 -4.385 0.00 0.00 A2 H

ATOM 7 CB LYS A 1 -5.194 -2.746 -4.385 1.00 0.00 A2 C

ATOM 8 HB1 LYS A 1 -5.953 -2.652 -5.196 0.00 0.00 A2 H

ATOM 9 HB2 LYS A 1 -5.402 -1.923 -3.665 0.00 0.00 A2 H

ATOM 10 CG LYS A 1 -3.843 -2.551 -4.992 1.00 0.00 A2 C

ATOM 11 HG1 LYS A 1 -3.084 -3.074 -4.367 0.00 0.00 A2 H

ATOM 12 HG2 LYS A 1 -3.827 -3.025 -6.000 0.00 0.00 A2 H

ATOM 13 CD LYS A 1 -3.535 -1.060 -5.065 1.00 0.00 A2 C

ATOM 14 HD1 LYS A 1 -4.477 -0.518 -5.309 0.00 0.00 A2 H

ATOM 15 HD2 LYS A 1 -3.209 -0.723 -4.054 0.00 0.00 A2 H

ATOM 16 CE LYS A 1 -2.468 -0.760 -6.086 1.00 0.00 A2 C

ATOM 17 HE1 LYS A 1 -2.928 -0.491 -7.063 0.00 0.00 A2 H

ATOM 18 HE2 LYS A 1 -1.833 0.089 -5.750 0.00 0.00 A2 H

ATOM 19 NZ LYS A 1 -1.607 -1.927 -6.289 1.00 0.00 A2 N

ATOM 20 HZ1 LYS A 1 -0.875 -1.711 -6.996 0.00 0.00 A2 H

ATOM 21 HZ2 LYS A 1 -1.153 -2.184 -5.389 0.00 0.00 A2 H

ATOM 22 HZ3 LYS A 1 -2.182 -2.727 -6.622 0.00 0.00 A2 H

ATOM 23 C LYS A 1 -4.359 -4.344 -2.657 1.00 0.00 A2 C

ATOM 24 O LYS A 1 -4.348 -3.722 -1.603 1.00 0.00 A2 O

ATOM 25 N VAL A 2 -3.452 -5.268 -2.954 1.00 0.00 A2 N

ATOM 26 HN VAL A 2 -3.506 -5.876 -3.742 0.00 0.00 A2 H

ATOM 27 CA VAL A 2 -2.274 -5.466 -2.121 1.00 0.00 A2 C

ATOM 28 HA VAL A 2 -2.388 -4.892 -1.210 0.00 0.00 A2 H

ATOM 29 CB VAL A 2 -2.048 -6.952 -1.785 1.00 0.00 A2 C

ATOM 30 HB VAL A 2 -1.865 -7.491 -2.747 0.00 0.00 A2 H

ATOM 31 CG1 VAL A 2 -0.835 -7.117 -0.880 1.00 0.00 A2 C

ATOM 32 1HG1 VAL A 2 -0.699 -8.186 -0.609 0.00 0.00 A2 H

ATOM 33 2HG1 VAL A 2 0.098 -6.782 -1.379 0.00 0.00 A2 H

ATOM 34 3HG1 VAL A 2 -0.964 -6.538 0.059 0.00 0.00 A2 H

ATOM 35 CG2 VAL A 2 -3.298 -7.541 -1.118 1.00 0.00 A2 C

ATOM 36 1HG2 VAL A 2 -3.130 -8.602 -0.836 0.00 0.00 A2 H

ATOM 37 2HG2 VAL A 2 -3.555 -6.971 -0.199 0.00 0.00 A2 H

ATOM 38 3HG2 VAL A 2 -4.171 -7.511 -1.803 0.00 0.00 A2 H

ATOM 39 C VAL A 2 -1.080 -4.902 -2.868 1.00 0.00 A2 C

ATOM 40 O VAL A 2 -0.762 -5.349 -3.969 1.00 0.00 A2 O

ATOM 41 N PHE A 3 -0.447 -3.889 -2.280 1.00 0.00 A2 N

ATOM 42 HN PHE A 3 -0.720 -3.506 -1.399 0.00 0.00 A2 H

ATOM 43 CA PHE A 3 0.714 -3.247 -2.884 1.00 0.00 A2 C

ATOM 44 HA PHE A 3 0.497 -3.138 -3.940 0.00 0.00 A2 H

ATOM 45 CB PHE A 3 1.056 -1.951 -2.144 1.00 0.00 A2 C

ATOM 46 HB1 PHE A 3 0.827 -2.078 -1.063 0.00 0.00 A2 H

ATOM 47 HB2 PHE A 3 2.140 -1.719 -2.224 0.00 0.00 A2 H

ATOM 48 CG PHE A 3 0.265 -0.752 -2.594 1.00 0.00 A2 C

ATOM 49 CD1 PHE A 3 0.780 0.121 -3.542 1.00 0.00 A2 C

ATOM 50 HD1 PHE A 3 1.754 -0.069 -3.971 0.00 0.00 A2 H

ATOM 51 CE1 PHE A 3 0.069 1.239 -3.945 1.00 0.00 A2 C

ATOM 52 HE1 PHE A 3 0.489 1.910 -4.681 0.00 0.00 A2 H

ATOM 53 CZ PHE A 3 -1.178 1.491 -3.411 1.00 0.00 A2 C

ATOM 54 HZ PHE A 3 -1.748 2.349 -3.738 0.00 0.00 A2 H

ATOM 55 CD2 PHE A 3 -0.973 -0.483 -2.050 1.00 0.00 A2 C

ATOM 56 HD2 PHE A 3 -1.378 -1.128 -1.283 0.00 0.00 A2 H

ATOM 57 CE2 PHE A 3 -1.704 0.633 -2.459 1.00 0.00 A2 C

ATOM 58 HE2 PHE A 3 -2.678 0.825 -2.031 0.00 0.00 A2 H

ATOM 59 C PHE A 3 1.922 -4.151 -2.770 1.00 0.00 A2 C

ATOM 60 O PHE A 3 2.087 -4.845 -1.771 1.00 0.00 A2 O

ATOM 61 N GLU A 4 2.777 -4.131 -3.780 1.00 0.00 A2 N

ATOM 62 HN GLU A 4 2.608 -3.730 -4.676 0.00 0.00 A2 H

ATOM 63 CA GLU A 4 4.102 -4.732 -3.638 1.00 0.00 A2 C

ATOM 64 HA GLU A 4 4.052 -5.615 -3.013 0.00 0.00 A2 H

ATOM 65 CB GLU A 4 4.701 -5.052 -5.010 1.00 0.00 A2 C

ATOM 66 HB1 GLU A 4 4.034 -5.786 -5.518 0.00 0.00 A2 H

ATOM 67 HB2 GLU A 4 4.691 -4.140 -5.648 0.00 0.00 A2 H

ATOM 68 CG GLU A 4 6.120 -5.630 -4.962 1.00 0.00 A2 C

ATOM 69 HG1 GLU A 4 6.286 -6.119 -3.985 0.00 0.00 A2 H

ATOM 70 HG2 GLU A 4 6.275 -6.371 -5.771 0.00 0.00 A2 H

ATOM 71 CD GLU A 4 7.174 -4.556 -5.076 1.00 0.00 A2 C

ATOM 72 OE1 GLU A 4 6.849 -3.420 -5.736 1.00 0.00 A2 O

ATOM 73 OE2 GLU A 4 8.386 -4.777 -4.518 1.00 0.00 A2 O

ATOM 74 C GLU A 4 4.955 -3.704 -2.902 1.00 0.00 A2 C

ATOM 75 O GLU A 4 4.689 -2.499 -2.985 1.00 0.00 A2 O

ATOM 76 N ARG A 5 5.965 -4.165 -2.173 1.00 0.00 A2 N

ATOM 77 HN ARG A 5 6.211 -5.132 -2.170 0.00 0.00 A2 H

ATOM 78 CA ARG A 5 6.774 -3.278 -1.332 1.00 0.00 A2 C

ATOM 79 HA ARG A 5 6.142 -2.950 -0.515 0.00 0.00 A2 H

ATOM 80 CB ARG A 5 7.967 -4.040 -0.735 1.00 0.00 A2 C

ATOM 81 HB1 ARG A 5 7.543 -4.933 -0.216 0.00 0.00 A2 H

ATOM 82 HB2 ARG A 5 8.604 -4.437 -1.556 0.00 0.00 A2 H

ATOM 83 CG ARG A 5 8.792 -3.239 0.268 1.00 0.00 A2 C

ATOM 84 HG1 ARG A 5 9.156 -2.314 -0.231 0.00 0.00 A2 H

ATOM 85 HG2 ARG A 5 8.125 -2.892 1.091 0.00 0.00 A2 H

ATOM 86 CD ARG A 5 9.917 -4.084 0.861 1.00 0.00 A2 C

ATOM 87 HD1 ARG A 5 10.540 -3.451 1.534 0.00 0.00 A2 H

ATOM 88 HD2 ARG A 5 9.517 -4.943 1.448 0.00 0.00 A2 H

ATOM 89 NE ARG A 5 10.787 -4.666 -0.162 1.00 0.00 A2 N

ATOM 90 HE ARG A 5 10.500 -5.587 -0.448 0.00 0.00 A2 H

ATOM 91 CZ ARG A 5 11.851 -4.064 -0.678 1.00 0.00 A2 C

ATOM 92 NH1 ARG A 5 12.221 -2.771 -0.237 1.00 0.00 A2 N

ATOM 93 1HH1 ARG A 5 12.999 -2.306 -0.636 0.00 0.00 A2 H

ATOM 94 2HH1 ARG A 5 11.582 -2.314 0.385 0.00 0.00 A2 H

ATOM 95 NH2 ARG A 5 12.627 -4.714 -1.669 1.00 0.00 A2 N

ATOM 96 1HH2 ARG A 5 13.467 -4.300 -1.988 0.00 0.00 A2 H

ATOM 97 2HH2 ARG A 5 12.410 -5.663 -1.858 0.00 0.00 A2 H

ATOM 98 C ARG A 5 7.255 -2.017 -2.045 1.00 0.00 A2 C

ATOM 99 O ARG A 5 6.946 -0.904 -1.624 1.00 0.00 A2 O

ATOM 100 CB CYS A 6 9.707 -1.409 -4.760 1.00 0.00 A2 C

ATOM 101 SG CYS A 6 11.225 -1.966 -3.884 1.00 0.00 A2 S

ATOM 102 N CYS A 6 8.031 -2.174 -3.113 1.00 0.00 A2 N

ATOM 103 HN CYS A 6 8.276 -3.050 -3.525 0.00 0.00 A2 H

ATOM 104 CA CYS A 6 8.600 -1.003 -3.779 1.00 0.00 A2 C

ATOM 105 HA CYS A 6 9.065 -0.412 -2.999 0.00 0.00 A2 H

ATOM 106 HB1 CYS A 6 9.309 -2.245 -5.379 0.00 0.00 A2 H

ATOM 107 HB2 CYS A 6 9.910 -0.574 -5.466 0.00 0.00 A2 H

ATOM 108 C CYS A 6 7.530 -0.138 -4.443 1.00 0.00 A2 C

ATOM 109 O CYS A 6 7.653 1.089 -4.490 1.00 0.00 A2 O

ATOM 110 N GLU A 7 6.478 -0.776 -4.939 1.00 0.00 A2 N

ATOM 111 HN GLU A 7 6.374 -1.766 -4.967 0.00 0.00 A2 H

ATOM 112 CA GLU A 7 5.349 -0.044 -5.503 1.00 0.00 A2 C

ATOM 113 HA GLU A 7 5.699 0.541 -6.344 0.00 0.00 A2 H

ATOM 114 CB GLU A 7 4.255 -1.013 -5.948 1.00 0.00 A2 C

ATOM 115 HB1 GLU A 7 4.685 -1.691 -6.721 0.00 0.00 A2 H

ATOM 116 HB2 GLU A 7 3.962 -1.668 -5.097 0.00 0.00 A2 H

ATOM 117 CG GLU A 7 3.028 -0.320 -6.519 1.00 0.00 A2 C

ATOM 118 HG1 GLU A 7 2.658 0.430 -5.796 0.00 0.00 A2 H

ATOM 119 HG2 GLU A 7 3.266 0.184 -7.477 0.00 0.00 A2 H

ATOM 120 CD GLU A 7 1.875 -1.277 -6.749 1.00 0.00 A2 C

ATOM 121 OE1 GLU A 7 1.880 -2.465 -6.101 1.00 0.00 A2 O

ATOM 122 OE2 GLU A 7 0.891 -0.902 -7.600 1.00 0.00 A2 O

ATOM 123 C GLU A 7 4.793 0.938 -4.465 1.00 0.00 A2 C

ATOM 124 O GLU A 7 4.537 2.105 -4.765 1.00 0.00 A2 O

ATOM 125 N LEU A 8 4.609 0.457 -3.244 1.00 0.00 A2 N

ATOM 126 HN LEU A 8 4.819 -0.478 -2.967 0.00 0.00 A2 H

ATOM 127 CA LEU A 8 4.065 1.291 -2.176 1.00 0.00 A2 C

ATOM 128 HA LEU A 8 3.169 1.755 -2.568 0.00 0.00 A2 H

ATOM 129 CB LEU A 8 3.745 0.443 -0.939 1.00 0.00 A2 C

ATOM 130 HB1 LEU A 8 3.012 -0.335 -1.254 0.00 0.00 A2 H

ATOM 131 HB2 LEU A 8 4.662 -0.106 -0.630 0.00 0.00 A2 H

ATOM 132 CG LEU A 8 3.157 1.212 0.256 1.00 0.00 A2 C

ATOM 133 HG LEU A 8 3.850 1.869 0.551 0.00 0.00 A2 H

ATOM 134 CD1 LEU A 8 1.891 1.990 -0.142 1.00 0.00 A2 C

ATOM 135 1HD1 LEU A 8 1.453 2.500 0.742 0.00 0.00 A2 H

ATOM 136 2HD1 LEU A 8 2.129 2.760 -0.907 0.00 0.00 A2 H

ATOM 137 3HD1 LEU A 8 1.125 1.304 -0.562 0.00 0.00 A2 H

ATOM 138 CD2 LEU A 8 2.850 0.277 1.414 1.00 0.00 A2 C

ATOM 139 1HD2 LEU A 8 2.444 0.850 2.276 0.00 0.00 A2 H

ATOM 140 2HD2 LEU A 8 2.098 -0.488 1.134 0.00 0.00 A2 H

ATOM 141 3HD2 LEU A 8 3.773 -0.245 1.748 0.00 0.00 A2 H

ATOM 142 C LEU A 8 5.036 2.407 -1.803 1.00 0.00 A2 C

ATOM 143 O LEU A 8 4.626 3.544 -1.554 1.00 0.00 A2 O

ATOM 144 N ALA A 9 6.326 2.078 -1.757 1.00 0.00 A2 N

ATOM 145 HN ALA A 9 6.670 1.165 -1.976 0.00 0.00 A2 H

ATOM 146 CA ALA A 9 7.338 3.052 -1.375 1.00 0.00 A2 C

ATOM 147 HA ALA A 9 7.150 3.442 -0.381 0.00 0.00 A2 H

ATOM 148 CB ALA A 9 8.718 2.424 -1.373 1.00 0.00 A2 C

ATOM 149 HB1 ALA A 9 8.746 1.588 -0.642 0.00 0.00 A2 H

ATOM 150 HB2 ALA A 9 8.971 2.012 -2.374 0.00 0.00 A2 H

ATOM 151 HB3 ALA A 9 9.501 3.158 -1.085 0.00 0.00 A2 H

ATOM 152 C ALA A 9 7.294 4.242 -2.318 1.00 0.00 A2 C

ATOM 153 O ALA A 9 7.272 5.385 -1.881 1.00 0.00 A2 O

ATOM 154 N ARG A 10 7.263 3.968 -3.617 1.00 0.00 A2 N

ATOM 155 HN ARG A 10 7.287 3.033 -3.962 0.00 0.00 A2 H

ATOM 156 CA ARG A 10 7.191 5.036 -4.617 1.00 0.00 A2 C

ATOM 157 HA ARG A 10 8.003 5.725 -4.414 0.00 0.00 A2 H

ATOM 158 CB ARG A 10 7.377 4.452 -6.018 1.00 0.00 A2 C

ATOM 159 HB1 ARG A 10 6.658 3.603 -6.103 0.00 0.00 A2 H

ATOM 160 HB2 ARG A 10 7.060 5.200 -6.778 0.00 0.00 A2 H

ATOM 161 CG ARG A 10 8.796 3.945 -6.262 1.00 0.00 A2 C

ATOM 162 HG1 ARG A 10 9.514 4.711 -5.894 0.00 0.00 A2 H

ATOM 163 HG2 ARG A 10 8.972 3.043 -5.632 0.00 0.00 A2 H

ATOM 164 CD ARG A 10 9.014 3.579 -7.715 1.00 0.00 A2 C

ATOM 165 HD1 ARG A 10 8.554 4.358 -8.366 0.00 0.00 A2 H

ATOM 166 HD2 ARG A 10 10.099 3.504 -7.959 0.00 0.00 A2 H

ATOM 167 NE ARG A 10 8.427 2.290 -8.032 1.00 0.00 A2 N

ATOM 168 HE ARG A 10 7.476 2.357 -8.356 0.00 0.00 A2 H

ATOM 169 CZ ARG A 10 9.072 1.135 -7.910 1.00 0.00 A2 C

ATOM 170 NH1 ARG A 10 10.412 1.108 -7.453 1.00 0.00 A2 N

ATOM 171 1HH1 ARG A 10 10.904 0.251 -7.385 0.00 0.00 A2 H

ATOM 172 2HH1 ARG A 10 10.861 1.997 -7.341 0.00 0.00 A2 H

ATOM 173 NH2 ARG A 10 8.419 -0.077 -8.243 1.00 0.00 A2 N

ATOM 174 1HH2 ARG A 10 8.867 -0.943 -8.071 0.00 0.00 A2 H

ATOM 175 2HH2 ARG A 10 7.450 -0.023 -8.444 0.00 0.00 A2 H

ATOM 176 C ARG A 10 5.895 5.852 -4.543 1.00 0.00 A2 C

ATOM 177 O ARG A 10 5.909 7.071 -4.707 1.00 0.00 A2 O

ATOM 178 N THR A 11 4.779 5.166 -4.321 1.00 0.00 A2 N

ATOM 179 HN THR A 11 4.754 4.171 -4.230 0.00 0.00 A2 H

ATOM 180 CA THR A 11 3.481 5.811 -4.189 1.00 0.00 A2 C

ATOM 181 HA THR A 11 3.362 6.414 -5.079 0.00 0.00 A2 H

ATOM 182 CB THR A 11 2.344 4.769 -4.045 1.00 0.00 A2 C

ATOM 183 HB THR A 11 2.622 4.124 -3.176 0.00 0.00 A2 H

ATOM 184 OG1 THR A 11 2.297 3.938 -5.215 1.00 0.00 A2 O

ATOM 185 HG1 THR A 11 1.568 3.326 -5.066 0.00 0.00 A2 H

ATOM 186 CG2 THR A 11 0.992 5.464 -3.876 1.00 0.00 A2 C

ATOM 187 1HG2 THR A 11 0.192 4.725 -3.659 0.00 0.00 A2 H

ATOM 188 2HG2 THR A 11 1.030 6.184 -3.031 0.00 0.00 A2 H

ATOM 189 3HG2 THR A 11 0.711 6.021 -4.796 0.00 0.00 A2 H

ATOM 190 C THR A 11 3.449 6.777 -3.000 1.00 0.00 A2 C

ATOM 191 O THR A 11 3.010 7.923 -3.138 1.00 0.00 A2 O

ATOM 192 N LEU A 12 3.919 6.316 -1.840 1.00 0.00 A2 N

ATOM 193 HN LEU A 12 4.289 5.399 -1.707 0.00 0.00 A2 H

ATOM 194 CA LEU A 12 3.928 7.147 -0.639 1.00 0.00 A2 C

ATOM 195 HA LEU A 12 2.923 7.529 -0.511 0.00 0.00 A2 H

ATOM 196 CB LEU A 12 4.357 6.331 0.588 1.00 0.00 A2 C

ATOM 197 HB1 LEU A 12 5.303 5.807 0.320 0.00 0.00 A2 H

ATOM 198 HB2 LEU A 12 4.615 7.029 1.415 0.00 0.00 A2 H

ATOM 199 CG LEU A 12 3.348 5.287 1.076 1.00 0.00 A2 C

ATOM 200 HG LEU A 12 3.153 4.664 0.319 0.00 0.00 A2 H

ATOM 201 CD1 LEU A 12 3.928 4.472 2.221 1.00 0.00 A2 C

ATOM 202 1HD1 LEU A 12 3.177 3.748 2.604 0.00 0.00 A2 H

ATOM 203 2HD1 LEU A 12 4.820 3.903 1.883 0.00 0.00 A2 H

ATOM 204 3HD1 LEU A 12 4.231 5.134 3.060 0.00 0.00 A2 H

ATOM 205 CD2 LEU A 12 2.028 5.956 1.493 1.00 0.00 A2 C

ATOM 206 1HD2 LEU A 12 1.296 5.190 1.828 0.00 0.00 A2 H

ATOM 207 2HD2 LEU A 12 2.176 6.668 2.330 0.00 0.00 A2 H

ATOM 208 3HD2 LEU A 12 1.585 6.509 0.637 0.00 0.00 A2 H

ATOM 209 C LEU A 12 4.855 8.343 -0.822 1.00 0.00 A2 C

ATOM 210 O LEU A 12 4.550 9.466 -0.406 1.00 0.00 A2 O

ATOM 211 N LYS A 13 5.995 8.098 -1.452 1.00 0.00 A2 N

ATOM 212 HN LYS A 13 6.295 7.193 -1.750 0.00 0.00 A2 H

ATOM 213 CA LYS A 13 6.912 9.184 -1.757 1.00 0.00 A2 C

ATOM 214 HA LYS A 13 7.153 9.680 -0.824 0.00 0.00 A2 H

ATOM 215 CB LYS A 13 8.194 8.653 -2.413 1.00 0.00 A2 C

ATOM 216 HB1 LYS A 13 8.593 7.850 -1.751 0.00 0.00 A2 H

ATOM 217 HB2 LYS A 13 7.931 8.162 -3.377 0.00 0.00 A2 H

ATOM 218 CG LYS A 13 9.254 9.742 -2.605 1.00 0.00 A2 C

ATOM 219 HG1 LYS A 13 8.890 10.476 -3.360 0.00 0.00 A2 H

ATOM 220 HG2 LYS A 13 9.387 10.292 -1.645 0.00 0.00 A2 H

ATOM 221 CD LYS A 13 10.578 9.161 -3.073 1.00 0.00 A2 C

ATOM 222 HD1 LYS A 13 10.863 8.334 -2.383 0.00 0.00 A2 H

ATOM 223 HD2 LYS A 13 10.423 8.715 -4.083 0.00 0.00 A2 H

ATOM 224 CE LYS A 13 11.668 10.232 -3.121 1.00 0.00 A2 C

ATOM 225 HE1 LYS A 13 12.625 9.798 -3.486 0.00 0.00 A2 H

ATOM 226 HE2 LYS A 13 11.374 11.059 -3.805 0.00 0.00 A2 H

ATOM 227 NZ LYS A 13 11.921 10.822 -1.777 1.00 0.00 A2 N

ATOM 228 HZ1 LYS A 13 12.667 11.545 -1.840 0.00 0.00 A2 H

ATOM 229 HZ2 LYS A 13 11.047 11.257 -1.419 0.00 0.00 A2 H

ATOM 230 HZ3 LYS A 13 12.223 10.073 -1.122 0.00 0.00 A2 H

ATOM 231 C LYS A 13 6.232 10.212 -2.656 1.00 0.00 A2 C

ATOM 232 O LYS A 13 6.322 11.411 -2.425 1.00 0.00 A2 O

ATOM 233 N ARG A 14 5.542 9.735 -3.684 1.00 0.00 A2 N

ATOM 234 HN ARG A 14 5.461 8.757 -3.860 0.00 0.00 A2 H

ATOM 235 CA ARG A 14 4.860 10.627 -4.618 1.00 0.00 A2 C

ATOM 236 HA ARG A 14 5.584 11.329 -5.015 0.00 0.00 A2 H

ATOM 237 CB ARG A 14 4.229 9.817 -5.766 1.00 0.00 A2 C

ATOM 238 HB1 ARG A 14 5.064 9.542 -6.454 0.00 0.00 A2 H

ATOM 239 HB2 ARG A 14 3.831 8.857 -5.369 0.00 0.00 A2 H

ATOM 240 CG ARG A 14 3.167 10.577 -6.560 1.00 0.00 A2 C

ATOM 241 HG1 ARG A 14 2.343 10.859 -5.869 0.00 0.00 A2 H

ATOM 242 HG2 ARG A 14 3.601 11.539 -6.917 0.00 0.00 A2 H

ATOM 243 CD ARG A 14 2.671 9.779 -7.781 1.00 0.00 A2 C

ATOM 244 HD1 ARG A 14 1.935 10.392 -8.350 0.00 0.00 A2 H

ATOM 245 HD2 ARG A 14 3.512 9.507 -8.461 0.00 0.00 A2 H

ATOM 246 NE ARG A 14 2.031 8.517 -7.408 1.00 0.00 A2 N

ATOM 247 HE ARG A 14 2.631 7.718 -7.531 0.00 0.00 A2 H

ATOM 248 CZ ARG A 14 0.784 8.414 -6.951 1.00 0.00 A2 C

ATOM 249 NH1 ARG A 14 -0.015 9.572 -6.794 1.00 0.00 A2 N

ATOM 250 1HH1 ARG A 14 -0.934 9.505 -6.431 0.00 0.00 A2 H

ATOM 251 2HH1 ARG A 14 0.444 10.451 -6.937 0.00 0.00 A2 H

ATOM 252 NH2 ARG A 14 0.250 7.144 -6.624 1.00 0.00 A2 N

ATOM 253 1HH2 ARG A 14 -0.703 7.060 -6.368 0.00 0.00 A2 H

ATOM 254 2HH2 ARG A 14 0.796 6.352 -6.866 0.00 0.00 A2 H

ATOM 255 C ARG A 14 3.804 11.450 -3.906 1.00 0.00 A2 C

ATOM 256 O ARG A 14 3.545 12.602 -4.279 1.00 0.00 A2 O

ATOM 257 N LEU A 15 3.198 10.850 -2.882 1.00 0.00 A2 N

ATOM 258 HN LEU A 15 3.448 9.938 -2.564 0.00 0.00 A2 H

ATOM 259 CA LEU A 15 2.106 11.475 -2.126 1.00 0.00 A2 C

ATOM 260 HA LEU A 15 1.630 12.153 -2.823 0.00 0.00 A2 H

ATOM 261 CB LEU A 15 1.076 10.404 -1.709 1.00 0.00 A2 C

ATOM 262 HB1 LEU A 15 1.640 9.580 -1.215 0.00 0.00 A2 H

ATOM 263 HB2 LEU A 15 0.407 10.830 -0.929 0.00 0.00 A2 H

ATOM 264 CG LEU A 15 0.279 9.849 -2.899 1.00 0.00 A2 C

ATOM 265 HG LEU A 15 0.934 9.506 -3.572 0.00 0.00 A2 H

ATOM 266 CD1 LEU A 15 -0.613 8.678 -2.490 1.00 0.00 A2 C

ATOM 267 1HD1 LEU A 15 -1.214 8.325 -3.355 0.00 0.00 A2 H

ATOM 268 2HD1 LEU A 15 0.001 7.828 -2.122 0.00 0.00 A2 H

ATOM 269 3HD1 LEU A 15 -1.313 8.981 -1.682 0.00 0.00 A2 H

ATOM 270 CD2 LEU A 15 -0.562 10.961 -3.525 1.00 0.00 A2 C

ATOM 271 1HD2 LEU A 15 -1.128 10.572 -4.399 0.00 0.00 A2 H

ATOM 272 2HD2 LEU A 15 -1.297 11.374 -2.805 0.00 0.00 A2 H

ATOM 273 3HD2 LEU A 15 0.089 11.791 -3.875 0.00 0.00 A2 H

ATOM 274 C LEU A 15 2.565 12.304 -0.911 1.00 0.00 A2 C

ATOM 275 O LEU A 15 1.765 12.622 -0.035 1.00 0.00 A2 O

ATOM 276 N GLY A 16 3.853 12.644 -0.867 1.00 0.00 A2 N

ATOM 277 HN GLY A 16 4.520 12.254 -1.500 0.00 0.00 A2 H

ATOM 278 CA GLY A 16 4.382 13.599 0.095 1.00 0.00 A2 C

ATOM 279 HA1 GLY A 16 3.637 14.380 0.163 0.00 0.00 A2 H

ATOM 280 HA2 GLY A 16 5.331 13.924 -0.309 0.00 0.00 A2 H

ATOM 281 C GLY A 16 4.628 13.060 1.495 1.00 0.00 A2 C

ATOM 282 O GLY A 16 4.658 13.826 2.460 1.00 0.00 A2 O

ATOM 283 N MET A 17 4.825 11.752 1.621 1.00 0.00 A2 N

ATOM 284 HN MET A 17 4.871 11.106 0.862 0.00 0.00 A2 H

ATOM 285 CA MET A 17 4.995 11.144 2.944 1.00 0.00 A2 C

ATOM 286 HA MET A 17 4.410 11.763 3.614 0.00 0.00 A2 H

ATOM 287 CB MET A 17 4.410 9.731 2.957 1.00 0.00 A2 C

ATOM 288 HB1 MET A 17 4.816 9.204 2.062 0.00 0.00 A2 H

ATOM 289 HB2 MET A 17 4.794 9.180 3.844 0.00 0.00 A2 H

ATOM 290 CG MET A 17 2.893 9.707 2.909 1.00 0.00 A2 C

ATOM 291 HG1 MET A 17 2.524 10.330 2.067 0.00 0.00 A2 H

ATOM 292 HG2 MET A 17 2.541 8.666 2.738 0.00 0.00 A2 H

ATOM 293 SD MET A 17 2.151 10.332 4.432 1.00 0.00 A2 S

ATOM 294 CE MET A 17 1.477 8.801 5.082 1.00 0.00 A2 C

ATOM 295 HE1 MET A 17 0.952 8.976 6.045 0.00 0.00 A2 H

ATOM 296 HE2 MET A 17 0.748 8.353 4.372 0.00 0.00 A2 H

ATOM 297 HE3 MET A 17 2.281 8.055 5.263 0.00 0.00 A2 H

ATOM 298 C MET A 17 6.431 11.108 3.482 1.00 0.00 A2 C

ATOM 299 O MET A 17 6.643 11.042 4.686 1.00 0.00 A2 O

ATOM 300 N ASP A 18 7.415 11.151 2.593 1.00 0.00 A2 N

ATOM 301 HN ASP A 18 7.319 11.322 1.616 0.00 0.00 A2 H

ATOM 302 CA ASP A 18 8.793 10.940 2.998 1.00 0.00 A2 C

ATOM 303 HA ASP A 18 8.807 10.061 3.632 0.00 0.00 A2 H

ATOM 304 CB ASP A 18 9.666 10.760 1.767 1.00 0.00 A2 C

ATOM 305 HB1 ASP A 18 9.247 9.950 1.137 0.00 0.00 A2 H

ATOM 306 HB2 ASP A 18 9.691 11.691 1.166 0.00 0.00 A2 H

ATOM 307 CG ASP A 18 11.092 10.373 2.112 1.00 0.00 A2 C

ATOM 308 OD1 ASP A 18 11.383 9.933 3.362 1.00 0.00 A2 O

ATOM 309 OD2 ASP A 18 12.017 10.489 1.126 1.00 0.00 A2 O

ATOM 310 C ASP A 18 9.304 12.110 3.851 1.00 0.00 A2 C

ATOM 311 O ASP A 18 9.590 13.180 3.332 1.00 0.00 A2 O

ATOM 312 N GLY A 19 9.389 11.901 5.160 1.00 0.00 A2 N

ATOM 313 HN GLY A 19 9.172 11.022 5.582 0.00 0.00 A2 H

ATOM 314 CA GLY A 19 9.809 12.951 6.074 1.00 0.00 A2 C

ATOM 315 HA1 GLY A 19 10.464 13.599 5.509 0.00 0.00 A2 H

ATOM 316 HA2 GLY A 19 10.286 12.457 6.910 0.00 0.00 A2 H

ATOM 317 C GLY A 19 8.668 13.793 6.616 1.00 0.00 A2 C

ATOM 318 O GLY A 19 8.907 14.778 7.318 1.00 0.00 A2 O

ATOM 319 N TYR A 20 7.428 13.420 6.300 1.00 0.00 A2 N

ATOM 320 HN TYR A 20 7.192 12.637 5.726 0.00 0.00 A2 H

ATOM 321 CA TYR A 20 6.276 14.176 6.802 1.00 0.00 A2 C

ATOM 322 HA TYR A 20 6.414 15.212 6.518 0.00 0.00 A2 H

ATOM 323 CB TYR A 20 4.950 13.632 6.255 1.00 0.00 A2 C

ATOM 324 HB1 TYR A 20 5.002 13.596 5.145 0.00 0.00 A2 H

ATOM 325 HB2 TYR A 20 4.775 12.593 6.610 0.00 0.00 A2 H

ATOM 326 CG TYR A 20 3.775 14.512 6.625 1.00 0.00 A2 C

ATOM 327 CD1 TYR A 20 3.437 15.618 5.847 1.00 0.00 A2 C

ATOM 328 HD1 TYR A 20 4.019 15.854 4.967 0.00 0.00 A2 H

ATOM 329 CE1 TYR A 20 2.369 16.442 6.199 1.00 0.00 A2 C

ATOM 330 HE1 TYR A 20 2.108 17.294 5.589 0.00 0.00 A2 H

ATOM 331 CZ TYR A 20 1.641 16.167 7.347 1.00 0.00 A2 C

ATOM 332 OH TYR A 20 0.584 16.971 7.713 1.00 0.00 A2 O

ATOM 333 HH TYR A 20 0.174 16.574 8.484 0.00 0.00 A2 H

ATOM 334 CD2 TYR A 20 3.031 14.265 7.770 1.00 0.00 A2 C

ATOM 335 HD2 TYR A 20 3.301 13.447 8.424 0.00 0.00 A2 H

ATOM 336 CE2 TYR A 20 1.963 15.082 8.131 1.00 0.00 A2 C

ATOM 337 HE2 TYR A 20 1.409 14.872 9.033 0.00 0.00 A2 H

ATOM 338 C TYR A 20 6.284 14.123 8.323 1.00 0.00 A2 C

ATOM 339 O TYR A 20 6.315 13.040 8.899 1.00 0.00 A2 O

ATOM 340 N ARG A 21 6.292 15.288 8.974 1.00 0.00 A2 N

ATOM 341 HN ARG A 21 6.219 16.160 8.496 0.00 0.00 A2 H

ATOM 342 CA ARG A 21 6.409 15.346 10.436 1.00 0.00 A2 C

ATOM 343 HA ARG A 21 6.538 16.386 10.710 0.00 0.00 A2 H

ATOM 344 CB ARG A 21 5.134 14.804 11.087 1.00 0.00 A2 C

ATOM 345 HB1 ARG A 21 4.858 13.883 10.520 0.00 0.00 A2 H

ATOM 346 HB2 ARG A 21 5.359 14.470 12.124 0.00 0.00 A2 H

ATOM 347 CG ARG A 21 3.986 15.796 11.036 1.00 0.00 A2 C

ATOM 348 HG1 ARG A 21 3.696 15.947 9.973 0.00 0.00 A2 H

ATOM 349 HG2 ARG A 21 3.094 15.343 11.528 0.00 0.00 A2 H

ATOM 350 CD ARG A 21 4.407 17.058 11.762 1.00 0.00 A2 C

ATOM 351 HD1 ARG A 21 5.354 17.442 11.318 0.00 0.00 A2 H

ATOM 352 HD2 ARG A 21 3.629 17.853 11.686 0.00 0.00 A2 H

ATOM 353 NE ARG A 21 4.607 16.782 13.186 1.00 0.00 A2 N

ATOM 354 HE ARG A 21 5.513 16.393 13.390 0.00 0.00 A2 H

ATOM 355 CZ ARG A 21 3.677 17.025 14.113 1.00 0.00 A2 C

ATOM 356 NH1 ARG A 21 2.439 17.598 13.735 1.00 0.00 A2 N

ATOM 357 1HH1 ARG A 21 1.751 17.806 14.416 0.00 0.00 A2 H

ATOM 358 2HH1 ARG A 21 2.362 17.899 12.782 0.00 0.00 A2 H

ATOM 359 NH2 ARG A 21 3.913 16.725 15.476 1.00 0.00 A2 N

ATOM 360 1HH2 ARG A 21 3.184 16.829 16.138 0.00 0.00 A2 H

ATOM 361 2HH2 ARG A 21 4.740 16.221 15.687 0.00 0.00 A2 H

ATOM 362 C ARG A 21 7.635 14.594 10.977 1.00 0.00 A2 C

ATOM 363 O ARG A 21 7.573 13.965 12.040 1.00 0.00 A2 O

ATOM 364 N GLY A 22 8.738 14.629 10.241 1.00 0.00 A2 N

ATOM 365 HN GLY A 22 8.805 15.123 9.375 0.00 0.00 A2 H

ATOM 366 CA GLY A 22 9.947 13.931 10.664 1.00 0.00 A2 C

ATOM 367 HA1 GLY A 22 10.089 14.199 11.702 0.00 0.00 A2 H

ATOM 368 HA2 GLY A 22 10.733 14.275 10.005 0.00 0.00 A2 H

ATOM 369 C GLY A 22 9.889 12.419 10.573 1.00 0.00 A2 C

ATOM 370 O GLY A 22 10.737 11.721 11.129 1.00 0.00 A2 O

ATOM 371 N ILE A 23 8.890 11.900 9.866 1.00 0.00 A2 N

ATOM 372 HN ILE A 23 8.218 12.442 9.366 0.00 0.00 A2 H

ATOM 373 CA ILE A 23 8.691 10.460 9.763 1.00 0.00 A2 C

ATOM 374 HA ILE A 23 9.321 10.001 10.514 0.00 0.00 A2 H

ATOM 375 CB ILE A 23 7.202 10.089 9.905 1.00 0.00 A2 C

ATOM 376 HB ILE A 23 6.686 10.699 9.121 0.00 0.00 A2 H

ATOM 377 CG2 ILE A 23 6.984 8.574 9.739 1.00 0.00 A2 C

ATOM 378 1HG2 ILE A 23 5.901 8.339 9.687 0.00 0.00 A2 H

ATOM 379 2HG2 ILE A 23 7.453 8.207 8.802 0.00 0.00 A2 H

ATOM 380 3HG2 ILE A 23 7.420 8.014 10.593 0.00 0.00 A2 H

ATOM 381 CG1 ILE A 23 6.663 10.555 11.253 1.00 0.00 A2 C

ATOM 382 1HG1 ILE A 23 7.081 9.920 12.066 0.00 0.00 A2 H

ATOM 383 2HG1 ILE A 23 7.009 11.597 11.439 0.00 0.00 A2 H

ATOM 384 CD ILE A 23 5.154 10.526 11.309 1.00 0.00 A2 C

ATOM 385 HD1 ILE A 23 4.796 10.926 12.282 0.00 0.00 A2 H

ATOM 386 HD2 ILE A 23 4.720 11.148 10.497 0.00 0.00 A2 H

ATOM 387 HD3 ILE A 23 4.771 9.489 11.202 0.00 0.00 A2 H

ATOM 388 C ILE A 23 9.185 9.962 8.409 1.00 0.00 A2 C

ATOM 389 O ILE A 23 8.650 10.336 7.356 1.00 0.00 A2 O

ATOM 390 N SER A 24 10.198 9.105 8.442 1.00 0.00 A2 N

ATOM 391 HN SER A 24 10.600 8.733 9.279 0.00 0.00 A2 H

ATOM 392 CA SER A 24 10.820 8.627 7.221 1.00 0.00 A2 C

ATOM 393 HA SER A 24 11.007 9.507 6.619 0.00 0.00 A2 H

ATOM 394 CB SER A 24 12.086 7.846 7.542 1.00 0.00 A2 C

ATOM 395 HB1 SER A 24 12.651 7.698 6.594 0.00 0.00 A2 H

ATOM 396 HB2 SER A 24 12.741 8.433 8.225 0.00 0.00 A2 H

ATOM 397 OG SER A 24 11.744 6.588 8.088 1.00 0.00 A2 O

ATOM 398 HG1 SER A 24 12.542 6.044 8.069 0.00 0.00 A2 H

ATOM 399 C SER A 24 9.880 7.730 6.431 1.00 0.00 A2 C

ATOM 400 O SER A 24 8.959 7.122 6.984 1.00 0.00 A2 O

ATOM 401 N LEU A 25 10.132 7.640 5.132 1.00 0.00 A2 N

ATOM 402 HN LEU A 25 10.842 8.161 4.662 0.00 0.00 A2 H

ATOM 403 CA LEU A 25 9.369 6.746 4.263 1.00 0.00 A2 C

ATOM 404 HA LEU A 25 8.335 7.063 4.310 0.00 0.00 A2 H

ATOM 405 CB LEU A 25 9.892 6.830 2.820 1.00 0.00 A2 C

ATOM 406 HB1 LEU A 25 9.887 7.906 2.530 0.00 0.00 A2 H

ATOM 407 HB2 LEU A 25 10.958 6.512 2.802 0.00 0.00 A2 H

ATOM 408 CG LEU A 25 9.165 6.084 1.704 1.00 0.00 A2 C

ATOM 409 HG LEU A 25 9.154 5.108 1.920 0.00 0.00 A2 H

ATOM 410 CD1 LEU A 25 7.738 6.573 1.588 1.00 0.00 A2 C

ATOM 411 1HD1 LEU A 25 7.220 6.067 0.746 0.00 0.00 A2 H

ATOM 412 2HD1 LEU A 25 7.175 6.361 2.522 0.00 0.00 A2 H

ATOM 413 3HD1 LEU A 25 7.712 7.668 1.403 0.00 0.00 A2 H

ATOM 414 CD2 LEU A 25 9.897 6.306 0.378 1.00 0.00 A2 C

ATOM 415 1HD2 LEU A 25 9.389 5.753 -0.441 0.00 0.00 A2 H

ATOM 416 2HD2 LEU A 25 9.919 7.378 0.097 0.00 0.00 A2 H

ATOM 417 3HD2 LEU A 25 10.945 5.941 0.446 0.00 0.00 A2 H

ATOM 418 C LEU A 25 9.436 5.314 4.786 1.00 0.00 A2 C

ATOM 419 O LEU A 25 8.437 4.605 4.781 1.00 0.00 A2 O

ATOM 420 N ALA A 26 10.605 4.886 5.263 1.00 0.00 A2 N

ATOM 421 HN ALA A 26 11.436 5.439 5.312 0.00 0.00 A2 H

ATOM 422 CA ALA A 26 10.732 3.511 5.764 1.00 0.00 A2 C

ATOM 423 HA ALA A 26 10.457 2.803 4.991 0.00 0.00 A2 H

ATOM 424 CB ALA A 26 12.184 3.197 6.151 1.00 0.00 A2 C

ATOM 425 HB1 ALA A 26 12.845 3.346 5.271 0.00 0.00 A2 H

ATOM 426 HB2 ALA A 26 12.536 3.872 6.961 0.00 0.00 A2 H

ATOM 427 HB3 ALA A 26 12.298 2.145 6.492 0.00 0.00 A2 H

ATOM 428 C ALA A 26 9.799 3.247 6.941 1.00 0.00 A2 C

ATOM 429 O ALA A 26 9.292 2.139 7.099 1.00 0.00 A2 O

ATOM 430 N ASN A 27 9.605 4.260 7.782 1.00 0.00 A2 N

ATOM 431 HN ASN A 27 10.048 5.151 7.688 0.00 0.00 A2 H

ATOM 432 CA ASN A 27 8.713 4.147 8.929 1.00 0.00 A2 C

ATOM 433 HA ASN A 27 9.014 3.231 9.426 0.00 0.00 A2 H

ATOM 434 CB ASN A 27 8.900 5.350 9.861 1.00 0.00 A2 C

ATOM 435 HB1 ASN A 27 9.173 6.227 9.234 0.00 0.00 A2 H

ATOM 436 HB2 ASN A 27 7.947 5.592 10.374 0.00 0.00 A2 H

ATOM 437 CG ASN A 27 10.007 5.124 10.872 1.00 0.00 A2 C

ATOM 438 OD1 ASN A 27 9.864 4.313 11.788 1.00 0.00 A2 O

ATOM 439 ND2 ASN A 27 11.122 5.820 10.700 1.00 0.00 A2 N

ATOM 440 1HD2 ASN A 27 11.860 5.671 11.352 0.00 0.00 A2 H

ATOM 441 2HD2 ASN A 27 11.209 6.439 9.926 0.00 0.00 A2 H

ATOM 442 C ASN A 27 7.245 4.002 8.543 1.00 0.00 A2 C

ATOM 443 O ASN A 27 6.495 3.247 9.157 1.00 0.00 A2 O

ATOM 444 N TRP A 28 6.828 4.759 7.538 1.00 0.00 A2 N

ATOM 445 HN TRP A 28 7.402 5.437 7.084 0.00 0.00 A2 H

ATOM 446 CA TRP A 28 5.477 4.651 7.018 1.00 0.00 A2 C

ATOM 447 HA TRP A 28 4.780 4.752 7.841 0.00 0.00 A2 H

ATOM 448 CB TRP A 28 5.272 5.699 5.929 1.00 0.00 A2 C

ATOM 449 HB1 TRP A 28 6.182 5.707 5.288 0.00 0.00 A2 H

ATOM 450 HB2 TRP A 28 4.421 5.405 5.277 0.00 0.00 A2 H

ATOM 451 CG TRP A 28 5.072 7.124 6.419 1.00 0.00 A2 C

ATOM 452 CD1 TRP A 28 5.871 8.195 6.159 1.00 0.00 A2 C

ATOM 453 HD1 TRP A 28 6.768 8.159 5.556 0.00 0.00 A2 H

ATOM 454 NE1 TRP A 28 5.343 9.330 6.742 1.00 0.00 A2 N

ATOM 455 HE1 TRP A 28 5.709 10.233 6.680 0.00 0.00 A2 H

ATOM 456 CE2 TRP A 28 4.181 9.003 7.391 1.00 0.00 A2 C

ATOM 457 CD2 TRP A 28 3.977 7.624 7.218 1.00 0.00 A2 C

ATOM 458 CE3 TRP A 28 2.845 7.031 7.794 1.00 0.00 A2 C

ATOM 459 HE3 TRP A 28 2.639 5.974 7.690 0.00 0.00 A2 H

ATOM 460 CZ3 TRP A 28 1.973 7.828 8.523 1.00 0.00 A2 C

ATOM 461 HZ3 TRP A 28 1.099 7.382 8.977 0.00 0.00 A2 H

ATOM 462 CZ2 TRP A 28 3.304 9.806 8.137 1.00 0.00 A2 C

ATOM 463 HZ2 TRP A 28 3.467 10.862 8.284 0.00 0.00 A2 H

ATOM 464 CH2 TRP A 28 2.207 9.199 8.685 1.00 0.00 A2 C

ATOM 465 HH2 TRP A 28 1.509 9.796 9.256 0.00 0.00 A2 H

ATOM 466 C TRP A 28 5.288 3.251 6.446 1.00 0.00 A2 C

ATOM 467 O TRP A 28 4.233 2.626 6.613 1.00 0.00 A2 O

ATOM 468 N MET A 29 6.330 2.753 5.783 1.00 0.00 A2 N

ATOM 469 HN MET A 29 7.185 3.248 5.644 0.00 0.00 A2 H

ATOM 470 CA MET A 29 6.305 1.418 5.195 1.00 0.00 A2 C

ATOM 471 HA MET A 29 5.434 1.366 4.553 0.00 0.00 A2 H

ATOM 472 CB MET A 29 7.578 1.157 4.393 1.00 0.00 A2 C

ATOM 473 HB1 MET A 29 8.436 1.443 5.045 0.00 0.00 A2 H

ATOM 474 HB2 MET A 29 7.679 0.065 4.206 0.00 0.00 A2 H

ATOM 475 CG MET A 29 7.665 1.937 3.092 1.00 0.00 A2 C

ATOM 476 HG1 MET A 29 7.441 3.010 3.270 0.00 0.00 A2 H

ATOM 477 HG2 MET A 29 8.698 1.872 2.686 0.00 0.00 A2 H

ATOM 478 SD MET A 29 6.505 1.316 1.859 1.00 0.00 A2 S

ATOM 479 CE MET A 29 7.182 -0.329 1.562 1.00 0.00 A2 C

ATOM 480 HE1 MET A 29 6.584 -0.872 0.799 0.00 0.00 A2 H

ATOM 481 HE2 MET A 29 8.230 -0.269 1.196 0.00 0.00 A2 H

ATOM 482 HE3 MET A 29 7.180 -0.936 2.493 0.00 0.00 A2 H

ATOM 483 C MET A 29 6.147 0.358 6.266 1.00 0.00 A2 C

ATOM 484 O MET A 29 5.321 -0.543 6.135 1.00 0.00 A2 O

ATOM 485 CB CYS A 30 7.935 -0.084 9.511 1.00 0.00 A2 C

ATOM 486 SG CYS A 30 8.019 -1.293 10.852 1.00 0.00 A2 S

ATOM 487 N CYS A 30 6.954 0.466 7.319 1.00 0.00 A2 N

ATOM 488 HN CYS A 30 7.648 1.179 7.404 0.00 0.00 A2 H

ATOM 489 CA CYS A 30 6.887 -0.461 8.446 1.00 0.00 A2 C

ATOM 490 HA CYS A 30 7.052 -1.455 8.047 0.00 0.00 A2 H

ATOM 491 HB1 CYS A 30 8.923 -0.037 9.000 0.00 0.00 A2 H

ATOM 492 HB2 CYS A 30 7.736 0.943 9.889 0.00 0.00 A2 H

ATOM 493 C CYS A 30 5.494 -0.435 9.068 1.00 0.00 A2 C

ATOM 494 O CYS A 30 4.952 -1.473 9.451 1.00 0.00 A2 O

ATOM 495 N LEU A 31 4.917 0.760 9.178 1.00 0.00 A2 N

ATOM 496 HN LEU A 31 5.354 1.619 8.921 0.00 0.00 A2 H

ATOM 497 CA LEU A 31 3.561 0.909 9.699 1.00 0.00 A2 C

ATOM 498 HA LEU A 31 3.548 0.434 10.672 0.00 0.00 A2 H

ATOM 499 CB LEU A 31 3.189 2.393 9.826 1.00 0.00 A2 C

ATOM 500 HB1 LEU A 31 3.847 2.831 10.612 0.00 0.00 A2 H

ATOM 501 HB2 LEU A 31 3.452 2.914 8.879 0.00 0.00 A2 H

ATOM 502 CG LEU A 31 1.740 2.736 10.198 1.00 0.00 A2 C

ATOM 503 HG LEU A 31 1.140 2.230 9.579 0.00 0.00 A2 H

ATOM 504 CD1 LEU A 31 1.404 2.299 11.633 1.00 0.00 A2 C

ATOM 505 1HD1 LEU A 31 0.368 2.599 11.899 0.00 0.00 A2 H

ATOM 506 2HD1 LEU A 31 1.485 1.196 11.735 0.00 0.00 A2 H

ATOM 507 3HD1 LEU A 31 2.099 2.771 12.360 0.00 0.00 A2 H

ATOM 508 CD2 LEU A 31 1.488 4.222 10.025 1.00 0.00 A2 C

ATOM 509 1HD2 LEU A 31 0.433 4.467 10.277 0.00 0.00 A2 H

ATOM 510 2HD2 LEU A 31 2.141 4.828 10.685 0.00 0.00 A2 H

ATOM 511 3HD2 LEU A 31 1.674 4.528 8.973 0.00 0.00 A2 H

ATOM 512 C LEU A 31 2.540 0.180 8.814 1.00 0.00 A2 C

ATOM 513 O LEU A 31 1.688 -0.539 9.313 1.00 0.00 A2 O

ATOM 514 N ALA A 32 2.630 0.364 7.499 1.00 0.00 A2 N

ATOM 515 HN ALA A 32 3.330 0.928 7.062 0.00 0.00 A2 H

ATOM 516 CA ALA A 32 1.677 -0.270 6.590 1.00 0.00 A2 C

ATOM 517 HA ALA A 32 0.659 -0.048 6.889 0.00 0.00 A2 H

ATOM 518 CB ALA A 32 1.873 0.229 5.183 1.00 0.00 A2 C

ATOM 519 HB1 ALA A 32 1.724 1.330 5.154 0.00 0.00 A2 H

ATOM 520 HB2 ALA A 32 2.903 0.013 4.823 0.00 0.00 A2 H

ATOM 521 HB3 ALA A 32 1.148 -0.234 4.480 0.00 0.00 A2 H

ATOM 522 C ALA A 32 1.813 -1.789 6.622 1.00 0.00 A2 C

ATOM 523 O ALA A 32 0.819 -2.519 6.549 1.00 0.00 A2 O

ATOM 524 N LYS A 33 3.055 -2.256 6.715 1.00 0.00 A2 N

ATOM 525 HN LYS A 33 3.862 -1.673 6.798 0.00 0.00 A2 H

ATOM 526 CA LYS A 33 3.325 -3.688 6.701 1.00 0.00 A2 C

ATOM 527 HA LYS A 33 2.873 -4.098 5.806 0.00 0.00 A2 H

ATOM 528 CB LYS A 33 4.840 -3.941 6.723 1.00 0.00 A2 C

ATOM 529 HB1 LYS A 33 5.259 -3.506 5.786 0.00 0.00 A2 H

ATOM 530 HB2 LYS A 33 5.291 -3.367 7.563 0.00 0.00 A2 H

ATOM 531 CG LYS A 33 5.223 -5.417 6.805 1.00 0.00 A2 C

ATOM 532 HG1 LYS A 33 6.314 -5.499 7.014 0.00 0.00 A2 H

ATOM 533 HG2 LYS A 33 4.685 -5.884 7.661 0.00 0.00 A2 H

ATOM 534 CD LYS A 33 4.902 -6.140 5.510 1.00 0.00 A2 C

ATOM 535 HD1 LYS A 33 3.800 -6.102 5.350 0.00 0.00 A2 H

ATOM 536 HD2 LYS A 33 5.380 -5.581 4.673 0.00 0.00 A2 H

ATOM 537 CE LYS A 33 5.395 -7.595 5.526 1.00 0.00 A2 C

ATOM 538 HE1 LYS A 33 6.507 -7.630 5.527 0.00 0.00 A2 H

ATOM 539 HE2 LYS A 33 5.026 -8.123 6.433 0.00 0.00 A2 H

ATOM 540 NZ LYS A 33 4.916 -8.347 4.333 1.00 0.00 A2 N

ATOM 541 HZ1 LYS A 33 5.264 -9.327 4.367 0.00 0.00 A2 H

ATOM 542 HZ2 LYS A 33 3.876 -8.350 4.319 0.00 0.00 A2 H

ATOM 543 HZ3 LYS A 33 5.268 -7.886 3.470 0.00 0.00 A2 H

ATOM 544 C LYS A 33 2.661 -4.355 7.889 1.00 0.00 A2 C

ATOM 545 O LYS A 33 1.936 -5.332 7.748 1.00 0.00 A2 O

ATOM 546 N TRP A 34 2.896 -3.807 9.070 1.00 0.00 A2 N

ATOM 547 HN TRP A 34 3.416 -2.966 9.195 0.00 0.00 A2 H

ATOM 548 CA TRP A 34 2.398 -4.411 10.296 1.00 0.00 A2 C

ATOM 549 HA TRP A 34 2.552 -5.478 10.190 0.00 0.00 A2 H

ATOM 550 CB TRP A 34 3.225 -3.925 11.486 1.00 0.00 A2 C

ATOM 551 HB1 TRP A 34 3.371 -2.828 11.374 0.00 0.00 A2 H

ATOM 552 HB2 TRP A 34 2.657 -4.080 12.429 0.00 0.00 A2 H

ATOM 553 CG TRP A 34 4.553 -4.586 11.513 1.00 0.00 A2 C

ATOM 554 CD1 TRP A 34 5.736 -4.088 11.047 1.00 0.00 A2 C

ATOM 555 HD1 TRP A 34 5.860 -3.098 10.628 0.00 0.00 A2 H

ATOM 556 NE1 TRP A 34 6.737 -5.005 11.228 1.00 0.00 A2 N

ATOM 557 HE1 TRP A 34 7.683 -4.882 11.020 0.00 0.00 A2 H

ATOM 558 CE2 TRP A 34 6.205 -6.130 11.805 1.00 0.00 A2 C

ATOM 559 CD2 TRP A 34 4.830 -5.898 11.993 1.00 0.00 A2 C

ATOM 560 CE3 TRP A 34 4.043 -6.907 12.567 1.00 0.00 A2 C

ATOM 561 HE3 TRP A 34 2.979 -6.785 12.717 0.00 0.00 A2 H

ATOM 562 CZ3 TRP A 34 4.656 -8.094 12.942 1.00 0.00 A2 C

ATOM 563 HZ3 TRP A 34 4.063 -8.881 13.386 0.00 0.00 A2 H

ATOM 564 CZ2 TRP A 34 6.818 -7.326 12.184 1.00 0.00 A2 C

ATOM 565 HZ2 TRP A 34 7.873 -7.503 12.045 0.00 0.00 A2 H

ATOM 566 CH2 TRP A 34 6.031 -8.289 12.751 1.00 0.00 A2 C

ATOM 567 HH2 TRP A 34 6.485 -9.221 13.056 0.00 0.00 A2 H

ATOM 568 C TRP A 34 0.909 -4.199 10.532 1.00 0.00 A2 C

ATOM 569 O TRP A 34 0.263 -5.022 11.177 1.00 0.00 A2 O

ATOM 570 N GLU A 35 0.360 -3.105 10.013 1.00 0.00 A2 N

ATOM 571 HN GLU A 35 0.846 -2.402 9.502 0.00 0.00 A2 H

ATOM 572 CA GLU A 35 -1.077 -2.846 10.167 1.00 0.00 A2 C

ATOM 573 HA GLU A 35 -1.344 -3.106 11.184 0.00 0.00 A2 H

ATOM 574 CB GLU A 35 -1.395 -1.368 9.913 1.00 0.00 A2 C

ATOM 575 HB1 GLU A 35 -0.930 -1.074 8.944 0.00 0.00 A2 H

ATOM 576 HB2 GLU A 35 -2.492 -1.236 9.777 0.00 0.00 A2 H

ATOM 577 CG GLU A 35 -0.903 -0.406 10.993 1.00 0.00 A2 C

ATOM 578 HG1 GLU A 35 0.184 -0.543 11.142 0.00 0.00 A2 H

ATOM 579 HG2 GLU A 35 -1.103 0.646 10.709 0.00 0.00 A2 H

ATOM 580 CD GLU A 35 -1.537 -0.664 12.351 1.00 0.00 A2 C

ATOM 581 OE1 GLU A 35 -2.691 -1.367 12.433 1.00 0.00 A2 O

ATOM 582 OE2 GLU A 35 -0.919 -0.165 13.447 1.00 0.00 A2 O

ATOM 583 C GLU A 35 -1.944 -3.703 9.236 1.00 0.00 A2 C

ATOM 584 O GLU A 35 -2.969 -4.236 9.654 1.00 0.00 A2 O

ATOM 585 N SER A 36 -1.547 -3.809 7.970 1.00 0.00 A2 N

ATOM 586 HN SER A 36 -0.652 -3.524 7.628 0.00 0.00 A2 H

ATOM 587 CA SER A 36 -2.429 -4.374 6.945 1.00 0.00 A2 C

ATOM 588 HA SER A 36 -3.150 -4.952 7.508 0.00 0.00 A2 H

ATOM 589 CB SER A 36 -3.037 -3.244 6.136 1.00 0.00 A2 C

ATOM 590 HB1 SER A 36 -3.802 -3.680 5.453 0.00 0.00 A2 H

ATOM 591 HB2 SER A 36 -3.566 -2.529 6.806 0.00 0.00 A2 H

ATOM 592 OG SER A 36 -2.014 -2.621 5.379 1.00 0.00 A2 O

ATOM 593 HG1 SER A 36 -2.448 -2.068 4.717 0.00 0.00 A2 H

ATOM 594 C SER A 36 -1.760 -5.325 5.949 1.00 0.00 A2 C

ATOM 595 O SER A 36 -2.433 -5.894 5.088 1.00 0.00 A2 O

ATOM 596 N GLY A 37 -0.444 -5.466 6.029 1.00 0.00 A2 N

ATOM 597 HN GLY A 37 0.112 -5.093 6.771 0.00 0.00 A2 H

ATOM 598 CA GLY A 37 0.282 -6.195 5.006 1.00 0.00 A2 C

ATOM 599 HA1 GLY A 37 -0.154 -7.183 4.947 0.00 0.00 A2 H

ATOM 600 HA2 GLY A 37 1.328 -6.187 5.283 0.00 0.00 A2 H

ATOM 601 C GLY A 37 0.154 -5.541 3.638 1.00 0.00 A2 C

ATOM 602 O GLY A 37 0.177 -6.210 2.605 1.00 0.00 A2 O

ATOM 603 N TYR A 38 0.015 -4.222 3.633 1.00 0.00 A2 N

ATOM 604 HN TYR A 38 -0.028 -3.673 4.466 0.00 0.00 A2 H

ATOM 605 CA TYR A 38 -0.088 -3.455 2.396 1.00 0.00 A2 C

ATOM 606 HA TYR A 38 -0.030 -2.414 2.689 0.00 0.00 A2 H

ATOM 607 CB TYR A 38 1.057 -3.781 1.417 1.00 0.00 A2 C

ATOM 608 HB1 TYR A 38 0.946 -4.829 1.063 0.00 0.00 A2 H

ATOM 609 HB2 TYR A 38 1.007 -3.124 0.521 0.00 0.00 A2 H

ATOM 610 CG TYR A 38 2.471 -3.696 1.969 1.00 0.00 A2 C

ATOM 611 CD1 TYR A 38 2.848 -2.689 2.854 1.00 0.00 A2 C

ATOM 612 HD1 TYR A 38 2.128 -1.941 3.156 0.00 0.00 A2 H

ATOM 613 CE1 TYR A 38 4.152 -2.614 3.345 1.00 0.00 A2 C

ATOM 614 HE1 TYR A 38 4.435 -1.833 4.035 0.00 0.00 A2 H

ATOM 615 CZ TYR A 38 5.088 -3.548 2.935 1.00 0.00 A2 C

ATOM 616 OH TYR A 38 6.375 -3.491 3.397 1.00 0.00 A2 O

ATOM 617 HH TYR A 38 6.838 -4.258 3.055 0.00 0.00 A2 H

ATOM 618 CD2 TYR A 38 3.440 -4.620 1.575 1.00 0.00 A2 C

ATOM 619 HD2 TYR A 38 3.193 -5.387 0.854 0.00 0.00 A2 H

ATOM 620 CE2 TYR A 38 4.739 -4.550 2.051 1.00 0.00 A2 C

ATOM 621 HE2 TYR A 38 5.473 -5.265 1.712 0.00 0.00 A2 H

ATOM 622 C TYR A 38 -1.434 -3.650 1.684 1.00 0.00 A2 C

ATOM 623 O TYR A 38 -1.539 -3.437 0.468 1.00 0.00 A2 O

ATOM 624 N ASN A 39 -2.451 -4.040 2.450 1.00 0.00 A2 N

ATOM 625 HN ASN A 39 -2.368 -4.139 3.441 0.00 0.00 A2 H

ATOM 626 CA ASN A 39 -3.777 -4.359 1.917 1.00 0.00 A2 C

ATOM 627 HA ASN A 39 -3.635 -4.489 0.849 0.00 0.00 A2 H

ATOM 628 CB ASN A 39 -4.308 -5.639 2.579 1.00 0.00 A2 C

ATOM 629 HB1 ASN A 39 -3.570 -6.450 2.393 0.00 0.00 A2 H

ATOM 630 HB2 ASN A 39 -4.383 -5.503 3.677 0.00 0.00 A2 H

ATOM 631 CG ASN A 39 -5.667 -6.097 2.010 1.00 0.00 A2 C

ATOM 632 OD1 ASN A 39 -6.149 -5.584 1.009 1.00 0.00 A2 O

ATOM 633 ND2 ASN A 39 -6.273 -7.076 2.664 1.00 0.00 A2 N

ATOM 634 1HD2 ASN A 39 -7.147 -7.394 2.306 0.00 0.00 A2 H

ATOM 635 2HD2 ASN A 39 -5.841 -7.485 3.462 0.00 0.00 A2 H

ATOM 636 C ASN A 39 -4.773 -3.209 2.117 1.00 0.00 A2 C

ATOM 637 O ASN A 39 -5.210 -2.942 3.238 1.00 0.00 A2 O

ATOM 638 N THR A 40 -5.128 -2.529 1.028 1.00 0.00 A2 N

ATOM 639 HN THR A 40 -4.813 -2.734 0.102 0.00 0.00 A2 H

ATOM 640 CA THR A 40 -6.041 -1.387 1.114 1.00 0.00 A2 C

ATOM 641 HA THR A 40 -5.641 -0.769 1.906 0.00 0.00 A2 H

ATOM 642 CB THR A 40 -6.119 -0.615 -0.214 1.00 0.00 A2 C

ATOM 643 HB THR A 40 -6.787 0.261 -0.025 0.00 0.00 A2 H

ATOM 644 OG1 THR A 40 -6.736 -1.437 -1.205 1.00 0.00 A2 O

ATOM 645 HG1 THR A 40 -6.745 -0.907 -2.009 0.00 0.00 A2 H

ATOM 646 CG2 THR A 40 -4.726 -0.204 -0.685 1.00 0.00 A2 C

ATOM 647 1HG2 THR A 40 -4.788 0.449 -1.581 0.00 0.00 A2 H

ATOM 648 2HG2 THR A 40 -4.198 0.360 0.113 0.00 0.00 A2 H

ATOM 649 3HG2 THR A 40 -4.113 -1.094 -0.946 0.00 0.00 A2 H

ATOM 650 C THR A 40 -7.460 -1.776 1.539 1.00 0.00 A2 C

ATOM 651 O THR A 40 -8.208 -0.946 2.036 1.00 0.00 A2 O

ATOM 652 N ARG A 41 -7.832 -3.038 1.343 1.00 0.00 A2 N

ATOM 653 HN ARG A 41 -7.230 -3.724 0.941 0.00 0.00 A2 H

ATOM 654 CA ARG A 41 -9.173 -3.482 1.719 1.00 0.00 A2 C

ATOM 655 HA ARG A 41 -9.826 -2.618 1.681 0.00 0.00 A2 H

ATOM 656 CB ARG A 41 -9.697 -4.529 0.721 1.00 0.00 A2 C

ATOM 657 HB1 ARG A 41 -8.842 -5.213 0.504 0.00 0.00 A2 H

ATOM 658 HB2 ARG A 41 -10.473 -5.156 1.213 0.00 0.00 A2 H

ATOM 659 CG ARG A 41 -10.206 -3.937 -0.599 1.00 0.00 A2 C

ATOM 660 HG1 ARG A 41 -11.247 -3.576 -0.447 0.00 0.00 A2 H

ATOM 661 HG2 ARG A 41 -9.607 -3.030 -0.844 0.00 0.00 A2 H

ATOM 662 CD ARG A 41 -10.071 -4.960 -1.737 1.00 0.00 A2 C

ATOM 663 HD1 ARG A 41 -9.017 -5.319 -1.790 0.00 0.00 A2 H

ATOM 664 HD2 ARG A 41 -10.740 -5.838 -1.579 0.00 0.00 A2 H

ATOM 665 NE ARG A 41 -10.432 -4.426 -3.055 1.00 0.00 A2 N

ATOM 666 HE ARG A 41 -11.203 -3.780 -3.022 0.00 0.00 A2 H

ATOM 667 CZ ARG A 41 -9.820 -4.745 -4.199 1.00 0.00 A2 C

ATOM 668 NH1 ARG A 41 -8.729 -5.648 -4.209 1.00 0.00 A2 N

ATOM 669 1HH1 ARG A 41 -8.261 -5.864 -5.054 0.00 0.00 A2 H

ATOM 670 2HH1 ARG A 41 -8.384 -5.937 -3.314 0.00 0.00 A2 H

ATOM 671 NH2 ARG A 41 -10.254 -4.171 -5.418 1.00 0.00 A2 N

ATOM 672 1HH2 ARG A 41 -9.859 -4.473 -6.274 0.00 0.00 A2 H

ATOM 673 2HH2 ARG A 41 -11.088 -3.636 -5.390 0.00 0.00 A2 H

ATOM 674 C ARG A 41 -9.244 -4.024 3.155 1.00 0.00 A2 C

ATOM 675 O ARG A 41 -10.311 -4.409 3.629 1.00 0.00 A2 O

ATOM 676 N ALA A 42 -8.121 -4.044 3.860 1.00 0.00 A2 N

ATOM 677 HN ALA A 42 -7.236 -3.715 3.532 0.00 0.00 A2 H

ATOM 678 CA ALA A 42 -8.127 -4.577 5.223 1.00 0.00 A2 C

ATOM 679 HA ALA A 42 -8.420 -5.621 5.205 0.00 0.00 A2 H

ATOM 680 CB ALA A 42 -6.723 -4.524 5.825 1.00 0.00 A2 C

ATOM 681 HB1 ALA A 42 -6.018 -5.086 5.176 0.00 0.00 A2 H

ATOM 682 HB2 ALA A 42 -6.359 -3.476 5.898 0.00 0.00 A2 H

ATOM 683 HB3 ALA A 42 -6.695 -4.980 6.838 0.00 0.00 A2 H

ATOM 684 C ALA A 42 -9.121 -3.866 6.141 1.00 0.00 A2 C

ATOM 685 O ALA A 42 -9.181 -2.635 6.165 1.00 0.00 A2 O

ATOM 686 N THR A 43 -9.896 -4.640 6.904 1.00 0.00 A2 N

ATOM 687 HN THR A 43 -9.975 -5.631 6.801 0.00 0.00 A2 H

ATOM 688 CA THR A 43 -10.716 -4.079 7.984 1.00 0.00 A2 C

ATOM 689 HA THR A 43 -10.276 -3.123 8.235 0.00 0.00 A2 H

ATOM 690 CB THR A 43 -12.211 -3.937 7.597 1.00 0.00 A2 C

ATOM 691 HB THR A 43 -12.722 -3.498 8.489 0.00 0.00 A2 H

ATOM 692 OG1 THR A 43 -12.771 -5.229 7.360 1.00 0.00 A2 O

ATOM 693 HG1 THR A 43 -13.689 -5.070 7.115 0.00 0.00 A2 H

ATOM 694 CG2 THR A 43 -12.375 -3.095 6.343 1.00 0.00 A2 C

ATOM 695 1HG2 THR A 43 -13.448 -2.901 6.135 0.00 0.00 A2 H

ATOM 696 2HG2 THR A 43 -11.869 -2.114 6.466 0.00 0.00 A2 H

ATOM 697 3HG2 THR A 43 -11.936 -3.605 5.458 0.00 0.00 A2 H

ATOM 698 C THR A 43 -10.607 -4.952 9.238 1.00 0.00 A2 C

ATOM 699 O THR A 43 -10.499 -6.176 9.143 1.00 0.00 A2 O

ATOM 700 N ASN A 44 -10.632 -4.323 10.408 1.00 0.00 A2 N

ATOM 701 HN ASN A 44 -10.705 -3.330 10.498 0.00 0.00 A2 H

ATOM 702 CA ASN A 44 -10.554 -5.042 11.678 1.00 0.00 A2 C

ATOM 703 HA ASN A 44 -10.783 -6.079 11.458 0.00 0.00 A2 H

ATOM 704 CB ASN A 44 -9.143 -4.924 12.292 1.00 0.00 A2 C

ATOM 705 HB1 ASN A 44 -8.425 -5.399 11.588 0.00 0.00 A2 H

ATOM 706 HB2 ASN A 44 -8.854 -3.858 12.393 0.00 0.00 A2 H

ATOM 707 CG ASN A 44 -9.015 -5.635 13.648 1.00 0.00 A2 C

ATOM 708 OD1 ASN A 44 -9.859 -6.439 14.032 1.00 0.00 A2 O

ATOM 709 ND2 ASN A 44 -7.941 -5.334 14.369 1.00 0.00 A2 N

ATOM 710 1HD2 ASN A 44 -7.835 -5.794 15.246 0.00 0.00 A2 H

ATOM 711 2HD2 ASN A 44 -7.266 -4.695 14.014 0.00 0.00 A2 H

ATOM 712 C ASN A 44 -11.630 -4.523 12.639 1.00 0.00 A2 C

ATOM 713 O ASN A 44 -11.512 -3.419 13.198 1.00 0.00 A2 O

ATOM 714 N TYR A 45 -12.686 -5.318 12.810 1.00 0.00 A2 N

ATOM 715 HN TYR A 45 -12.791 -6.196 12.346 0.00 0.00 A2 H

ATOM 716 CA TYR A 45 -13.789 -4.967 13.700 1.00 0.00 A2 C

ATOM 717 HA TYR A 45 -13.866 -3.886 13.695 0.00 0.00 A2 H

ATOM 718 CB TYR A 45 -15.086 -5.644 13.243 1.00 0.00 A2 C

ATOM 719 HB1 TYR A 45 -15.422 -5.181 12.290 0.00 0.00 A2 H

ATOM 720 HB2 TYR A 45 -14.916 -6.724 13.042 0.00 0.00 A2 H

ATOM 721 CG TYR A 45 -16.224 -5.490 14.230 1.00 0.00 A2 C

ATOM 722 CD1 TYR A 45 -16.698 -4.227 14.585 1.00 0.00 A2 C

ATOM 723 HD1 TYR A 45 -16.234 -3.343 14.170 0.00 0.00 A2 H

ATOM 724 CE1 TYR A 45 -17.750 -4.080 15.487 1.00 0.00 A2 C

ATOM 725 HE1 TYR A 45 -18.112 -3.098 15.754 0.00 0.00 A2 H

ATOM 726 CZ TYR A 45 -18.327 -5.208 16.048 1.00 0.00 A2 C

ATOM 727 OH TYR A 45 -19.360 -5.070 16.943 1.00 0.00 A2 O

ATOM 728 HH TYR A 45 -19.671 -5.951 17.162 0.00 0.00 A2 H

ATOM 729 CD2 TYR A 45 -16.824 -6.605 14.810 1.00 0.00 A2 C

ATOM 730 HD2 TYR A 45 -16.450 -7.595 14.590 0.00 0.00 A2 H

ATOM 731 CE2 TYR A 45 -17.872 -6.469 15.713 1.00 0.00 A2 C

ATOM 732 HE2 TYR A 45 -18.309 -7.348 16.163 0.00 0.00 A2 H

ATOM 733 C TYR A 45 -13.473 -5.388 15.129 1.00 0.00 A2 C

ATOM 734 O TYR A 45 -13.115 -6.542 15.375 1.00 0.00 A2 O

ATOM 735 N ASN A 46 -13.599 -4.448 16.060 1.00 0.00 A2 N

ATOM 736 HN ASN A 46 -13.884 -3.512 15.856 0.00 0.00 A2 H

ATOM 737 CA ASN A 46 -13.330 -4.705 17.473 1.00 0.00 A2 C

ATOM 738 HA ASN A 46 -12.844 -5.674 17.524 0.00 0.00 A2 H

ATOM 739 CB ASN A 46 -12.480 -3.578 18.075 1.00 0.00 A2 C

ATOM 740 HB1 ASN A 46 -13.143 -2.699 18.233 0.00 0.00 A2 H

ATOM 741 HB2 ASN A 46 -12.086 -3.880 19.067 0.00 0.00 A2 H

ATOM 742 CG ASN A 46 -11.332 -3.148 17.156 1.00 0.00 A2 C

ATOM 743 OD1 ASN A 46 -10.464 -3.951 16.818 1.00 0.00 A2 O

ATOM 744 ND2 ASN A 46 -11.331 -1.872 16.743 1.00 0.00 A2 N

ATOM 745 1HD2 ASN A 46 -10.594 -1.588 16.136 0.00 0.00 A2 H

ATOM 746 2HD2 ASN A 46 -12.064 -1.258 17.019 0.00 0.00 A2 H

ATOM 747 C ASN A 46 -14.643 -4.808 18.234 1.00 0.00 A2 C

ATOM 748 O ASN A 46 -15.251 -3.788 18.561 1.00 0.00 A2 O

ATOM 749 N ALA A 47 -15.085 -6.033 18.508 1.00 0.00 A2 N

ATOM 750 HN ALA A 47 -14.575 -6.863 18.283 0.00 0.00 A2 H

ATOM 751 CA ALA A 47 -16.374 -6.247 19.170 1.00 0.00 A2 C

ATOM 752 HA ALA A 47 -17.185 -5.885 18.549 0.00 0.00 A2 H

ATOM 753 CB ALA A 47 -16.617 -7.736 19.412 1.00 0.00 A2 C

ATOM 754 HB1 ALA A 47 -16.603 -8.278 18.443 0.00 0.00 A2 H

ATOM 755 HB2 ALA A 47 -15.823 -8.170 20.058 0.00 0.00 A2 H

ATOM 756 HB3 ALA A 47 -17.603 -7.917 19.892 0.00 0.00 A2 H

ATOM 757 C ALA A 47 -16.457 -5.472 20.482 1.00 0.00 A2 C

ATOM 758 O ALA A 47 -17.526 -5.004 20.865 1.00 0.00 A2 O

ATOM 759 N GLY A 48 -15.313 -5.335 21.148 1.00 0.00 A2 N

ATOM 760 HN GLY A 48 -14.468 -5.743 20.804 0.00 0.00 A2 H

ATOM 761 CA GLY A 48 -15.194 -4.602 22.398 1.00 0.00 A2 C

ATOM 762 HA1 GLY A 48 -14.134 -4.452 22.553 0.00 0.00 A2 H

ATOM 763 HA2 GLY A 48 -15.671 -5.214 23.152 0.00 0.00 A2 H

ATOM 764 C GLY A 48 -15.868 -3.241 22.405 1.00 0.00 A2 C

ATOM 765 O GLY A 48 -16.815 -3.023 23.157 1.00 0.00 A2 O

ATOM 766 N ASP A 49 -15.390 -2.319 21.571 1.00 0.00 A2 N

ATOM 767 HN ASP A 49 -14.639 -2.432 20.926 0.00 0.00 A2 H

ATOM 768 CA ASP A 49 -15.973 -0.978 21.537 1.00 0.00 A2 C

ATOM 769 HA ASP A 49 -16.704 -0.939 22.337 0.00 0.00 A2 H

ATOM 770 CB ASP A 49 -14.907 0.085 21.751 1.00 0.00 A2 C

ATOM 771 HB1 ASP A 49 -15.359 1.088 21.614 0.00 0.00 A2 H

ATOM 772 HB2 ASP A 49 -14.496 0.025 22.778 0.00 0.00 A2 H

ATOM 773 CG ASP A 49 -13.766 -0.036 20.768 1.00 0.00 A2 C

ATOM 774 OD1 ASP A 49 -13.042 -1.182 20.749 1.00 0.00 A2 O

ATOM 775 OD2 ASP A 49 -13.514 1.023 19.959 1.00 0.00 A2 O

ATOM 776 C ASP A 49 -16.722 -0.709 20.247 1.00 0.00 A2 C

ATOM 777 O ASP A 49 -17.052 0.434 19.932 1.00 0.00 A2 O

ATOM 778 N ARG A 50 -16.985 -1.768 19.500 1.00 0.00 A2 N

ATOM 779 HN ARG A 50 -16.654 -2.679 19.735 0.00 0.00 A2 H

ATOM 780 CA ARG A 50 -17.779 -1.655 18.286 1.00 0.00 A2 C

ATOM 781 HA ARG A 50 -17.776 -2.626 17.805 0.00 0.00 A2 H

ATOM 782 CB ARG A 50 -19.236 -1.310 18.630 1.00 0.00 A2 C

ATOM 783 HB1 ARG A 50 -19.200 -0.410 19.289 0.00 0.00 A2 H

ATOM 784 HB2 ARG A 50 -19.771 -0.993 17.708 0.00 0.00 A2 H

ATOM 785 CG ARG A 50 -19.977 -2.422 19.353 1.00 0.00 A2 C

ATOM 786 HG1 ARG A 50 -20.089 -3.283 18.658 0.00 0.00 A2 H

ATOM 787 HG2 ARG A 50 -19.343 -2.794 20.191 0.00 0.00 A2 H

ATOM 788 CD ARG A 50 -21.302 -1.938 19.916 1.00 0.00 A2 C

ATOM 789 HD1 ARG A 50 -21.890 -1.446 19.108 0.00 0.00 A2 H

ATOM 790 HD2 ARG A 50 -21.902 -2.781 20.332 0.00 0.00 A2 H

ATOM 791 NE ARG A 50 -21.116 -0.987 21.009 1.00 0.00 A2 N

ATOM 792 HE ARG A 50 -21.099 -0.029 20.701 0.00 0.00 A2 H

ATOM 793 CZ ARG A 50 -20.981 -1.334 22.285 1.00 0.00 A2 C

ATOM 794 NH1 ARG A 50 -21.012 -2.699 22.657 1.00 0.00 A2 N

ATOM 795 1HH1 ARG A 50 -20.938 -2.964 23.608 0.00 0.00 A2 H

ATOM 796 2HH1 ARG A 50 -21.248 -3.351 21.933 0.00 0.00 A2 H

ATOM 797 NH2 ARG A 50 -20.805 -0.338 23.277 1.00 0.00 A2 N

ATOM 798 1HH2 ARG A 50 -20.618 -0.601 24.213 0.00 0.00 A2 H

ATOM 799 2HH2 ARG A 50 -20.667 0.591 22.960 0.00 0.00 A2 H

ATOM 800 C ARG A 50 -17.198 -0.663 17.274 1.00 0.00 A2 C

ATOM 801 O ARG A 50 -17.927 -0.090 16.459 1.00 0.00 A2 O

ATOM 802 N SER A 51 -15.882 -0.486 17.304 1.00 0.00 A2 N

ATOM 803 HN SER A 51 -15.266 -0.874 17.990 0.00 0.00 A2 H

ATOM 804 CA SER A 51 -15.213 0.326 16.290 1.00 0.00 A2 C

ATOM 805 HA SER A 51 -15.995 0.845 15.751 0.00 0.00 A2 H

ATOM 806 CB SER A 51 -14.243 1.315 16.942 1.00 0.00 A2 C

ATOM 807 HB1 SER A 51 -13.935 2.058 16.171 0.00 0.00 A2 H

ATOM 808 HB2 SER A 51 -14.753 1.880 17.755 0.00 0.00 A2 H

ATOM 809 OG SER A 51 -13.087 0.651 17.425 1.00 0.00 A2 O

ATOM 810 HG1 SER A 51 -12.428 1.331 17.615 0.00 0.00 A2 H

ATOM 811 C SER A 51 -14.482 -0.565 15.284 1.00 0.00 A2 C

ATOM 812 O SER A 51 -14.268 -1.748 15.534 1.00 0.00 A2 O

ATOM 813 N THR A 52 -14.098 0.011 14.148 1.00 0.00 A2 N

ATOM 814 HN THR A 52 -14.236 0.979 13.940 0.00 0.00 A2 H

ATOM 815 CA THR A 52 -13.430 -0.740 13.091 1.00 0.00 A2 C

ATOM 816 HA THR A 52 -13.041 -1.636 13.556 0.00 0.00 A2 H

ATOM 817 CB THR A 52 -14.402 -1.044 11.913 1.00 0.00 A2 C

ATOM 818 HB THR A 52 -14.767 -0.053 11.548 0.00 0.00 A2 H

ATOM 819 OG1 THR A 52 -15.542 -1.783 12.386 1.00 0.00 A2 O

ATOM 820 HG1 THR A 52 -16.086 -1.942 11.607 0.00 0.00 A2 H

ATOM 821 CG2 THR A 52 -13.713 -1.828 10.789 1.00 0.00 A2 C

ATOM 822 1HG2 THR A 52 -14.386 -1.936 9.912 0.00 0.00 A2 H

ATOM 823 2HG2 THR A 52 -12.797 -1.298 10.452 0.00 0.00 A2 H

ATOM 824 3HG2 THR A 52 -13.421 -2.844 11.131 0.00 0.00 A2 H

ATOM 825 C THR A 52 -12.224 0.037 12.568 1.00 0.00 A2 C

ATOM 826 O THR A 52 -12.294 1.260 12.388 1.00 0.00 A2 O

ATOM 827 N ASP A 53 -11.118 -0.666 12.333 1.00 0.00 A2 N

ATOM 828 HN ASP A 53 -10.994 -1.630 12.555 0.00 0.00 A2 H

ATOM 829 CA ASP A 53 -9.934 -0.066 11.702 1.00 0.00 A2 C

ATOM 830 HA ASP A 53 -9.976 1.005 11.865 0.00 0.00 A2 H

ATOM 831 CB ASP A 53 -8.654 -0.717 12.233 1.00 0.00 A2 C

ATOM 832 HB1 ASP A 53 -8.656 -1.794 11.971 0.00 0.00 A2 H

ATOM 833 HB2 ASP A 53 -7.759 -0.249 11.777 0.00 0.00 A2 H

ATOM 834 CG ASP A 53 -8.528 -0.619 13.753 1.00 0.00 A2 C

ATOM 835 OD1 ASP A 53 -8.749 0.574 14.358 1.00 0.00 A2 O

ATOM 836 OD2 ASP A 53 -8.190 -1.730 14.453 1.00 0.00 A2 O

ATOM 837 C ASP A 53 -10.007 -0.285 10.191 1.00 0.00 A2 C

ATOM 838 O ASP A 53 -10.298 -1.394 9.744 1.00 0.00 A2 O

ATOM 839 N TYR A 54 -9.712 0.753 9.411 1.00 0.00 A2 N

ATOM 840 HN TYR A 54 -9.342 1.616 9.750 0.00 0.00 A2 H

ATOM 841 CA TYR A 54 -9.912 0.705 7.965 1.00 0.00 A2 C

ATOM 842 HA TYR A 54 -10.253 -0.303 7.760 0.00 0.00 A2 H

ATOM 843 CB TYR A 54 -10.952 1.755 7.532 1.00 0.00 A2 C

ATOM 844 HB1 TYR A 54 -10.705 2.730 8.006 0.00 0.00 A2 H

ATOM 845 HB2 TYR A 54 -10.921 1.908 6.431 0.00 0.00 A2 H

ATOM 846 CG TYR A 54 -12.366 1.423 7.945 1.00 0.00 A2 C

ATOM 847 CD1 TYR A 54 -12.863 1.831 9.179 1.00 0.00 A2 C

ATOM 848 HD1 TYR A 54 -12.226 2.378 9.861 0.00 0.00 A2 H

ATOM 849 CE1 TYR A 54 -14.156 1.532 9.566 1.00 0.00 A2 C

ATOM 850 HE1 TYR A 54 -14.528 1.847 10.530 0.00 0.00 A2 H

ATOM 851 CZ TYR A 54 -14.976 0.817 8.714 1.00 0.00 A2 C

ATOM 852 OH TYR A 54 -16.261 0.498 9.108 1.00 0.00 A2 O

ATOM 853 HH TYR A 54 -16.692 0.070 8.365 0.00 0.00 A2 H

ATOM 854 CD2 TYR A 54 -13.206 0.698 7.104 1.00 0.00 A2 C

ATOM 855 HD2 TYR A 54 -12.838 0.335 6.154 0.00 0.00 A2 H

ATOM 856 CE2 TYR A 54 -14.504 0.387 7.488 1.00 0.00 A2 C

ATOM 857 HE2 TYR A 54 -15.128 -0.206 6.836 0.00 0.00 A2 H

ATOM 858 C TYR A 54 -8.648 0.925 7.150 1.00 0.00 A2 C

ATOM 859 O TYR A 54 -7.886 1.865 7.388 1.00 0.00 A2 O

ATOM 860 N GLY A 55 -8.442 0.056 6.173 1.00 0.00 A2 N

ATOM 861 HN GLY A 55 -8.923 -0.818 6.107 0.00 0.00 A2 H

ATOM 862 CA GLY A 55 -7.487 0.326 5.119 1.00 0.00 A2 C

ATOM 863 HA1 GLY A 55 -7.584 1.384 4.917 0.00 0.00 A2 H

ATOM 864 HA2 GLY A 55 -7.782 -0.312 4.297 0.00 0.00 A2 H

ATOM 865 C GLY A 55 -6.034 0.040 5.434 1.00 0.00 A2 C

ATOM 866 O GLY A 55 -5.689 -0.588 6.431 1.00 0.00 A2 O

ATOM 867 N ILE A 56 -5.174 0.507 4.546 1.00 0.00 A2 N

ATOM 868 HN ILE A 56 -5.424 1.126 3.804 0.00 0.00 A2 H

ATOM 869 CA ILE A 56 -3.766 0.152 4.576 1.00 0.00 A2 C

ATOM 870 HA ILE A 56 -3.734 -0.930 4.582 0.00 0.00 A2 H

ATOM 871 CB ILE A 56 -3.072 0.686 3.309 1.00 0.00 A2 C

ATOM 872 HB ILE A 56 -3.719 0.333 2.466 0.00 0.00 A2 H

ATOM 873 CG2 ILE A 56 -3.014 2.222 3.327 1.00 0.00 A2 C

ATOM 874 1HG2 ILE A 56 -2.658 2.611 2.350 0.00 0.00 A2 H

ATOM 875 2HG2 ILE A 56 -4.019 2.654 3.515 0.00 0.00 A2 H

ATOM 876 3HG2 ILE A 56 -2.323 2.584 4.117 0.00 0.00 A2 H

ATOM 877 CG1 ILE A 56 -1.704 0.040 3.101 1.00 0.00 A2 C

ATOM 878 1HG1 ILE A 56 -0.995 0.400 3.880 0.00 0.00 A2 H

ATOM 879 2HG1 ILE A 56 -1.802 -1.062 3.229 0.00 0.00 A2 H

ATOM 880 CD ILE A 56 -1.110 0.313 1.714 1.00 0.00 A2 C

ATOM 881 HD1 ILE A 56 -0.145 -0.225 1.595 0.00 0.00 A2 H

ATOM 882 HD2 ILE A 56 -1.802 -0.033 0.916 0.00 0.00 A2 H

ATOM 883 HD3 ILE A 56 -0.918 1.397 1.569 0.00 0.00 A2 H

ATOM 884 C ILE A 56 -3.071 0.625 5.864 1.00 0.00 A2 C

ATOM 885 O ILE A 56 -2.114 -0.009 6.316 1.00 0.00 A2 O

ATOM 886 N PHE A 57 -3.571 1.710 6.465 1.00 0.00 A2 N

ATOM 887 HN PHE A 57 -4.338 2.239 6.105 0.00 0.00 A2 H

ATOM 888 CA PHE A 57 -3.018 2.222 7.733 1.00 0.00 A2 C

ATOM 889 HA PHE A 57 -2.069 1.716 7.858 0.00 0.00 A2 H

ATOM 890 CB PHE A 57 -2.792 3.751 7.695 1.00 0.00 A2 C

ATOM 891 HB1 PHE A 57 -3.753 4.255 7.451 0.00 0.00 A2 H

ATOM 892 HB2 PHE A 57 -2.465 4.132 8.686 0.00 0.00 A2 H

ATOM 893 CG PHE A 57 -1.810 4.205 6.646 1.00 0.00 A2 C

ATOM 894 CD1 PHE A 57 -0.490 3.784 6.686 1.00 0.00 A2 C

ATOM 895 HD1 PHE A 57 -0.161 3.130 7.482 0.00 0.00 A2 H

ATOM 896 CE1 PHE A 57 0.419 4.198 5.718 1.00 0.00 A2 C

ATOM 897 HE1 PHE A 57 1.441 3.847 5.751 0.00 0.00 A2 H

ATOM 898 CZ PHE A 57 0.011 5.056 4.707 1.00 0.00 A2 C

ATOM 899 HZ PHE A 57 0.711 5.381 3.951 0.00 0.00 A2 H

ATOM 900 CD2 PHE A 57 -2.203 5.063 5.632 1.00 0.00 A2 C

ATOM 901 HD2 PHE A 57 -3.219 5.430 5.598 0.00 0.00 A2 H

ATOM 902 CE2 PHE A 57 -1.305 5.488 4.664 1.00 0.00 A2 C

ATOM 903 HE2 PHE A 57 -1.634 6.156 3.880 0.00 0.00 A2 H

ATOM 904 C PHE A 57 -3.878 1.869 8.947 1.00 0.00 A2 C

ATOM 905 O PHE A 57 -3.591 2.307 10.066 1.00 0.00 A2 O

ATOM 906 N GLN A 58 -4.931 1.081 8.728 1.00 0.00 A2 N

ATOM 907 HN GLN A 58 -5.173 0.721 7.829 0.00 0.00 A2 H

ATOM 908 CA GLN A 58 -5.832 0.674 9.806 1.00 0.00 A2 C

ATOM 909 HA GLN A 58 -6.711 0.267 9.321 0.00 0.00 A2 H

ATOM 910 CB GLN A 58 -5.206 -0.448 10.632 1.00 0.00 A2 C

ATOM 911 HB1 GLN A 58 -4.178 -0.114 10.902 0.00 0.00 A2 H

ATOM 912 HB2 GLN A 58 -5.761 -0.553 11.592 0.00 0.00 A2 H

ATOM 913 CG GLN A 58 -5.153 -1.765 9.855 1.00 0.00 A2 C

ATOM 914 HG1 GLN A 58 -4.706 -1.604 8.850 0.00 0.00 A2 H

ATOM 915 HG2 GLN A 58 -4.530 -2.511 10.389 0.00 0.00 A2 H

ATOM 916 CD GLN A 58 -6.535 -2.347 9.628 1.00 0.00 A2 C

ATOM 917 OE1 GLN A 58 -7.144 -2.130 8.580 1.00 0.00 A2 O

ATOM 918 NE2 GLN A 58 -7.038 -3.098 10.608 1.00 0.00 A2 N

ATOM 919 1HE2 GLN A 58 -7.944 -3.485 10.459 0.00 0.00 A2 H

ATOM 920 2HE2 GLN A 58 -6.517 -3.234 11.444 0.00 0.00 A2 H

ATOM 921 C GLN A 58 -6.290 1.836 10.691 1.00 0.00 A2 C

ATOM 922 O GLN A 58 -6.109 1.835 11.912 1.00 0.00 A2 O

ATOM 923 N ILE A 59 -6.909 2.818 10.056 1.00 0.00 A2 N

ATOM 924 HN ILE A 59 -7.085 2.815 9.074 0.00 0.00 A2 H

ATOM 925 CA ILE A 59 -7.395 4.009 10.748 1.00 0.00 A2 C

ATOM 926 HA ILE A 59 -6.692 4.203 11.548 0.00 0.00 A2 H

ATOM 927 CB ILE A 59 -7.431 5.187 9.759 1.00 0.00 A2 C

ATOM 928 HB ILE A 59 -7.903 4.764 8.837 0.00 0.00 A2 H

ATOM 929 CG2 ILE A 59 -8.220 6.365 10.329 1.00 0.00 A2 C

ATOM 930 1HG2 ILE A 59 -8.373 7.145 9.554 0.00 0.00 A2 H

ATOM 931 2HG2 ILE A 59 -9.221 6.038 10.680 0.00 0.00 A2 H

ATOM 932 3HG2 ILE A 59 -7.682 6.827 11.184 0.00 0.00 A2 H

ATOM 933 CG1 ILE A 59 -5.987 5.581 9.398 1.00 0.00 A2 C

ATOM 934 1HG1 ILE A 59 -5.512 6.100 10.260 0.00 0.00 A2 H

ATOM 935 2HG1 ILE A 59 -5.398 4.655 9.206 0.00 0.00 A2 H

ATOM 936 CD ILE A 59 -5.849 6.472 8.169 1.00 0.00 A2 C

ATOM 937 HD1 ILE A 59 -4.776 6.660 7.947 0.00 0.00 A2 H

ATOM 938 HD2 ILE A 59 -6.309 5.988 7.280 0.00 0.00 A2 H

ATOM 939 HD3 ILE A 59 -6.343 7.453 8.331 0.00 0.00 A2 H

ATOM 940 C ILE A 59 -8.765 3.755 11.409 1.00 0.00 A2 C

ATOM 941 O ILE A 59 -9.676 3.210 10.781 1.00 0.00 A2 O

ATOM 942 N ASN A 60 -8.894 4.156 12.670 1.00 0.00 A2 N

ATOM 943 HN ASN A 60 -8.226 4.747 13.121 0.00 0.00 A2 H

ATOM 944 CA ASN A 60 -10.032 3.766 13.515 1.00 0.00 A2 C

ATOM 945 HA ASN A 60 -10.327 2.796 13.129 0.00 0.00 A2 H

ATOM 946 CB ASN A 60 -9.593 3.631 14.986 1.00 0.00 A2 C

ATOM 947 HB1 ASN A 60 -8.694 2.977 15.014 0.00 0.00 A2 H

ATOM 948 HB2 ASN A 60 -9.298 4.620 15.393 0.00 0.00 A2 H

ATOM 949 CG ASN A 60 -10.668 2.998 15.860 1.00 0.00 A2 C

ATOM 950 OD1 ASN A 60 -11.364 3.684 16.593 1.00 0.00 A2 O

ATOM 951 ND2 ASN A 60 -10.803 1.681 15.778 1.00 0.00 A2 N

ATOM 952 1HD2 ASN A 60 -11.511 1.258 16.337 0.00 0.00 A2 H

ATOM 953 2HD2 ASN A 60 -10.230 1.159 15.154 0.00 0.00 A2 H

ATOM 954 C ASN A 60 -11.244 4.683 13.431 1.00 0.00 A2 C

ATOM 955 O ASN A 60 -11.125 5.905 13.497 1.00 0.00 A2 O

ATOM 956 N SER A 61 -12.416 4.067 13.309 1.00 0.00 A2 N

ATOM 957 HN SER A 61 -12.509 3.072 13.330 0.00 0.00 A2 H

ATOM 958 CA SER A 61 -13.672 4.779 13.134 1.00 0.00 A2 C

ATOM 959 HA SER A 61 -13.457 5.536 12.392 0.00 0.00 A2 H

ATOM 960 CB SER A 61 -14.751 3.798 12.655 1.00 0.00 A2 C

ATOM 961 HB1 SER A 61 -15.676 4.380 12.439 0.00 0.00 A2 H

ATOM 962 HB2 SER A 61 -14.439 3.313 11.702 0.00 0.00 A2 H

ATOM 963 OG SER A 61 -15.031 2.836 13.663 1.00 0.00 A2 O

ATOM 964 HG1 SER A 61 -15.852 2.393 13.412 0.00 0.00 A2 H

ATOM 965 C SER A 61 -14.166 5.501 14.392 1.00 0.00 A2 C

ATOM 966 O SER A 61 -15.106 6.290 14.322 1.00 0.00 A2 O

ATOM 967 N ARG A 62 -13.568 5.233 15.549 1.00 0.00 A2 N

ATOM 968 HN ARG A 62 -12.825 4.579 15.667 0.00 0.00 A2 H

ATOM 969 CA ARG A 62 -14.018 5.938 16.747 1.00 0.00 A2 C

ATOM 970 HA ARG A 62 -15.102 5.946 16.744 0.00 0.00 A2 H

ATOM 971 CB ARG A 62 -13.588 5.233 18.040 1.00 0.00 A2 C

ATOM 972 HB1 ARG A 62 -14.080 4.231 18.031 0.00 0.00 A2 H

ATOM 973 HB2 ARG A 62 -12.493 5.037 18.011 0.00 0.00 A2 H

ATOM 974 CG ARG A 62 -14.002 5.989 19.319 1.00 0.00 A2 C

ATOM 975 HG1 ARG A 62 -13.401 6.922 19.389 0.00 0.00 A2 H

ATOM 976 HG2 ARG A 62 -15.060 6.322 19.216 0.00 0.00 A2 H

ATOM 977 CD ARG A 62 -13.877 5.101 20.553 1.00 0.00 A2 C

ATOM 978 HD1 ARG A 62 -12.835 4.712 20.625 0.00 0.00 A2 H

ATOM 979 HD2 ARG A 62 -14.114 5.663 21.487 0.00 0.00 A2 H

ATOM 980 NE ARG A 62 -14.817 3.988 20.461 1.00 0.00 A2 N

ATOM 981 HE ARG A 62 -14.374 3.114 20.232 0.00 0.00 A2 H

ATOM 982 CZ ARG A 62 -16.123 4.122 20.660 1.00 0.00 A2 C

ATOM 983 NH1 ARG A 62 -16.646 5.394 20.998 1.00 0.00 A2 N

ATOM 984 1HH1 ARG A 62 -17.613 5.511 21.175 0.00 0.00 A2 H

ATOM 985 2HH1 ARG A 62 -15.975 6.116 21.178 0.00 0.00 A2 H

ATOM 986 NH2 ARG A 62 -16.988 3.008 20.534 1.00 0.00 A2 N

ATOM 987 1HH2 ARG A 62 -17.968 3.134 20.598 0.00 0.00 A2 H

ATOM 988 2HH2 ARG A 62 -16.593 2.172 20.177 0.00 0.00 A2 H

ATOM 989 C ARG A 62 -13.569 7.406 16.752 1.00 0.00 A2 C

ATOM 990 O ARG A 62 -14.290 8.273 17.234 1.00 0.00 A2 O

ATOM 991 N TYR A 63 -12.404 7.691 16.185 1.00 0.00 A2 N

ATOM 992 HN TYR A 63 -11.821 7.052 15.685 0.00 0.00 A2 H

ATOM 993 CA TYR A 63 -11.880 9.050 16.270 1.00 0.00 A2 C

ATOM 994 HA TYR A 63 -12.634 9.608 16.812 0.00 0.00 A2 H

ATOM 995 CB TYR A 63 -10.560 9.069 17.051 1.00 0.00 A2 C

ATOM 996 HB1 TYR A 63 -9.769 8.579 16.442 0.00 0.00 A2 H

ATOM 997 HB2 TYR A 63 -10.230 10.114 17.239 0.00 0.00 A2 H

ATOM 998 CG TYR A 63 -10.601 8.324 18.372 1.00 0.00 A2 C

ATOM 999 CD1 TYR A 63 -11.079 8.934 19.526 1.00 0.00 A2 C

ATOM 1000 HD1 TYR A 63 -11.412 9.962 19.492 0.00 0.00 A2 H

ATOM 1001 CE1 TYR A 63 -11.118 8.256 20.732 1.00 0.00 A2 C

ATOM 1002 HE1 TYR A 63 -11.493 8.739 21.622 0.00 0.00 A2 H

ATOM 1003 CZ TYR A 63 -10.664 6.949 20.792 1.00 0.00 A2 C

ATOM 1004 OH TYR A 63 -10.696 6.259 21.981 1.00 0.00 A2 O

ATOM 1005 HH TYR A 63 -10.411 5.361 21.801 0.00 0.00 A2 H

ATOM 1006 CD2 TYR A 63 -10.159 7.005 18.461 1.00 0.00 A2 C

ATOM 1007 HD2 TYR A 63 -9.752 6.511 17.589 0.00 0.00 A2 H

ATOM 1008 CE2 TYR A 63 -10.184 6.323 19.663 1.00 0.00 A2 C

ATOM 1009 HE2 TYR A 63 -9.808 5.312 19.714 0.00 0.00 A2 H

ATOM 1010 C TYR A 63 -11.688 9.743 14.923 1.00 0.00 A2 C

ATOM 1011 O TYR A 63 -11.790 10.972 14.830 1.00 0.00 A2 O

ATOM 1012 N TRP A 64 -11.431 8.963 13.877 1.00 0.00 A2 N

ATOM 1013 HN TRP A 64 -11.610 7.983 13.837 0.00 0.00 A2 H

ATOM 1014 CA TRP A 64 -10.841 9.534 12.666 1.00 0.00 A2 C

ATOM 1015 HA TRP A 64 -10.618 10.561 12.930 0.00 0.00 A2 H

ATOM 1016 CB TRP A 64 -9.512 8.843 12.351 1.00 0.00 A2 C

ATOM 1017 HB1 TRP A 64 -9.717 7.759 12.207 0.00 0.00 A2 H

ATOM 1018 HB2 TRP A 64 -9.107 9.224 11.389 0.00 0.00 A2 H

ATOM 1019 CG TRP A 64 -8.500 8.978 13.455 1.00 0.00 A2 C

ATOM 1020 CD1 TRP A 64 -8.081 7.988 14.307 1.00 0.00 A2 C

ATOM 1021 HD1 TRP A 64 -8.417 6.960 14.265 0.00 0.00 A2 H

ATOM 1022 NE1 TRP A 64 -7.145 8.487 15.187 1.00 0.00 A2 N

ATOM 1023 HE1 TRP A 64 -6.665 7.981 15.871 0.00 0.00 A2 H

ATOM 1024 CE2 TRP A 64 -6.948 9.818 14.919 1.00 0.00 A2 C

ATOM 1025 CD2 TRP A 64 -7.787 10.164 13.837 1.00 0.00 A2 C

ATOM 1026 CE3 TRP A 64 -7.772 11.482 13.367 1.00 0.00 A2 C

ATOM 1027 HE3 TRP A 64 -8.394 11.800 12.541 0.00 0.00 A2 H

ATOM 1028 CZ3 TRP A 64 -6.949 12.397 13.979 1.00 0.00 A2 C

ATOM 1029 HZ3 TRP A 64 -6.935 13.419 13.626 0.00 0.00 A2 H

ATOM 1030 CZ2 TRP A 64 -6.115 10.744 15.543 1.00 0.00 A2 C

ATOM 1031 HZ2 TRP A 64 -5.477 10.482 16.373 0.00 0.00 A2 H

ATOM 1032 CH2 TRP A 64 -6.125 12.024 15.064 1.00 0.00 A2 C

ATOM 1033 HH2 TRP A 64 -5.488 12.764 15.529 0.00 0.00 A2 H

ATOM 1034 C TRP A 64 -11.711 9.577 11.410 1.00 0.00 A2 C

ATOM 1035 O TRP A 64 -11.618 10.519 10.638 1.00 0.00 A2 O

ATOM 1036 CB CYS A 65 -12.804 7.546 8.938 1.00 0.00 A2 C

ATOM 1037 SG CYS A 65 -12.671 5.782 9.452 1.00 0.00 A2 S

ATOM 1038 N CYS A 65 -12.522 8.553 11.178 1.00 0.00 A2 N

ATOM 1039 HN CYS A 65 -12.617 7.759 11.776 0.00 0.00 A2 H

ATOM 1040 CA CYS A 65 -13.356 8.523 9.989 1.00 0.00 A2 C

ATOM 1041 HA CYS A 65 -13.392 9.529 9.588 0.00 0.00 A2 H

ATOM 1042 HB1 CYS A 65 -13.490 7.582 8.061 0.00 0.00 A2 H

ATOM 1043 HB2 CYS A 65 -11.817 7.905 8.571 0.00 0.00 A2 H

ATOM 1044 C CYS A 65 -14.766 8.137 10.393 1.00 0.00 A2 C

ATOM 1045 O CYS A 65 -14.972 7.578 11.462 1.00 0.00 A2 O

ATOM 1046 N ASN A 66 -15.737 8.430 9.539 1.00 0.00 A2 N

ATOM 1047 HN ASN A 66 -15.618 8.895 8.662 0.00 0.00 A2 H

ATOM 1048 CA ASN A 66 -17.110 8.070 9.849 1.00 0.00 A2 C

ATOM 1049 HA ASN A 66 -17.108 7.848 10.911 0.00 0.00 A2 H

ATOM 1050 CB ASN A 66 -18.055 9.223 9.532 1.00 0.00 A2 C

ATOM 1051 HB1 ASN A 66 -17.788 10.074 10.196 0.00 0.00 A2 H

ATOM 1052 HB2 ASN A 66 -17.914 9.561 8.485 0.00 0.00 A2 H

ATOM 1053 CG ASN A 66 -19.491 8.855 9.783 1.00 0.00 A2 C

ATOM 1054 OD1 ASN A 66 -19.834 8.392 10.868 1.00 0.00 A2 O

ATOM 1055 ND2 ASN A 66 -20.341 9.038 8.773 1.00 0.00 A2 N

ATOM 1056 1HD2 ASN A 66 -21.297 8.808 8.932 0.00 0.00 A2 H

ATOM 1057 2HD2 ASN A 66 -20.018 9.419 7.912 0.00 0.00 A2 H

ATOM 1058 C ASN A 66 -17.585 6.816 9.115 1.00 0.00 A2 C

ATOM 1059 O ASN A 66 -17.566 6.766 7.889 1.00 0.00 A2 O

ATOM 1060 N ASP A 67 -18.026 5.807 9.865 1.00 0.00 A2 N

ATOM 1061 HN ASP A 67 -17.978 5.737 10.858 0.00 0.00 A2 H

ATOM 1062 CA ASP A 67 -18.659 4.644 9.242 1.00 0.00 A2 C

ATOM 1063 HA ASP A 67 -18.608 4.806 8.171 0.00 0.00 A2 H

ATOM 1064 CB ASP A 67 -17.886 3.342 9.508 1.00 0.00 A2 C

ATOM 1065 HB1 ASP A 67 -18.337 2.522 8.914 0.00 0.00 A2 H

ATOM 1066 HB2 ASP A 67 -16.825 3.449 9.205 0.00 0.00 A2 H

ATOM 1067 CG ASP A 67 -17.914 2.896 10.971 1.00 0.00 A2 C

ATOM 1068 OD1 ASP A 67 -18.638 3.563 11.903 1.00 0.00 A2 O

ATOM 1069 OD2 ASP A 67 -17.194 1.790 11.282 1.00 0.00 A2 O

ATOM 1070 C ASP A 67 -20.147 4.515 9.600 1.00 0.00 A2 C

ATOM 1071 O ASP A 67 -20.822 3.547 9.214 1.00 0.00 A2 O

ATOM 1072 N GLY A 68 -20.645 5.508 10.328 1.00 0.00 A2 N

ATOM 1073 HN GLY A 68 -20.075 6.253 10.673 0.00 0.00 A2 H

ATOM 1074 CA GLY A 68 -22.048 5.582 10.673 1.00 0.00 A2 C

ATOM 1075 HA1 GLY A 68 -22.593 5.528 9.740 0.00 0.00 A2 H

ATOM 1076 HA2 GLY A 68 -22.183 6.509 11.213 0.00 0.00 A2 H

ATOM 1077 C GLY A 68 -22.524 4.451 11.560 1.00 0.00 A2 C

ATOM 1078 O GLY A 68 -23.723 4.272 11.750 1.00 0.00 A2 O

ATOM 1079 N LYS A 69 -21.603 3.686 12.129 1.00 0.00 A2 N

ATOM 1080 HN LYS A 69 -20.614 3.782 12.029 0.00 0.00 A2 H

ATOM 1081 CA LYS A 69 -22.033 2.592 12.988 1.00 0.00 A2 C

ATOM 1082 HA LYS A 69 -23.045 2.838 13.286 0.00 0.00 A2 H

ATOM 1083 CB LYS A 69 -22.140 1.278 12.198 1.00 0.00 A2 C

ATOM 1084 HB1 LYS A 69 -22.281 0.459 12.941 0.00 0.00 A2 H

ATOM 1085 HB2 LYS A 69 -23.065 1.302 11.579 0.00 0.00 A2 H

ATOM 1086 CG LYS A 69 -20.932 0.947 11.331 1.00 0.00 A2 C

ATOM 1087 HG1 LYS A 69 -20.597 1.870 10.805 0.00 0.00 A2 H

ATOM 1088 HG2 LYS A 69 -20.095 0.616 11.987 0.00 0.00 A2 H

ATOM 1089 CD LYS A 69 -21.258 -0.131 10.294 1.00 0.00 A2 C

ATOM 1090 HD1 LYS A 69 -21.745 -0.986 10.817 0.00 0.00 A2 H

ATOM 1091 HD2 LYS A 69 -22.002 0.290 9.578 0.00 0.00 A2 H

ATOM 1092 CE LYS A 69 -19.991 -0.579 9.561 1.00 0.00 A2 C

ATOM 1093 HE1 LYS A 69 -19.437 0.302 9.166 0.00 0.00 A2 H

ATOM 1094 HE2 LYS A 69 -19.319 -1.138 10.249 0.00 0.00 A2 H

ATOM 1095 NZ LYS A 69 -20.243 -1.482 8.387 1.00 0.00 A2 N

ATOM 1096 HZ1 LYS A 69 -19.341 -1.744 7.939 0.00 0.00 A2 H

ATOM 1097 HZ2 LYS A 69 -20.731 -2.342 8.709 0.00 0.00 A2 H

ATOM 1098 HZ3 LYS A 69 -20.842 -0.989 7.694 0.00 0.00 A2 H

ATOM 1099 C LYS A 69 -21.211 2.421 14.265 1.00 0.00 A2 C

ATOM 1100 O LYS A 69 -21.341 1.409 14.952 1.00 0.00 A2 O

ATOM 1101 N THR A 70 -20.398 3.422 14.592 1.00 0.00 A2 N

ATOM 1102 HN THR A 70 -20.315 4.272 14.072 0.00 0.00 A2 H

ATOM 1103 CA THR A 70 -19.540 3.353 15.770 1.00 0.00 A2 C

ATOM 1104 HA THR A 70 -19.640 2.343 16.144 0.00 0.00 A2 H

ATOM 1105 CB THR A 70 -18.079 3.699 15.421 1.00 0.00 A2 C

ATOM 1106 HB THR A 70 -18.090 4.754 15.053 0.00 0.00 A2 H

ATOM 1107 OG1 THR A 70 -17.638 2.854 14.348 1.00 0.00 A2 O

ATOM 1108 HG1 THR A 70 -16.723 3.106 14.183 0.00 0.00 A2 H

ATOM 1109 CG2 THR A 70 -17.169 3.498 16.650 1.00 0.00 A2 C

ATOM 1110 1HG2 THR A 70 -16.138 3.853 16.440 0.00 0.00 A2 H

ATOM 1111 2HG2 THR A 70 -17.558 4.072 17.518 0.00 0.00 A2 H

ATOM 1112 3HG2 THR A 70 -17.116 2.426 16.938 0.00 0.00 A2 H

ATOM 1113 C THR A 70 -20.026 4.278 16.882 1.00 0.00 A2 C

ATOM 1114 O THR A 70 -20.052 5.498 16.719 1.00 0.00 A2 O

ATOM 1115 N PRO A 71 -20.410 3.697 18.025 1.00 0.00 A2 N

ATOM 1116 CD PRO A 71 -20.437 2.255 18.323 1.00 0.00 A2 C

ATOM 1117 HD1 PRO A 71 -21.051 2.642 17.478 0.00 0.00 A2 H

ATOM 1118 HD2 PRO A 71 -20.029 1.247 18.078 0.00 0.00 A2 H

ATOM 1119 CA PRO A 71 -20.891 4.501 19.152 1.00 0.00 A2 C

ATOM 1120 HA PRO A 71 -21.831 4.981 18.905 0.00 0.00 A2 H

ATOM 1121 CB PRO A 71 -21.031 3.469 20.277 1.00 0.00 A2 C

ATOM 1122 HB1 PRO A 71 -22.025 2.975 20.194 0.00 0.00 A2 H

ATOM 1123 HB2 PRO A 71 -20.936 3.919 21.286 0.00 0.00 A2 H

ATOM 1124 CG PRO A 71 -21.268 2.183 19.572 1.00 0.00 A2 C

ATOM 1125 HG1 PRO A 71 -21.784 1.433 20.203 0.00 0.00 A2 H

ATOM 1126 HG2 PRO A 71 -21.881 1.595 19.044 0.00 0.00 A2 H

ATOM 1127 C PRO A 71 -19.880 5.563 19.547 1.00 0.00 A2 C

ATOM 1128 O PRO A 71 -18.672 5.335 19.425 1.00 0.00 A2 O

ATOM 1129 N GLY A 72 -20.376 6.717 19.986 1.00 0.00 A2 N

ATOM 1130 HN GLY A 72 -21.354 6.918 19.941 0.00 0.00 A2 H

ATOM 1131 CA GLY A 72 -19.546 7.762 20.558 1.00 0.00 A2 C

ATOM 1132 HA1 GLY A 72 -19.138 7.353 21.472 0.00 0.00 A2 H

ATOM 1133 HA2 GLY A 72 -20.192 8.618 20.703 0.00 0.00 A2 H

ATOM 1134 C GLY A 72 -18.392 8.193 19.682 1.00 0.00 A2 C

ATOM 1135 O GLY A 72 -17.340 8.595 20.182 1.00 0.00 A2 O

ATOM 1136 N ALA A 73 -18.585 8.118 18.372 1.00 0.00 A2 N

ATOM 1137 HN ALA A 73 -19.459 7.874 17.953 0.00 0.00 A2 H

ATOM 1138 CA ALA A 73 -17.501 8.396 17.441 1.00 0.00 A2 C

ATOM 1139 HA ALA A 73 -16.548 8.146 17.893 0.00 0.00 A2 H

ATOM 1140 CB ALA A 73 -17.648 7.568 16.191 1.00 0.00 A2 C

ATOM 1141 HB1 ALA A 73 -17.652 6.489 16.457 0.00 0.00 A2 H

ATOM 1142 HB2 ALA A 73 -18.604 7.796 15.671 0.00 0.00 A2 H

ATOM 1143 HB3 ALA A 73 -16.810 7.743 15.483 0.00 0.00 A2 H

ATOM 1144 C ALA A 73 -17.427 9.860 17.072 1.00 0.00 A2 C

ATOM 1145 O ALA A 73 -18.433 10.565 17.054 1.00 0.00 A2 O

ATOM 1146 N VAL A 74 -16.215 10.316 16.789 1.00 0.00 A2 N

ATOM 1147 HN VAL A 74 -15.364 9.858 17.033 0.00 0.00 A2 H

ATOM 1148 CA VAL A 74 -16.040 11.564 16.075 1.00 0.00 A2 C

ATOM 1149 HA VAL A 74 -17.014 11.981 15.850 0.00 0.00 A2 H

ATOM 1150 CB VAL A 74 -15.237 12.605 16.890 1.00 0.00 A2 C

ATOM 1151 HB VAL A 74 -15.128 13.519 16.256 0.00 0.00 A2 H

ATOM 1152 CG1 VAL A 74 -15.981 12.962 18.164 1.00 0.00 A2 C

ATOM 1153 1HG1 VAL A 74 -15.387 13.680 18.769 0.00 0.00 A2 H

ATOM 1154 2HG1 VAL A 74 -16.958 13.442 17.947 0.00 0.00 A2 H

ATOM 1155 3HG1 VAL A 74 -16.158 12.056 18.782 0.00 0.00 A2 H

ATOM 1156 CG2 VAL A 74 -13.873 12.073 17.234 1.00 0.00 A2 C

ATOM 1157 1HG2 VAL A 74 -13.311 12.804 17.853 0.00 0.00 A2 H

ATOM 1158 2HG2 VAL A 74 -13.957 11.123 17.805 0.00 0.00 A2 H

ATOM 1159 3HG2 VAL A 74 -13.275 11.879 16.319 0.00 0.00 A2 H

ATOM 1160 C VAL A 74 -15.359 11.242 14.743 1.00 0.00 A2 C

ATOM 1161 O VAL A 74 -14.973 10.094 14.485 1.00 0.00 A2 O

ATOM 1162 N ASN A 75 -15.207 12.259 13.909 1.00 0.00 A2 N

ATOM 1163 HN ASN A 75 -15.363 13.208 14.182 0.00 0.00 A2 H

ATOM 1164 CA ASN A 75 -14.802 12.090 12.529 1.00 0.00 A2 C

ATOM 1165 HA ASN A 75 -14.314 11.124 12.453 0.00 0.00 A2 H

ATOM 1166 CB ASN A 75 -16.037 12.273 11.640 1.00 0.00 A2 C

ATOM 1167 HB1 ASN A 75 -16.772 11.484 11.912 0.00 0.00 A2 H

ATOM 1168 HB2 ASN A 75 -16.514 13.254 11.840 0.00 0.00 A2 H

ATOM 1169 CG ASN A 75 -15.736 12.136 10.163 1.00 0.00 A2 C

ATOM 1170 OD1 ASN A 75 -14.714 11.572 9.761 1.00 0.00 A2 O

ATOM 1171 ND2 ASN A 75 -16.643 12.641 9.338 1.00 0.00 A2 N

ATOM 1172 1HD2 ASN A 75 -16.472 12.553 8.360 0.00 0.00 A2 H

ATOM 1173 2HD2 ASN A 75 -17.469 13.060 9.702 0.00 0.00 A2 H

ATOM 1174 C ASN A 75 -13.764 13.159 12.206 1.00 0.00 A2 C

ATOM 1175 O ASN A 75 -14.003 14.024 11.375 1.00 0.00 A2 O

ATOM 1176 N ALA A 76 -12.611 13.078 12.859 1.00 0.00 A2 N

ATOM 1177 HN ALA A 76 -12.363 12.292 13.425 0.00 0.00 A2 H

ATOM 1178 CA ALA A 76 -11.617 14.141 12.795 1.00 0.00 A2 C

ATOM 1179 HA ALA A 76 -12.079 15.080 13.078 0.00 0.00 A2 H

ATOM 1180 CB ALA A 76 -10.517 13.925 13.831 1.00 0.00 A2 C

ATOM 1181 HB1 ALA A 76 -10.970 13.822 14.840 0.00 0.00 A2 H

ATOM 1182 HB2 ALA A 76 -9.944 12.996 13.617 0.00 0.00 A2 H

ATOM 1183 HB3 ALA A 76 -9.808 14.780 13.859 0.00 0.00 A2 H

ATOM 1184 C ALA A 76 -11.026 14.354 11.406 1.00 0.00 A2 C

ATOM 1185 O ALA A 76 -10.601 15.459 11.102 1.00 0.00 A2 O

ATOM 1186 CB CYS A 77 -9.952 12.180 8.624 1.00 0.00 A2 C

ATOM 1187 SG CYS A 77 -8.358 11.673 9.329 1.00 0.00 A2 S

ATOM 1188 N CYS A 77 -10.994 13.321 10.567 1.00 0.00 A2 N

ATOM 1189 HN CYS A 77 -11.279 12.394 10.801 0.00 0.00 A2 H

ATOM 1190 CA CYS A 77 -10.518 13.488 9.190 1.00 0.00 A2 C

ATOM 1191 HA CYS A 77 -9.721 14.221 9.240 0.00 0.00 A2 H

ATOM 1192 HB1 CYS A 77 -10.694 11.378 8.841 0.00 0.00 A2 H

ATOM 1193 HB2 CYS A 77 -9.886 12.246 7.515 0.00 0.00 A2 H

ATOM 1194 C CYS A 77 -11.597 14.037 8.255 1.00 0.00 A2 C

ATOM 1195 O CYS A 77 -11.315 14.374 7.094 1.00 0.00 A2 O

ATOM 1196 N HSD A 78 -12.822 14.133 8.769 1.00 0.00 A2 N

ATOM 1197 HN HSD A 78 -13.025 13.904 9.720 0.00 0.00 A2 H

ATOM 1198 CA HSD A 78 -13.968 14.582 7.978 1.00 0.00 A2 C

ATOM 1199 HA HSD A 78 -14.866 14.400 8.555 0.00 0.00 A2 H

ATOM 1200 CB HSD A 78 -13.846 16.074 7.632 1.00 0.00 A2 C

ATOM 1201 HB1 HSD A 78 -12.986 16.217 6.942 0.00 0.00 A2 H

ATOM 1202 HB2 HSD A 78 -14.755 16.404 7.084 0.00 0.00 A2 H

ATOM 1203 ND1 HSD A 78 -14.154 16.663 10.068 1.00 0.00 A2 N

ATOM 1204 HD1 HSD A 78 -14.627 15.821 10.329 0.00 0.00 A2 H

ATOM 1205 CG HSD A 78 -13.647 16.960 8.822 1.00 0.00 A2 C

ATOM 1206 CE1 HSD A 78 -13.823 17.618 10.917 1.00 0.00 A2 C

ATOM 1207 HE1 HSD A 78 -14.099 17.616 11.975 0.00 0.00 A2 H

ATOM 1208 NE2 HSD A 78 -13.120 18.525 10.266 1.00 0.00 A2 N

ATOM 1209 CD2 HSD A 78 -12.999 18.140 8.954 1.00 0.00 A2 C

ATOM 1210 HD2 HSD A 78 -12.494 18.738 8.205 0.00 0.00 A2 H

ATOM 1211 C HSD A 78 -14.077 13.767 6.701 1.00 0.00 A2 C

ATOM 1212 O HSD A 78 -14.007 14.314 5.598 1.00 0.00 A2 O

ATOM 1213 N LEU A 79 -14.275 12.462 6.850 1.00 0.00 A2 N

ATOM 1214 HN LEU A 79 -14.498 12.020 7.716 0.00 0.00 A2 H

ATOM 1215 CA LEU A 79 -14.178 11.554 5.726 1.00 0.00 A2 C

ATOM 1216 HA LEU A 79 -14.658 12.042 4.887 0.00 0.00 A2 H

ATOM 1217 CB LEU A 79 -12.695 11.264 5.491 1.00 0.00 A2 C

ATOM 1218 HB1 LEU A 79 -12.166 12.245 5.477 0.00 0.00 A2 H

ATOM 1219 HB2 LEU A 79 -12.291 10.710 6.367 0.00 0.00 A2 H

ATOM 1220 CG LEU A 79 -12.286 10.529 4.233 1.00 0.00 A2 C

ATOM 1221 HG LEU A 79 -12.712 9.625 4.199 0.00 0.00 A2 H

ATOM 1222 CD1 LEU A 79 -12.743 11.318 3.037 1.00 0.00 A2 C

ATOM 1223 1HD1 LEU A 79 -12.414 10.826 2.097 0.00 0.00 A2 H

ATOM 1224 2HD1 LEU A 79 -13.851 11.397 3.021 0.00 0.00 A2 H

ATOM 1225 3HD1 LEU A 79 -12.318 12.344 3.058 0.00 0.00 A2 H

ATOM 1226 CD2 LEU A 79 -10.775 10.344 4.229 1.00 0.00 A2 C

ATOM 1227 1HD2 LEU A 79 -10.457 9.789 3.320 0.00 0.00 A2 H

ATOM 1228 2HD2 LEU A 79 -10.243 11.317 4.233 0.00 0.00 A2 H

ATOM 1229 3HD2 LEU A 79 -10.452 9.765 5.121 0.00 0.00 A2 H

ATOM 1230 C LEU A 79 -14.941 10.254 6.003 1.00 0.00 A2 C

ATOM 1231 O LEU A 79 -14.984 9.787 7.138 1.00 0.00 A2 O

ATOM 1232 N SER A 80 -15.543 9.665 4.974 1.00 0.00 A2 N

ATOM 1233 HN SER A 80 -15.629 10.052 4.056 0.00 0.00 A2 H

ATOM 1234 CA SER A 80 -16.151 8.341 5.127 1.00 0.00 A2 C

ATOM 1235 HA SER A 80 -16.689 8.358 6.066 0.00 0.00 A2 H

ATOM 1236 CB SER A 80 -17.032 7.992 3.928 1.00 0.00 A2 C

ATOM 1237 HB1 SER A 80 -16.382 7.939 3.025 0.00 0.00 A2 H

ATOM 1238 HB2 SER A 80 -17.488 6.985 4.062 0.00 0.00 A2 H

ATOM 1239 OG SER A 80 -18.022 8.986 3.726 1.00 0.00 A2 O

ATOM 1240 HG1 SER A 80 -18.396 8.840 2.848 0.00 0.00 A2 H

ATOM 1241 C SER A 80 -15.075 7.273 5.242 1.00 0.00 A2 C

ATOM 1242 O SER A 80 -14.084 7.298 4.522 1.00 0.00 A2 O

ATOM 1243 SG CYS A 81 -14.561 5.128 9.015 1.00 0.00 A2 S

ATOM 1244 CB CYS A 81 -14.722 4.304 7.407 1.00 0.00 A2 C

ATOM 1245 N CYS A 81 -15.286 6.310 6.124 1.00 0.00 A2 N

ATOM 1246 HN CYS A 81 -16.063 6.258 6.749 0.00 0.00 A2 H

ATOM 1247 CA CYS A 81 -14.340 5.211 6.236 1.00 0.00 A2 C

ATOM 1248 HA CYS A 81 -13.379 5.665 6.447 0.00 0.00 A2 H

ATOM 1249 HB1 CYS A 81 -15.784 4.004 7.259 0.00 0.00 A2 H

ATOM 1250 HB2 CYS A 81 -14.129 3.364 7.365 0.00 0.00 A2 H

ATOM 1251 C CYS A 81 -14.206 4.420 4.925 1.00 0.00 A2 C

ATOM 1252 O CYS A 81 -13.170 3.796 4.670 1.00 0.00 A2 O

ATOM 1253 N SER A 82 -15.236 4.454 4.084 1.00 0.00 A2 N

ATOM 1254 HN SER A 82 -16.091 4.951 4.230 0.00 0.00 A2 H

ATOM 1255 CA SER A 82 -15.177 3.721 2.815 1.00 0.00 A2 C

ATOM 1256 HA SER A 82 -14.878 2.712 3.067 0.00 0.00 A2 H

ATOM 1257 CB SER A 82 -16.551 3.676 2.117 1.00 0.00 A2 C

ATOM 1258 HB1 SER A 82 -16.453 3.051 1.200 0.00 0.00 A2 H

ATOM 1259 HB2 SER A 82 -17.303 3.180 2.772 0.00 0.00 A2 H

ATOM 1260 OG SER A 82 -16.979 4.973 1.740 1.00 0.00 A2 O

ATOM 1261 HG1 SER A 82 -17.714 4.861 1.124 0.00 0.00 A2 H

ATOM 1262 C SER A 82 -14.096 4.300 1.885 1.00 0.00 A2 C

ATOM 1263 O SER A 82 -13.484 3.589 1.094 1.00 0.00 A2 O

ATOM 1264 N ALA A 83 -13.846 5.594 2.008 1.00 0.00 A2 N

ATOM 1265 HN ALA A 83 -14.370 6.197 2.608 0.00 0.00 A2 H

ATOM 1266 CA ALA A 83 -12.771 6.234 1.263 1.00 0.00 A2 C

ATOM 1267 HA ALA A 83 -12.897 6.066 0.200 0.00 0.00 A2 H

ATOM 1268 CB ALA A 83 -12.789 7.754 1.516 1.00 0.00 A2 C

ATOM 1269 HB1 ALA A 83 -13.768 8.173 1.198 0.00 0.00 A2 H

ATOM 1270 HB2 ALA A 83 -12.654 7.981 2.596 0.00 0.00 A2 H

ATOM 1271 HB3 ALA A 83 -11.991 8.274 0.943 0.00 0.00 A2 H

ATOM 1272 C ALA A 83 -11.402 5.652 1.633 1.00 0.00 A2 C

ATOM 1273 O ALA A 83 -10.449 5.782 0.865 1.00 0.00 A2 O

ATOM 1274 N LEU A 84 -11.302 5.026 2.808 1.00 0.00 A2 N

ATOM 1275 HN LEU A 84 -12.052 4.954 3.462 0.00 0.00 A2 H

ATOM 1276 CA LEU A 84 -10.052 4.377 3.245 1.00 0.00 A2 C

ATOM 1277 HA LEU A 84 -9.252 4.926 2.765 0.00 0.00 A2 H

ATOM 1278 CB LEU A 84 -9.903 4.458 4.765 1.00 0.00 A2 C

ATOM 1279 HB1 LEU A 84 -10.691 3.806 5.208 0.00 0.00 A2 H

ATOM 1280 HB2 LEU A 84 -8.929 4.007 5.058 0.00 0.00 A2 H

ATOM 1281 CG LEU A 84 -10.049 5.878 5.295 1.00 0.00 A2 C

ATOM 1282 HG LEU A 84 -10.978 6.180 5.081 0.00 0.00 A2 H

ATOM 1283 CD1 LEU A 84 -9.906 5.958 6.815 1.00 0.00 A2 C

ATOM 1284 1HD1 LEU A 84 -9.970 7.013 7.158 0.00 0.00 A2 H

ATOM 1285 2HD1 LEU A 84 -10.712 5.379 7.315 0.00 0.00 A2 H

ATOM 1286 3HD1 LEU A 84 -8.926 5.548 7.139 0.00 0.00 A2 H

ATOM 1287 CD2 LEU A 84 -9.058 6.793 4.612 1.00 0.00 A2 C

ATOM 1288 1HD2 LEU A 84 -9.175 7.834 4.983 0.00 0.00 A2 H

ATOM 1289 2HD2 LEU A 84 -8.012 6.482 4.806 0.00 0.00 A2 H

ATOM 1290 3HD2 LEU A 84 -9.224 6.795 3.513 0.00 0.00 A2 H

ATOM 1291 C LEU A 84 -9.941 2.911 2.798 1.00 0.00 A2 C

ATOM 1292 O LEU A 84 -9.006 2.210 3.178 1.00 0.00 A2 O

ATOM 1293 N LEU A 85 -10.883 2.456 1.980 1.00 0.00 A2 N

ATOM 1294 HN LEU A 85 -11.658 2.991 1.649 0.00 0.00 A2 H

ATOM 1295 CA LEU A 85 -10.848 1.082 1.488 1.00 0.00 A2 C

ATOM 1296 HA LEU A 85 -9.983 0.613 1.938 0.00 0.00 A2 H

ATOM 1297 CB LEU A 85 -12.125 0.336 1.884 1.00 0.00 A2 C

ATOM 1298 HB1 LEU A 85 -12.986 0.925 1.491 0.00 0.00 A2 H

ATOM 1299 HB2 LEU A 85 -12.153 -0.641 1.353 0.00 0.00 A2 H

ATOM 1300 CG LEU A 85 -12.354 0.112 3.377 1.00 0.00 A2 C

ATOM 1301 HG LEU A 85 -12.382 1.000 3.835 0.00 0.00 A2 H

ATOM 1302 CD1 LEU A 85 -13.680 -0.601 3.595 1.00 0.00 A2 C

ATOM 1303 1HD1 LEU A 85 -13.836 -0.813 4.675 0.00 0.00 A2 H

ATOM 1304 2HD1 LEU A 85 -14.525 0.026 3.237 0.00 0.00 A2 H

ATOM 1305 3HD1 LEU A 85 -13.701 -1.566 3.046 0.00 0.00 A2 H

ATOM 1306 CD2 LEU A 85 -11.204 -0.708 3.980 1.00 0.00 A2 C

ATOM 1307 1HD2 LEU A 85 -11.364 -0.853 5.070 0.00 0.00 A2 H

ATOM 1308 2HD2 LEU A 85 -11.128 -1.711 3.514 0.00 0.00 A2 H

ATOM 1309 3HD2 LEU A 85 -10.235 -0.182 3.838 0.00 0.00 A2 H

ATOM 1310 C LEU A 85 -10.659 1.041 -0.026 1.00 0.00 A2 C

ATOM 1311 O LEU A 85 -11.024 0.071 -0.685 1.00 0.00 A2 O

ATOM 1312 N GLN A 86 -10.073 2.093 -0.578 1.00 0.00 A2 N

ATOM 1313 HN GLN A 86 -9.688 2.863 -0.072 0.00 0.00 A2 H

ATOM 1314 CA GLN A 86 -9.947 2.199 -2.025 1.00 0.00 A2 C

ATOM 1315 HA GLN A 86 -10.616 1.455 -2.439 0.00 0.00 A2 H

ATOM 1316 CB GLN A 86 -10.414 3.573 -2.486 1.00 0.00 A2 C

ATOM 1317 HB1 GLN A 86 -9.772 4.322 -1.967 0.00 0.00 A2 H

ATOM 1318 HB2 GLN A 86 -10.207 3.687 -3.575 0.00 0.00 A2 H

ATOM 1319 CG GLN A 86 -11.896 3.833 -2.163 1.00 0.00 A2 C

ATOM 1320 HG1 GLN A 86 -12.040 3.941 -1.067 0.00 0.00 A2 H

ATOM 1321 HG2 GLN A 86 -12.245 4.769 -2.646 0.00 0.00 A2 H

ATOM 1322 CD GLN A 86 -12.814 2.675 -2.609 1.00 0.00 A2 C

ATOM 1323 OE1 GLN A 86 -12.759 2.224 -3.757 1.00 0.00 A2 O

ATOM 1324 NE2 GLN A 86 -13.672 2.208 -1.697 1.00 0.00 A2 N

ATOM 1325 1HE2 GLN A 86 -14.264 1.458 -1.978 0.00 0.00 A2 H

ATOM 1326 2HE2 GLN A 86 -13.707 2.621 -0.793 0.00 0.00 A2 H

ATOM 1327 C GLN A 86 -8.544 1.887 -2.530 1.00 0.00 A2 C

ATOM 1328 O GLN A 86 -7.565 2.003 -1.794 1.00 0.00 A2 O

ATOM 1329 N ASP A 87 -8.456 1.480 -3.793 1.00 0.00 A2 N

ATOM 1330 HN ASP A 87 -9.224 1.359 -4.417 0.00 0.00 A2 H

ATOM 1331 CA ASP A 87 -7.175 1.162 -4.402 1.00 0.00 A2 C

ATOM 1332 HA ASP A 87 -6.670 0.461 -3.747 0.00 0.00 A2 H

ATOM 1333 CB ASP A 87 -7.377 0.581 -5.803 1.00 0.00 A2 C

ATOM 1334 HB1 ASP A 87 -8.140 1.180 -6.340 0.00 0.00 A2 H

ATOM 1335 HB2 ASP A 87 -6.433 0.619 -6.382 0.00 0.00 A2 H

ATOM 1336 CG ASP A 87 -7.867 -0.858 -5.773 1.00 0.00 A2 C

ATOM 1337 OD1 ASP A 87 -8.090 -1.451 -4.574 1.00 0.00 A2 O

ATOM 1338 OD2 ASP A 87 -8.042 -1.500 -6.954 1.00 0.00 A2 O

ATOM 1339 C ASP A 87 -6.301 2.403 -4.474 1.00 0.00 A2 C

ATOM 1340 O ASP A 87 -5.080 2.329 -4.310 1.00 0.00 A2 O

ATOM 1341 N ASN A 88 -6.930 3.538 -4.748 1.00 0.00 A2 N

ATOM 1342 HN ASN A 88 -7.897 3.596 -4.992 0.00 0.00 A2 H

ATOM 1343 CA ASN A 88 -6.236 4.822 -4.713 1.00 0.00 A2 C

ATOM 1344 HA ASN A 88 -5.237 4.650 -5.099 0.00 0.00 A2 H

ATOM 1345 CB ASN A 88 -7.009 5.844 -5.546 1.00 0.00 A2 C

ATOM 1346 HB1 ASN A 88 -7.001 5.498 -6.603 0.00 0.00 A2 H

ATOM 1347 HB2 ASN A 88 -8.068 5.887 -5.218 0.00 0.00 A2 H

ATOM 1348 CG ASN A 88 -6.390 7.231 -5.502 1.00 0.00 A2 C

ATOM 1349 OD1 ASN A 88 -5.974 7.709 -4.449 1.00 0.00 A2 O

ATOM 1350 ND2 ASN A 88 -6.339 7.887 -6.651 1.00 0.00 A2 N

ATOM 1351 1HD2 ASN A 88 -5.926 8.794 -6.643 0.00 0.00 A2 H

ATOM 1352 2HD2 ASN A 88 -6.678 7.461 -7.484 0.00 0.00 A2 H

ATOM 1353 C ASN A 88 -6.098 5.278 -3.242 1.00 0.00 A2 C

ATOM 1354 O ASN A 88 -7.090 5.488 -2.554 1.00 0.00 A2 O

ATOM 1355 N ILE A 89 -4.870 5.383 -2.750 1.00 0.00 A2 N

ATOM 1356 HN ILE A 89 -4.028 5.291 -3.278 0.00 0.00 A2 H

ATOM 1357 CA ILE A 89 -4.659 5.654 -1.330 1.00 0.00 A2 C

ATOM 1358 HA ILE A 89 -5.576 5.335 -0.851 0.00 0.00 A2 H

ATOM 1359 CB ILE A 89 -3.474 4.819 -0.761 1.00 0.00 A2 C

ATOM 1360 HB ILE A 89 -3.441 5.084 0.326 0.00 0.00 A2 H

ATOM 1361 CG2 ILE A 89 -3.698 3.335 -1.026 1.00 0.00 A2 C

ATOM 1362 1HG2 ILE A 89 -2.942 2.724 -0.491 0.00 0.00 A2 H

ATOM 1363 2HG2 ILE A 89 -4.701 3.016 -0.671 0.00 0.00 A2 H

ATOM 1364 3HG2 ILE A 89 -3.622 3.109 -2.111 0.00 0.00 A2 H

ATOM 1365 CG1 ILE A 89 -2.146 5.273 -1.361 1.00 0.00 A2 C

ATOM 1366 1HG1 ILE A 89 -2.165 5.136 -2.465 0.00 0.00 A2 H

ATOM 1367 2HG1 ILE A 89 -2.016 6.362 -1.167 0.00 0.00 A2 H

ATOM 1368 CD ILE A 89 -0.916 4.522 -0.774 1.00 0.00 A2 C

ATOM 1369 HD1 ILE A 89 0.025 4.930 -1.201 0.00 0.00 A2 H

ATOM 1370 HD2 ILE A 89 -0.876 4.639 0.330 0.00 0.00 A2 H

ATOM 1371 HD3 ILE A 89 -0.960 3.439 -1.013 0.00 0.00 A2 H

ATOM 1372 C ILE A 89 -4.463 7.138 -0.972 1.00 0.00 A2 C

ATOM 1373 O ILE A 89 -4.034 7.451 0.135 1.00 0.00 A2 O

ATOM 1374 N ALA A 90 -4.783 8.055 -1.884 1.00 0.00 A2 N

ATOM 1375 HN ALA A 90 -5.121 7.848 -2.801 0.00 0.00 A2 H

ATOM 1376 CA ALA A 90 -4.647 9.487 -1.569 1.00 0.00 A2 C

ATOM 1377 HA ALA A 90 -3.603 9.704 -1.372 0.00 0.00 A2 H

ATOM 1378 CB ALA A 90 -4.978 10.357 -2.791 1.00 0.00 A2 C

ATOM 1379 HB1 ALA A 90 -4.364 10.031 -3.658 0.00 0.00 A2 H

ATOM 1380 HB2 ALA A 90 -6.049 10.258 -3.074 0.00 0.00 A2 H

ATOM 1381 HB3 ALA A 90 -4.760 11.430 -2.600 0.00 0.00 A2 H

ATOM 1382 C ALA A 90 -5.434 9.956 -0.321 1.00 0.00 A2 C

ATOM 1383 O ALA A 90 -4.903 10.722 0.486 1.00 0.00 A2 O

ATOM 1384 N ASP A 91 -6.689 9.522 -0.165 1.00 0.00 A2 N

ATOM 1385 HN ASP A 91 -7.188 8.938 -0.800 0.00 0.00 A2 H

ATOM 1386 CA ASP A 91 -7.482 9.890 1.023 1.00 0.00 A2 C

ATOM 1387 HA ASP A 91 -7.413 10.967 1.127 0.00 0.00 A2 H

ATOM 1388 CB ASP A 91 -8.938 9.416 0.910 1.00 0.00 A2 C

ATOM 1389 HB1 ASP A 91 -8.952 8.341 0.639 0.00 0.00 A2 H

ATOM 1390 HB2 ASP A 91 -9.465 9.539 1.877 0.00 0.00 A2 H

ATOM 1391 CG ASP A 91 -9.739 10.160 -0.166 1.00 0.00 A2 C

ATOM 1392 OD1 ASP A 91 -9.106 10.657 -1.257 1.00 0.00 A2 O

ATOM 1393 OD2 ASP A 91 -11.081 10.269 -0.007 1.00 0.00 A2 O

ATOM 1394 C ASP A 91 -6.896 9.287 2.295 1.00 0.00 A2 C

ATOM 1395 O ASP A 91 -6.828 9.943 3.338 1.00 0.00 A2 O

ATOM 1396 N ALA A 92 -6.510 8.018 2.220 1.00 0.00 A2 N

ATOM 1397 HN ALA A 92 -6.629 7.450 1.406 0.00 0.00 A2 H

ATOM 1398 CA ALA A 92 -5.871 7.360 3.356 1.00 0.00 A2 C

ATOM 1399 HA ALA A 92 -6.522 7.379 4.222 0.00 0.00 A2 H

ATOM 1400 CB ALA A 92 -5.585 5.893 3.036 1.00 0.00 A2 C

ATOM 1401 HB1 ALA A 92 -6.535 5.373 2.790 0.00 0.00 A2 H

ATOM 1402 HB2 ALA A 92 -4.907 5.800 2.159 0.00 0.00 A2 H

ATOM 1403 HB3 ALA A 92 -5.121 5.369 3.899 0.00 0.00 A2 H

ATOM 1404 C ALA A 92 -4.591 8.082 3.764 1.00 0.00 A2 C

ATOM 1405 O ALA A 92 -4.319 8.279 4.955 1.00 0.00 A2 O

ATOM 1406 N VAL A 93 -3.788 8.463 2.777 1.00 0.00 A2 N

ATOM 1407 HN VAL A 93 -3.947 8.286 1.809 0.00 0.00 A2 H

ATOM 1408 CA VAL A 93 -2.562 9.201 3.061 1.00 0.00 A2 C

ATOM 1409 HA VAL A 93 -1.996 8.624 3.781 0.00 0.00 A2 H

ATOM 1410 CB VAL A 93 -1.698 9.393 1.794 1.00 0.00 A2 C

ATOM 1411 HB VAL A 93 -2.376 9.725 0.970 0.00 0.00 A2 H

ATOM 1412 CG1 VAL A 93 -0.643 10.455 2.037 1.00 0.00 A2 C

ATOM 1413 1HG1 VAL A 93 0.005 10.567 1.141 0.00 0.00 A2 H

ATOM 1414 2HG1 VAL A 93 -1.099 11.446 2.242 0.00 0.00 A2 H

ATOM 1415 3HG1 VAL A 93 0.004 10.176 2.896 0.00 0.00 A2 H

ATOM 1416 CG2 VAL A 93 -1.030 8.060 1.378 1.00 0.00 A2 C

ATOM 1417 1HG2 VAL A 93 -0.378 8.207 0.491 0.00 0.00 A2 H

ATOM 1418 2HG2 VAL A 93 -0.407 7.660 2.207 0.00 0.00 A2 H

ATOM 1419 3HG2 VAL A 93 -1.789 7.296 1.112 0.00 0.00 A2 H

ATOM 1420 C VAL A 93 -2.874 10.566 3.704 1.00 0.00 A2 C

ATOM 1421 O VAL A 93 -2.202 10.976 4.641 1.00 0.00 A2 O

ATOM 1422 N ALA A 94 -3.885 11.269 3.196 1.00 0.00 A2 N

ATOM 1423 HN ALA A 94 -4.443 10.967 2.424 0.00 0.00 A2 H

ATOM 1424 CA ALA A 94 -4.248 12.572 3.763 1.00 0.00 A2 C

ATOM 1425 HA ALA A 94 -3.399 13.246 3.774 0.00 0.00 A2 H

ATOM 1426 CB ALA A 94 -5.358 13.246 2.941 1.00 0.00 A2 C

ATOM 1427 HB1 ALA A 94 -5.005 13.407 1.900 0.00 0.00 A2 H

ATOM 1428 HB2 ALA A 94 -6.267 12.608 2.899 0.00 0.00 A2 H

ATOM 1429 HB3 ALA A 94 -5.639 14.234 3.365 0.00 0.00 A2 H

ATOM 1430 C ALA A 94 -4.680 12.399 5.219 1.00 0.00 A2 C

ATOM 1431 O ALA A 94 -4.348 13.219 6.077 1.00 0.00 A2 O

ATOM 1432 SG CYS A 95 -7.811 9.894 8.503 1.00 0.00 A2 S

ATOM 1433 CB CYS A 95 -7.027 10.037 6.872 1.00 0.00 A2 C

ATOM 1434 N CYS A 95 -5.413 11.325 5.500 1.00 0.00 A2 N

ATOM 1435 HN CYS A 95 -5.677 10.633 4.831 0.00 0.00 A2 H

ATOM 1436 CA CYS A 95 -5.899 11.080 6.862 1.00 0.00 A2 C

ATOM 1437 HA CYS A 95 -6.288 12.026 7.220 0.00 0.00 A2 H

ATOM 1438 HB1 CYS A 95 -7.787 10.365 6.127 0.00 0.00 A2 H

ATOM 1439 HB2 CYS A 95 -6.641 9.060 6.506 0.00 0.00 A2 H

ATOM 1440 C CYS A 95 -4.762 10.657 7.802 1.00 0.00 A2 C

ATOM 1441 O CYS A 95 -4.707 11.095 8.960 1.00 0.00 A2 O

ATOM 1442 N ALA A 96 -3.849 9.821 7.307 1.00 0.00 A2 N

ATOM 1443 HN ALA A 96 -3.902 9.411 6.397 0.00 0.00 A2 H

ATOM 1444 CA ALA A 96 -2.676 9.444 8.099 1.00 0.00 A2 C

ATOM 1445 HA ALA A 96 -2.973 8.984 9.034 0.00 0.00 A2 H

ATOM 1446 CB ALA A 96 -1.816 8.459 7.331 1.00 0.00 A2 C

ATOM 1447 HB1 ALA A 96 -2.404 7.543 7.111 0.00 0.00 A2 H

ATOM 1448 HB2 ALA A 96 -1.482 8.891 6.363 0.00 0.00 A2 H

ATOM 1449 HB3 ALA A 96 -0.918 8.159 7.913 0.00 0.00 A2 H

ATOM 1450 C ALA A 96 -1.856 10.686 8.469 1.00 0.00 A2 C

ATOM 1451 O ALA A 96 -1.340 10.798 9.582 1.00 0.00 A2 O

ATOM 1452 N LYS A 97 -1.720 11.611 7.522 1.00 0.00 A2 N

ATOM 1453 HN LYS A 97 -2.056 11.507 6.587 0.00 0.00 A2 H

ATOM 1454 CA LYS A 97 -1.051 12.873 7.801 1.00 0.00 A2 C

ATOM 1455 HA LYS A 97 -0.053 12.638 8.151 0.00 0.00 A2 H

ATOM 1456 CB LYS A 97 -0.976 13.740 6.540 1.00 0.00 A2 C

ATOM 1457 HB1 LYS A 97 -1.979 13.702 6.056 0.00 0.00 A2 H

ATOM 1458 HB2 LYS A 97 -0.813 14.800 6.838 0.00 0.00 A2 H

ATOM 1459 CG LYS A 97 0.076 13.310 5.518 1.00 0.00 A2 C

ATOM 1460 HG1 LYS A 97 1.090 13.453 5.957 0.00 0.00 A2 H

ATOM 1461 HG2 LYS A 97 -0.045 12.222 5.309 0.00 0.00 A2 H

ATOM 1462 CD LYS A 97 -0.060 14.143 4.233 1.00 0.00 A2 C

ATOM 1463 HD1 LYS A 97 -1.109 14.056 3.867 0.00 0.00 A2 H

ATOM 1464 HD2 LYS A 97 0.115 15.213 4.493 0.00 0.00 A2 H

ATOM 1465 CE LYS A 97 0.923 13.697 3.153 1.00 0.00 A2 C

ATOM 1466 HE1 LYS A 97 1.972 13.845 3.495 0.00 0.00 A2 H

ATOM 1467 HE2 LYS A 97 0.780 12.620 2.915 0.00 0.00 A2 H

ATOM 1468 NZ LYS A 97 0.723 14.492 1.888 1.00 0.00 A2 N

ATOM 1469 HZ1 LYS A 97 1.399 14.180 1.161 0.00 0.00 A2 H

ATOM 1470 HZ2 LYS A 97 -0.247 14.353 1.540 0.00 0.00 A2 H

ATOM 1471 HZ3 LYS A 97 0.873 15.502 2.087 0.00 0.00 A2 H

ATOM 1472 C LYS A 97 -1.759 13.625 8.920 1.00 0.00 A2 C

ATOM 1473 O LYS A 97 -1.113 14.182 9.800 1.00 0.00 A2 O

ATOM 1474 N ARG A 98 -3.090 13.656 8.880 1.00 0.00 A2 N

ATOM 1475 HN ARG A 98 -3.615 13.205 8.162 0.00 0.00 A2 H

ATOM 1476 CA ARG A 98 -3.853 14.364 9.904 1.00 0.00 A2 C

ATOM 1477 HA ARG A 98 -3.476 15.377 9.982 0.00 0.00 A2 H

ATOM 1478 CB ARG A 98 -5.341 14.383 9.576 1.00 0.00 A2 C

ATOM 1479 HB1 ARG A 98 -5.469 15.106 8.735 0.00 0.00 A2 H

ATOM 1480 HB2 ARG A 98 -5.647 13.391 9.176 0.00 0.00 A2 H

ATOM 1481 CG ARG A 98 -6.235 14.822 10.756 1.00 0.00 A2 C

ATOM 1482 HG1 ARG A 98 -7.288 14.552 10.522 0.00 0.00 A2 H

ATOM 1483 HG2 ARG A 98 -5.966 14.223 11.656 0.00 0.00 A2 H

ATOM 1484 CD ARG A 98 -6.072 16.310 11.096 1.00 0.00 A2 C

ATOM 1485 HD1 ARG A 98 -5.015 16.509 11.389 0.00 0.00 A2 H

ATOM 1486 HD2 ARG A 98 -6.331 16.959 10.227 0.00 0.00 A2 H

ATOM 1487 NE ARG A 98 -6.950 16.729 12.195 1.00 0.00 A2 N

ATOM 1488 HE ARG A 98 -7.797 17.171 11.878 0.00 0.00 A2 H

ATOM 1489 CZ ARG A 98 -6.672 16.551 13.480 1.00 0.00 A2 C

ATOM 1490 NH1 ARG A 98 -5.461 15.924 13.861 1.00 0.00 A2 N

ATOM 1491 1HH1 ARG A 98 -5.255 15.763 14.816 0.00 0.00 A2 H

ATOM 1492 2HH1 ARG A 98 -4.908 15.539 13.119 0.00 0.00 A2 H

ATOM 1493 NH2 ARG A 98 -7.582 16.996 14.470 1.00 0.00 A2 N

ATOM 1494 1HH2 ARG A 98 -7.336 16.942 15.427 0.00 0.00 A2 H

ATOM 1495 2HH2 ARG A 98 -8.350 17.540 14.160 0.00 0.00 A2 H

ATOM 1496 C ARG A 98 -3.641 13.715 11.262 1.00 0.00 A2 C

ATOM 1497 O ARG A 98 -3.508 14.405 12.272 1.00 0.00 A2 O

ATOM 1498 N VAL A 99 -3.601 12.389 11.279 1.00 0.00 A2 N

ATOM 1499 HN VAL A 99 -3.739 11.810 10.479 0.00 0.00 A2 H

ATOM 1500 CA VAL A 99 -3.343 11.659 12.513 1.00 0.00 A2 C

ATOM 1501 HA VAL A 99 -4.135 11.910 13.207 0.00 0.00 A2 H

ATOM 1502 CB VAL A 99 -3.336 10.129 12.301 1.00 0.00 A2 C

ATOM 1503 HB VAL A 99 -2.624 9.907 11.469 0.00 0.00 A2 H

ATOM 1504 CG1 VAL A 99 -2.864 9.409 13.578 1.00 0.00 A2 C

ATOM 1505 1HG1 VAL A 99 -2.898 8.307 13.434 0.00 0.00 A2 H

ATOM 1506 2HG1 VAL A 99 -1.819 9.677 13.837 0.00 0.00 A2 H

ATOM 1507 3HG1 VAL A 99 -3.520 9.664 14.438 0.00 0.00 A2 H

ATOM 1508 CG2 VAL A 99 -4.726 9.643 11.907 1.00 0.00 A2 C

ATOM 1509 1HG2 VAL A 99 -4.738 8.538 11.792 0.00 0.00 A2 H

ATOM 1510 2HG2 VAL A 99 -5.471 9.922 12.683 0.00 0.00 A2 H

ATOM 1511 3HG2 VAL A 99 -5.047 10.084 10.941 0.00 0.00 A2 H

ATOM 1512 C VAL A 99 -2.023 12.090 13.148 1.00 0.00 A2 C

ATOM 1513 O VAL A 99 -1.982 12.409 14.332 1.00 0.00 A2 O

ATOM 1514 N VAL A 100 -0.941 12.109 12.375 1.00 0.00 A2 N

ATOM 1515 HN VAL A 100 -0.913 11.954 11.391 0.00 0.00 A2 H

ATOM 1516 CA VAL A 100 0.362 12.380 12.982 1.00 0.00 A2 C

ATOM 1517 HA VAL A 100 0.371 11.867 13.936 0.00 0.00 A2 H

ATOM 1518 CB VAL A 100 1.553 11.784 12.191 1.00 0.00 A2 C

ATOM 1519 HB VAL A 100 2.490 12.055 12.737 0.00 0.00 A2 H

ATOM 1520 CG1 VAL A 100 1.417 10.275 12.129 1.00 0.00 A2 C

ATOM 1521 1HG1 VAL A 100 2.245 9.838 11.530 0.00 0.00 A2 H

ATOM 1522 2HG1 VAL A 100 1.462 9.818 13.139 0.00 0.00 A2 H

ATOM 1523 3HG1 VAL A 100 0.457 9.987 11.650 0.00 0.00 A2 H

ATOM 1524 CG2 VAL A 100 1.648 12.393 10.783 1.00 0.00 A2 C

ATOM 1525 1HG2 VAL A 100 2.480 11.931 10.210 0.00 0.00 A2 H

ATOM 1526 2HG2 VAL A 100 0.703 12.228 10.222 0.00 0.00 A2 H

ATOM 1527 3HG2 VAL A 100 1.840 13.485 10.830 0.00 0.00 A2 H

ATOM 1528 C VAL A 100 0.592 13.863 13.257 1.00 0.00 A2 C

ATOM 1529 O VAL A 100 1.557 14.228 13.938 1.00 0.00 A2 O

ATOM 1530 N ASP A 101 -0.296 14.714 12.752 1.00 0.00 A2 N

ATOM 1531 HN ASP A 101 -1.032 14.502 12.114 0.00 0.00 A2 H

ATOM 1532 CA ASP A 101 -0.254 16.124 13.123 1.00 0.00 A2 C

ATOM 1533 HA ASP A 101 0.789 16.420 13.100 0.00 0.00 A2 H

ATOM 1534 CB ASP A 101 -1.059 16.978 12.143 1.00 0.00 A2 C

ATOM 1535 HB1 ASP A 101 -2.021 16.473 11.923 0.00 0.00 A2 H

ATOM 1536 HB2 ASP A 101 -1.279 17.972 12.581 0.00 0.00 A2 H

ATOM 1537 CG ASP A 101 -0.336 17.170 10.842 1.00 0.00 A2 C

ATOM 1538 OD1 ASP A 101 1.015 17.052 10.853 1.00 0.00 A2 O

ATOM 1539 OD2 ASP A 101 -1.054 17.446 9.726 1.00 0.00 A2 O

ATOM 1540 C ASP A 101 -0.744 16.357 14.542 1.00 0.00 A2 C

ATOM 1541 O ASP A 101 -0.547 17.449 15.093 1.00 0.00 A2 O

ATOM 1542 N ASP A 102 -1.414 15.363 15.123 1.00 0.00 A2 N

ATOM 1543 HN ASP A 102 -1.721 14.518 14.693 0.00 0.00 A2 H

ATOM 1544 CA ASP A 102 -1.776 15.446 16.545 1.00 0.00 A2 C

ATOM 1545 HA ASP A 102 -2.137 16.450 16.740 0.00 0.00 A2 H

ATOM 1546 CB ASP A 102 -2.854 14.414 16.910 1.00 0.00 A2 C

ATOM 1547 HB1 ASP A 102 -2.854 13.603 16.154 0.00 0.00 A2 H

ATOM 1548 HB2 ASP A 102 -2.646 13.966 17.902 0.00 0.00 A2 H

ATOM 1549 CG ASP A 102 -4.254 15.024 16.932 1.00 0.00 A2 C

ATOM 1550 OD1 ASP A 102 -4.423 16.256 16.390 1.00 0.00 A2 O

ATOM 1551 OD2 ASP A 102 -5.274 14.328 17.494 1.00 0.00 A2 O

ATOM 1552 C ASP A 102 -0.521 15.258 17.390 1.00 0.00 A2 C

ATOM 1553 O ASP A 102 0.475 14.752 16.885 1.00 0.00 A2 O

ATOM 1554 N PRO A 103 -0.561 15.672 18.672 1.00 0.00 A2 N

ATOM 1555 CD PRO A 103 -1.749 16.256 19.331 1.00 0.00 A2 C

ATOM 1556 HD1 PRO A 103 -1.337 16.773 18.435 0.00 0.00 A2 H

ATOM 1557 HD2 PRO A 103 -2.862 16.320 19.341 0.00 0.00 A2 H

ATOM 1558 CA PRO A 103 0.616 15.685 19.556 1.00 0.00 A2 C

ATOM 1559 HA PRO A 103 1.268 16.486 19.227 0.00 0.00 A2 H

ATOM 1560 CB PRO A 103 0.022 16.028 20.927 1.00 0.00 A2 C

ATOM 1561 HB1 PRO A 103 -0.067 17.134 21.017 0.00 0.00 A2 H

ATOM 1562 HB2 PRO A 103 0.636 15.646 21.768 0.00 0.00 A2 H

ATOM 1563 CG PRO A 103 -1.183 16.875 20.593 1.00 0.00 A2 C

ATOM 1564 HG1 PRO A 103 -1.490 17.537 21.427 0.00 0.00 A2 H

ATOM 1565 HG2 PRO A 103 -1.529 17.752 20.261 0.00 0.00 A2 H

ATOM 1566 C PRO A 103 1.425 14.386 19.646 1.00 0.00 A2 C

ATOM 1567 O PRO A 103 2.646 14.473 19.678 1.00 0.00 A2 O

ATOM 1568 N GLN A 104 0.795 13.216 19.695 1.00 0.00 A2 N

ATOM 1569 HN GLN A 104 -0.188 13.065 19.607 0.00 0.00 A2 H

ATOM 1570 CA GLN A 104 1.583 11.992 19.897 1.00 0.00 A2 C

ATOM 1571 HA GLN A 104 2.410 12.278 20.535 0.00 0.00 A2 H

ATOM 1572 CB GLN A 104 0.808 10.933 20.694 1.00 0.00 A2 C

ATOM 1573 HB1 GLN A 104 1.566 10.258 21.154 0.00 0.00 A2 H

ATOM 1574 HB2 GLN A 104 0.276 11.425 21.540 0.00 0.00 A2 H

ATOM 1575 CG GLN A 104 -0.154 10.108 19.878 1.00 0.00 A2 C

ATOM 1576 HG1 GLN A 104 -0.952 10.753 19.452 0.00 0.00 A2 H

ATOM 1577 HG2 GLN A 104 0.370 9.613 19.035 0.00 0.00 A2 H

ATOM 1578 CD GLN A 104 -0.859 9.044 20.707 1.00 0.00 A2 C

ATOM 1579 OE1 GLN A 104 -0.253 8.397 21.556 1.00 0.00 A2 O

ATOM 1580 NE2 GLN A 104 -2.147 8.853 20.448 1.00 0.00 A2 N

ATOM 1581 1HE2 GLN A 104 -2.617 8.161 20.989 0.00 0.00 A2 H

ATOM 1582 2HE2 GLN A 104 -2.590 9.379 19.730 0.00 0.00 A2 H

ATOM 1583 C GLN A 104 2.174 11.412 18.607 1.00 0.00 A2 C

ATOM 1584 O GLN A 104 2.880 10.413 18.643 1.00 0.00 A2 O

ATOM 1585 N GLY A 105 1.902 12.055 17.470 1.00 0.00 A2 N

ATOM 1586 HN GLY A 105 1.283 12.838 17.430 0.00 0.00 A2 H

ATOM 1587 CA GLY A 105 2.493 11.656 16.205 1.00 0.00 A2 C

ATOM 1588 HA1 GLY A 105 3.563 11.769 16.313 0.00 0.00 A2 H

ATOM 1589 HA2 GLY A 105 2.055 12.284 15.441 0.00 0.00 A2 H

ATOM 1590 C GLY A 105 2.206 10.205 15.841 1.00 0.00 A2 C

ATOM 1591 O GLY A 105 1.145 9.669 16.159 1.00 0.00 A2 O

ATOM 1592 N ILE A 106 3.165 9.569 15.177 1.00 0.00 A2 N

ATOM 1593 HN ILE A 106 4.044 9.986 14.957 0.00 0.00 A2 H

ATOM 1594 CA ILE A 106 3.020 8.191 14.704 1.00 0.00 A2 C

ATOM 1595 HA ILE A 106 2.091 8.173 14.149 0.00 0.00 A2 H

ATOM 1596 CB ILE A 106 4.191 7.818 13.753 1.00 0.00 A2 C

ATOM 1597 HB ILE A 106 4.220 8.650 13.005 0.00 0.00 A2 H

ATOM 1598 CG2 ILE A 106 5.515 7.732 14.515 1.00 0.00 A2 C

ATOM 1599 1HG2 ILE A 106 6.365 7.621 13.810 0.00 0.00 A2 H

ATOM 1600 2HG2 ILE A 106 5.690 8.653 15.110 0.00 0.00 A2 H

ATOM 1601 3HG2 ILE A 106 5.521 6.862 15.205 0.00 0.00 A2 H

ATOM 1602 CG1 ILE A 106 3.879 6.536 12.971 1.00 0.00 A2 C

ATOM 1603 1HG1 ILE A 106 4.024 5.649 13.628 0.00 0.00 A2 H

ATOM 1604 2HG1 ILE A 106 2.807 6.550 12.669 0.00 0.00 A2 H

ATOM 1605 CD ILE A 106 4.722 6.373 11.723 1.00 0.00 A2 C

ATOM 1606 HD1 ILE A 106 4.415 5.461 11.167 0.00 0.00 A2 H

ATOM 1607 HD2 ILE A 106 4.599 7.249 11.050 0.00 0.00 A2 H

ATOM 1608 HD3 ILE A 106 5.798 6.273 11.979 0.00 0.00 A2 H

ATOM 1609 C ILE A 106 2.876 7.169 15.844 1.00 0.00 A2 C

ATOM 1610 O ILE A 106 2.533 6.005 15.614 1.00 0.00 A2 O

ATOM 1611 N ARG A 107 3.121 7.603 17.081 1.00 0.00 A2 N

ATOM 1612 HN ARG A 107 3.420 8.535 17.274 0.00 0.00 A2 H

ATOM 1613 CA ARG A 107 2.959 6.712 18.234 1.00 0.00 A2 C

ATOM 1614 HA ARG A 107 3.438 5.768 18.003 0.00 0.00 A2 H

ATOM 1615 CB ARG A 107 3.599 7.313 19.491 1.00 0.00 A2 C

ATOM 1616 HB1 ARG A 107 3.290 8.385 19.520 0.00 0.00 A2 H

ATOM 1617 HB2 ARG A 107 3.156 6.842 20.396 0.00 0.00 A2 H

ATOM 1618 CG ARG A 107 5.109 7.226 19.484 1.00 0.00 A2 C

ATOM 1619 HG1 ARG A 107 5.403 6.168 19.662 0.00 0.00 A2 H

ATOM 1620 HG2 ARG A 107 5.481 7.472 18.463 0.00 0.00 A2 H

ATOM 1621 CD ARG A 107 5.728 8.192 20.481 1.00 0.00 A2 C

ATOM 1622 HD1 ARG A 107 6.831 8.229 20.325 0.00 0.00 A2 H

ATOM 1623 HD2 ARG A 107 5.316 9.221 20.363 0.00 0.00 A2 H

ATOM 1624 NE ARG A 107 5.492 7.846 21.885 1.00 0.00 A2 N

ATOM 1625 HE ARG A 107 4.581 8.121 22.212 0.00 0.00 A2 H

ATOM 1626 CZ ARG A 107 6.374 7.230 22.673 1.00 0.00 A2 C

ATOM 1627 NH1 ARG A 107 7.634 6.821 22.172 1.00 0.00 A2 N

ATOM 1628 1HH1 ARG A 107 8.276 6.341 22.753 0.00 0.00 A2 H

ATOM 1629 2HH1 ARG A 107 7.773 6.921 21.184 0.00 0.00 A2 H

ATOM 1630 NH2 ARG A 107 6.048 6.970 24.026 1.00 0.00 A2 N

ATOM 1631 1HH2 ARG A 107 6.731 6.592 24.635 0.00 0.00 A2 H

ATOM 1632 2HH2 ARG A 107 5.209 7.370 24.370 0.00 0.00 A2 H

ATOM 1633 C ARG A 107 1.488 6.401 18.499 1.00 0.00 A2 C

ATOM 1634 O ARG A 107 1.166 5.596 19.366 1.00 0.00 A2 O

ATOM 1635 N ALA A 108 0.598 7.048 17.755 1.00 0.00 A2 N

ATOM 1636 HN ALA A 108 0.843 7.760 17.098 0.00 0.00 A2 H

ATOM 1637 CA ALA A 108 -0.825 6.751 17.854 1.00 0.00 A2 C

ATOM 1638 HA ALA A 108 -1.178 6.909 18.867 0.00 0.00 A2 H

ATOM 1639 CB ALA A 108 -1.630 7.664 16.925 1.00 0.00 A2 C

ATOM 1640 HB1 ALA A 108 -1.457 8.725 17.204 0.00 0.00 A2 H

ATOM 1641 HB2 ALA A 108 -1.316 7.533 15.866 0.00 0.00 A2 H

ATOM 1642 HB3 ALA A 108 -2.721 7.463 16.998 0.00 0.00 A2 H

ATOM 1643 C ALA A 108 -1.108 5.286 17.523 1.00 0.00 A2 C

ATOM 1644 O ALA A 108 -2.085 4.716 18.005 1.00 0.00 A2 O

ATOM 1645 N TRP A 109 -0.257 4.685 16.693 1.00 0.00 A2 N

ATOM 1646 HN TRP A 109 0.533 5.144 16.292 0.00 0.00 A2 H

ATOM 1647 CA TRP A 109 -0.423 3.285 16.303 1.00 0.00 A2 C

ATOM 1648 HA TRP A 109 -1.470 3.036 16.429 0.00 0.00 A2 H

ATOM 1649 CB TRP A 109 0.033 3.066 14.864 1.00 0.00 A2 C

ATOM 1650 HB1 TRP A 109 0.995 3.607 14.725 0.00 0.00 A2 H

ATOM 1651 HB2 TRP A 109 0.243 1.987 14.694 0.00 0.00 A2 H

ATOM 1652 CG TRP A 109 -0.923 3.583 13.819 1.00 0.00 A2 C

ATOM 1653 CD1 TRP A 109 -2.042 2.951 13.344 1.00 0.00 A2 C

ATOM 1654 HD1 TRP A 109 -2.409 1.999 13.704 0.00 0.00 A2 H

ATOM 1655 NE1 TRP A 109 -2.647 3.728 12.375 1.00 0.00 A2 N

ATOM 1656 HE1 TRP A 109 -3.478 3.523 11.905 0.00 0.00 A2 H

ATOM 1657 CE2 TRP A 109 -1.918 4.876 12.205 1.00 0.00 A2 C

ATOM 1658 CD2 TRP A 109 -0.829 4.824 13.104 1.00 0.00 A2 C

ATOM 1659 CE3 TRP A 109 0.079 5.894 13.128 1.00 0.00 A2 C

ATOM 1660 HE3 TRP A 109 0.939 5.898 13.784 0.00 0.00 A2 H

ATOM 1661 CZ3 TRP A 109 -0.138 6.975 12.272 1.00 0.00 A2 C

ATOM 1662 HZ3 TRP A 109 0.552 7.807 12.281 0.00 0.00 A2 H

ATOM 1663 CZ2 TRP A 109 -2.136 5.960 11.357 1.00 0.00 A2 C

ATOM 1664 HZ2 TRP A 109 -2.979 6.005 10.685 0.00 0.00 A2 H

ATOM 1665 CH2 TRP A 109 -1.233 6.995 11.400 1.00 0.00 A2 C

ATOM 1666 HH2 TRP A 109 -1.376 7.841 10.742 0.00 0.00 A2 H

ATOM 1667 C TRP A 109 0.360 2.364 17.209 1.00 0.00 A2 C

ATOM 1668 O TRP A 109 1.591 2.380 17.191 1.00 0.00 A2 O

ATOM 1669 N VAL A 110 -0.346 1.562 18.002 1.00 0.00 A2 N

ATOM 1670 HN VAL A 110 -1.341 1.523 18.030 0.00 0.00 A2 H

ATOM 1671 CA VAL A 110 0.320 0.646 18.924 1.00 0.00 A2 C

ATOM 1672 HA VAL A 110 0.974 1.243 19.548 0.00 0.00 A2 H

ATOM 1673 CB VAL A 110 -0.691 -0.076 19.835 1.00 0.00 A2 C

ATOM 1674 HB VAL A 110 -1.208 0.702 20.449 0.00 0.00 A2 H

ATOM 1675 CG1 VAL A 110 -1.711 -0.815 19.000 1.00 0.00 A2 C

ATOM 1676 1HG1 VAL A 110 -2.418 -1.368 19.655 0.00 0.00 A2 H

ATOM 1677 2HG1 VAL A 110 -2.312 -0.120 18.378 0.00 0.00 A2 H

ATOM 1678 3HG1 VAL A 110 -1.213 -1.551 18.333 0.00 0.00 A2 H

ATOM 1679 CG2 VAL A 110 0.030 -1.043 20.766 1.00 0.00 A2 C

ATOM 1680 1HG2 VAL A 110 -0.697 -1.596 21.398 0.00 0.00 A2 H

ATOM 1681 2HG2 VAL A 110 0.618 -1.783 20.181 0.00 0.00 A2 H

ATOM 1682 3HG2 VAL A 110 0.723 -0.505 21.445 0.00 0.00 A2 H

ATOM 1683 C VAL A 110 1.196 -0.376 18.184 1.00 0.00 A2 C

ATOM 1684 O VAL A 110 2.277 -0.743 18.652 1.00 0.00 A2 O

ATOM 1685 N ALA A 111 0.742 -0.825 17.018 1.00 0.00 A2 N

ATOM 1686 HN ALA A 111 -0.137 -0.566 16.619 0.00 0.00 A2 H

ATOM 1687 CA ALA A 111 1.536 -1.756 16.230 1.00 0.00 A2 C

ATOM 1688 HA ALA A 111 1.760 -2.644 16.809 0.00 0.00 A2 H

ATOM 1689 CB ALA A 111 0.773 -2.210 15.000 1.00 0.00 A2 C

ATOM 1690 HB1 ALA A 111 -0.179 -2.691 15.309 0.00 0.00 A2 H

ATOM 1691 HB2 ALA A 111 0.523 -1.348 14.344 0.00 0.00 A2 H

ATOM 1692 HB3 ALA A 111 1.354 -2.949 14.407 0.00 0.00 A2 H

ATOM 1693 C ALA A 111 2.882 -1.152 15.829 1.00 0.00 A2 C

ATOM 1694 O ALA A 111 3.885 -1.862 15.757 1.00 0.00 A2 O

ATOM 1695 N TRP A 112 2.904 0.150 15.558 1.00 0.00 A2 N

ATOM 1696 HN TRP A 112 2.109 0.751 15.599 0.00 0.00 A2 H

ATOM 1697 CA TRP A 112 4.150 0.801 15.167 1.00 0.00 A2 C

ATOM 1698 HA TRP A 112 4.649 0.167 14.444 0.00 0.00 A2 H

ATOM 1699 CB TRP A 112 3.899 2.181 14.540 1.00 0.00 A2 C

ATOM 1700 HB1 TRP A 112 3.282 2.033 13.626 0.00 0.00 A2 H

ATOM 1701 HB2 TRP A 112 3.300 2.809 15.235 0.00 0.00 A2 H

ATOM 1702 CG TRP A 112 5.172 2.887 14.130 1.00 0.00 A2 C

ATOM 1703 CD1 TRP A 112 5.793 2.817 12.914 1.00 0.00 A2 C

ATOM 1704 HD1 TRP A 112 5.415 2.263 12.065 0.00 0.00 A2 H

ATOM 1705 NE1 TRP A 112 6.933 3.582 12.922 1.00 0.00 A2 N

ATOM 1706 HE1 TRP A 112 7.541 3.720 12.170 0.00 0.00 A2 H

ATOM 1707 CE2 TRP A 112 7.065 4.174 14.150 1.00 0.00 A2 C

ATOM 1708 CD2 TRP A 112 5.975 3.756 14.939 1.00 0.00 A2 C

ATOM 1709 CE3 TRP A 112 5.885 4.215 16.259 1.00 0.00 A2 C

ATOM 1710 HE3 TRP A 112 5.074 3.920 16.911 0.00 0.00 A2 H

ATOM 1711 CZ3 TRP A 112 6.865 5.059 16.734 1.00 0.00 A2 C

ATOM 1712 HZ3 TRP A 112 6.808 5.420 17.751 0.00 0.00 A2 H

ATOM 1713 CZ2 TRP A 112 8.053 5.025 14.631 1.00 0.00 A2 C

ATOM 1714 HZ2 TRP A 112 8.885 5.347 14.024 0.00 0.00 A2 H

ATOM 1715 CH2 TRP A 112 7.940 5.457 15.923 1.00 0.00 A2 C

ATOM 1716 HH2 TRP A 112 8.696 6.117 16.325 0.00 0.00 A2 H

ATOM 1717 C TRP A 112 5.076 0.910 16.376 1.00 0.00 A2 C

ATOM 1718 O TRP A 112 6.281 0.680 16.265 1.00 0.00 A2 O

ATOM 1719 N ARG A 113 4.511 1.255 17.529 1.00 0.00 A2 N

ATOM 1720 HN ARG A 113 3.551 1.519 17.594 0.00 0.00 A2 H

ATOM 1721 CA ARG A 113 5.280 1.264 18.772 1.00 0.00 A2 C

ATOM 1722 HA ARG A 113 6.080 1.987 18.669 0.00 0.00 A2 H

ATOM 1723 CB ARG A 113 4.396 1.669 19.953 1.00 0.00 A2 C

ATOM 1724 HB1 ARG A 113 3.581 0.909 20.012 0.00 0.00 A2 H

ATOM 1725 HB2 ARG A 113 4.972 1.573 20.900 0.00 0.00 A2 H

ATOM 1726 CG ARG A 113 3.786 3.056 19.788 1.00 0.00 A2 C

ATOM 1727 HG1 ARG A 113 4.479 3.677 19.179 0.00 0.00 A2 H

ATOM 1728 HG2 ARG A 113 2.852 2.972 19.186 0.00 0.00 A2 H

ATOM 1729 CD ARG A 113 3.449 3.688 21.118 1.00 0.00 A2 C

ATOM 1730 HD1 ARG A 113 4.162 3.324 21.893 0.00 0.00 A2 H

ATOM 1731 HD2 ARG A 113 3.511 4.800 21.070 0.00 0.00 A2 H

ATOM 1732 NE ARG A 113 2.094 3.388 21.571 1.00 0.00 A2 N

ATOM 1733 HE ARG A 113 1.398 3.990 21.163 0.00 0.00 A2 H

ATOM 1734 CZ ARG A 113 1.793 2.424 22.431 1.00 0.00 A2 C

ATOM 1735 NH1 ARG A 113 2.821 1.603 22.958 1.00 0.00 A2 N

ATOM 1736 1HH1 ARG A 113 2.606 0.864 23.581 0.00 0.00 A2 H

ATOM 1737 2HH1 ARG A 113 3.734 1.726 22.563 0.00 0.00 A2 H

ATOM 1738 NH2 ARG A 113 0.451 2.218 22.830 1.00 0.00 A2 N

ATOM 1739 1HH2 ARG A 113 0.242 1.556 23.536 0.00 0.00 A2 H

ATOM 1740 2HH2 ARG A 113 -0.213 2.893 22.536 0.00 0.00 A2 H

ATOM 1741 C ARG A 113 5.944 -0.088 19.035 1.00 0.00 A2 C

ATOM 1742 O ARG A 113 7.129 -0.154 19.392 1.00 0.00 A2 O

ATOM 1743 N ASN A 114 5.190 -1.164 18.828 1.00 0.00 A2 N

ATOM 1744 HN ASN A 114 4.260 -1.128 18.464 0.00 0.00 A2 H

ATOM 1745 CA ASN A 114 5.675 -2.515 19.124 1.00 0.00 A2 C

ATOM 1746 HA ASN A 114 6.215 -2.394 20.057 0.00 0.00 A2 H

ATOM 1747 CB ASN A 114 4.490 -3.472 19.331 1.00 0.00 A2 C

ATOM 1748 HB1 ASN A 114 3.794 -3.342 18.474 0.00 0.00 A2 H

ATOM 1749 HB2 ASN A 114 4.838 -4.525 19.320 0.00 0.00 A2 H

ATOM 1750 CG ASN A 114 3.733 -3.180 20.602 1.00 0.00 A2 C

ATOM 1751 OD1 ASN A 114 4.270 -2.571 21.525 1.00 0.00 A2 O

ATOM 1752 ND2 ASN A 114 2.482 -3.616 20.661 1.00 0.00 A2 N

ATOM 1753 1HD2 ASN A 114 1.967 -3.419 21.491 0.00 0.00 A2 H

ATOM 1754 2HD2 ASN A 114 2.083 -4.092 19.883 0.00 0.00 A2 H

ATOM 1755 C ASN A 114 6.642 -3.122 18.105 1.00 0.00 A2 C

ATOM 1756 O ASN A 114 7.538 -3.886 18.475 1.00 0.00 A2 O

ATOM 1757 N HSD A 115 6.454 -2.811 16.826 1.00 0.00 A2 N

ATOM 1758 HN HSD A 115 5.802 -2.124 16.508 0.00 0.00 A2 H

ATOM 1759 CA HSD A 115 7.218 -3.480 15.773 1.00 0.00 A2 C

ATOM 1760 HA HSD A 115 7.813 -4.230 16.279 0.00 0.00 A2 H

ATOM 1761 CB HSD A 115 6.258 -4.177 14.798 1.00 0.00 A2 C

ATOM 1762 HB1 HSD A 115 5.620 -3.410 14.308 0.00 0.00 A2 H

ATOM 1763 HB2 HSD A 115 6.842 -4.672 13.991 0.00 0.00 A2 H

ATOM 1764 ND1 HSD A 115 5.851 -6.370 15.985 1.00 0.00 A2 N

ATOM 1765 HD1 HSD A 115 6.812 -6.640 16.054 0.00 0.00 A2 H

ATOM 1766 CG HSD A 115 5.370 -5.188 15.455 1.00 0.00 A2 C

ATOM 1767 CE1 HSD A 115 4.847 -7.057 16.501 1.00 0.00 A2 C

ATOM 1768 HE1 HSD A 115 4.960 -8.029 16.989 0.00 0.00 A2 H

ATOM 1769 NE2 HSD A 115 3.734 -6.366 16.326 1.00 0.00 A2 N

ATOM 1770 CD2 HSD A 115 4.033 -5.195 15.670 1.00 0.00 A2 C

ATOM 1771 HD2 HSD A 115 3.282 -4.471 15.377 0.00 0.00 A2 H

ATOM 1772 C HSD A 115 8.177 -2.593 14.985 1.00 0.00 A2 C

ATOM 1773 O HSD A 115 8.959 -3.095 14.180 1.00 0.00 A2 O

ATOM 1774 SG CYS A 116 6.868 -0.565 12.375 1.00 0.00 A2 S

ATOM 1775 CB CYS A 116 8.005 0.434 13.394 1.00 0.00 A2 C

ATOM 1776 N CYS A 116 8.103 -1.281 15.177 1.00 0.00 A2 N

ATOM 1777 HN CYS A 116 7.529 -0.835 15.861 0.00 0.00 A2 H

ATOM 1778 CA CYS A 116 8.900 -0.367 14.353 1.00 0.00 A2 C

ATOM 1779 HA CYS A 116 9.570 -1.003 13.787 0.00 0.00 A2 H

ATOM 1780 HB1 CYS A 116 7.388 1.122 14.015 0.00 0.00 A2 H

ATOM 1781 HB2 CYS A 116 8.633 1.085 12.747 0.00 0.00 A2 H

ATOM 1782 C CYS A 116 9.758 0.609 15.165 1.00 0.00 A2 C

ATOM 1783 O CYS A 116 10.937 0.815 14.860 1.00 0.00 A2 O

ATOM 1784 N GLN A 117 9.156 1.212 16.183 1.00 0.00 A2 N

ATOM 1785 HN GLN A 117 8.227 1.004 16.484 0.00 0.00 A2 H

ATOM 1786 CA GLN A 117 9.824 2.252 16.960 1.00 0.00 A2 C

ATOM 1787 HA GLN A 117 10.017 3.078 16.286 0.00 0.00 A2 H

ATOM 1788 CB GLN A 117 8.923 2.707 18.118 1.00 0.00 A2 C

ATOM 1789 HB1 GLN A 117 7.963 3.046 17.665 0.00 0.00 A2 H

ATOM 1790 HB2 GLN A 117 8.672 1.830 18.757 0.00 0.00 A2 H

ATOM 1791 CG GLN A 117 9.503 3.839 18.968 1.00 0.00 A2 C

ATOM 1792 HG1 GLN A 117 10.402 3.488 19.520 0.00 0.00 A2 H

ATOM 1793 HG2 GLN A 117 9.808 4.693 18.329 0.00 0.00 A2 H

ATOM 1794 CD GLN A 117 8.526 4.343 20.020 1.00 0.00 A2 C

ATOM 1795 OE1 GLN A 117 8.180 5.515 20.041 1.00 0.00 A2 O

ATOM 1796 NE2 GLN A 117 8.078 3.455 20.891 1.00 0.00 A2 N

ATOM 1797 1HE2 GLN A 117 7.435 3.783 21.577 0.00 0.00 A2 H

ATOM 1798 2HE2 GLN A 117 8.399 2.515 20.848 0.00 0.00 A2 H

ATOM 1799 C GLN A 117 11.171 1.762 17.485 1.00 0.00 A2 C

ATOM 1800 O GLN A 117 11.278 0.626 17.946 1.00 0.00 A2 O

ATOM 1801 N ASN A 118 12.193 2.620 17.398 1.00 0.00 A2 N

ATOM 1802 HN ASN A 118 12.081 3.539 17.022 0.00 0.00 A2 H

ATOM 1803 CA ASN A 118 13.566 2.302 17.841 1.00 0.00 A2 C

ATOM 1804 HA ASN A 118 14.116 3.214 17.632 0.00 0.00 A2 H

ATOM 1805 CB ASN A 118 13.624 2.050 19.358 1.00 0.00 A2 C

ATOM 1806 HB1 ASN A 118 12.971 1.178 19.583 0.00 0.00 A2 H

ATOM 1807 HB2 ASN A 118 14.655 1.782 19.665 0.00 0.00 A2 H

ATOM 1808 CG ASN A 118 13.125 3.233 20.178 1.00 0.00 A2 C

ATOM 1809 OD1 ASN A 118 12.658 3.062 21.306 1.00 0.00 A2 O

ATOM 1810 ND2 ASN A 118 13.232 4.433 19.628 1.00 0.00 A2 N

ATOM 1811 1HD2 ASN A 118 12.918 5.212 20.164 0.00 0.00 A2 H

ATOM 1812 2HD2 ASN A 118 13.641 4.533 18.726 0.00 0.00 A2 H

ATOM 1813 C ASN A 118 14.276 1.150 17.106 1.00 0.00 A2 C

ATOM 1814 O ASN A 118 15.388 0.774 17.476 1.00 0.00 A2 O

ATOM 1815 N ARG A 119 13.656 0.589 16.071 1.00 0.00 A2 N

ATOM 1816 HN ARG A 119 12.778 0.922 15.736 0.00 0.00 A2 H

ATOM 1817 CA ARG A 119 14.236 -0.551 15.369 1.00 0.00 A2 C

ATOM 1818 HA ARG A 119 15.083 -0.898 15.949 0.00 0.00 A2 H

ATOM 1819 CB ARG A 119 13.207 -1.681 15.211 1.00 0.00 A2 C

ATOM 1820 HB1 ARG A 119 12.334 -1.239 14.674 0.00 0.00 A2 H

ATOM 1821 HB2 ARG A 119 13.616 -2.466 14.537 0.00 0.00 A2 H

ATOM 1822 CG ARG A 119 12.723 -2.275 16.520 1.00 0.00 A2 C

ATOM 1823 HG1 ARG A 119 13.573 -2.803 17.005 0.00 0.00 A2 H

ATOM 1824 HG2 ARG A 119 12.450 -1.447 17.214 0.00 0.00 A2 H

ATOM 1825 CD ARG A 119 11.521 -3.152 16.285 1.00 0.00 A2 C

ATOM 1826 HD1 ARG A 119 10.756 -2.585 15.706 0.00 0.00 A2 H

ATOM 1827 HD2 ARG A 119 11.791 -4.072 15.715 0.00 0.00 A2 H

ATOM 1828 NE ARG A 119 10.917 -3.609 17.533 1.00 0.00 A2 N

ATOM 1829 HE ARG A 119 10.214 -2.981 17.885 0.00 0.00 A2 H

ATOM 1830 CZ ARG A 119 11.245 -4.733 18.157 1.00 0.00 A2 C

ATOM 1831 NH1 ARG A 119 12.244 -5.584 17.625 1.00 0.00 A2 N

ATOM 1832 1HH1 ARG A 119 12.473 -6.436 18.075 0.00 0.00 A2 H

ATOM 1833 2HH1 ARG A 119 12.594 -5.344 16.717 0.00 0.00 A2 H

ATOM 1834 NH2 ARG A 119 10.588 -5.080 19.362 1.00 0.00 A2 N

ATOM 1835 1HH2 ARG A 119 10.887 -5.870 19.877 0.00 0.00 A2 H

ATOM 1836 2HH2 ARG A 119 9.986 -4.398 19.757 0.00 0.00 A2 H

ATOM 1837 C ARG A 119 14.771 -0.184 13.995 1.00 0.00 A2 C

ATOM 1838 O ARG A 119 14.413 0.843 13.429 1.00 0.00 A2 O

ATOM 1839 N ASP A 120 15.616 -1.053 13.449 1.00 0.00 A2 N

ATOM 1840 HN ASP A 120 15.977 -1.871 13.889 0.00 0.00 A2 H

ATOM 1841 CA ASP A 120 16.111 -0.873 12.091 1.00 0.00 A2 C

ATOM 1842 HA ASP A 120 16.394 0.167 11.977 0.00 0.00 A2 H

ATOM 1843 CB ASP A 120 17.275 -1.827 11.829 1.00 0.00 A2 C

ATOM 1844 HB1 ASP A 120 18.006 -1.747 12.659 0.00 0.00 A2 H

ATOM 1845 HB2 ASP A 120 16.918 -2.875 11.779 0.00 0.00 A2 H

ATOM 1846 CG ASP A 120 18.015 -1.509 10.541 1.00 0.00 A2 C

ATOM 1847 OD1 ASP A 120 17.305 -1.287 9.407 1.00 0.00 A2 O

ATOM 1848 OD2 ASP A 120 19.368 -1.433 10.575 1.00 0.00 A2 O

ATOM 1849 C ASP A 120 14.972 -1.135 11.093 1.00 0.00 A2 C

ATOM 1850 O ASP A 120 14.357 -2.197 11.114 1.00 0.00 A2 O

ATOM 1851 N VAL A 121 14.690 -0.167 10.222 1.00 0.00 A2 N

ATOM 1852 HN VAL A 121 15.163 0.709 10.180 0.00 0.00 A2 H

ATOM 1853 CA VAL A 121 13.629 -0.328 9.231 1.00 0.00 A2 C

ATOM 1854 HA VAL A 121 13.309 -1.362 9.266 0.00 0.00 A2 H

ATOM 1855 CB VAL A 121 12.409 0.584 9.544 1.00 0.00 A2 C

ATOM 1856 HB VAL A 121 11.715 0.531 8.669 0.00 0.00 A2 H

ATOM 1857 CG1 VAL A 121 11.682 0.094 10.794 1.00 0.00 A2 C

ATOM 1858 1HG1 VAL A 121 10.834 0.770 11.037 0.00 0.00 A2 H

ATOM 1859 2HG1 VAL A 121 11.263 -0.923 10.652 0.00 0.00 A2 H

ATOM 1860 3HG1 VAL A 121 12.369 0.077 11.667 0.00 0.00 A2 H

ATOM 1861 CG2 VAL A 121 12.844 2.041 9.706 1.00 0.00 A2 C

ATOM 1862 1HG2 VAL A 121 11.976 2.683 9.969 0.00 0.00 A2 H

ATOM 1863 2HG2 VAL A 121 13.605 2.134 10.511 0.00 0.00 A2 H

ATOM 1864 3HG2 VAL A 121 13.281 2.437 8.766 0.00 0.00 A2 H

ATOM 1865 C VAL A 121 14.147 -0.054 7.812 1.00 0.00 A2 C

ATOM 1866 O VAL A 121 13.368 0.083 6.866 1.00 0.00 A2 O

ATOM 1867 N ARG A 122 15.468 0.023 7.676 1.00 0.00 A2 N

ATOM 1868 HN ARG A 122 16.080 -0.141 8.446 0.00 0.00 A2 H

ATOM 1869 CA ARG A 122 16.095 0.350 6.398 1.00 0.00 A2 C

ATOM 1870 HA ARG A 122 15.737 1.326 6.093 0.00 0.00 A2 H

ATOM 1871 CB ARG A 122 17.625 0.385 6.530 1.00 0.00 A2 C

ATOM 1872 HB1 ARG A 122 17.925 -0.590 6.984 0.00 0.00 A2 H

ATOM 1873 HB2 ARG A 122 18.086 0.405 5.518 0.00 0.00 A2 H

ATOM 1874 CG ARG A 122 18.182 1.512 7.403 1.00 0.00 A2 C

ATOM 1875 HG1 ARG A 122 17.863 2.485 6.970 0.00 0.00 A2 H

ATOM 1876 HG2 ARG A 122 17.705 1.461 8.409 0.00 0.00 A2 H

ATOM 1877 CD ARG A 122 19.712 1.389 7.563 1.00 0.00 A2 C

ATOM 1878 HD1 ARG A 122 20.179 1.251 6.561 0.00 0.00 A2 H

ATOM 1879 HD2 ARG A 122 20.148 2.299 8.038 0.00 0.00 A2 H

ATOM 1880 NE ARG A 122 20.091 0.263 8.414 1.00 0.00 A2 N

ATOM 1881 HE ARG A 122 19.300 -0.212 8.816 0.00 0.00 A2 H

ATOM 1882 CZ ARG A 122 21.344 -0.126 8.657 1.00 0.00 A2 C

ATOM 1883 NH1 ARG A 122 22.444 0.560 8.087 1.00 0.00 A2 N

ATOM 1884 1HH1 ARG A 122 23.371 0.251 8.251 0.00 0.00 A2 H

ATOM 1885 2HH1 ARG A 122 22.224 1.271 7.415 0.00 0.00 A2 H

ATOM 1886 NH2 ARG A 122 21.582 -1.239 9.498 1.00 0.00 A2 N

ATOM 1887 1HH2 ARG A 122 22.509 -1.478 9.751 0.00 0.00 A2 H

ATOM 1888 2HH2 ARG A 122 20.799 -1.616 9.975 0.00 0.00 A2 H

ATOM 1889 C ARG A 122 15.701 -0.624 5.293 1.00 0.00 A2 C

ATOM 1890 O ARG A 122 15.635 -0.237 4.121 1.00 0.00 A2 O

ATOM 1891 N GLN A 123 15.432 -1.881 5.655 1.00 0.00 A2 N

ATOM 1892 HN GLN A 123 15.430 -2.216 6.596 0.00 0.00 A2 H

ATOM 1893 CA GLN A 123 15.106 -2.899 4.646 1.00 0.00 A2 C

ATOM 1894 HA GLN A 123 15.908 -2.858 3.919 0.00 0.00 A2 H

ATOM 1895 CB GLN A 123 15.109 -4.305 5.259 1.00 0.00 A2 C

ATOM 1896 HB1 GLN A 123 15.002 -5.025 4.415 0.00 0.00 A2 H

ATOM 1897 HB2 GLN A 123 16.105 -4.507 5.714 0.00 0.00 A2 H

ATOM 1898 CG GLN A 123 13.994 -4.551 6.275 1.00 0.00 A2 C

ATOM 1899 HG1 GLN A 123 13.147 -3.854 6.097 0.00 0.00 A2 H

ATOM 1900 HG2 GLN A 123 13.606 -5.587 6.189 0.00 0.00 A2 H

ATOM 1901 CD GLN A 123 14.427 -4.322 7.730 1.00 0.00 A2 C

ATOM 1902 OE1 GLN A 123 15.222 -3.414 8.048 1.00 0.00 A2 O

ATOM 1903 NE2 GLN A 123 13.892 -5.151 8.624 1.00 0.00 A2 N

ATOM 1904 1HE2 GLN A 123 14.156 -5.018 9.575 0.00 0.00 A2 H

ATOM 1905 2HE2 GLN A 123 13.275 -5.870 8.324 0.00 0.00 A2 H

ATOM 1906 C GLN A 123 13.785 -2.639 3.896 1.00 0.00 A2 C

ATOM 1907 O GLN A 123 13.587 -3.123 2.776 1.00 0.00 A2 O

ATOM 1908 N TYR A 124 12.887 -1.864 4.493 1.00 0.00 A2 N

ATOM 1909 HN TYR A 124 12.984 -1.476 5.408 0.00 0.00 A2 H

ATOM 1910 CA TYR A 124 11.637 -1.514 3.812 1.00 0.00 A2 C

ATOM 1911 HA TYR A 124 11.196 -2.462 3.528 0.00 0.00 A2 H

ATOM 1912 CB TYR A 124 10.664 -0.807 4.763 1.00 0.00 A2 C

ATOM 1913 HB1 TYR A 124 11.215 -0.030 5.337 0.00 0.00 A2 H

ATOM 1914 HB2 TYR A 124 9.864 -0.288 4.192 0.00 0.00 A2 H

ATOM 1915 CG TYR A 124 10.070 -1.773 5.747 1.00 0.00 A2 C

ATOM 1916 CD1 TYR A 124 8.961 -2.534 5.408 1.00 0.00 A2 C

ATOM 1917 HD1 TYR A 124 8.495 -2.403 4.441 0.00 0.00 A2 H

ATOM 1918 CE1 TYR A 124 8.426 -3.442 6.290 1.00 0.00 A2 C

ATOM 1919 HE1 TYR A 124 7.556 -4.023 6.021 0.00 0.00 A2 H

ATOM 1920 CZ TYR A 124 9.006 -3.600 7.527 1.00 0.00 A2 C

ATOM 1921 OH TYR A 124 8.470 -4.497 8.411 1.00 0.00 A2 O

ATOM 1922 HH TYR A 124 9.043 -4.521 9.180 0.00 0.00 A2 H

ATOM 1923 CD2 TYR A 124 10.642 -1.961 7.001 1.00 0.00 A2 C

ATOM 1924 HD2 TYR A 124 11.493 -1.365 7.302 0.00 0.00 A2 H

ATOM 1925 CE2 TYR A 124 10.114 -2.859 7.888 1.00 0.00 A2 C

ATOM 1926 HE2 TYR A 124 10.554 -2.966 8.868 0.00 0.00 A2 H

ATOM 1927 C TYR A 124 11.820 -0.703 2.535 1.00 0.00 A2 C

ATOM 1928 O TYR A 124 11.005 -0.799 1.614 1.00 0.00 A2 O

ATOM 1929 N VAL A 125 12.884 0.087 2.468 1.00 0.00 A2 N

ATOM 1930 HN VAL A 125 13.585 0.157 3.173 0.00 0.00 A2 H

ATOM 1931 CA VAL A 125 13.108 0.945 1.305 1.00 0.00 A2 C

ATOM 1932 HA VAL A 125 12.235 0.856 0.671 0.00 0.00 A2 H

ATOM 1933 CB VAL A 125 13.252 2.444 1.714 1.00 0.00 A2 C

ATOM 1934 HB VAL A 125 13.447 3.031 0.783 0.00 0.00 A2 H

ATOM 1935 CG1 VAL A 125 11.959 2.956 2.370 1.00 0.00 A2 C

ATOM 1936 1HG1 VAL A 125 12.083 4.012 2.694 0.00 0.00 A2 H

ATOM 1937 2HG1 VAL A 125 11.102 2.924 1.666 0.00 0.00 A2 H

ATOM 1938 3HG1 VAL A 125 11.706 2.350 3.266 0.00 0.00 A2 H

ATOM 1939 CG2 VAL A 125 14.439 2.642 2.649 1.00 0.00 A2 C

ATOM 1940 1HG2 VAL A 125 14.514 3.703 2.969 0.00 0.00 A2 H

ATOM 1941 2HG2 VAL A 125 14.331 2.009 3.556 0.00 0.00 A2 H

ATOM 1942 3HG2 VAL A 125 15.393 2.374 2.149 0.00 0.00 A2 H

ATOM 1943 C VAL A 125 14.321 0.488 0.484 1.00 0.00 A2 C

ATOM 1944 O VAL A 125 14.649 1.074 -0.546 1.00 0.00 A2 O

ATOM 1945 N GLN A 126 14.983 -0.563 0.947 1.00 0.00 A2 N

ATOM 1946 HN GLN A 126 14.741 -1.071 1.772 0.00 0.00 A2 H

ATOM 1947 CA GLN A 126 16.157 -1.067 0.253 1.00 0.00 A2 C

ATOM 1948 HA GLN A 126 16.872 -0.254 0.219 0.00 0.00 A2 H

ATOM 1949 CB GLN A 126 16.741 -2.269 0.995 1.00 0.00 A2 C

ATOM 1950 HB1 GLN A 126 17.295 -1.867 1.875 0.00 0.00 A2 H

ATOM 1951 HB2 GLN A 126 15.911 -2.887 1.407 0.00 0.00 A2 H

ATOM 1952 CG GLN A 126 17.679 -3.111 0.141 1.00 0.00 A2 C

ATOM 1953 HG1 GLN A 126 17.154 -3.473 -0.769 0.00 0.00 A2 H

ATOM 1954 HG2 GLN A 126 18.555 -2.514 -0.186 0.00 0.00 A2 H

ATOM 1955 CD GLN A 126 18.180 -4.349 0.862 1.00 0.00 A2 C

ATOM 1956 OE1 GLN A 126 17.925 -4.539 2.056 1.00 0.00 A2 O

ATOM 1957 NE2 GLN A 126 18.899 -5.200 0.137 1.00 0.00 A2 N

ATOM 1958 1HE2 GLN A 126 19.229 -6.018 0.599 0.00 0.00 A2 H

ATOM 1959 2HE2 GLN A 126 19.096 -4.994 -0.816 0.00 0.00 A2 H

ATOM 1960 C GLN A 126 15.839 -1.460 -1.187 1.00 0.00 A2 C

ATOM 1961 O GLN A 126 14.946 -2.266 -1.439 1.00 0.00 A2 O

ATOM 1962 N GLY A 127 16.578 -0.882 -2.127 1.00 0.00 A2 N

ATOM 1963 HN GLY A 127 17.241 -0.163 -1.921 0.00 0.00 A2 H

ATOM 1964 CA GLY A 127 16.473 -1.258 -3.526 1.00 0.00 A2 C

ATOM 1965 HA1 GLY A 127 16.464 -2.339 -3.534 0.00 0.00 A2 H

ATOM 1966 HA2 GLY A 127 17.333 -0.817 -4.013 0.00 0.00 A2 H

ATOM 1967 C GLY A 127 15.227 -0.772 -4.246 1.00 0.00 A2 C

ATOM 1968 O GLY A 127 14.886 -1.295 -5.308 1.00 0.00 A2 O

ATOM 1969 SG CYS A 128 11.578 -0.424 -2.538 1.00 0.00 A2 S

ATOM 1970 CB CYS A 128 12.273 1.060 -3.259 1.00 0.00 A2 C

ATOM 1971 N CYS A 128 14.549 0.225 -3.681 1.00 0.00 A2 N

ATOM 1972 HN CYS A 128 14.804 0.654 -2.816 0.00 0.00 A2 H

ATOM 1973 CA CYS A 128 13.352 0.775 -4.306 1.00 0.00 A2 C

ATOM 1974 HA CYS A 128 13.001 0.015 -4.994 0.00 0.00 A2 H

ATOM 1975 HB1 CYS A 128 12.748 1.661 -2.451 0.00 0.00 A2 H

ATOM 1976 HB2 CYS A 128 11.483 1.708 -3.699 0.00 0.00 A2 H

ATOM 1977 C CYS A 128 13.640 2.031 -5.112 1.00 0.00 A2 C

ATOM 1978 O CYS A 128 12.771 2.530 -5.827 1.00 0.00 A2 O

ATOM 1979 N GLY A 129 14.858 2.546 -4.992 1.00 0.00 A2 N

ATOM 1980 HN GLY A 129 15.565 2.148 -4.408 0.00 0.00 A2 H

ATOM 1981 CA GLY A 129 15.245 3.747 -5.719 1.00 0.00 A2 C

ATOM 1982 HA1 GLY A 129 15.028 3.549 -6.760 0.00 0.00 A2 H

ATOM 1983 HA2 GLY A 129 16.294 3.905 -5.506 0.00 0.00 A2 H

ATOM 1984 C GLY A 129 14.490 5.002 -5.312 1.00 0.00 A2 C

ATOM 1985 O GLY A 129 14.249 5.890 -6.135 1.00 0.00 A2 O

ATOM 1986 C VAL A 130 14.275 7.066 -2.610 1.00 0.00 A2 C

ATOM 1987 OT1 VAL A 130 15.179 6.641 -2.560 0.00 0.00 A2 O

ATOM 1988 OT2 VAL A 130 13.752 7.847 -2.270 0.00 0.00 A2 O

ATOM 1989 N VAL A 130 14.120 5.080 -4.038 1.00 0.00 A2 N

ATOM 1990 HN VAL A 130 14.314 4.378 -3.358 0.00 0.00 A2 H

ATOM 1991 CA VAL A 130 13.382 6.230 -3.525 1.00 0.00 A2 C

ATOM 1992 HA VAL A 130 13.109 6.851 -4.369 0.00 0.00 A2 H

ATOM 1993 CB VAL A 130 12.143 5.788 -2.726 1.00 0.00 A2 C

ATOM 1994 HB VAL A 130 11.626 6.712 -2.366 0.00 0.00 A2 H

ATOM 1995 CG1 VAL A 130 11.198 4.997 -3.614 1.00 0.00 A2 C

ATOM 1996 1HG1 VAL A 130 10.322 4.644 -3.028 0.00 0.00 A2 H

ATOM 1997 2HG1 VAL A 130 10.809 5.614 -4.450 0.00 0.00 A2 H

ATOM 1998 3HG1 VAL A 130 11.710 4.106 -4.037 0.00 0.00 A2 H

ATOM 1999 CG2 VAL A 130 12.568 4.960 -1.513 1.00 0.00 A2 C

ATOM 2000 1HG2 VAL A 130 11.679 4.602 -0.951 0.00 0.00 A2 H

ATOM 2001 2HG2 VAL A 130 13.160 4.075 -1.832 0.00 0.00 A2 H

ATOM 2002 3HG2 VAL A 130 13.187 5.560 -0.814 0.00 0.00 A2 H

TER

**coordinates of SNP**

ATOM 1 OX1 PHE P 1 1.619 34.375 -44.284 0.00 0.00 O

ATOM 2 SI1 PHE P 2 3.040 34.040 -43.662 0.00 0.00 S

ATOM 3 OX1 PHE P 3 4.336 34.575 -44.407 0.00 0.00 O

ATOM 4 OX1 PHE P 4 3.143 32.757 -42.728 0.00 0.00 O

ATOM 5 SI1 PHE P 5 4.250 32.529 -41.609 0.00 0.00 S

ATOM 6 OX1 PHE P 6 6.294 30.284 -40.058 0.00 0.00 O

ATOM 7 OX1 PHE P 7 3.981 31.124 -40.906 0.00 0.00 O

ATOM 8 OX1 PHE P 8 9.114 32.186 -40.415 0.00 0.00 O

ATOM 9 SI1 PHE P 9 10.086 32.614 -41.596 0.00 0.00 S

ATOM 10 SI1 PHE P 10 10.875 34.986 -42.869 0.00 0.00 S

ATOM 11 OX1 PHE P 11 10.611 34.113 -41.569 0.00 0.00 O

ATOM 12 OX1 PHE P 12 11.024 31.472 -42.191 0.00 0.00 O

ATOM 13 SI1 PHE P 13 12.118 30.646 -41.374 0.00 0.00 S

ATOM 14 OX1 PHE P 14 14.242 27.683 -43.236 0.00 0.00 O

ATOM 15 OX1 PHE P 15 12.684 29.507 -42.336 0.00 0.00 O

ATOM 16 OX1 PHE P 16 13.321 31.556 -40.859 0.00 0.00 O

ATOM 17 OX1 PHE P 17 13.762 33.396 -42.705 0.00 0.00 O

ATOM 18 OX1 PHE P 18 17.121 30.036 -45.488 0.00 0.00 O

ATOM 19 SI1 PHE P 19 14.408 32.309 -41.748 0.00 0.00 S

ATOM 20 SI1 PHE P 20 18.205 30.082 -44.330 0.00 0.00 S

ATOM 21 SI1 PHE P 21 19.217 32.665 -44.632 0.00 0.00 S

ATOM 22 OX1 PHE P 22 18.544 31.524 -43.755 0.00 0.00 O

ATOM 23 OX1 PHE P 23 19.319 28.945 -44.382 0.00 0.00 O

ATOM 24 SI1 PHE P 24 20.100 28.312 -43.144 0.00 0.00 S

ATOM 25 OX1 PHE P 25 22.343 25.000 -43.959 0.00 0.00 O

ATOM 26 OX1 PHE P 26 20.835 27.012 -43.697 0.00 0.00 O

ATOM 27 OX1 PHE P 27 21.152 29.312 -42.490 0.00 0.00 O

ATOM 28 OX1 PHE P 28 22.015 30.847 -44.440 0.00 0.00 O

ATOM 29 OX1 PHE P 29 26.557 28.238 -45.748 0.00 0.00 O

ATOM 30 SI1 PHE P 30 22.427 29.933 -43.212 0.00 0.00 S

ATOM 31 SI1 PHE P 31 27.760 28.167 -44.716 0.00 0.00 S

ATOM 32 SI1 PHE P 32 29.689 29.958 -43.739 0.00 0.00 S

ATOM 33 OX1 PHE P 33 28.190 29.559 -44.082 0.00 0.00 O

ATOM 34 OX1 PHE P 34 27.809 26.827 -43.853 0.00 0.00 O

ATOM 35 SI1 PHE P 35 28.923 26.328 -42.831 0.00 0.00 S

ATOM 36 OX1 PHE P 36 30.718 23.591 -41.729 0.00 0.00 O

ATOM 37 OX1 PHE P 37 28.510 24.884 -42.306 0.00 0.00 O

ATOM 38 OX1 PHE P 38 30.399 26.391 -43.416 0.00 0.00 O

ATOM 39 OX1 PHE P 39 -12.346 27.872 -41.550 0.00 0.00 O

ATOM 40 SI1 PHE P 40 -11.360 27.719 -42.784 0.00 0.00 S

ATOM 41 OX1 PHE P 41 -11.063 26.271 -43.364 0.00 0.00 O

ATOM 42 OX1 PHE P 42 -10.349 28.901 -43.102 0.00 0.00 O

ATOM 43 SI1 PHE P 43 -9.094 29.272 -42.204 0.00 0.00 S

ATOM 44 SI1 PHE P 44 -8.039 31.428 -43.646 0.00 0.00 S

ATOM 45 OX1 PHE P 45 -8.603 30.780 -42.311 0.00 0.00 O

ATOM 46 OX1 PHE P 46 -8.005 28.140 -41.935 0.00 0.00 O

ATOM 47 SI1 PHE P 47 -7.103 28.057 -40.623 0.00 0.00 S

ATOM 48 OX1 PHE P 48 -3.668 26.705 -40.506 0.00 0.00 O

ATOM 49 OX1 PHE P 49 -6.264 26.694 -40.689 0.00 0.00 O

ATOM 50 OX1 PHE P 50 -6.254 29.401 -40.468 0.00 0.00 O

ATOM 51 OX1 PHE P 51 -5.213 29.621 -42.893 0.00 0.00 O

ATOM 52 SI1 PHE P 52 -3.066 26.510 -41.979 0.00 0.00 S

ATOM 53 OX1 PHE P 53 -2.832 25.012 -42.464 0.00 0.00 O

ATOM 54 OX1 PHE P 54 -1.913 27.549 -42.361 0.00 0.00 O

ATOM 55 SI1 PHE P 55 -5.048 29.952 -41.348 0.00 0.00 S

ATOM 56 OX1 PHE P 56 -3.623 29.568 -40.776 0.00 0.00 O

ATOM 57 SI1 PHE P 57 -0.533 27.738 -41.580 0.00 0.00 S

ATOM 58 SI1 PHE P 58 0.506 30.315 -41.226 0.00 0.00 S

ATOM 59 OX1 PHE P 59 -0.301 29.049 -40.714 0.00 0.00 O

ATOM 60 OX1 PHE P 60 0.667 26.705 -41.756 0.00 0.00 O

ATOM 61 OX1 PHE P 61 0.763 25.370 -39.470 0.00 0.00 O

ATOM 62 OX1 PHE P 62 -1.091 27.096 -38.580 0.00 0.00 O

ATOM 63 OX1 PHE P 63 0.708 31.482 -40.169 0.00 0.00 O

ATOM 64 SI1 PHE P 64 1.596 26.114 -40.598 0.00 0.00 S

ATOM 65 OX1 PHE P 65 4.843 23.917 -41.190 0.00 0.00 O

ATOM 66 OX1 PHE P 66 2.516 25.005 -41.293 0.00 0.00 O

ATOM 67 OX1 PHE P 67 2.449 27.247 -39.869 0.00 0.00 O

ATOM 68 OX1 PHE P 68 4.007 28.173 -41.763 0.00 0.00 O

ATOM 69 SI1 PHE P 69 5.314 23.814 -42.704 0.00 0.00 S

ATOM 70 OX1 PHE P 70 5.558 22.324 -43.180 0.00 0.00 O

ATOM 71 OX1 PHE P 71 6.218 25.000 -43.293 0.00 0.00 O

ATOM 72 SI1 PHE P 72 3.899 27.801 -40.221 0.00 0.00 S

ATOM 73 SI1 PHE P 73 4.768 30.482 -39.680 0.00 0.00 S

ATOM 74 OX1 PHE P 74 4.078 29.094 -39.303 0.00 0.00 O

ATOM 75 OX1 PHE P 75 4.876 26.590 -39.860 0.00 0.00 O

ATOM 76 SI1 PHE P 76 7.677 25.512 -42.869 0.00 0.00 S

ATOM 77 SI1 PHE P 77 6.406 26.491 -39.437 0.00 0.00 S

ATOM 78 SI1 PHE P 78 9.245 27.897 -42.957 0.00 0.00 S

ATOM 79 OX1 PHE P 79 8.047 26.958 -43.433 0.00 0.00 O

ATOM 80 OX1 PHE P 80 8.867 24.456 -42.797 0.00 0.00 O

ATOM 81 OX1 PHE P 81 9.000 23.531 -40.345 0.00 0.00 O

ATOM 82 OX1 PHE P 82 6.746 24.922 -39.525 0.00 0.00 O

ATOM 83 OX1 PHE P 83 7.250 27.405 -40.415 0.00 0.00 O

ATOM 84 OX1 PHE P 84 9.107 28.702 -41.590 0.00 0.00 O

ATOM 85 SI1 PHE P 85 9.848 24.051 -41.599 0.00 0.00 S

ATOM 86 OX1 PHE P 86 12.995 21.800 -42.187 0.00 0.00 O

ATOM 87 OX1 PHE P 87 10.597 22.777 -42.237 0.00 0.00 O

ATOM 88 OX1 PHE P 88 10.907 25.157 -41.108 0.00 0.00 O

ATOM 89 OX1 PHE P 89 11.848 25.922 -43.377 0.00 0.00 O

ATOM 90 SI1 PHE P 90 13.471 21.332 -43.622 0.00 0.00 S

ATOM 91 OX1 PHE P 91 13.451 19.771 -43.816 0.00 0.00 O

ATOM 92 OX1 PHE P 92 14.402 22.230 -44.561 0.00 0.00 O

ATOM 93 SI1 PHE P 93 8.694 28.033 -40.211 0.00 0.00 S

ATOM 94 SI1 PHE P 94 12.212 25.733 -41.842 0.00 0.00 S

ATOM 95 SI1 PHE P 95 13.567 28.249 -41.914 0.00 0.00 S

ATOM 96 OX1 PHE P 96 12.623 27.153 -41.227 0.00 0.00 O

ATOM 97 OX1 PHE P 97 13.464 24.740 -41.752 0.00 0.00 O

ATOM 98 SI1 PHE P 98 15.800 22.944 -44.264 0.00 0.00 S

ATOM 99 SI1 PHE P 99 14.411 24.326 -40.524 0.00 0.00 S

ATOM 100 SI1 PHE P 100 16.841 25.416 -43.381 0.00 0.00 S

ATOM 101 OX1 PHE P 101 15.689 24.339 -43.518 0.00 0.00 O

ATOM 102 OX1 PHE P 102 17.110 22.055 -44.040 0.00 0.00 O

ATOM 103 OX1 PHE P 103 16.764 21.048 -41.643 0.00 0.00 O

ATOM 104 OX1 PHE P 104 15.258 23.103 -41.109 0.00 0.00 O

ATOM 105 OX1 PHE P 105 15.351 25.506 -40.035 0.00 0.00 O

ATOM 106 OX1 PHE P 106 16.711 26.442 -42.171 0.00 0.00 O

ATOM 107 SI1 PHE P 107 17.825 21.607 -42.681 0.00 0.00 S

ATOM 108 OX1 PHE P 108 21.133 19.603 -42.749 0.00 0.00 O

ATOM 109 OX1 PHE P 109 18.764 20.379 -43.080 0.00 0.00 O

ATOM 110 OX1 PHE P 110 18.661 22.798 -42.002 0.00 0.00 O

ATOM 111 OX1 PHE P 111 20.834 22.476 -43.435 0.00 0.00 O

ATOM 112 SI1 PHE P 112 21.724 19.221 -44.181 0.00 0.00 S

ATOM 113 OX1 PHE P 113 22.004 17.662 -44.364 0.00 0.00 O

ATOM 114 OX1 PHE P 114 22.714 20.295 -44.836 0.00 0.00 O

ATOM 115 SI1 PHE P 115 16.717 26.067 -40.620 0.00 0.00 S

ATOM 116 OX1 PHE P 116 17.976 25.198 -40.204 0.00 0.00 O

ATOM 117 SI1 PHE P 117 20.201 23.212 -42.178 0.00 0.00 S

ATOM 118 SI1 PHE P 118 21.417 25.752 -42.914 0.00 0.00 S

ATOM 119 OX1 PHE P 119 20.215 24.786 -42.488 0.00 0.00 O

ATOM 120 OX1 PHE P 120 20.897 22.718 -40.814 0.00 0.00 O

ATOM 121 SI1 PHE P 121 24.016 21.008 -44.232 0.00 0.00 S

ATOM 122 SI1 PHE P 122 22.405 22.455 -40.339 0.00 0.00 S

ATOM 123 SI1 PHE P 123 25.154 23.370 -43.355 0.00 0.00 S

ATOM 124 OX1 PHE P 124 23.894 22.413 -43.503 0.00 0.00 O

ATOM 125 OX1 PHE P 125 25.301 20.134 -43.899 0.00 0.00 O

ATOM 126 OX1 PHE P 126 24.784 19.185 -41.482 0.00 0.00 O

ATOM 127 OX1 PHE P 127 23.070 21.150 -41.020 0.00 0.00 O

ATOM 128 OX1 PHE P 128 23.178 23.810 -40.651 0.00 0.00 O

ATOM 129 OX1 PHE P 129 25.152 24.459 -42.200 0.00 0.00 O

ATOM 130 SI1 PHE P 130 25.901 19.686 -42.490 0.00 0.00 S

ATOM 131 OX1 PHE P 131 28.954 17.368 -42.367 0.00 0.00 O

ATOM 132 OX1 PHE P 132 26.742 18.405 -42.893 0.00 0.00 O

ATOM 133 OX1 PHE P 133 26.788 20.765 -41.693 0.00 0.00 O

ATOM 134 OX1 PHE P 134 28.091 22.020 -43.532 0.00 0.00 O

ATOM 135 SI1 PHE P 135 29.512 16.999 -43.801 0.00 0.00 S

ATOM 136 OX1 PHE P 136 29.384 15.482 -44.169 0.00 0.00 O

ATOM 137 OX1 PHE P 137 30.433 17.961 -44.679 0.00 0.00 O

ATOM 138 SI1 PHE P 138 24.722 24.189 -40.693 0.00 0.00 S

ATOM 139 SI1 PHE P 139 28.180 21.474 -42.044 0.00 0.00 S

ATOM 140 SI1 PHE P 140 29.262 23.981 -41.230 0.00 0.00 S

ATOM 141 OX1 PHE P 141 28.370 22.680 -41.016 0.00 0.00 O

ATOM 142 OX1 PHE P 142 29.450 20.510 -41.959 0.00 0.00 O

ATOM 143 SI1 PHE P 143 31.839 18.640 -44.352 0.00 0.00 S

ATOM 144 SI1 PHE P 144 30.243 19.929 -40.694 0.00 0.00 S

ATOM 145 SI1 PHE P 145 32.833 21.247 -44.325 0.00 0.00 S

ATOM 146 OX1 PHE P 146 31.800 20.144 -43.844 0.00 0.00 O

ATOM 147 OX1 PHE P 147 33.134 17.744 -44.075 0.00 0.00 O

ATOM 148 OX1 PHE P 148 32.636 16.686 -41.759 0.00 0.00 O

ATOM 149 OX1 PHE P 149 31.154 18.755 -41.269 0.00 0.00 O

ATOM 150 OX1 PHE P 150 32.603 22.732 -43.818 0.00 0.00 O

ATOM 151 SI1 PHE P 151 33.768 17.305 -42.675 0.00 0.00 S

ATOM 152 OX1 PHE P 152 36.945 14.737 -42.335 0.00 0.00 O

ATOM 153 OX1 PHE P 153 34.859 16.167 -42.929 0.00 0.00 O

ATOM 154 OX1 PHE P 154 34.458 18.517 -41.898 0.00 0.00 O

ATOM 155 OX1 PHE P 155 35.746 19.941 -43.728 0.00 0.00 O

ATOM 156 SI1 PHE P 156 37.280 14.192 -43.791 0.00 0.00 S

ATOM 157 OX1 PHE P 157 38.290 12.970 -43.896 0.00 0.00 O

ATOM 158 OX1 PHE P 158 37.225 15.247 -44.978 0.00 0.00 O

ATOM 159 SI1 PHE P 159 35.801 19.278 -42.288 0.00 0.00 S

ATOM 160 OX1 PHE P 160 37.097 18.385 -42.109 0.00 0.00 O

ATOM 161 SI1 PHE P 161 38.394 16.248 -45.374 0.00 0.00 S

ATOM 162 SI1 PHE P 162 39.579 18.699 -44.754 0.00 0.00 S

ATOM 163 OX1 PHE P 163 38.787 17.387 -44.340 0.00 0.00 O

ATOM 164 OX1 PHE P 164 39.474 15.767 -46.432 0.00 0.00 O

ATOM 165 SI1 PHE P 165 -20.469 22.862 -44.275 0.00 0.00 S

ATOM 166 OX1 PHE P 166 -20.268 21.286 -44.289 0.00 0.00 O

ATOM 167 OX1 PHE P 167 -19.625 23.718 -45.311 0.00 0.00 O

ATOM 168 OX1 PHE P 168 -16.361 23.121 -44.651 0.00 0.00 O

ATOM 169 SI1 PHE P 169 -16.228 22.778 -43.107 0.00 0.00 S

ATOM 170 OX1 PHE P 170 -12.835 20.930 -41.770 0.00 0.00 O

ATOM 171 OX1 PHE P 171 -14.906 21.922 -42.896 0.00 0.00 O

ATOM 172 OX1 PHE P 172 -16.129 24.132 -42.275 0.00 0.00 O

ATOM 173 OX1 PHE P 173 -14.531 25.581 -43.809 0.00 0.00 O

ATOM 174 SI1 PHE P 174 -12.120 20.550 -43.135 0.00 0.00 S

ATOM 175 OX1 PHE P 175 -10.771 19.732 -43.029 0.00 0.00 O

ATOM 176 OX1 PHE P 176 -12.248 21.577 -44.338 0.00 0.00 O

ATOM 177 SI1 PHE P 177 -14.902 25.151 -42.327 0.00 0.00 S

ATOM 178 OX1 PHE P 178 -13.631 24.668 -41.514 0.00 0.00 O

ATOM 179 SI1 PHE P 179 -10.318 25.100 -42.591 0.00 0.00 S

ATOM 180 OX1 PHE P 180 -10.539 23.633 -43.146 0.00 0.00 O

ATOM 181 OX1 PHE P 181 -8.092 22.329 -42.213 0.00 0.00 O

ATOM 182 OX1 PHE P 182 -8.913 20.858 -40.274 0.00 0.00 O

ATOM 183 OX1 PHE P 183 -10.677 22.818 -39.960 0.00 0.00 O

ATOM 184 OX1 PHE P 184 -9.023 25.377 -41.716 0.00 0.00 O

ATOM 185 SI1 PHE P 185 -7.637 21.580 -40.891 0.00 0.00 S

ATOM 186 OX1 PHE P 186 -5.021 18.550 -41.280 0.00 0.00 O

ATOM 187 OX1 PHE P 187 -6.674 20.391 -41.297 0.00 0.00 O

ATOM 188 OX1 PHE P 188 -6.843 22.640 -39.980 0.00 0.00 O

ATOM 189 OX1 PHE P 189 -5.672 23.908 -41.923 0.00 0.00 O

ATOM 190 SI1 PHE P 190 -4.498 18.700 -42.772 0.00 0.00 S

ATOM 191 OX1 PHE P 191 -3.128 17.941 -43.021 0.00 0.00 O

ATOM 192 OX1 PHE P 192 -4.600 20.149 -43.414 0.00 0.00 O

ATOM 193 SI1 PHE P 193 -5.482 23.376 -40.436 0.00 0.00 S

ATOM 194 SI1 PHE P 194 -5.022 26.200 -39.811 0.00 0.00 S

ATOM 195 OX1 PHE P 195 -5.132 24.624 -39.498 0.00 0.00 O

ATOM 196 OX1 PHE P 196 -4.260 22.346 -40.471 0.00 0.00 O

ATOM 197 OX1 PHE P 197 -4.295 20.709 -38.380 0.00 0.00 O

ATOM 198 OX1 PHE P 198 -5.056 26.874 -38.374 0.00 0.00 O

ATOM 199 SI1 PHE P 199 -3.417 21.582 -39.342 0.00 0.00 S

ATOM 200 SI1 PHE P 200 -1.828 24.052 -41.699 0.00 0.00 S

ATOM 201 OX1 PHE P 201 -1.699 22.567 -42.240 0.00 0.00 O

ATOM 202 OX1 PHE P 202 -0.373 20.034 -43.285 0.00 0.00 O

ATOM 203 OX1 PHE P 203 -1.013 18.745 -41.118 0.00 0.00 O

ATOM 204 OX1 PHE P 204 -2.458 20.641 -40.194 0.00 0.00 O

ATOM 205 OX1 PHE P 205 -2.637 22.576 -38.377 0.00 0.00 O

ATOM 206 OX1 PHE P 206 -1.933 24.312 -40.138 0.00 0.00 O

ATOM 207 SI1 PHE P 207 0.186 19.366 -41.958 0.00 0.00 S

ATOM 208 OX1 PHE P 208 2.930 16.556 -41.709 0.00 0.00 O

ATOM 209 OX1 PHE P 209 0.992 18.042 -42.297 0.00 0.00 O

ATOM 210 OX1 PHE P 210 1.082 20.453 -41.179 0.00 0.00 O

ATOM 211 OX1 PHE P 211 2.236 21.795 -43.089 0.00 0.00 O

ATOM 212 SI1 PHE P 212 3.403 16.543 -43.238 0.00 0.00 S

ATOM 213 OX1 PHE P 213 4.700 15.729 -43.623 0.00 0.00 O

ATOM 214 OX1 PHE P 214 3.238 17.909 -44.030 0.00 0.00 O

ATOM 215 SI1 PHE P 215 -1.452 23.505 -38.860 0.00 0.00 S

ATOM 216 SI1 PHE P 216 -0.152 25.861 -38.254 0.00 0.00 S

ATOM 217 OX1 PHE P 217 -1.030 24.611 -37.787 0.00 0.00 O

ATOM 218 OX1 PHE P 218 -0.173 22.592 -39.131 0.00 0.00 O

ATOM 219 SI1 PHE P 219 2.421 21.247 -41.611 0.00 0.00 S

ATOM 220 SI1 PHE P 220 0.660 21.885 -37.967 0.00 0.00 S

ATOM 221 SI1 PHE P 221 3.374 23.861 -40.579 0.00 0.00 S

ATOM 222 OX1 PHE P 222 2.590 22.467 -40.592 0.00 0.00 O

ATOM 223 OX1 PHE P 223 3.733 20.330 -41.503 0.00 0.00 O

ATOM 224 OX1 PHE P 224 3.603 19.164 -39.098 0.00 0.00 O

ATOM 225 OX1 PHE P 225 1.666 20.843 -38.623 0.00 0.00 O

ATOM 226 OX1 PHE P 226 3.558 24.184 -39.032 0.00 0.00 O

ATOM 227 SI1 PHE P 227 4.532 19.810 -40.204 0.00 0.00 S

ATOM 228 SI1 PHE P 228 6.772 21.477 -42.619 0.00 0.00 S

ATOM 229 OX1 PHE P 229 6.971 20.040 -43.222 0.00 0.00 O

ATOM 230 OX1 PHE P 230 7.510 17.525 -43.497 0.00 0.00 O

ATOM 231 OX1 PHE P 231 7.057 16.601 -41.107 0.00 0.00 O

ATOM 232 OX1 PHE P 232 5.472 18.615 -40.730 0.00 0.00 O

ATOM 233 OX1 PHE P 233 5.334 21.004 -39.487 0.00 0.00 O

ATOM 234 OX1 PHE P 234 7.281 21.807 -41.152 0.00 0.00 O

ATOM 235 SI1 PHE P 235 8.174 17.100 -42.124 0.00 0.00 S

ATOM 236 OX1 PHE P 236 10.948 14.305 -41.916 0.00 0.00 O

ATOM 237 OX1 PHE P 237 8.959 15.758 -42.460 0.00 0.00 O

ATOM 238 OX1 PHE P 238 9.098 18.284 -41.538 0.00 0.00 O

ATOM 239 OX1 PHE P 239 10.204 19.284 -43.673 0.00 0.00 O

ATOM 240 SI1 PHE P 240 11.372 14.220 -43.456 0.00 0.00 S

ATOM 241 OX1 PHE P 241 12.691 13.438 -43.846 0.00 0.00 O

ATOM 242 OX1 PHE P 242 11.117 15.526 -44.321 0.00 0.00 O

ATOM 243 SI1 PHE P 243 6.875 21.386 -39.663 0.00 0.00 S

ATOM 244 SI1 PHE P 244 8.006 24.001 -39.166 0.00 0.00 S

ATOM 245 OX1 PHE P 245 7.379 22.587 -38.704 0.00 0.00 O

ATOM 246 OX1 PHE P 246 7.648 20.066 -39.219 0.00 0.00 O

ATOM 247 SI1 PHE P 247 10.441 18.942 -42.141 0.00 0.00 S

ATOM 248 SI1 PHE P 248 9.031 19.909 -38.444 0.00 0.00 S

ATOM 249 SI1 PHE P 249 11.553 21.721 -41.514 0.00 0.00 S

ATOM 250 OX1 PHE P 250 10.806 20.288 -41.344 0.00 0.00 O

ATOM 251 OX1 PHE P 251 11.621 17.878 -41.961 0.00 0.00 O

ATOM 252 OX1 PHE P 252 11.545 16.876 -39.426 0.00 0.00 O

ATOM 253 OX1 PHE P 253 9.467 18.427 -38.827 0.00 0.00 O

ATOM 254 OX1 PHE P 254 9.961 21.028 -39.021 0.00 0.00 O

ATOM 255 OX1 PHE P 255 11.789 22.295 -40.038 0.00 0.00 O

ATOM 256 SI1 PHE P 256 12.410 17.432 -40.633 0.00 0.00 S

ATOM 257 SI1 PHE P 257 14.493 18.684 -43.325 0.00 0.00 S

ATOM 258 OX1 PHE P 258 13.945 17.279 -43.800 0.00 0.00 O

ATOM 259 OX1 PHE P 259 15.787 15.472 -43.852 0.00 0.00 O

ATOM 260 OX1 PHE P 260 15.129 14.438 -41.498 0.00 0.00 O

ATOM 261 OX1 PHE P 261 13.399 16.268 -41.112 0.00 0.00 O

ATOM 262 OX1 PHE P 262 13.192 18.712 -40.137 0.00 0.00 O

ATOM 263 OX1 PHE P 263 15.209 18.694 -41.885 0.00 0.00 O

ATOM 264 SI1 PHE P 264 16.312 14.959 -42.453 0.00 0.00 S

ATOM 265 OX1 PHE P 265 19.010 12.240 -42.135 0.00 0.00 O

ATOM 266 OX1 PHE P 266 17.021 13.577 -42.771 0.00 0.00 O

ATOM 267 OX1 PHE P 267 17.313 16.063 -41.828 0.00 0.00 O

ATOM 268 OX1 PHE P 268 18.660 16.783 -43.946 0.00 0.00 O

ATOM 269 SI1 PHE P 269 19.529 12.043 -43.632 0.00 0.00 S

ATOM 270 OX1 PHE P 270 20.926 11.319 -43.840 0.00 0.00 O

ATOM 271 OX1 PHE P 271 19.261 13.258 -44.619 0.00 0.00 O

ATOM 272 SI1 PHE P 272 11.404 21.567 -38.683 0.00 0.00 S

ATOM 273 OX1 PHE P 273 12.507 20.538 -38.214 0.00 0.00 O

ATOM 274 SI1 PHE P 274 14.691 19.132 -40.426 0.00 0.00 S

ATOM 275 SI1 PHE P 275 15.879 21.758 -40.524 0.00 0.00 S

ATOM 276 OX1 PHE P 276 14.693 20.726 -40.275 0.00 0.00 O

ATOM 277 OX1 PHE P 277 15.472 18.395 -39.248 0.00 0.00 O

ATOM 278 SI1 PHE P 278 18.724 16.657 -42.364 0.00 0.00 S

ATOM 279 SI1 PHE P 279 16.989 18.189 -38.817 0.00 0.00 S

ATOM 280 SI1 PHE P 280 19.675 19.496 -42.112 0.00 0.00 S

ATOM 281 OX1 PHE P 281 18.953 18.103 -41.736 0.00 0.00 O

ATOM 282 OX1 PHE P 282 19.969 15.752 -41.915 0.00 0.00 O

ATOM 283 OX1 PHE P 283 19.520 14.962 -39.435 0.00 0.00 O

ATOM 284 OX1 PHE P 284 17.448 16.702 -39.239 0.00 0.00 O

ATOM 285 OX1 PHE P 285 17.841 19.331 -39.503 0.00 0.00 O

ATOM 286 OX1 PHE P 286 19.873 20.251 -40.719 0.00 0.00 O

ATOM 287 SI1 PHE P 287 20.615 15.450 -40.466 0.00 0.00 S

ATOM 288 SI1 PHE P 288 22.783 16.837 -43.248 0.00 0.00 S

ATOM 289 OX1 PHE P 289 22.648 15.262 -43.334 0.00 0.00 O

ATOM 290 OX1 PHE P 290 23.481 12.497 -43.187 0.00 0.00 O

ATOM 291 OX1 PHE P 291 23.335 12.363 -40.556 0.00 0.00 O

ATOM 292 OX1 PHE P 292 21.667 14.285 -40.724 0.00 0.00 O

ATOM 293 OX1 PHE P 293 21.438 16.641 -39.789 0.00 0.00 O

ATOM 294 OX1 PHE P 294 22.629 17.576 -41.850 0.00 0.00 O

ATOM 295 SI1 PHE P 295 24.257 12.675 -41.816 0.00 0.00 S

ATOM 296 OX1 PHE P 296 26.961 9.734 -41.806 0.00 0.00 O

ATOM 297 OX1 PHE P 297 25.454 11.652 -41.743 0.00 0.00 O

ATOM 298 OX1 PHE P 298 24.971 14.100 -41.679 0.00 0.00 O

ATOM 299 OX1 PHE P 299 26.063 14.858 -43.929 0.00 0.00 O

ATOM 300 SI1 PHE P 300 27.452 9.748 -43.316 0.00 0.00 S

ATOM 301 OX1 PHE P 301 28.858 9.033 -43.479 0.00 0.00 O

ATOM 302 OX1 PHE P 302 27.243 11.093 -44.132 0.00 0.00 O

ATOM 303 SI1 PHE P 303 19.346 19.787 -39.296 0.00 0.00 S

ATOM 304 SI1 PHE P 304 22.805 17.289 -40.302 0.00 0.00 S

ATOM 305 SI1 PHE P 305 23.915 19.931 -40.369 0.00 0.00 S

ATOM 306 OX1 PHE P 306 22.994 18.779 -39.714 0.00 0.00 O

ATOM 307 OX1 PHE P 307 24.081 16.344 -40.051 0.00 0.00 O

ATOM 308 SI1 PHE P 308 26.309 14.663 -42.374 0.00 0.00 S

ATOM 309 SI1 PHE P 309 24.980 15.963 -38.768 0.00 0.00 S

ATOM 310 SI1 PHE P 310 27.441 17.398 -41.889 0.00 0.00 S

ATOM 311 OX1 PHE P 311 26.601 16.057 -41.656 0.00 0.00 O

ATOM 312 OX1 PHE P 312 27.520 13.637 -42.123 0.00 0.00 O

ATOM 313 OX1 PHE P 313 27.239 12.579 -39.699 0.00 0.00 O

ATOM 314 OX1 PHE P 314 25.961 14.783 -39.213 0.00 0.00 O

ATOM 315 OX1 PHE P 315 25.759 17.209 -38.166 0.00 0.00 O

ATOM 316 OX1 PHE P 316 27.444 17.981 -40.417 0.00 0.00 O

ATOM 317 SI1 PHE P 317 28.260 13.114 -40.789 0.00 0.00 S

ATOM 318 SI1 PHE P 318 30.254 14.317 -43.554 0.00 0.00 S

ATOM 319 OX1 PHE P 319 29.632 12.931 -43.996 0.00 0.00 O

ATOM 320 OX1 PHE P 320 31.491 11.166 -43.689 0.00 0.00 O

ATOM 321 OX1 PHE P 321 31.002 10.068 -41.370 0.00 0.00 O

ATOM 322 OX1 PHE P 322 29.199 11.906 -41.254 0.00 0.00 O

ATOM 323 OX1 PHE P 323 29.133 14.264 -40.137 0.00 0.00 O

ATOM 324 OX1 PHE P 324 30.976 14.385 -42.119 0.00 0.00 O

ATOM 325 SI1 PHE P 325 32.117 10.688 -42.321 0.00 0.00 S

ATOM 326 OX1 PHE P 326 34.974 7.948 -42.399 0.00 0.00 O

ATOM 327 OX1 PHE P 327 33.049 9.477 -42.717 0.00 0.00 O

ATOM 328 OX1 PHE P 328 32.945 11.886 -41.636 0.00 0.00 O

ATOM 329 OX1 PHE P 329 33.729 13.157 -43.755 0.00 0.00 O

ATOM 330 SI1 PHE P 330 35.238 7.545 -43.907 0.00 0.00 S

ATOM 331 OX1 PHE P 331 36.602 6.766 -44.101 0.00 0.00 O

ATOM 332 OX1 PHE P 332 34.855 8.618 -45.010 0.00 0.00 O

ATOM 333 SI1 PHE P 333 30.544 14.810 -40.623 0.00 0.00 S

ATOM 334 SI1 PHE P 334 31.720 17.393 -40.664 0.00 0.00 S

ATOM 335 OX1 PHE P 335 30.504 16.392 -40.433 0.00 0.00 O

ATOM 336 OX1 PHE P 336 31.660 14.221 -39.659 0.00 0.00 O

ATOM 337 SI1 PHE P 337 34.170 12.685 -42.303 0.00 0.00 S

ATOM 338 SI1 PHE P 338 35.512 15.211 -41.821 0.00 0.00 S

ATOM 339 OX1 PHE P 339 34.544 13.985 -41.447 0.00 0.00 O

ATOM 340 OX1 PHE P 340 35.436 11.714 -42.431 0.00 0.00 O

ATOM 341 SI1 PHE P 341 36.450 11.232 -41.289 0.00 0.00 S

ATOM 342 SI1 PHE P 342 39.826 13.028 -43.502 0.00 0.00 S

ATOM 343 OX1 PHE P 343 40.875 13.560 -44.566 0.00 0.00 O

ATOM 344 OX1 PHE P 344 39.540 9.334 -44.166 0.00 0.00 O

ATOM 345 OX1 PHE P 345 38.803 7.947 -42.073 0.00 0.00 O

ATOM 346 OX1 PHE P 346 37.222 9.938 -41.810 0.00 0.00 O

ATOM 347 OX1 PHE P 347 37.427 12.379 -40.797 0.00 0.00 O

ATOM 348 OX1 PHE P 348 40.237 12.997 -41.969 0.00 0.00 O

ATOM 349 SI1 PHE P 349 40.032 8.535 -42.886 0.00 0.00 S

ATOM 350 OX1 PHE P 350 42.815 5.631 -42.857 0.00 0.00 O

ATOM 351 OX1 PHE P 351 40.902 7.279 -43.308 0.00 0.00 O

ATOM 352 OX1 PHE P 352 40.924 9.493 -41.972 0.00 0.00 O

ATOM 353 OX1 PHE P 353 42.383 10.843 -43.723 0.00 0.00 O

ATOM 354 SI1 PHE P 354 43.193 5.437 -44.391 0.00 0.00 S

ATOM 355 OX1 PHE P 355 44.532 4.634 -44.656 0.00 0.00 O

ATOM 356 OX1 PHE P 356 42.977 6.703 -45.321 0.00 0.00 O

ATOM 357 SI1 PHE P 357 42.369 10.058 -42.344 0.00 0.00 S

ATOM 358 OX1 PHE P 358 43.012 10.905 -41.170 0.00 0.00 O

ATOM 359 OX1 PHE P 359 43.498 8.947 -42.288 0.00 0.00 O

ATOM 360 OX1 PHE P 360 -28.470 16.322 -46.151 0.00 0.00 O

ATOM 361 OX1 PHE P 361 -22.515 15.619 -43.431 0.00 0.00 O

ATOM 362 OX1 PHE P 362 -23.784 17.743 -44.304 0.00 0.00 O

ATOM 363 SI1 PHE P 363 -21.183 15.381 -44.284 0.00 0.00 S

ATOM 364 OX1 PHE P 364 -20.114 14.430 -43.596 0.00 0.00 O

ATOM 365 OX1 PHE P 365 -20.502 16.636 -44.974 0.00 0.00 O

ATOM 366 SI1 PHE P 366 -19.836 17.831 -44.161 0.00 0.00 S

ATOM 367 SI1 PHE P 367 -20.523 20.312 -43.056 0.00 0.00 S

ATOM 368 OX1 PHE P 368 -20.858 18.803 -43.426 0.00 0.00 O

ATOM 369 OX1 PHE P 369 -18.385 17.692 -43.502 0.00 0.00 O

ATOM 370 OX1 PHE P 370 -19.145 17.111 -41.082 0.00 0.00 O

ATOM 371 OX1 PHE P 371 -19.882 20.644 -41.640 0.00 0.00 O

ATOM 372 SI1 PHE P 372 -17.946 17.019 -42.118 0.00 0.00 S

ATOM 373 OX1 PHE P 373 -15.086 14.748 -41.882 0.00 0.00 O

ATOM 374 OX1 PHE P 374 -17.522 15.476 -42.292 0.00 0.00 O

ATOM 375 OX1 PHE P 375 -16.844 17.977 -41.462 0.00 0.00 O

ATOM 376 OX1 PHE P 376 -15.414 18.642 -43.522 0.00 0.00 O

ATOM 377 SI1 PHE P 377 -14.480 14.270 -43.291 0.00 0.00 S

ATOM 378 OX1 PHE P 378 -14.310 12.690 -43.451 0.00 0.00 O

ATOM 379 OX1 PHE P 379 -13.335 15.207 -43.911 0.00 0.00 O

ATOM 380 SI1 PHE P 380 -15.407 18.466 -41.943 0.00 0.00 S

ATOM 381 SI1 PHE P 381 -14.386 21.196 -41.576 0.00 0.00 S

ATOM 382 OX1 PHE P 382 -15.248 19.893 -41.248 0.00 0.00 O

ATOM 383 OX1 PHE P 383 -14.260 17.438 -41.511 0.00 0.00 O

ATOM 384 SI1 PHE P 384 -11.988 15.760 -43.238 0.00 0.00 S

ATOM 385 SI1 PHE P 385 -13.650 17.089 -40.068 0.00 0.00 S

ATOM 386 SI1 PHE P 386 -10.820 18.273 -42.425 0.00 0.00 S

ATOM 387 OX1 PHE P 387 -11.843 17.341 -43.215 0.00 0.00 O

ATOM 388 OX1 PHE P 388 -10.716 14.828 -43.005 0.00 0.00 O

ATOM 389 OX1 PHE P 389 -10.831 14.087 -40.473 0.00 0.00 O

ATOM 390 OX1 PHE P 390 -12.694 15.833 -40.326 0.00 0.00 O

ATOM 391 OX1 PHE P 391 -12.883 18.389 -39.591 0.00 0.00 O

ATOM 392 OX1 PHE P 392 -10.570 18.094 -40.857 0.00 0.00 O

ATOM 393 SI1 PHE P 393 -9.877 14.568 -41.666 0.00 0.00 S

ATOM 394 OX1 PHE P 394 -6.850 12.011 -41.435 0.00 0.00 O

ATOM 395 OX1 PHE P 395 -8.981 13.296 -42.031 0.00 0.00 O

ATOM 396 OX1 PHE P 396 -8.985 15.793 -41.125 0.00 0.00 O

ATOM 397 OX1 PHE P 397 -7.672 16.752 -43.170 0.00 0.00 O

ATOM 398 SI1 PHE P 398 -6.229 12.015 -42.920 0.00 0.00 S

ATOM 399 OX1 PHE P 399 -5.174 10.903 -43.297 0.00 0.00 O

ATOM 400 OX1 PHE P 400 -5.698 13.384 -43.539 0.00 0.00 O

ATOM 401 SI1 PHE P 401 -11.329 18.693 -39.583 0.00 0.00 S

ATOM 402 SI1 PHE P 402 -10.175 21.391 -39.479 0.00 0.00 S

ATOM 403 OX1 PHE P 403 -11.325 20.295 -39.568 0.00 0.00 O

ATOM 404 OX1 PHE P 404 -10.771 17.990 -38.259 0.00 0.00 O

ATOM 405 SI1 PHE P 405 -7.620 16.500 -41.604 0.00 0.00 S

ATOM 406 SI1 PHE P 406 -9.290 17.702 -37.736 0.00 0.00 S

ATOM 407 SI1 PHE P 407 -6.399 18.968 -40.622 0.00 0.00 S

ATOM 408 OX1 PHE P 408 -7.581 17.918 -40.859 0.00 0.00 O

ATOM 409 OX1 PHE P 409 -6.326 15.604 -41.298 0.00 0.00 O

ATOM 410 OX1 PHE P 410 -6.593 14.650 -38.873 0.00 0.00 O

ATOM 411 OX1 PHE P 411 -8.557 16.453 -38.461 0.00 0.00 O

ATOM 412 OX1 PHE P 412 -8.554 19.076 -37.968 0.00 0.00 O

ATOM 413 OX1 PHE P 413 -6.140 18.937 -39.040 0.00 0.00 O

ATOM 414 SI1 PHE P 414 -4.221 13.968 -43.312 0.00 0.00 S

ATOM 415 SI1 PHE P 415 -5.568 15.166 -39.954 0.00 0.00 S

ATOM 416 SI1 PHE P 416 -2.980 16.422 -42.594 0.00 0.00 S

ATOM 417 OX1 PHE P 417 -4.091 15.541 -43.306 0.00 0.00 O

ATOM 418 OX1 PHE P 418 -2.925 13.029 -43.255 0.00 0.00 O

ATOM 419 OX1 PHE P 419 -3.078 12.031 -40.827 0.00 0.00 O

ATOM 420 OX1 PHE P 420 -4.698 13.908 -40.389 0.00 0.00 O

ATOM 421 OX1 PHE P 421 -4.605 16.313 -39.396 0.00 0.00 O

ATOM 422 OX1 PHE P 422 -2.517 16.087 -41.095 0.00 0.00 O

ATOM 423 SI1 PHE P 423 -2.086 12.567 -41.967 0.00 0.00 S

ATOM 424 OX1 PHE P 424 0.939 10.302 -41.735 0.00 0.00 O

ATOM 425 OX1 PHE P 425 -1.333 11.214 -42.358 0.00 0.00 O

ATOM 426 OX1 PHE P 426 -1.047 13.658 -41.378 0.00 0.00 O

ATOM 427 OX1 PHE P 427 0.178 14.629 -43.456 0.00 0.00 O

ATOM 428 SI1 PHE P 428 1.557 9.934 -43.168 0.00 0.00 S

ATOM 429 OX1 PHE P 429 1.369 8.440 -43.628 0.00 0.00 O

ATOM 430 OX1 PHE P 430 2.475 10.899 -44.065 0.00 0.00 O

ATOM 431 SI1 PHE P 431 -7.039 19.475 -37.830 0.00 0.00 S

ATOM 432 SI1 PHE P 432 -3.049 16.604 -39.674 0.00 0.00 S

ATOM 433 SI1 PHE P 433 -1.817 19.230 -39.847 0.00 0.00 S

ATOM 434 OX1 PHE P 434 -2.997 18.197 -39.614 0.00 0.00 O

ATOM 435 OX1 PHE P 435 -2.286 15.876 -38.471 0.00 0.00 O

ATOM 436 OX1 PHE P 436 -1.122 14.931 -36.473 0.00 0.00 O

ATOM 437 OX1 PHE P 437 -0.900 19.231 -38.564 0.00 0.00 O

ATOM 438 SI1 PHE P 438 0.338 14.309 -41.909 0.00 0.00 S

ATOM 439 SI1 PHE P 439 -0.827 15.472 -37.936 0.00 0.00 S

ATOM 440 SI1 PHE P 440 1.460 16.932 -41.238 0.00 0.00 S

ATOM 441 OX1 PHE P 441 0.484 15.676 -41.101 0.00 0.00 O

ATOM 442 OX1 PHE P 442 1.667 13.411 -41.688 0.00 0.00 O

ATOM 443 OX1 PHE P 443 1.583 12.378 -39.268 0.00 0.00 O

ATOM 444 OX1 PHE P 444 -0.180 14.282 -38.774 0.00 0.00 O

ATOM 445 OX1 PHE P 445 0.196 16.650 -37.620 0.00 0.00 O

ATOM 446 OX1 PHE P 446 1.417 17.631 -39.802 0.00 0.00 O

ATOM 447 SI1 PHE P 447 3.893 11.606 -43.815 0.00 0.00 S

ATOM 448 SI1 PHE P 448 2.529 12.931 -40.401 0.00 0.00 S

ATOM 449 SI1 PHE P 449 4.883 14.231 -43.166 0.00 0.00 S

ATOM 450 OX1 PHE P 450 3.830 13.197 -43.755 0.00 0.00 O

ATOM 451 OX1 PHE P 451 5.262 10.774 -43.670 0.00 0.00 O

ATOM 452 OX1 PHE P 452 4.953 9.719 -41.320 0.00 0.00 O

ATOM 453 OX1 PHE P 453 3.408 11.704 -40.893 0.00 0.00 O

ATOM 454 OX1 PHE P 454 3.539 13.999 -39.774 0.00 0.00 O

ATOM 455 OX1 PHE P 455 5.437 14.046 -41.696 0.00 0.00 O

ATOM 456 SI1 PHE P 456 6.031 10.287 -42.346 0.00 0.00 S

ATOM 457 OX1 PHE P 457 9.066 7.983 -41.962 0.00 0.00 O

ATOM 458 OX1 PHE P 458 6.869 8.970 -42.678 0.00 0.00 O

ATOM 459 OX1 PHE P 459 7.011 11.378 -41.671 0.00 0.00 O

ATOM 460 OX1 PHE P 460 8.321 12.337 -43.693 0.00 0.00 O

ATOM 461 SI1 PHE P 461 9.735 7.589 -43.365 0.00 0.00 S

ATOM 462 OX1 PHE P 462 9.612 6.078 -43.777 0.00 0.00 O

ATOM 463 OX1 PHE P 463 10.679 8.532 -44.253 0.00 0.00 O

ATOM 464 SI1 PHE P 464 1.555 17.163 -38.279 0.00 0.00 S

ATOM 465 SI1 PHE P 465 2.645 19.774 -37.979 0.00 0.00 S

ATOM 466 OX1 PHE P 466 1.835 18.508 -37.444 0.00 0.00 O

ATOM 467 OX1 PHE P 467 2.787 16.138 -38.122 0.00 0.00 O

ATOM 468 SI1 PHE P 468 5.000 14.489 -40.224 0.00 0.00 S

ATOM 469 SI1 PHE P 469 3.768 16.032 -36.857 0.00 0.00 S

ATOM 470 SI1 PHE P 470 6.022 17.241 -40.085 0.00 0.00 S

ATOM 471 OX1 PHE P 471 4.924 16.087 -40.001 0.00 0.00 O

ATOM 472 OX1 PHE P 472 6.231 13.645 -39.629 0.00 0.00 O

ATOM 473 OX1 PHE P 473 6.374 12.827 -37.037 0.00 0.00 O

ATOM 474 OX1 PHE P 474 4.916 14.932 -36.919 0.00 0.00 O

ATOM 475 OX1 PHE P 475 4.533 17.416 -36.794 0.00 0.00 O

ATOM 476 OX1 PHE P 476 6.633 17.354 -38.609 0.00 0.00 O

ATOM 477 SI1 PHE P 477 8.422 12.014 -42.141 0.00 0.00 S

ATOM 478 SI1 PHE P 478 7.131 13.271 -38.357 0.00 0.00 S

ATOM 479 SI1 PHE P 479 9.473 14.651 -41.431 0.00 0.00 S

ATOM 480 OX1 PHE P 480 8.527 13.375 -41.325 0.00 0.00 O

ATOM 481 OX1 PHE P 481 9.747 11.115 -41.874 0.00 0.00 O

ATOM 482 OX1 PHE P 482 9.630 10.148 -39.417 0.00 0.00 O

ATOM 483 OX1 PHE P 483 7.926 12.050 -38.987 0.00 0.00 O

ATOM 484 OX1 PHE P 484 8.148 14.387 -37.857 0.00 0.00 O

ATOM 485 OX1 PHE P 485 9.401 15.342 -40.000 0.00 0.00 O

ATOM 486 SI1 PHE P 486 12.078 9.257 -43.963 0.00 0.00 S

ATOM 487 SI1 PHE P 487 10.604 10.650 -40.571 0.00 0.00 S

ATOM 488 SI1 PHE P 488 12.940 11.948 -43.387 0.00 0.00 S

ATOM 489 OX1 PHE P 489 11.962 10.848 -43.983 0.00 0.00 O

ATOM 490 OX1 PHE P 490 13.459 8.451 -43.750 0.00 0.00 O

ATOM 491 OX1 PHE P 491 13.155 7.390 -41.421 0.00 0.00 O

ATOM 492 OX1 PHE P 492 11.516 9.428 -41.064 0.00 0.00 O

ATOM 493 OX1 PHE P 493 11.620 11.736 -39.981 0.00 0.00 O

ATOM 494 OX1 PHE P 494 13.509 11.763 -41.926 0.00 0.00 O

ATOM 495 SI1 PHE P 495 14.230 8.027 -42.399 0.00 0.00 S

ATOM 496 OX1 PHE P 496 17.291 5.210 -42.534 0.00 0.00 O

ATOM 497 OX1 PHE P 497 15.352 6.939 -42.753 0.00 0.00 O

ATOM 498 OX1 PHE P 498 15.013 9.242 -41.725 0.00 0.00 O

ATOM 499 OX1 PHE P 499 16.182 10.205 -43.843 0.00 0.00 O

ATOM 500 SI1 PHE P 500 18.166 5.181 -43.871 0.00 0.00 S

ATOM 501 OX1 PHE P 501 19.322 4.096 -43.779 0.00 0.00 O

ATOM 502 OX1 PHE P 502 18.792 6.526 -44.471 0.00 0.00 O

ATOM 503 SI1 PHE P 503 6.018 17.830 -37.196 0.00 0.00 S

ATOM 504 SI1 PHE P 504 9.528 14.882 -38.479 0.00 0.00 S

ATOM 505 SI1 PHE P 505 10.615 17.487 -38.274 0.00 0.00 S

ATOM 506 OX1 PHE P 506 9.862 16.220 -37.668 0.00 0.00 O

ATOM 507 OX1 PHE P 507 10.741 13.838 -38.296 0.00 0.00 O

ATOM 508 SI1 PHE P 508 13.085 12.202 -40.454 0.00 0.00 S

ATOM 509 SI1 PHE P 509 11.811 13.861 -37.098 0.00 0.00 S

ATOM 510 SI1 PHE P 510 14.082 14.989 -40.428 0.00 0.00 S

ATOM 511 OX1 PHE P 511 13.022 13.803 -40.266 0.00 0.00 O

ATOM 512 OX1 PHE P 512 14.313 11.365 -39.842 0.00 0.00 O

ATOM 513 OX1 PHE P 513 14.440 10.700 -37.214 0.00 0.00 O

ATOM 514 OX1 PHE P 514 12.942 12.748 -37.149 0.00 0.00 O

ATOM 515 OX1 PHE P 515 12.573 15.247 -37.239 0.00 0.00 O

ATOM 516 OX1 PHE P 516 14.728 15.186 -38.971 0.00 0.00 O

ATOM 517 SI1 PHE P 517 16.362 9.888 -42.296 0.00 0.00 S

ATOM 518 SI1 PHE P 518 15.201 11.163 -38.528 0.00 0.00 S

ATOM 519 SI1 PHE P 519 17.520 12.537 -41.674 0.00 0.00 S

ATOM 520 OX1 PHE P 520 16.584 11.254 -41.504 0.00 0.00 O

ATOM 521 OX1 PHE P 521 17.565 8.842 -42.090 0.00 0.00 O

ATOM 522 OX1 PHE P 522 17.769 8.074 -39.446 0.00 0.00 O

ATOM 523 OX1 PHE P 523 16.318 10.105 -38.917 0.00 0.00 O

ATOM 524 OX1 PHE P 524 16.012 12.472 -38.145 0.00 0.00 O

ATOM 525 OX1 PHE P 525 17.420 13.315 -40.279 0.00 0.00 O

ATOM 526 SI1 PHE P 526 20.181 7.212 -44.031 0.00 0.00 S

ATOM 527 SI1 PHE P 527 18.472 8.544 -40.797 0.00 0.00 S

ATOM 528 SI1 PHE P 528 21.191 9.838 -43.343 0.00 0.00 S

ATOM 529 OX1 PHE P 529 20.204 8.803 -44.049 0.00 0.00 O

ATOM 530 OX1 PHE P 530 21.512 6.337 -43.801 0.00 0.00 O

ATOM 531 OX1 PHE P 531 21.097 5.359 -41.423 0.00 0.00 O

ATOM 532 OX1 PHE P 532 19.541 7.418 -41.163 0.00 0.00 O

ATOM 533 OX1 PHE P 533 19.237 9.921 -40.601 0.00 0.00 O

ATOM 534 OX1 PHE P 534 21.568 9.664 -41.802 0.00 0.00 O

ATOM 535 SI1 PHE P 535 22.204 5.913 -42.410 0.00 0.00 S

ATOM 536 OX1 PHE P 536 25.134 3.210 -42.009 0.00 0.00 O

ATOM 537 OX1 PHE P 537 23.176 4.673 -42.644 0.00 0.00 O

ATOM 538 OX1 PHE P 538 23.027 7.097 -41.719 0.00 0.00 O

ATOM 539 OX1 PHE P 539 24.530 7.858 -43.699 0.00 0.00 O

ATOM 540 SI1 PHE P 540 25.778 3.109 -43.490 0.00 0.00 S

ATOM 541 OX1 PHE P 541 26.528 1.739 -43.834 0.00 0.00 O

ATOM 542 OX1 PHE P 542 26.618 4.373 -43.986 0.00 0.00 O

ATOM 543 SI1 PHE P 543 14.088 15.632 -37.558 0.00 0.00 S

ATOM 544 SI1 PHE P 544 17.413 12.977 -38.720 0.00 0.00 S

ATOM 545 SI1 PHE P 545 18.426 15.660 -38.503 0.00 0.00 S

ATOM 546 OX1 PHE P 546 17.607 14.391 -37.978 0.00 0.00 O

ATOM 547 OX1 PHE P 547 18.590 11.923 -38.387 0.00 0.00 O

ATOM 548 SI1 PHE P 548 20.799 10.214 -40.516 0.00 0.00 S

ATOM 549 SI1 PHE P 549 19.257 11.725 -36.944 0.00 0.00 S

ATOM 550 SI1 PHE P 550 21.946 12.891 -39.999 0.00 0.00 S

ATOM 551 OX1 PHE P 551 20.830 11.817 -40.370 0.00 0.00 O

ATOM 552 OX1 PHE P 552 21.268 9.394 -39.253 0.00 0.00 O

ATOM 553 OX1 PHE P 553 22.105 8.768 -36.976 0.00 0.00 O

ATOM 554 OX1 PHE P 554 20.436 10.654 -36.817 0.00 0.00 O

ATOM 555 OX1 PHE P 555 19.968 13.121 -36.702 0.00 0.00 O

ATOM 556 OX1 PHE P 556 22.169 12.987 -38.414 0.00 0.00 O

ATOM 557 SI1 PHE P 557 24.441 7.739 -42.117 0.00 0.00 S

ATOM 558 SI1 PHE P 558 22.616 9.111 -38.456 0.00 0.00 S

ATOM 559 SI1 PHE P 559 25.593 10.186 -41.156 0.00 0.00 S

ATOM 560 OX1 PHE P 560 24.391 9.222 -41.534 0.00 0.00 O

ATOM 561 OX1 PHE P 561 25.722 6.878 -41.648 0.00 0.00 O

ATOM 562 OX1 PHE P 562 25.384 6.114 -39.166 0.00 0.00 O

ATOM 563 OX1 PHE P 563 23.423 7.883 -39.078 0.00 0.00 O

ATOM 564 OX1 PHE P 564 23.439 10.470 -38.493 0.00 0.00 O

ATOM 565 OX1 PHE P 565 25.809 10.151 -39.562 0.00 0.00 O

ATOM 566 SI1 PHE P 566 27.982 4.954 -43.374 0.00 0.00 S

ATOM 567 SI1 PHE P 567 26.443 6.543 -40.246 0.00 0.00 S

ATOM 568 SI1 PHE P 568 29.070 7.586 -42.864 0.00 0.00 S

ATOM 569 OX1 PHE P 569 28.092 6.528 -43.534 0.00 0.00 O

ATOM 570 OX1 PHE P 570 29.277 4.050 -43.078 0.00 0.00 O

ATOM 571 OX1 PHE P 571 29.204 3.443 -40.539 0.00 0.00 O

ATOM 572 OX1 PHE P 572 27.347 5.257 -40.534 0.00 0.00 O

ATOM 573 OX1 PHE P 573 27.366 7.718 -39.687 0.00 0.00 O

ATOM 574 OX1 PHE P 574 29.493 7.462 -41.322 0.00 0.00 O

ATOM 575 SI1 PHE P 575 30.159 3.922 -41.734 0.00 0.00 S

ATOM 576 OX1 PHE P 576 32.995 0.995 -42.046 0.00 0.00 O

ATOM 577 OX1 PHE P 577 31.267 2.809 -41.998 0.00 0.00 O

ATOM 578 OX1 PHE P 578 31.024 5.219 -41.365 0.00 0.00 O

ATOM 579 OX1 PHE P 579 32.083 6.017 -43.613 0.00 0.00 O

ATOM 580 SI1 PHE P 580 33.531 0.858 -43.564 0.00 0.00 S

ATOM 581 OX1 PHE P 581 34.489 -0.405 -43.795 0.00 0.00 O

ATOM 582 OX1 PHE P 582 34.241 2.139 -44.194 0.00 0.00 O

ATOM 583 SI1 PHE P 583 21.434 13.595 -37.122 0.00 0.00 S

ATOM 584 SI1 PHE P 584 24.985 10.822 -38.358 0.00 0.00 S

ATOM 585 SI1 PHE P 585 26.349 13.346 -38.622 0.00 0.00 S

ATOM 586 OX1 PHE P 586 25.061 12.419 -38.472 0.00 0.00 O

ATOM 587 OX1 PHE P 587 25.580 10.352 -36.965 0.00 0.00 O

ATOM 588 SI1 PHE P 588 28.910 8.061 -39.952 0.00 0.00 S

ATOM 589 SI1 PHE P 589 29.952 10.772 -40.414 0.00 0.00 S

ATOM 590 OX1 PHE P 590 28.882 9.667 -39.987 0.00 0.00 O

ATOM 591 OX1 PHE P 591 29.716 7.441 -38.706 0.00 0.00 O

ATOM 592 OX1 PHE P 592 30.693 11.375 -39.145 0.00 0.00 O

ATOM 593 SI1 PHE P 593 32.353 5.811 -42.062 0.00 0.00 S

ATOM 594 SI1 PHE P 594 31.260 7.332 -38.294 0.00 0.00 S

ATOM 595 SI1 PHE P 595 33.640 8.416 -41.701 0.00 0.00 S

ATOM 596 OX1 PHE P 596 32.631 7.222 -41.370 0.00 0.00 O

ATOM 597 OX1 PHE P 597 33.617 4.832 -41.857 0.00 0.00 O

ATOM 598 OX1 PHE P 598 33.419 3.896 -39.421 0.00 0.00 O

ATOM 599 OX1 PHE P 599 32.093 6.121 -38.929 0.00 0.00 O

ATOM 600 OX1 PHE P 600 31.996 8.654 -38.770 0.00 0.00 O

ATOM 601 OX1 PHE P 601 33.980 9.201 -40.348 0.00 0.00 O

ATOM 602 SI1 PHE P 602 35.659 2.745 -43.752 0.00 0.00 S

ATOM 603 SI1 PHE P 603 34.422 4.384 -40.531 0.00 0.00 S

ATOM 604 SI1 PHE P 604 36.796 5.380 -43.363 0.00 0.00 S

ATOM 605 OX1 PHE P 605 35.745 4.320 -43.909 0.00 0.00 O

ATOM 606 OX1 PHE P 606 36.973 1.840 -43.555 0.00 0.00 O

ATOM 607 OX1 PHE P 607 37.059 1.204 -40.995 0.00 0.00 O

ATOM 608 OX1 PHE P 608 35.279 3.078 -40.878 0.00 0.00 O

ATOM 609 OX1 PHE P 609 35.363 5.544 -39.964 0.00 0.00 O

ATOM 610 OX1 PHE P 610 37.300 5.387 -41.839 0.00 0.00 O

ATOM 611 SI1 PHE P 611 37.922 1.646 -42.265 0.00 0.00 S

ATOM 612 OX1 PHE P 612 40.914 -1.240 -42.264 0.00 0.00 O

ATOM 613 OX1 PHE P 613 38.899 0.423 -42.618 0.00 0.00 O

ATOM 614 OX1 PHE P 614 38.763 2.959 -41.877 0.00 0.00 O

ATOM 615 OX1 PHE P 615 39.889 3.654 -44.138 0.00 0.00 O

ATOM 616 SI1 PHE P 616 41.414 -1.213 -43.781 0.00 0.00 S

ATOM 617 OX1 PHE P 617 42.477 -2.307 -44.212 0.00 0.00 O

ATOM 618 OX1 PHE P 618 41.969 0.176 -44.303 0.00 0.00 O

ATOM 619 SI1 PHE P 619 33.573 8.868 -38.842 0.00 0.00 S

ATOM 620 SI1 PHE P 620 36.787 6.070 -40.477 0.00 0.00 S

ATOM 621 SI1 PHE P 621 37.872 8.698 -41.036 0.00 0.00 S

ATOM 622 OX1 PHE P 622 36.732 7.668 -40.595 0.00 0.00 O

ATOM 623 OX1 PHE P 623 37.855 5.693 -39.365 0.00 0.00 O

ATOM 624 SI1 PHE P 624 40.071 3.592 -42.562 0.00 0.00 S

ATOM 625 SI1 PHE P 625 41.429 6.166 -42.292 0.00 0.00 S

ATOM 626 OX1 PHE P 626 40.316 5.057 -41.969 0.00 0.00 O

ATOM 627 OX1 PHE P 627 41.322 2.688 -42.167 0.00 0.00 O

ATOM 628 SI1 PHE P 628 43.389 0.732 -43.835 0.00 0.00 S

ATOM 629 SI1 PHE P 629 41.969 2.635 -40.707 0.00 0.00 S

ATOM 630 SI1 PHE P 630 44.726 3.245 -43.928 0.00 0.00 S

ATOM 631 OX1 PHE P 631 43.694 2.159 -44.446 0.00 0.00 O

ATOM 632 OX1 PHE P 632 44.578 -0.324 -43.658 0.00 0.00 O

ATOM 633 OX1 PHE P 633 42.989 1.459 -40.419 0.00 0.00 O

ATOM 634 OX1 PHE P 634 45.145 3.268 -42.399 0.00 0.00 O

ATOM 635 SI1 PHE P 635 45.855 -0.177 -42.713 0.00 0.00 S

ATOM 636 OX1 PHE P 636 49.540 -4.160 -41.568 0.00 0.00 O

ATOM 637 OX1 PHE P 637 46.819 -1.429 -42.585 0.00 0.00 O

ATOM 638 OX1 PHE P 638 46.928 0.947 -43.022 0.00 0.00 O

ATOM 639 SI1 PHE P 639 49.814 -4.247 -43.130 0.00 0.00 S

ATOM 640 OX1 PHE P 640 50.639 -5.484 -43.697 0.00 0.00 O

ATOM 641 OX1 PHE P 641 49.874 -2.881 -43.943 0.00 0.00 O

ATOM 642 SI1 PHE P 642 51.132 -1.906 -43.920 0.00 0.00 S

ATOM 643 SI1 PHE P 643 -31.358 11.131 -45.993 0.00 0.00 S

ATOM 644 OX1 PHE P 644 -29.526 9.868 -43.296 0.00 0.00 O

ATOM 645 OX1 PHE P 645 -31.254 9.715 -45.259 0.00 0.00 O

ATOM 646 OX1 PHE P 646 -30.072 11.899 -46.536 0.00 0.00 O

ATOM 647 SI1 PHE P 647 -29.154 12.838 -45.643 0.00 0.00 S

ATOM 648 SI1 PHE P 648 -29.171 15.568 -44.949 0.00 0.00 S

ATOM 649 OX1 PHE P 649 -29.855 14.135 -45.042 0.00 0.00 O

ATOM 650 OX1 PHE P 650 -28.156 12.014 -44.724 0.00 0.00 O

ATOM 651 SI1 PHE P 651 -27.314 12.447 -43.448 0.00 0.00 S

ATOM 652 OX1 PHE P 652 -24.271 10.602 -42.390 0.00 0.00 O

ATOM 653 OX1 PHE P 653 -26.747 11.029 -42.959 0.00 0.00 O

ATOM 654 OX1 PHE P 654 -26.309 13.660 -43.760 0.00 0.00 O

ATOM 655 OX1 PHE P 655 -25.714 13.378 -46.277 0.00 0.00 O

ATOM 656 SI1 PHE P 656 -23.636 9.887 -43.674 0.00 0.00 S

ATOM 657 OX1 PHE P 657 -21.125 7.477 -43.107 0.00 0.00 O

ATOM 658 OX1 PHE P 658 -23.562 8.297 -43.721 0.00 0.00 O

ATOM 659 OX1 PHE P 659 -22.407 10.693 -44.268 0.00 0.00 O

ATOM 660 SI1 PHE P 660 -25.161 13.824 -44.859 0.00 0.00 S

ATOM 661 SI1 PHE P 661 -23.917 16.222 -43.881 0.00 0.00 S

ATOM 662 OX1 PHE P 662 -24.661 15.337 -44.982 0.00 0.00 O

ATOM 663 OX1 PHE P 663 -23.911 12.938 -44.491 0.00 0.00 O

ATOM 664 SI1 PHE P 664 -21.231 11.291 -43.384 0.00 0.00 S

ATOM 665 SI1 PHE P 665 -20.453 13.685 -42.242 0.00 0.00 S

ATOM 666 OX1 PHE P 666 -21.515 12.525 -42.426 0.00 0.00 O

ATOM 667 OX1 PHE P 667 -20.026 10.330 -43.052 0.00 0.00 O

ATOM 668 OX1 PHE P 668 -20.442 9.385 -40.566 0.00 0.00 O

ATOM 669 OX1 PHE P 669 -22.429 11.109 -40.215 0.00 0.00 O

ATOM 670 OX1 PHE P 670 -22.154 13.676 -39.050 0.00 0.00 O

ATOM 671 OX1 PHE P 671 -20.694 14.741 -41.082 0.00 0.00 O

ATOM 672 SI1 PHE P 672 -19.415 9.941 -41.641 0.00 0.00 S

ATOM 673 OX1 PHE P 673 -16.395 7.765 -41.646 0.00 0.00 O

ATOM 674 OX1 PHE P 674 -18.585 8.660 -42.033 0.00 0.00 O

ATOM 675 OX1 PHE P 675 -18.535 11.112 -41.003 0.00 0.00 O

ATOM 676 OX1 PHE P 676 -17.317 11.934 -43.149 0.00 0.00 O

ATOM 677 SI1 PHE P 677 -15.882 7.546 -43.162 0.00 0.00 S

ATOM 678 OX1 PHE P 678 -13.255 5.015 -43.074 0.00 0.00 O

ATOM 679 OX1 PHE P 679 -15.670 6.004 -43.538 0.00 0.00 O

ATOM 680 OX1 PHE P 680 -14.924 8.642 -43.834 0.00 0.00 O

ATOM 681 SI1 PHE P 681 -20.872 14.476 -39.533 0.00 0.00 S

ATOM 682 OX1 PHE P 682 -19.709 13.702 -38.779 0.00 0.00 O

ATOM 683 SI1 PHE P 683 -17.191 11.760 -41.574 0.00 0.00 S

ATOM 684 SI1 PHE P 684 -16.577 14.638 -41.303 0.00 0.00 S

ATOM 685 OX1 PHE P 685 -17.106 13.188 -40.895 0.00 0.00 O

ATOM 686 OX1 PHE P 686 -15.934 10.834 -41.212 0.00 0.00 O

ATOM 687 OX1 PHE P 687 -16.345 10.126 -38.728 0.00 0.00 O

ATOM 688 OX1 PHE P 688 -16.674 15.280 -39.860 0.00 0.00 O

ATOM 689 SI1 PHE P 689 -13.472 9.140 -43.378 0.00 0.00 S

ATOM 690 SI1 PHE P 690 -15.246 10.406 -39.819 0.00 0.00 S

ATOM 691 SI1 PHE P 691 -13.257 11.911 -42.552 0.00 0.00 S

ATOM 692 OX1 PHE P 692 -13.345 10.344 -42.365 0.00 0.00 O

ATOM 693 OX1 PHE P 693 -12.320 8.075 -43.156 0.00 0.00 O

ATOM 694 OX1 PHE P 694 -12.725 7.217 -40.657 0.00 0.00 O

ATOM 695 OX1 PHE P 695 -14.420 9.100 -40.244 0.00 0.00 O

ATOM 696 OX1 PHE P 696 -14.348 11.547 -39.153 0.00 0.00 O

ATOM 697 OX1 PHE P 697 -13.099 12.666 -41.173 0.00 0.00 O

ATOM 698 SI1 PHE P 698 -11.662 7.689 -41.752 0.00 0.00 S

ATOM 699 OX1 PHE P 699 -8.710 5.339 -41.419 0.00 0.00 O

ATOM 700 OX1 PHE P 700 -10.882 6.339 -42.057 0.00 0.00 O

ATOM 701 OX1 PHE P 701 -10.741 8.857 -41.138 0.00 0.00 O

ATOM 702 OX1 PHE P 702 -9.410 9.778 -43.176 0.00 0.00 O

ATOM 703 SI1 PHE P 703 -8.274 4.995 -42.915 0.00 0.00 S

ATOM 704 OX1 PHE P 704 -5.675 2.967 -43.823 0.00 0.00 O

ATOM 705 OX1 PHE P 705 -8.139 3.442 -43.203 0.00 0.00 O

ATOM 706 OX1 PHE P 706 -7.417 6.059 -43.737 0.00 0.00 O

ATOM 707 SI1 PHE P 707 -12.991 12.212 -39.655 0.00 0.00 S

ATOM 708 SI1 PHE P 708 -11.871 14.771 -39.462 0.00 0.00 S

ATOM 709 OX1 PHE P 709 -12.789 13.597 -38.894 0.00 0.00 O

ATOM 710 OX1 PHE P 710 -11.710 11.270 -39.435 0.00 0.00 O

ATOM 711 OX1 PHE P 711 -11.180 15.367 -38.180 0.00 0.00 O

ATOM 712 SI1 PHE P 712 -9.357 9.527 -41.608 0.00 0.00 S

ATOM 713 SI1 PHE P 713 -10.943 10.831 -38.090 0.00 0.00 S

ATOM 714 SI1 PHE P 714 -8.372 12.245 -40.995 0.00 0.00 S

ATOM 715 OX1 PHE P 715 -9.258 10.924 -40.848 0.00 0.00 O

ATOM 716 OX1 PHE P 716 -8.092 8.612 -41.233 0.00 0.00 O

ATOM 717 OX1 PHE P 717 -8.367 7.659 -38.746 0.00 0.00 O

ATOM 718 OX1 PHE P 718 -10.166 9.491 -38.513 0.00 0.00 O

ATOM 719 OX1 PHE P 719 -9.958 11.966 -37.519 0.00 0.00 O

ATOM 720 OX1 PHE P 720 -8.538 12.962 -39.576 0.00 0.00 O

ATOM 721 SI1 PHE P 721 -6.089 6.807 -43.268 0.00 0.00 S

ATOM 722 SI1 PHE P 722 -7.368 8.191 -39.853 0.00 0.00 S

ATOM 723 SI1 PHE P 723 -5.151 9.446 -42.721 0.00 0.00 S

ATOM 724 OX1 PHE P 724 -6.171 8.394 -43.326 0.00 0.00 O

ATOM 725 OX1 PHE P 725 -4.723 5.998 -43.074 0.00 0.00 O

ATOM 726 OX1 PHE P 726 -4.755 5.151 -40.544 0.00 0.00 O

ATOM 727 OX1 PHE P 727 -6.458 6.960 -40.261 0.00 0.00 O

ATOM 728 OX1 PHE P 728 -6.415 9.315 -39.254 0.00 0.00 O

ATOM 729 OX1 PHE P 729 -4.599 9.332 -41.245 0.00 0.00 O

ATOM 730 SI1 PHE P 730 -3.866 5.666 -41.760 0.00 0.00 S

ATOM 731 OX1 PHE P 731 -0.899 3.193 -41.702 0.00 0.00 O

ATOM 732 OX1 PHE P 732 -3.070 4.328 -42.144 0.00 0.00 O

ATOM 733 OX1 PHE P 733 -2.930 6.855 -41.215 0.00 0.00 O

ATOM 734 OX1 PHE P 734 -1.777 7.766 -43.349 0.00 0.00 O

ATOM 735 SI1 PHE P 735 -0.530 2.705 -43.183 0.00 0.00 S

ATOM 736 OX1 PHE P 736 2.077 0.709 -43.987 0.00 0.00 O

ATOM 737 OX1 PHE P 737 -0.393 1.130 -43.301 0.00 0.00 O

ATOM 738 OX1 PHE P 738 0.518 3.536 -44.062 0.00 0.00 O

ATOM 739 SI1 PHE P 739 -8.561 12.576 -38.027 0.00 0.00 S

ATOM 740 SI1 PHE P 740 -7.596 15.315 -37.832 0.00 0.00 S

ATOM 741 OX1 PHE P 741 -8.406 14.021 -37.295 0.00 0.00 O

ATOM 742 OX1 PHE P 742 -7.328 11.575 -37.755 0.00 0.00 O

ATOM 743 SI1 PHE P 743 -4.997 9.834 -39.782 0.00 0.00 S

ATOM 744 SI1 PHE P 744 -6.329 11.576 -36.502 0.00 0.00 S

ATOM 745 SI1 PHE P 745 -4.048 12.621 -39.707 0.00 0.00 S

ATOM 746 OX1 PHE P 746 -5.092 11.427 -39.551 0.00 0.00 O

ATOM 747 OX1 PHE P 747 -3.729 9.042 -39.226 0.00 0.00 O

ATOM 748 OX1 PHE P 748 -3.597 8.420 -36.627 0.00 0.00 O

ATOM 749 OX1 PHE P 749 -5.132 10.521 -36.554 0.00 0.00 O

ATOM 750 OX1 PHE P 750 -5.623 12.989 -36.588 0.00 0.00 O

ATOM 751 OX1 PHE P 751 -3.416 12.873 -38.253 0.00 0.00 O

ATOM 752 SI1 PHE P 752 -1.579 7.496 -41.797 0.00 0.00 S

ATOM 753 SI1 PHE P 753 -2.815 8.805 -37.949 0.00 0.00 S

ATOM 754 SI1 PHE P 754 -0.601 10.278 -41.287 0.00 0.00 S

ATOM 755 OX1 PHE P 755 -1.358 8.884 -41.040 0.00 0.00 O

ATOM 756 OX1 PHE P 756 -0.353 6.492 -41.557 0.00 0.00 O

ATOM 757 OX1 PHE P 757 -0.391 5.623 -39.012 0.00 0.00 O

ATOM 758 OX1 PHE P 758 -1.934 7.592 -38.483 0.00 0.00 O

ATOM 759 OX1 PHE P 759 -1.894 10.049 -37.593 0.00 0.00 O

ATOM 760 OX1 PHE P 760 -0.806 11.012 -39.878 0.00 0.00 O

ATOM 761 SI1 PHE P 761 1.814 4.359 -43.612 0.00 0.00 S

ATOM 762 SI1 PHE P 762 0.489 6.053 -40.257 0.00 0.00 S

ATOM 763 SI1 PHE P 763 2.307 7.220 -43.261 0.00 0.00 S

ATOM 764 OX1 PHE P 764 1.464 5.894 -43.443 0.00 0.00 O

ATOM 765 OX1 PHE P 765 3.167 3.632 -43.141 0.00 0.00 O

ATOM 766 OX1 PHE P 766 3.283 3.125 -40.438 0.00 0.00 O

ATOM 767 OX1 PHE P 767 1.295 4.729 -40.632 0.00 0.00 O

ATOM 768 OX1 PHE P 768 1.522 7.166 -39.806 0.00 0.00 O

ATOM 769 OX1 PHE P 769 3.198 7.303 -41.949 0.00 0.00 O

ATOM 770 SI1 PHE P 770 4.051 3.490 -41.801 0.00 0.00 S

ATOM 771 OX1 PHE P 771 7.126 1.070 -41.805 0.00 0.00 O

ATOM 772 OX1 PHE P 772 4.874 2.158 -42.171 0.00 0.00 O

ATOM 773 OX1 PHE P 773 4.995 4.736 -41.437 0.00 0.00 O

ATOM 774 OX1 PHE P 774 6.218 5.447 -43.615 0.00 0.00 O

ATOM 775 SI1 PHE P 775 7.508 0.511 -43.255 0.00 0.00 S

ATOM 776 OX1 PHE P 776 10.183 -1.539 -43.770 0.00 0.00 O

ATOM 777 OX1 PHE P 777 7.666 -1.066 -43.274 0.00 0.00 O

ATOM 778 OX1 PHE P 778 8.600 1.291 -44.122 0.00 0.00 O

ATOM 779 SI1 PHE P 779 -4.113 13.398 -36.889 0.00 0.00 S

ATOM 780 SI1 PHE P 780 -0.609 10.612 -38.353 0.00 0.00 S

ATOM 781 SI1 PHE P 781 0.700 13.102 -38.163 0.00 0.00 S

ATOM 782 OX1 PHE P 782 -0.242 11.961 -37.578 0.00 0.00 O

ATOM 783 OX1 PHE P 783 0.589 9.563 -38.313 0.00 0.00 O

ATOM 784 OX1 PHE P 784 0.615 9.234 -35.759 0.00 0.00 O

ATOM 785 OX1 PHE P 785 1.590 13.655 -36.974 0.00 0.00 O

ATOM 786 SI1 PHE P 786 2.909 7.669 -40.419 0.00 0.00 S

ATOM 787 SI1 PHE P 787 1.515 9.267 -37.061 0.00 0.00 S

ATOM 788 SI1 PHE P 788 3.947 10.341 -40.260 0.00 0.00 S

ATOM 789 OX1 PHE P 789 2.810 9.243 -40.158 0.00 0.00 O

ATOM 790 OX1 PHE P 790 4.207 6.932 -39.848 0.00 0.00 O

ATOM 791 OX1 PHE P 791 4.151 6.197 -37.313 0.00 0.00 O

ATOM 792 OX1 PHE P 792 2.233 7.872 -37.331 0.00 0.00 O

ATOM 793 OX1 PHE P 793 2.492 10.512 -36.922 0.00 0.00 O

ATOM 794 OX1 PHE P 794 4.574 10.407 -38.801 0.00 0.00 O

ATOM 795 SI1 PHE P 795 6.382 5.262 -42.047 0.00 0.00 S

ATOM 796 SI1 PHE P 796 5.032 6.714 -38.501 0.00 0.00 S

ATOM 797 SI1 PHE P 797 7.516 8.025 -41.557 0.00 0.00 S

ATOM 798 OX1 PHE P 798 6.670 6.674 -41.342 0.00 0.00 O

ATOM 799 OX1 PHE P 799 7.530 4.190 -41.714 0.00 0.00 O

ATOM 800 OX1 PHE P 800 7.656 3.568 -39.017 0.00 0.00 O

ATOM 801 OX1 PHE P 801 5.969 5.423 -38.910 0.00 0.00 O

ATOM 802 OX1 PHE P 802 5.872 7.977 -38.037 0.00 0.00 O

ATOM 803 OX1 PHE P 803 7.350 8.799 -40.154 0.00 0.00 O

ATOM 804 SI1 PHE P 804 9.904 2.066 -43.622 0.00 0.00 S

ATOM 805 SI1 PHE P 805 8.396 3.914 -40.384 0.00 0.00 S

ATOM 806 SI1 PHE P 806 10.528 4.885 -43.290 0.00 0.00 S

ATOM 807 OX1 PHE P 807 9.615 3.606 -43.435 0.00 0.00 O

ATOM 808 OX1 PHE P 808 11.188 1.293 -43.062 0.00 0.00 O

ATOM 809 OX1 PHE P 809 11.312 1.014 -40.335 0.00 0.00 O

ATOM 810 OX1 PHE P 810 9.293 2.610 -40.656 0.00 0.00 O

ATOM 811 OX1 PHE P 811 9.263 5.225 -40.185 0.00 0.00 O

ATOM 812 OX1 PHE P 812 11.326 5.016 -41.917 0.00 0.00 O

ATOM 813 SI1 PHE P 813 12.071 1.289 -41.719 0.00 0.00 S

ATOM 814 OX1 PHE P 814 15.132 -0.993 -41.661 0.00 0.00 O

ATOM 815 OX1 PHE P 815 12.852 -0.080 -41.993 0.00 0.00 O

ATOM 816 OX1 PHE P 816 12.998 2.592 -41.534 0.00 0.00 O

ATOM 817 OX1 PHE P 817 14.341 2.593 -43.751 0.00 0.00 O

ATOM 818 SI1 PHE P 818 15.582 -1.427 -43.143 0.00 0.00 S

ATOM 819 OX1 PHE P 819 18.364 -3.755 -42.983 0.00 0.00 O

ATOM 820 OX1 PHE P 820 15.843 -2.995 -43.278 0.00 0.00 O

ATOM 821 OX1 PHE P 821 16.609 -0.441 -43.861 0.00 0.00 O

ATOM 822 SI1 PHE P 822 3.968 10.886 -37.399 0.00 0.00 S

ATOM 823 SI1 PHE P 823 4.965 13.378 -36.585 0.00 0.00 S

ATOM 824 OX1 PHE P 824 3.949 12.479 -37.393 0.00 0.00 O

ATOM 825 OX1 PHE P 825 4.944 10.266 -36.308 0.00 0.00 O

ATOM 826 SI1 PHE P 826 7.291 8.442 -38.587 0.00 0.00 S

ATOM 827 SI1 PHE P 827 8.717 10.867 -38.310 0.00 0.00 S

ATOM 828 OX1 PHE P 828 7.666 9.782 -37.797 0.00 0.00 O

ATOM 829 OX1 PHE P 829 8.269 7.256 -38.184 0.00 0.00 O

ATOM 830 OX1 PHE P 830 9.228 7.862 -35.766 0.00 0.00 O

ATOM 831 OX1 PHE P 831 9.536 11.415 -37.067 0.00 0.00 O

ATOM 832 SI1 PHE P 832 10.802 5.481 -40.479 0.00 0.00 S

ATOM 833 SI1 PHE P 833 9.519 7.258 -37.206 0.00 0.00 S

ATOM 834 SI1 PHE P 834 12.124 8.087 -40.425 0.00 0.00 S

ATOM 835 OX1 PHE P 835 10.911 7.063 -40.308 0.00 0.00 O

ATOM 836 OX1 PHE P 836 11.516 4.537 -39.434 0.00 0.00 O

ATOM 837 OX1 PHE P 837 12.472 5.203 -37.182 0.00 0.00 O

ATOM 838 OX1 PHE P 838 9.901 5.721 -37.115 0.00 0.00 O

ATOM 839 OX1 PHE P 839 10.555 8.197 -37.914 0.00 0.00 O

ATOM 840 OX1 PHE P 840 12.793 8.265 -38.986 0.00 0.00 O

ATOM 841 SI1 PHE P 841 14.406 2.964 -42.209 0.00 0.00 S

ATOM 842 SI1 PHE P 842 12.851 4.561 -38.599 0.00 0.00 S

ATOM 843 SI1 PHE P 843 15.835 5.608 -42.002 0.00 0.00 S

ATOM 844 OX1 PHE P 844 14.620 4.559 -42.163 0.00 0.00 O

ATOM 845 OX1 PHE P 845 15.558 2.039 -41.598 0.00 0.00 O

ATOM 846 OX1 PHE P 846 15.493 1.618 -38.895 0.00 0.00 O

ATOM 847 OX1 PHE P 847 13.190 3.003 -38.467 0.00 0.00 O

ATOM 848 OX1 PHE P 848 13.928 5.371 -39.450 0.00 0.00 O

ATOM 849 OX1 PHE P 849 16.208 5.683 -40.443 0.00 0.00 O

ATOM 850 SI1 PHE P 850 17.935 0.207 -43.252 0.00 0.00 S

ATOM 851 SI1 PHE P 851 16.340 1.922 -40.208 0.00 0.00 S

ATOM 852 SI1 PHE P 852 19.194 2.721 -43.008 0.00 0.00 S

ATOM 853 OX1 PHE P 853 18.112 1.738 -43.609 0.00 0.00 O

ATOM 854 OX1 PHE P 854 19.179 -0.688 -42.798 0.00 0.00 O

ATOM 855 OX1 PHE P 855 19.180 -1.067 -40.135 0.00 0.00 O

ATOM 856 OX1 PHE P 856 17.200 0.579 -40.400 0.00 0.00 O

ATOM 857 OX1 PHE P 857 17.236 3.225 -40.108 0.00 0.00 O

ATOM 858 OX1 PHE P 858 19.498 2.691 -41.443 0.00 0.00 O

ATOM 859 SI1 PHE P 859 20.050 -0.760 -41.448 0.00 0.00 S

ATOM 860 OX1 PHE P 860 23.134 -3.049 -41.257 0.00 0.00 O

ATOM 861 OX1 PHE P 861 20.858 -2.104 -41.752 0.00 0.00 O

ATOM 862 OX1 PHE P 862 21.010 0.485 -41.102 0.00 0.00 O

ATOM 863 OX1 PHE P 863 22.349 0.955 -43.290 0.00 0.00 O

ATOM 864 SI1 PHE P 864 23.579 -3.507 -42.720 0.00 0.00 S

ATOM 865 OX1 PHE P 865 26.106 -5.761 -43.373 0.00 0.00 O

ATOM 866 OX1 PHE P 866 23.652 -5.087 -42.866 0.00 0.00 O

ATOM 867 OX1 PHE P 867 24.567 -2.594 -43.592 0.00 0.00 O

ATOM 868 SI1 PHE P 868 12.030 8.702 -37.662 0.00 0.00 S

ATOM 869 SI1 PHE P 869 13.004 11.202 -36.782 0.00 0.00 S

ATOM 870 OX1 PHE P 870 11.956 10.292 -37.547 0.00 0.00 O

ATOM 871 OX1 PHE P 871 12.728 8.058 -36.387 0.00 0.00 O

ATOM 872 SI1 PHE P 872 15.361 5.987 -39.129 0.00 0.00 S

ATOM 873 SI1 PHE P 873 16.494 8.558 -38.632 0.00 0.00 S

ATOM 874 OX1 PHE P 874 15.286 7.584 -38.993 0.00 0.00 O

ATOM 875 OX1 PHE P 875 15.986 5.173 -37.886 0.00 0.00 O

ATOM 876 OX1 PHE P 876 17.122 6.104 -35.707 0.00 0.00 O

ATOM 877 OX1 PHE P 877 16.749 8.445 -37.073 0.00 0.00 O

ATOM 878 SI1 PHE P 878 18.807 3.449 -40.226 0.00 0.00 S

ATOM 879 SI1 PHE P 879 17.343 5.253 -37.030 0.00 0.00 S

ATOM 880 SI1 PHE P 880 20.078 6.086 -40.454 0.00 0.00 S

ATOM 881 OX1 PHE P 881 18.888 5.035 -40.400 0.00 0.00 O

ATOM 882 OX1 PHE P 882 19.358 2.910 -38.844 0.00 0.00 O

ATOM 883 OX1 PHE P 883 20.368 3.147 -36.650 0.00 0.00 O

ATOM 884 OX1 PHE P 884 17.716 3.745 -36.669 0.00 0.00 O

ATOM 885 OX1 PHE P 885 18.475 5.878 -37.929 0.00 0.00 O

ATOM 886 OX1 PHE P 886 20.696 6.294 -38.986 0.00 0.00 O

ATOM 887 SI1 PHE P 887 22.407 1.002 -41.704 0.00 0.00 S

ATOM 888 SI1 PHE P 888 20.748 2.630 -38.120 0.00 0.00 S

ATOM 889 SI1 PHE P 889 23.685 3.658 -41.513 0.00 0.00 S

ATOM 890 OX1 PHE P 890 22.614 2.503 -41.220 0.00 0.00 O

ATOM 891 OX1 PHE P 891 23.626 0.113 -41.177 0.00 0.00 O

ATOM 892 OX1 PHE P 892 23.359 -0.435 -38.565 0.00 0.00 O

ATOM 893 OX1 PHE P 893 21.050 1.042 -38.220 0.00 0.00 O

ATOM 894 OX1 PHE P 894 21.874 3.518 -38.821 0.00 0.00 O

ATOM 895 OX1 PHE P 895 23.739 4.434 -40.104 0.00 0.00 O

ATOM 896 SI1 PHE P 896 25.955 -1.872 -43.234 0.00 0.00 S

ATOM 897 SI1 PHE P 897 24.361 -0.086 -39.759 0.00 0.00 S

ATOM 898 SI1 PHE P 898 26.879 0.728 -42.665 0.00 0.00 S

ATOM 899 OX1 PHE P 899 25.851 -0.461 -42.534 0.00 0.00 O

ATOM 900 OX1 PHE P 900 27.283 -2.703 -42.930 0.00 0.00 O

ATOM 901 OX1 PHE P 901 27.059 -3.233 -40.339 0.00 0.00 O

ATOM 902 OX1 PHE P 902 25.277 -1.363 -40.089 0.00 0.00 O

ATOM 903 OX1 PHE P 903 25.329 1.113 -39.359 0.00 0.00 O

ATOM 904 OX1 PHE P 904 27.097 1.624 -41.379 0.00 0.00 O

ATOM 905 SI1 PHE P 905 28.057 -2.899 -41.541 0.00 0.00 S

ATOM 906 OX1 PHE P 906 31.092 -5.098 -40.878 0.00 0.00 O

ATOM 907 OX1 PHE P 907 28.850 -4.273 -41.740 0.00 0.00 O

ATOM 908 OX1 PHE P 908 29.029 -1.698 -41.096 0.00 0.00 O

ATOM 909 OX1 PHE P 909 30.569 -1.438 -43.165 0.00 0.00 O

ATOM 910 SI1 PHE P 910 31.790 -5.649 -42.196 0.00 0.00 S

ATOM 911 OX1 PHE P 911 34.111 -8.055 -43.097 0.00 0.00 O

ATOM 912 OX1 PHE P 912 31.802 -7.211 -42.301 0.00 0.00 O

ATOM 913 OX1 PHE P 913 32.519 -4.775 -43.317 0.00 0.00 O

ATOM 914 SI1 PHE P 914 19.872 6.578 -37.638 0.00 0.00 S

ATOM 915 SI1 PHE P 915 20.611 9.095 -36.499 0.00 0.00 S

ATOM 916 OX1 PHE P 916 19.632 8.135 -37.326 0.00 0.00 O

ATOM 917 OX1 PHE P 917 20.655 5.911 -36.427 0.00 0.00 O

ATOM 918 SI1 PHE P 918 23.350 4.094 -38.564 0.00 0.00 S

ATOM 919 SI1 PHE P 919 24.261 6.792 -38.266 0.00 0.00 S

ATOM 920 OX1 PHE P 920 23.333 5.539 -37.832 0.00 0.00 O

ATOM 921 OX1 PHE P 921 24.387 3.105 -37.794 0.00 0.00 O

ATOM 922 OX1 PHE P 922 24.956 7.461 -37.003 0.00 0.00 O

ATOM 923 SI1 PHE P 923 26.819 1.462 -39.838 0.00 0.00 S

ATOM 924 SI1 PHE P 924 25.536 3.420 -36.681 0.00 0.00 S

ATOM 925 SI1 PHE P 925 28.060 4.133 -39.650 0.00 0.00 S

ATOM 926 OX1 PHE P 926 27.057 2.945 -39.272 0.00 0.00 O

ATOM 927 OX1 PHE P 927 27.837 0.327 -39.371 0.00 0.00 O

ATOM 928 OX1 PHE P 928 28.367 0.304 -36.723 0.00 0.00 O

ATOM 929 OX1 PHE P 929 26.769 2.395 -36.692 0.00 0.00 O

ATOM 930 OX1 PHE P 930 26.234 4.812 -37.005 0.00 0.00 O

ATOM 931 OX1 PHE P 931 28.606 4.657 -38.234 0.00 0.00 O

ATOM 932 SI1 PHE P 932 30.480 -1.211 -41.596 0.00 0.00 S

ATOM 933 SI1 PHE P 933 28.927 0.324 -38.215 0.00 0.00 S

ATOM 934 SI1 PHE P 934 31.660 1.437 -41.284 0.00 0.00 S

ATOM 935 OX1 PHE P 935 30.474 0.377 -41.392 0.00 0.00 O

ATOM 936 OX1 PHE P 936 31.737 -2.035 -40.983 0.00 0.00 O

ATOM 937 OX1 PHE P 937 31.658 -2.785 -38.441 0.00 0.00 O

ATOM 938 OX1 PHE P 938 29.824 -0.955 -38.534 0.00 0.00 O

ATOM 939 OX1 PHE P 939 29.700 1.681 -38.442 0.00 0.00 O

ATOM 940 OX1 PHE P 940 32.020 1.509 -39.712 0.00 0.00 O

ATOM 941 SI1 PHE P 941 33.900 -3.975 -43.253 0.00 0.00 S

ATOM 942 SI1 PHE P 942 32.590 -2.330 -39.632 0.00 0.00 S

ATOM 943 SI1 PHE P 943 34.820 -1.388 -42.594 0.00 0.00 S

ATOM 944 OX1 PHE P 944 33.813 -2.593 -42.499 0.00 0.00 O

ATOM 945 OX1 PHE P 945 35.264 -4.777 -43.049 0.00 0.00 O

ATOM 946 OX1 PHE P 946 35.034 -5.639 -40.577 0.00 0.00 O

ATOM 947 OX1 PHE P 947 33.477 -3.599 -40.085 0.00 0.00 O

ATOM 948 OX1 PHE P 948 33.501 -1.097 -39.152 0.00 0.00 O

ATOM 949 OX1 PHE P 949 34.939 -0.465 -41.309 0.00 0.00 O

ATOM 950 SI1 PHE P 950 36.046 -5.100 -41.690 0.00 0.00 S

ATOM 951 OX1 PHE P 951 39.058 -7.862 -41.664 0.00 0.00 O

ATOM 952 OX1 PHE P 952 37.030 -6.297 -42.074 0.00 0.00 O

ATOM 953 OX1 PHE P 953 36.907 -3.852 -41.145 0.00 0.00 O

ATOM 954 OX1 PHE P 954 37.822 -2.903 -43.374 0.00 0.00 O

ATOM 955 SI1 PHE P 955 39.513 -8.239 -43.149 0.00 0.00 S

ATOM 956 OX1 PHE P 956 40.656 -9.332 -43.231 0.00 0.00 O

ATOM 957 OX1 PHE P 957 39.957 -7.038 -44.092 0.00 0.00 O

ATOM 958 SI1 PHE P 958 27.773 5.244 -36.991 0.00 0.00 S

ATOM 959 SI1 PHE P 959 31.226 2.083 -38.429 0.00 0.00 S

ATOM 960 SI1 PHE P 960 32.426 4.638 -38.434 0.00 0.00 S

ATOM 961 OX1 PHE P 961 31.171 3.678 -38.461 0.00 0.00 O

ATOM 962 OX1 PHE P 962 31.939 1.612 -37.089 0.00 0.00 O

ATOM 963 SI1 PHE P 963 34.903 -0.569 -39.731 0.00 0.00 S

ATOM 964 SI1 PHE P 964 36.112 2.027 -39.999 0.00 0.00 S

ATOM 965 OX1 PHE P 965 35.172 0.950 -39.249 0.00 0.00 O

ATOM 966 OX1 PHE P 966 36.096 -1.519 -39.284 0.00 0.00 O

ATOM 967 OX1 PHE P 967 37.003 2.811 -38.945 0.00 0.00 O

ATOM 968 SI1 PHE P 968 38.187 -3.183 -41.855 0.00 0.00 S

ATOM 969 SI1 PHE P 969 36.614 -1.745 -37.797 0.00 0.00 S

ATOM 970 SI1 PHE P 970 39.579 -0.640 -41.625 0.00 0.00 S

ATOM 971 OX1 PHE P 971 38.565 -1.793 -41.158 0.00 0.00 O

ATOM 972 OX1 PHE P 972 39.426 -4.199 -41.781 0.00 0.00 O

ATOM 973 OX1 PHE P 973 39.671 -4.593 -39.166 0.00 0.00 O

ATOM 974 OX1 PHE P 974 37.743 -2.858 -37.791 0.00 0.00 O

ATOM 975 OX1 PHE P 975 37.091 -0.419 -37.068 0.00 0.00 O

ATOM 976 OX1 PHE P 976 40.003 0.063 -40.271 0.00 0.00 O

ATOM 977 SI1 PHE P 977 41.384 -6.323 -43.974 0.00 0.00 S

ATOM 978 SI1 PHE P 978 40.412 -4.539 -40.559 0.00 0.00 S

ATOM 979 SI1 PHE P 979 42.549 -3.785 -43.678 0.00 0.00 S

ATOM 980 OX1 PHE P 980 41.376 -4.746 -44.124 0.00 0.00 O

ATOM 981 OX1 PHE P 981 42.744 -7.178 -43.837 0.00 0.00 O

ATOM 982 OX1 PHE P 982 42.583 -8.015 -41.370 0.00 0.00 O

ATOM 983 OX1 PHE P 983 41.103 -5.926 -40.970 0.00 0.00 O

ATOM 984 OX1 PHE P 984 41.443 -3.346 -40.396 0.00 0.00 O

ATOM 985 OX1 PHE P 985 43.232 -3.996 -42.259 0.00 0.00 O

ATOM 986 SI1 PHE P 986 43.582 -7.556 -42.513 0.00 0.00 S

ATOM 987 OX1 PHE P 987 46.736 -10.188 -42.185 0.00 0.00 O

ATOM 988 OX1 PHE P 988 44.577 -8.778 -42.803 0.00 0.00 O

ATOM 989 OX1 PHE P 989 44.470 -6.368 -41.930 0.00 0.00 O

ATOM 990 OX1 PHE P 990 45.662 -5.320 -44.036 0.00 0.00 O

ATOM 991 SI1 PHE P 991 47.205 -10.445 -43.686 0.00 0.00 S

ATOM 992 OX1 PHE P 992 48.309 -11.568 -43.889 0.00 0.00 O

ATOM 993 OX1 PHE P 993 47.485 -9.122 -44.525 0.00 0.00 O

ATOM 994 SI1 PHE P 994 42.959 -3.301 -40.855 0.00 0.00 S

ATOM 995 SI1 PHE P 995 45.823 -5.780 -42.530 0.00 0.00 S

ATOM 996 OX1 PHE P 996 46.430 -4.643 -41.610 0.00 0.00 O

ATOM 997 SI1 PHE P 997 48.886 -8.359 -44.519 0.00 0.00 S

ATOM 998 SI1 PHE P 998 50.732 -6.895 -42.966 0.00 0.00 S

ATOM 999 OX1 PHE P 999 49.549 -7.949 -43.132 0.00 0.00 O

ATOM 1000 OX1 PHE P1000 49.804 -8.470 -45.807 0.00 0.00 O

ATOM 1001 SI1 PHE P1001 -32.603 5.546 -45.542 0.00 0.00 S

ATOM 1002 OX1 PHE P1002 -31.096 3.162 -43.511 0.00 0.00 O

ATOM 1003 OX1 PHE P1003 -32.759 3.974 -45.354 0.00 0.00 O

ATOM 1004 OX1 PHE P1004 -31.177 6.207 -45.325 0.00 0.00 O

ATOM 1005 SI1 PHE P1005 -30.676 6.651 -43.887 0.00 0.00 S

ATOM 1006 SI1 PHE P1006 -31.011 9.485 -43.697 0.00 0.00 S

ATOM 1007 OX1 PHE P1007 -31.300 7.978 -43.270 0.00 0.00 O

ATOM 1008 OX1 PHE P1008 -29.288 6.033 -43.428 0.00 0.00 O

ATOM 1009 SI1 PHE P1009 -28.674 5.875 -41.969 0.00 0.00 S

ATOM 1010 OX1 PHE P1010 -25.792 3.193 -42.628 0.00 0.00 O

ATOM 1011 OX1 PHE P1011 -27.486 4.849 -42.176 0.00 0.00 O

ATOM 1012 OX1 PHE P1012 -27.997 7.219 -41.416 0.00 0.00 O

ATOM 1013 OX1 PHE P1013 -26.579 7.939 -43.443 0.00 0.00 O

ATOM 1014 SI1 PHE P1014 -25.246 3.046 -44.118 0.00 0.00 S

ATOM 1015 OX1 PHE P1015 -22.674 0.417 -43.693 0.00 0.00 O

ATOM 1016 OX1 PHE P1016 -24.959 1.501 -44.434 0.00 0.00 O

ATOM 1017 OX1 PHE P1017 -24.255 4.187 -44.640 0.00 0.00 O

ATOM 1018 SI1 PHE P1018 -26.557 7.761 -41.863 0.00 0.00 S

ATOM 1019 SI1 PHE P1019 -25.751 10.573 -41.799 0.00 0.00 S

ATOM 1020 OX1 PHE P1020 -26.228 9.168 -41.175 0.00 0.00 O

ATOM 1021 OX1 PHE P1021 -25.491 6.614 -41.504 0.00 0.00 O

ATOM 1022 SI1 PHE P1022 -22.964 4.784 -43.909 0.00 0.00 S

ATOM 1023 SI1 PHE P1023 -24.977 5.888 -40.160 0.00 0.00 S

ATOM 1024 SI1 PHE P1024 -22.669 7.411 -42.751 0.00 0.00 S

ATOM 1025 OX1 PHE P1025 -23.173 5.908 -42.811 0.00 0.00 O

ATOM 1026 OX1 PHE P1026 -21.728 3.823 -43.640 0.00 0.00 O

ATOM 1027 OX1 PHE P1027 -22.407 2.795 -41.264 0.00 0.00 O

ATOM 1028 OX1 PHE P1028 -24.128 4.655 -40.733 0.00 0.00 O

ATOM 1029 OX1 PHE P1029 -24.049 6.865 -39.303 0.00 0.00 O

ATOM 1030 OX1 PHE P1030 -22.938 8.039 -41.310 0.00 0.00 O

ATOM 1031 SI1 PHE P1031 -21.225 3.296 -42.219 0.00 0.00 S

ATOM 1032 OX1 PHE P1032 -18.340 1.011 -41.620 0.00 0.00 O

ATOM 1033 OX1 PHE P1033 -20.486 1.913 -42.509 0.00 0.00 O

ATOM 1034 OX1 PHE P1034 -20.297 4.364 -41.451 0.00 0.00 O

ATOM 1035 OX1 PHE P1035 -18.733 5.259 -43.315 0.00 0.00 O

ATOM 1036 SI1 PHE P1036 -17.620 0.677 -43.006 0.00 0.00 S

ATOM 1037 OX1 PHE P1037 -15.545 -1.853 -44.277 0.00 0.00 O

ATOM 1038 OX1 PHE P1038 -17.768 -0.826 -43.428 0.00 0.00 O

ATOM 1039 OX1 PHE P1039 -16.747 1.675 -43.906 0.00 0.00 O

ATOM 1040 SI1 PHE P1040 -22.690 7.528 -39.818 0.00 0.00 S

ATOM 1041 SI1 PHE P1041 -21.491 10.021 -39.555 0.00 0.00 S

ATOM 1042 OX1 PHE P1042 -22.356 8.826 -38.951 0.00 0.00 O

ATOM 1043 OX1 PHE P1043 -21.432 6.538 -39.722 0.00 0.00 O

ATOM 1044 SI1 PHE P1044 -18.832 4.955 -41.759 0.00 0.00 S

ATOM 1045 SI1 PHE P1045 -20.685 6.037 -38.390 0.00 0.00 S

ATOM 1046 SI1 PHE P1046 -17.874 7.669 -41.032 0.00 0.00 S

ATOM 1047 OX1 PHE P1047 -18.688 6.298 -40.926 0.00 0.00 O

ATOM 1048 OX1 PHE P1048 -17.649 3.980 -41.290 0.00 0.00 O

ATOM 1049 OX1 PHE P1049 -18.045 2.887 -38.880 0.00 0.00 O

ATOM 1050 OX1 PHE P1050 -19.847 4.736 -38.790 0.00 0.00 O

ATOM 1051 OX1 PHE P1051 -19.742 7.150 -37.755 0.00 0.00 O

ATOM 1052 OX1 PHE P1052 -17.988 8.271 -39.545 0.00 0.00 O

ATOM 1053 SI1 PHE P1053 -15.330 2.370 -43.636 0.00 0.00 S

ATOM 1054 SI1 PHE P1054 -16.992 3.488 -39.895 0.00 0.00 S

ATOM 1055 SI1 PHE P1055 -14.778 5.031 -42.641 0.00 0.00 S

ATOM 1056 OX1 PHE P1056 -15.387 3.576 -42.623 0.00 0.00 O

ATOM 1057 OX1 PHE P1057 -14.025 1.463 -43.478 0.00 0.00 O

ATOM 1058 OX1 PHE P1058 -14.575 0.402 -41.144 0.00 0.00 O

ATOM 1059 OX1 PHE P1059 -16.063 2.325 -40.444 0.00 0.00 O

ATOM 1060 OX1 PHE P1060 -16.102 4.580 -39.144 0.00 0.00 O

ATOM 1061 OX1 PHE P1061 -14.954 5.657 -41.194 0.00 0.00 O

ATOM 1062 SI1 PHE P1062 -13.419 0.922 -42.099 0.00 0.00 S

ATOM 1063 OX1 PHE P1063 -10.511 -1.277 -41.430 0.00 0.00 O

ATOM 1064 OX1 PHE P1064 -12.680 -0.460 -42.368 0.00 0.00 O

ATOM 1065 OX1 PHE P1065 -12.474 1.981 -41.344 0.00 0.00 O

ATOM 1066 OX1 PHE P1066 -11.068 2.947 -43.280 0.00 0.00 O

ATOM 1067 SI1 PHE P1067 -9.787 -1.527 -42.829 0.00 0.00 S

ATOM 1068 OX1 PHE P1068 -7.536 -3.983 -44.118 0.00 0.00 O

ATOM 1069 OX1 PHE P1069 -9.809 -3.028 -43.277 0.00 0.00 O

ATOM 1070 OX1 PHE P1070 -8.910 -0.458 -43.635 0.00 0.00 O

ATOM 1071 SI1 PHE P1071 -18.257 7.629 -38.095 0.00 0.00 S

ATOM 1072 OX1 PHE P1072 -17.160 6.588 -37.646 0.00 0.00 O

ATOM 1073 SI1 PHE P1073 -14.768 5.278 -39.675 0.00 0.00 S

ATOM 1074 SI1 PHE P1074 -13.650 7.889 -39.540 0.00 0.00 S

ATOM 1075 OX1 PHE P1075 -14.595 6.717 -38.984 0.00 0.00 O

ATOM 1076 OX1 PHE P1076 -13.476 4.353 -39.511 0.00 0.00 O

ATOM 1077 OX1 PHE P1077 -13.807 3.499 -37.088 0.00 0.00 O

ATOM 1078 OX1 PHE P1078 -12.790 8.363 -38.313 0.00 0.00 O

ATOM 1079 SI1 PHE P1079 -11.047 2.618 -41.726 0.00 0.00 S

ATOM 1080 SI1 PHE P1080 -12.730 3.857 -38.188 0.00 0.00 S

ATOM 1081 SI1 PHE P1081 -10.232 5.370 -40.972 0.00 0.00 S

ATOM 1082 OX1 PHE P1082 -10.986 3.967 -40.878 0.00 0.00 O

ATOM 1083 OX1 PHE P1083 -9.777 1.685 -41.377 0.00 0.00 O

ATOM 1084 OX1 PHE P1084 -10.089 0.721 -38.929 0.00 0.00 O

ATOM 1085 OX1 PHE P1085 -11.901 2.579 -38.679 0.00 0.00 O

ATOM 1086 OX1 PHE P1086 -11.812 4.992 -37.531 0.00 0.00 O

ATOM 1087 OX1 PHE P1087 -10.470 6.076 -39.559 0.00 0.00 O

ATOM 1088 SI1 PHE P1088 -7.383 -0.037 -43.427 0.00 0.00 S

ATOM 1089 SI1 PHE P1089 -9.044 1.256 -39.998 0.00 0.00 S

ATOM 1090 SI1 PHE P1090 -6.822 2.698 -42.757 0.00 0.00 S

ATOM 1091 OX1 PHE P1091 -7.021 1.170 -42.492 0.00 0.00 O

ATOM 1092 OX1 PHE P1092 -6.253 -1.121 -43.224 0.00 0.00 O

ATOM 1093 OX1 PHE P1093 -6.464 -1.976 -40.698 0.00 0.00 O

ATOM 1094 OX1 PHE P1094 -8.047 0.050 -40.354 0.00 0.00 O

ATOM 1095 OX1 PHE P1095 -8.177 2.458 -39.411 0.00 0.00 O

ATOM 1096 OX1 PHE P1096 -6.374 3.279 -41.361 0.00 0.00 O

ATOM 1097 SI1 PHE P1097 -5.502 -1.445 -41.849 0.00 0.00 S

ATOM 1098 OX1 PHE P1098 -2.286 -3.406 -42.122 0.00 0.00 O

ATOM 1099 OX1 PHE P1099 -4.641 -2.690 -42.317 0.00 0.00 O

ATOM 1100 OX1 PHE P1100 -4.649 -0.229 -41.209 0.00 0.00 O

ATOM 1101 OX1 PHE P1101 -3.400 0.748 -43.233 0.00 0.00 O

ATOM 1102 SI1 PHE P1102 -1.769 -3.792 -43.580 0.00 0.00 S

ATOM 1103 OX1 PHE P1103 0.811 -6.376 -43.150 0.00 0.00 O

ATOM 1104 OX1 PHE P1104 -1.567 -5.374 -43.734 0.00 0.00 O

ATOM 1105 OX1 PHE P1105 -0.663 -2.780 -44.140 0.00 0.00 O

ATOM 1106 SI1 PHE P1106 -10.442 5.652 -38.023 0.00 0.00 S

ATOM 1107 SI1 PHE P1107 -9.392 8.334 -37.728 0.00 0.00 S

ATOM 1108 OX1 PHE P1108 -10.289 7.076 -37.258 0.00 0.00 O

ATOM 1109 OX1 PHE P1109 -9.214 4.639 -37.774 0.00 0.00 O

ATOM 1110 OX1 PHE P1110 -8.675 8.978 -36.467 0.00 0.00 O

ATOM 1111 SI1 PHE P1111 -6.737 3.032 -39.829 0.00 0.00 S

ATOM 1112 SI1 PHE P1112 -8.224 4.581 -36.514 0.00 0.00 S

ATOM 1113 SI1 PHE P1113 -5.788 5.738 -39.494 0.00 0.00 S

ATOM 1114 OX1 PHE P1114 -6.802 4.532 -39.275 0.00 0.00 O

ATOM 1115 OX1 PHE P1115 -5.496 2.120 -39.361 0.00 0.00 O

ATOM 1116 OX1 PHE P1116 -5.475 1.476 -36.743 0.00 0.00 O

ATOM 1117 OX1 PHE P1117 -7.074 3.473 -36.625 0.00 0.00 O

ATOM 1118 OX1 PHE P1118 -7.511 6.001 -36.582 0.00 0.00 O

ATOM 1119 OX1 PHE P1119 -5.195 5.994 -38.034 0.00 0.00 O

ATOM 1120 SI1 PHE P1120 -3.295 0.506 -41.667 0.00 0.00 S

ATOM 1121 SI1 PHE P1121 -4.642 1.907 -38.016 0.00 0.00 S

ATOM 1122 SI1 PHE P1122 -2.382 3.301 -41.122 0.00 0.00 S

ATOM 1123 OX1 PHE P1123 -3.188 1.923 -40.920 0.00 0.00 O

ATOM 1124 OX1 PHE P1124 -2.024 -0.420 -41.326 0.00 0.00 O

ATOM 1125 OX1 PHE P1125 -1.936 -1.138 -38.622 0.00 0.00 O

ATOM 1126 OX1 PHE P1126 -3.514 0.828 -38.319 0.00 0.00 O

ATOM 1127 OX1 PHE P1127 -3.818 3.194 -37.578 0.00 0.00 O

ATOM 1128 OX1 PHE P1128 -2.477 4.075 -39.723 0.00 0.00 O

ATOM 1129 SI1 PHE P1129 0.673 -2.332 -43.387 0.00 0.00 S

ATOM 1130 SI1 PHE P1130 -1.183 -0.744 -39.983 0.00 0.00 S

ATOM 1131 SI1 PHE P1131 0.980 0.484 -42.859 0.00 0.00 S

ATOM 1132 OX1 PHE P1132 0.846 -1.039 -42.499 0.00 0.00 O

ATOM 1133 OX1 PHE P1133 1.672 -3.434 -42.886 0.00 0.00 O

ATOM 1134 OX1 PHE P1134 1.586 -3.800 -40.155 0.00 0.00 O

ATOM 1135 OX1 PHE P1135 -0.342 -2.060 -40.351 0.00 0.00 O

ATOM 1136 OX1 PHE P1136 -0.241 0.466 -39.555 0.00 0.00 O

ATOM 1137 OX1 PHE P1137 1.452 1.252 -41.554 0.00 0.00 O

ATOM 1138 SI1 PHE P1138 2.444 -3.507 -41.492 0.00 0.00 S

ATOM 1139 OX1 PHE P1139 5.523 -5.649 -41.551 0.00 0.00 O

ATOM 1140 OX1 PHE P1140 3.206 -4.848 -41.869 0.00 0.00 O

ATOM 1141 OX1 PHE P1141 3.384 -2.264 -41.101 0.00 0.00 O

ATOM 1142 OX1 PHE P1142 4.614 -1.484 -43.238 0.00 0.00 O

ATOM 1143 SI1 PHE P1143 6.024 -5.954 -43.050 0.00 0.00 S

ATOM 1144 OX1 PHE P1144 8.704 -8.402 -43.131 0.00 0.00 O

ATOM 1145 OX1 PHE P1145 6.216 -7.526 -43.308 0.00 0.00 O

ATOM 1146 OX1 PHE P1146 7.114 -4.945 -43.665 0.00 0.00 O

ATOM 1147 SI1 PHE P1147 -5.989 6.461 -36.717 0.00 0.00 S

ATOM 1148 SI1 PHE P1148 -4.981 9.000 -36.112 0.00 0.00 S

ATOM 1149 OX1 PHE P1149 -6.092 8.049 -36.712 0.00 0.00 O

ATOM 1150 OX1 PHE P1150 -5.193 5.922 -35.450 0.00 0.00 O

ATOM 1151 SI1 PHE P1151 -2.443 3.767 -38.158 0.00 0.00 S

ATOM 1152 SI1 PHE P1152 -1.242 6.309 -37.851 0.00 0.00 S

ATOM 1153 OX1 PHE P1153 -2.319 5.200 -37.470 0.00 0.00 O

ATOM 1154 OX1 PHE P1154 -1.160 2.844 -37.900 0.00 0.00 O

ATOM 1155 OX1 PHE P1155 -1.280 2.278 -35.372 0.00 0.00 O

ATOM 1156 OX1 PHE P1156 -0.365 6.552 -36.547 0.00 0.00 O

ATOM 1157 SI1 PHE P1157 1.196 1.015 -40.004 0.00 0.00 S

ATOM 1158 SI1 PHE P1158 -0.312 2.474 -36.592 0.00 0.00 S

ATOM 1159 SI1 PHE P1159 2.047 3.736 -39.635 0.00 0.00 S

ATOM 1160 OX1 PHE P1160 1.190 2.448 -39.310 0.00 0.00 O

ATOM 1161 OX1 PHE P1161 2.467 0.135 -39.567 0.00 0.00 O

ATOM 1162 OX1 PHE P1162 2.268 -0.667 -37.078 0.00 0.00 O

ATOM 1163 OX1 PHE P1163 0.406 1.104 -36.979 0.00 0.00 O

ATOM 1164 OX1 PHE P1164 0.715 3.616 -36.128 0.00 0.00 O

ATOM 1165 OX1 PHE P1165 2.178 4.528 -38.241 0.00 0.00 O

ATOM 1166 SI1 PHE P1166 4.752 -1.645 -41.664 0.00 0.00 S

ATOM 1167 SI1 PHE P1167 3.261 -0.173 -38.202 0.00 0.00 S

ATOM 1168 SI1 PHE P1168 5.653 1.159 -41.196 0.00 0.00 S

ATOM 1169 OX1 PHE P1169 4.877 -0.218 -40.957 0.00 0.00 O

ATOM 1170 OX1 PHE P1170 6.023 -2.557 -41.284 0.00 0.00 O

ATOM 1171 OX1 PHE P1171 5.873 -3.317 -38.686 0.00 0.00 O

ATOM 1172 OX1 PHE P1172 4.198 -1.413 -38.545 0.00 0.00 O

ATOM 1173 OX1 PHE P1173 4.183 1.030 -37.692 0.00 0.00 O

ATOM 1174 OX1 PHE P1174 5.591 1.921 -39.801 0.00 0.00 O

ATOM 1175 SI1 PHE P1175 8.553 -4.507 -43.104 0.00 0.00 S

ATOM 1176 SI1 PHE P1176 6.799 -2.885 -39.903 0.00 0.00 S

ATOM 1177 SI1 PHE P1177 9.008 -1.689 -42.713 0.00 0.00 S

ATOM 1178 OX1 PHE P1178 8.820 -3.186 -42.287 0.00 0.00 O

ATOM 1179 OX1 PHE P1179 9.650 -5.555 -42.679 0.00 0.00 O

ATOM 1180 OX1 PHE P1180 9.511 -5.951 -39.979 0.00 0.00 O

ATOM 1181 OX1 PHE P1181 7.605 -4.237 -40.204 0.00 0.00 O

ATOM 1182 OX1 PHE P1182 7.756 -1.694 -39.409 0.00 0.00 O

ATOM 1183 OX1 PHE P1183 9.400 -0.863 -41.417 0.00 0.00 O

ATOM 1184 SI1 PHE P1184 10.421 -5.633 -41.278 0.00 0.00 S

ATOM 1185 OX1 PHE P1185 13.485 -7.841 -41.115 0.00 0.00 O

ATOM 1186 OX1 PHE P1186 11.211 -6.964 -41.620 0.00 0.00 O

ATOM 1187 OX1 PHE P1187 11.388 -4.409 -40.862 0.00 0.00 O

ATOM 1188 OX1 PHE P1188 12.822 -3.783 -42.925 0.00 0.00 O

ATOM 1189 SI1 PHE P1189 13.948 -8.184 -42.605 0.00 0.00 S

ATOM 1190 OX1 PHE P1190 16.530 -10.505 -43.182 0.00 0.00 O

ATOM 1191 OX1 PHE P1191 14.056 -9.758 -42.841 0.00 0.00 O

ATOM 1192 OX1 PHE P1192 14.955 -7.171 -43.341 0.00 0.00 O

ATOM 1193 SI1 PHE P1193 -0.896 6.647 -35.041 0.00 0.00 S

ATOM 1194 SI1 PHE P1194 2.118 4.186 -36.661 0.00 0.00 S

ATOM 1195 SI1 PHE P1195 3.064 6.887 -36.404 0.00 0.00 S

ATOM 1196 OX1 PHE P1196 2.226 5.614 -35.914 0.00 0.00 O

ATOM 1197 OX1 PHE P1197 3.374 3.274 -36.231 0.00 0.00 O

ATOM 1198 SI1 PHE P1198 5.575 1.622 -38.236 0.00 0.00 S

ATOM 1199 SI1 PHE P1199 4.314 3.391 -34.924 0.00 0.00 S

ATOM 1200 SI1 PHE P1200 6.625 4.289 -38.042 0.00 0.00 S

ATOM 1201 OX1 PHE P1201 5.595 3.127 -37.703 0.00 0.00 O

ATOM 1202 OX1 PHE P1202 6.867 0.757 -37.809 0.00 0.00 O

ATOM 1203 OX1 PHE P1203 7.032 0.255 -35.107 0.00 0.00 O

ATOM 1204 OX1 PHE P1204 5.536 2.367 -34.946 0.00 0.00 O

ATOM 1205 OX1 PHE P1205 5.026 4.818 -34.943 0.00 0.00 O

ATOM 1206 OX1 PHE P1206 7.228 4.664 -36.613 0.00 0.00 O

ATOM 1207 SI1 PHE P1207 9.186 -1.105 -39.861 0.00 0.00 S

ATOM 1208 SI1 PHE P1208 7.777 0.519 -36.488 0.00 0.00 S

ATOM 1209 SI1 PHE P1209 10.042 1.646 -39.616 0.00 0.00 S

ATOM 1210 OX1 PHE P1210 9.191 0.361 -39.225 0.00 0.00 O

ATOM 1211 OX1 PHE P1211 10.469 -1.977 -39.409 0.00 0.00 O

ATOM 1212 OX1 PHE P1212 10.275 -2.731 -36.892 0.00 0.00 O

ATOM 1213 OX1 PHE P1213 8.503 -0.856 -36.856 0.00 0.00 O

ATOM 1214 OX1 PHE P1214 8.826 1.703 -36.146 0.00 0.00 O

ATOM 1215 OX1 PHE P1215 10.241 2.507 -38.280 0.00 0.00 O

ATOM 1216 SI1 PHE P1216 12.827 -3.855 -41.339 0.00 0.00 S

ATOM 1217 SI1 PHE P1217 11.260 -2.292 -38.033 0.00 0.00 S

ATOM 1218 SI1 PHE P1218 13.681 -1.004 -40.996 0.00 0.00 S

ATOM 1219 OX1 PHE P1219 12.941 -2.393 -40.710 0.00 0.00 O

ATOM 1220 OX1 PHE P1220 14.072 -4.736 -40.806 0.00 0.00 O

ATOM 1221 OX1 PHE P1221 13.856 -5.488 -38.247 0.00 0.00 O

ATOM 1222 OX1 PHE P1222 12.142 -3.591 -38.308 0.00 0.00 O

ATOM 1223 OX1 PHE P1223 12.216 -1.120 -37.514 0.00 0.00 O

ATOM 1224 OX1 PHE P1224 13.653 -0.244 -39.592 0.00 0.00 O

ATOM 1225 SI1 PHE P1225 16.352 -6.551 -42.848 0.00 0.00 S

ATOM 1226 SI1 PHE P1226 14.828 -4.994 -39.388 0.00 0.00 S

ATOM 1227 SI1 PHE P1227 16.906 -3.741 -42.357 0.00 0.00 S

ATOM 1228 OX1 PHE P1228 16.392 -5.200 -42.028 0.00 0.00 O

ATOM 1229 OX1 PHE P1229 17.599 -7.479 -42.549 0.00 0.00 O

ATOM 1230 OX1 PHE P1230 17.489 -8.101 -39.899 0.00 0.00 O

ATOM 1231 OX1 PHE P1231 15.846 -6.186 -39.674 0.00 0.00 O

ATOM 1232 OX1 PHE P1232 15.680 -3.744 -38.882 0.00 0.00 O

ATOM 1233 OX1 PHE P1233 16.911 -2.870 -41.028 0.00 0.00 O

ATOM 1234 SI1 PHE P1234 18.383 -7.679 -41.168 0.00 0.00 S

ATOM 1235 OX1 PHE P1235 21.437 -9.864 -41.054 0.00 0.00 O

ATOM 1236 OX1 PHE P1236 19.177 -9.012 -41.506 0.00 0.00 O

ATOM 1237 OX1 PHE P1237 19.325 -6.455 -40.712 0.00 0.00 O

ATOM 1238 OX1 PHE P1238 20.639 -5.666 -42.808 0.00 0.00 O

ATOM 1239 SI1 PHE P1239 21.958 -10.200 -42.543 0.00 0.00 S

ATOM 1240 OX1 PHE P1240 24.599 -12.723 -42.381 0.00 0.00 O

ATOM 1241 OX1 PHE P1241 22.166 -11.767 -42.793 0.00 0.00 O

ATOM 1242 OX1 PHE P1242 22.958 -9.170 -43.259 0.00 0.00 O

ATOM 1243 SI1 PHE P1243 6.516 5.290 -35.313 0.00 0.00 S

ATOM 1244 OX1 PHE P1244 7.508 5.204 -34.080 0.00 0.00 O

ATOM 1245 SI1 PHE P1245 10.262 2.197 -36.708 0.00 0.00 S

ATOM 1246 SI1 PHE P1246 11.100 5.037 -36.346 0.00 0.00 S

ATOM 1247 OX1 PHE P1247 10.562 3.579 -35.954 0.00 0.00 O

ATOM 1248 OX1 PHE P1248 11.462 1.238 -36.235 0.00 0.00 O

ATOM 1249 OX1 PHE P1249 11.280 5.761 -34.942 0.00 0.00 O

ATOM 1250 SI1 PHE P1250 13.645 -0.599 -38.027 0.00 0.00 S

ATOM 1251 SI1 PHE P1251 12.242 1.329 -34.822 0.00 0.00 S

ATOM 1252 SI1 PHE P1252 14.419 2.191 -37.857 0.00 0.00 S

ATOM 1253 OX1 PHE P1253 13.851 0.810 -37.321 0.00 0.00 O

ATOM 1254 OX1 PHE P1254 14.873 -1.512 -37.524 0.00 0.00 O

ATOM 1255 OX1 PHE P1255 15.033 -1.731 -34.786 0.00 0.00 O

ATOM 1256 OX1 PHE P1256 13.490 0.346 -34.704 0.00 0.00 O

ATOM 1257 OX1 PHE P1257 12.907 2.779 -34.735 0.00 0.00 O

ATOM 1258 OX1 PHE P1258 15.028 2.847 -36.538 0.00 0.00 O

ATOM 1259 SI1 PHE P1259 17.029 -3.116 -39.476 0.00 0.00 S

ATOM 1260 SI1 PHE P1260 15.780 -1.493 -36.175 0.00 0.00 S

ATOM 1261 SI1 PHE P1261 17.973 -0.357 -39.358 0.00 0.00 S

ATOM 1262 OX1 PHE P1262 17.150 -1.635 -38.872 0.00 0.00 O

ATOM 1263 OX1 PHE P1263 18.302 -4.038 -39.169 0.00 0.00 O

ATOM 1264 OX1 PHE P1264 18.313 -4.766 -36.534 0.00 0.00 O

ATOM 1265 OX1 PHE P1265 16.906 -2.614 -36.308 0.00 0.00 O

ATOM 1266 OX1 PHE P1266 16.624 -0.135 -35.988 0.00 0.00 O

ATOM 1267 OX1 PHE P1267 18.204 0.582 -38.076 0.00 0.00 O

ATOM 1268 SI1 PHE P1268 20.719 -5.836 -41.231 0.00 0.00 S

ATOM 1269 SI1 PHE P1269 19.123 -4.383 -37.830 0.00 0.00 S

ATOM 1270 SI1 PHE P1270 21.632 -3.037 -40.724 0.00 0.00 S

ATOM 1271 OX1 PHE P1271 20.855 -4.419 -40.507 0.00 0.00 O

ATOM 1272 OX1 PHE P1272 21.983 -6.739 -40.821 0.00 0.00 O

ATOM 1273 OX1 PHE P1273 21.788 -7.458 -38.235 0.00 0.00 O

ATOM 1274 OX1 PHE P1274 19.951 -5.690 -38.200 0.00 0.00 O

ATOM 1275 OX1 PHE P1275 20.048 -3.192 -37.322 0.00 0.00 O

ATOM 1276 OX1 PHE P1276 21.534 -2.274 -39.335 0.00 0.00 O

ATOM 1277 SI1 PHE P1277 24.406 -8.657 -42.807 0.00 0.00 S

ATOM 1278 SI1 PHE P1278 22.739 -7.021 -39.420 0.00 0.00 S

ATOM 1279 SI1 PHE P1279 24.919 -5.864 -42.323 0.00 0.00 S

ATOM 1280 OX1 PHE P1280 24.602 -7.355 -41.944 0.00 0.00 O

ATOM 1281 OX1 PHE P1281 25.588 -9.652 -42.479 0.00 0.00 O

ATOM 1282 OX1 PHE P1282 25.276 -10.215 -39.870 0.00 0.00 O

ATOM 1283 OX1 PHE P1283 23.627 -8.307 -39.715 0.00 0.00 O

ATOM 1284 OX1 PHE P1284 23.644 -5.794 -38.956 0.00 0.00 O

ATOM 1285 OX1 PHE P1285 25.398 -5.163 -40.988 0.00 0.00 O

ATOM 1286 SI1 PHE P1286 26.296 -9.820 -41.053 0.00 0.00 S

ATOM 1287 OX1 PHE P1287 29.336 -12.026 -40.527 0.00 0.00 O

ATOM 1288 OX1 PHE P1288 27.136 -11.147 -41.303 0.00 0.00 O

ATOM 1289 OX1 PHE P1289 27.210 -8.581 -40.553 0.00 0.00 O

ATOM 1290 OX1 PHE P1290 28.494 -7.721 -42.636 0.00 0.00 O

ATOM 1291 SI1 PHE P1291 29.986 -12.580 -41.870 0.00 0.00 S

ATOM 1292 OX1 PHE P1292 32.198 -15.151 -42.728 0.00 0.00 O

ATOM 1293 OX1 PHE P1293 29.924 -14.131 -41.989 0.00 0.00 O

ATOM 1294 OX1 PHE P1294 30.950 -11.807 -42.877 0.00 0.00 O

ATOM 1295 SI1 PHE P1295 14.355 3.327 -35.160 0.00 0.00 S

ATOM 1296 SI1 PHE P1296 18.091 0.314 -36.497 0.00 0.00 S

ATOM 1297 SI1 PHE P1297 18.940 3.071 -35.892 0.00 0.00 S

ATOM 1298 OX1 PHE P1298 18.420 1.600 -35.606 0.00 0.00 O

ATOM 1299 OX1 PHE P1299 19.196 -0.742 -36.060 0.00 0.00 O

ATOM 1300 SI1 PHE P1300 21.473 -2.662 -37.794 0.00 0.00 S

ATOM 1301 SI1 PHE P1301 19.829 -0.859 -34.598 0.00 0.00 S

ATOM 1302 SI1 PHE P1302 22.224 0.152 -37.578 0.00 0.00 S

ATOM 1303 OX1 PHE P1303 21.648 -1.252 -37.057 0.00 0.00 O

ATOM 1304 OX1 PHE P1304 22.633 -3.684 -37.348 0.00 0.00 O

ATOM 1305 OX1 PHE P1305 23.211 -3.364 -34.742 0.00 0.00 O

ATOM 1306 OX1 PHE P1306 20.463 -2.288 -34.353 0.00 0.00 O

ATOM 1307 OX1 PHE P1307 20.839 0.348 -34.271 0.00 0.00 O

ATOM 1308 OX1 PHE P1308 22.702 0.930 -36.268 0.00 0.00 O

ATOM 1309 SI1 PHE P1309 25.081 -5.266 -39.430 0.00 0.00 S

ATOM 1310 SI1 PHE P1310 23.760 -3.458 -36.224 0.00 0.00 S

ATOM 1311 SI1 PHE P1311 26.046 -2.536 -39.317 0.00 0.00 S

ATOM 1312 OX1 PHE P1312 25.038 -3.726 -38.994 0.00 0.00 O

ATOM 1313 OX1 PHE P1313 26.322 -6.147 -38.904 0.00 0.00 O

ATOM 1314 OX1 PHE P1314 26.370 -6.721 -36.246 0.00 0.00 O

ATOM 1315 OX1 PHE P1315 24.933 -4.535 -36.283 0.00 0.00 O

ATOM 1316 OX1 PHE P1316 24.398 -2.060 -36.590 0.00 0.00 O

ATOM 1317 OX1 PHE P1317 26.696 -2.168 -37.901 0.00 0.00 O

ATOM 1318 SI1 PHE P1318 28.600 -7.934 -41.066 0.00 0.00 S

ATOM 1319 SI1 PHE P1319 27.177 -6.399 -37.561 0.00 0.00 S

ATOM 1320 SI1 PHE P1320 29.529 -5.110 -40.565 0.00 0.00 S

ATOM 1321 OX1 PHE P1321 28.766 -6.510 -40.339 0.00 0.00 O

ATOM 1322 OX1 PHE P1322 29.848 -8.888 -40.721 0.00 0.00 O

ATOM 1323 OX1 PHE P1323 29.729 -9.642 -38.137 0.00 0.00 O

ATOM 1324 OX1 PHE P1324 28.049 -7.680 -37.954 0.00 0.00 O

ATOM 1325 OX1 PHE P1325 28.047 -5.142 -37.097 0.00 0.00 O

ATOM 1326 OX1 PHE P1326 29.291 -4.246 -39.244 0.00 0.00 O

ATOM 1327 SI1 PHE P1327 32.326 -11.059 -42.597 0.00 0.00 S

ATOM 1328 SI1 PHE P1328 30.641 -9.228 -39.356 0.00 0.00 S

ATOM 1329 SI1 PHE P1329 32.967 -8.236 -42.015 0.00 0.00 S

ATOM 1330 OX1 PHE P1330 32.086 -9.526 -42.285 0.00 0.00 O

ATOM 1331 OX1 PHE P1331 33.610 -11.867 -42.111 0.00 0.00 O

ATOM 1332 OX1 PHE P1332 33.379 -12.346 -39.509 0.00 0.00 O

ATOM 1333 OX1 PHE P1333 31.557 -10.505 -39.685 0.00 0.00 O

ATOM 1334 OX1 PHE P1334 31.498 -7.971 -38.900 0.00 0.00 O

ATOM 1335 OX1 PHE P1335 33.596 -8.180 -40.534 0.00 0.00 O

ATOM 1336 SI1 PHE P1336 34.343 -11.879 -40.695 0.00 0.00 S

ATOM 1337 OX1 PHE P1337 37.227 -14.482 -40.947 0.00 0.00 O

ATOM 1338 OX1 PHE P1338 35.505 -12.937 -40.808 0.00 0.00 O

ATOM 1339 OX1 PHE P1339 35.122 -10.505 -40.453 0.00 0.00 O

ATOM 1340 OX1 PHE P1340 36.184 -9.897 -42.774 0.00 0.00 O

ATOM 1341 SI1 PHE P1341 37.505 -14.781 -42.476 0.00 0.00 S

ATOM 1342 OX1 PHE P1342 39.668 -17.332 -43.334 0.00 0.00 O

ATOM 1343 OX1 PHE P1343 37.447 -16.330 -42.619 0.00 0.00 O

ATOM 1344 OX1 PHE P1344 38.440 -13.934 -43.464 0.00 0.00 O

ATOM 1345 SI1 PHE P1345 22.359 0.620 -34.727 0.00 0.00 S

ATOM 1346 SI1 PHE P1346 25.914 -1.604 -36.616 0.00 0.00 S

ATOM 1347 SI1 PHE P1347 27.000 0.916 -36.171 0.00 0.00 S

ATOM 1348 OX1 PHE P1348 25.828 -0.022 -36.667 0.00 0.00 O

ATOM 1349 OX1 PHE P1349 26.652 -2.084 -35.289 0.00 0.00 O

ATOM 1350 SI1 PHE P1350 29.385 -4.503 -37.680 0.00 0.00 S

ATOM 1351 SI1 PHE P1351 30.603 -1.960 -37.580 0.00 0.00 S

ATOM 1352 OX1 PHE P1352 29.592 -3.060 -37.003 0.00 0.00 O

ATOM 1353 OX1 PHE P1353 30.667 -5.396 -37.350 0.00 0.00 O

ATOM 1354 OX1 PHE P1354 31.315 -1.163 -36.407 0.00 0.00 O

ATOM 1355 SI1 PHE P1355 33.039 -7.638 -39.125 0.00 0.00 S

ATOM 1356 SI1 PHE P1356 31.371 -5.515 -35.921 0.00 0.00 S

ATOM 1357 SI1 PHE P1357 34.141 -4.924 -39.458 0.00 0.00 S

ATOM 1358 OX1 PHE P1358 33.021 -6.022 -39.121 0.00 0.00 O

ATOM 1359 OX1 PHE P1359 33.809 -8.322 -37.880 0.00 0.00 O

ATOM 1360 OX1 PHE P1360 32.456 -6.670 -35.904 0.00 0.00 O

ATOM 1361 OX1 PHE P1361 31.971 -4.143 -35.393 0.00 0.00 O

ATOM 1362 OX1 PHE P1362 35.012 -4.571 -38.183 0.00 0.00 O

ATOM 1363 SI1 PHE P1363 36.436 -9.950 -41.208 0.00 0.00 S

ATOM 1364 SI1 PHE P1364 35.358 -8.449 -37.480 0.00 0.00 S

ATOM 1365 SI1 PHE P1365 37.690 -7.321 -41.048 0.00 0.00 S

ATOM 1366 OX1 PHE P1366 36.679 -8.487 -40.642 0.00 0.00 O

ATOM 1367 OX1 PHE P1367 37.713 -10.891 -40.955 0.00 0.00 O

ATOM 1368 OX1 PHE P1368 37.465 -11.916 -38.600 0.00 0.00 O

ATOM 1369 OX1 PHE P1369 36.167 -9.687 -38.089 0.00 0.00 O

ATOM 1370 OX1 PHE P1370 36.091 -7.185 -38.087 0.00 0.00 O

ATOM 1371 OX1 PHE P1371 37.978 -6.514 -39.693 0.00 0.00 O

ATOM 1372 SI1 PHE P1372 39.842 -13.197 -43.250 0.00 0.00 S

ATOM 1373 SI1 PHE P1373 38.511 -11.422 -39.659 0.00 0.00 S

ATOM 1374 SI1 PHE P1374 40.736 -10.620 -42.332 0.00 0.00 S

ATOM 1375 OX1 PHE P1375 39.659 -11.736 -42.657 0.00 0.00 O

ATOM 1376 OX1 PHE P1376 41.198 -14.024 -43.022 0.00 0.00 O

ATOM 1377 OX1 PHE P1377 40.963 -14.739 -40.554 0.00 0.00 O

ATOM 1378 OX1 PHE P1378 39.339 -12.691 -40.190 0.00 0.00 O

ATOM 1379 OX1 PHE P1379 39.515 -10.403 -38.956 0.00 0.00 O

ATOM 1380 OX1 PHE P1380 41.370 -10.507 -40.860 0.00 0.00 O

ATOM 1381 SI1 PHE P1381 42.001 -14.274 -41.659 0.00 0.00 S

ATOM 1382 OX1 PHE P1382 45.036 -17.052 -40.975 0.00 0.00 O

ATOM 1383 OX1 PHE P1383 43.062 -15.466 -41.839 0.00 0.00 O

ATOM 1384 OX1 PHE P1384 42.782 -12.959 -41.191 0.00 0.00 O

ATOM 1385 OX1 PHE P1385 43.574 -11.930 -43.468 0.00 0.00 O

ATOM 1386 SI1 PHE P1386 45.570 -17.585 -42.378 0.00 0.00 S

ATOM 1387 OX1 PHE P1387 48.319 -20.632 -41.906 0.00 0.00 O

ATOM 1388 OX1 PHE P1388 46.759 -18.645 -42.357 0.00 0.00 O

ATOM 1389 OX1 PHE P1389 45.905 -16.411 -43.394 0.00 0.00 O

ATOM 1390 SI1 PHE P1390 37.657 -6.929 -38.183 0.00 0.00 S

ATOM 1391 SI1 PHE P1391 40.870 -9.775 -39.518 0.00 0.00 S

ATOM 1392 SI1 PHE P1392 41.817 -7.169 -40.255 0.00 0.00 S

ATOM 1393 OX1 PHE P1393 40.716 -8.190 -39.705 0.00 0.00 O

ATOM 1394 OX1 PHE P1394 41.975 -10.009 -38.403 0.00 0.00 O

ATOM 1395 SI1 PHE P1395 43.985 -12.238 -41.964 0.00 0.00 S

ATOM 1396 SI1 PHE P1396 45.308 -9.677 -41.687 0.00 0.00 S

ATOM 1397 OX1 PHE P1397 44.355 -10.876 -41.215 0.00 0.00 O

ATOM 1398 OX1 PHE P1398 45.232 -13.211 -41.944 0.00 0.00 O

ATOM 1399 SI1 PHE P1399 47.247 -15.566 -43.243 0.00 0.00 S

ATOM 1400 SI1 PHE P1400 48.261 -12.975 -43.163 0.00 0.00 S

ATOM 1401 OX1 PHE P1401 47.258 -14.061 -43.748 0.00 0.00 O

ATOM 1402 OX1 PHE P1402 48.634 -16.335 -43.120 0.00 0.00 O

ATOM 1403 SI1 PHE P1403 -34.694 -1.247 -44.483 0.00 0.00 S

ATOM 1404 OX1 PHE P1404 -33.741 -4.034 -42.974 0.00 0.00 O

ATOM 1405 OX1 PHE P1405 -35.090 -2.757 -44.786 0.00 0.00 O

ATOM 1406 OX1 PHE P1406 -33.799 -0.458 -45.529 0.00 0.00 O

ATOM 1407 SI1 PHE P1407 -32.656 0.552 -45.082 0.00 0.00 S

ATOM 1408 SI1 PHE P1408 -32.615 3.194 -43.973 0.00 0.00 S

ATOM 1409 OX1 PHE P1409 -33.191 1.726 -44.149 0.00 0.00 O

ATOM 1410 OX1 PHE P1410 -31.266 -0.123 -44.680 0.00 0.00 O

ATOM 1411 OX1 PHE P1411 -33.484 3.906 -42.847 0.00 0.00 O

ATOM 1412 SI1 PHE P1412 -30.880 -0.743 -43.259 0.00 0.00 S

ATOM 1413 OX1 PHE P1413 -28.045 -3.135 -42.720 0.00 0.00 O

ATOM 1414 OX1 PHE P1414 -30.312 -2.222 -43.497 0.00 0.00 O

ATOM 1415 OX1 PHE P1415 -29.893 0.279 -42.516 0.00 0.00 O

ATOM 1416 OX1 PHE P1416 -28.158 0.678 -44.419 0.00 0.00 O

ATOM 1417 SI1 PHE P1417 -27.264 -3.660 -44.008 0.00 0.00 S

ATOM 1418 OX1 PHE P1418 -24.922 -6.141 -44.648 0.00 0.00 O

ATOM 1419 OX1 PHE P1419 -27.306 -5.224 -44.229 0.00 0.00 O

ATOM 1420 OX1 PHE P1420 -26.200 -2.780 -44.818 0.00 0.00 O

ATOM 1421 SI1 PHE P1421 -28.404 0.756 -42.853 0.00 0.00 S

ATOM 1422 SI1 PHE P1422 -27.235 3.299 -41.976 0.00 0.00 S

ATOM 1423 OX1 PHE P1423 -28.369 2.324 -42.522 0.00 0.00 O

ATOM 1424 OX1 PHE P1424 -27.309 -0.233 -42.213 0.00 0.00 O

ATOM 1425 SI1 PHE P1425 -24.803 -2.148 -44.361 0.00 0.00 S

ATOM 1426 SI1 PHE P1426 -26.794 -0.789 -40.791 0.00 0.00 S

ATOM 1427 SI1 PHE P1427 -24.226 0.519 -43.401 0.00 0.00 S

ATOM 1428 OX1 PHE P1428 -24.930 -0.895 -43.409 0.00 0.00 O

ATOM 1429 OX1 PHE P1429 -23.562 -3.098 -44.031 0.00 0.00 O

ATOM 1430 OX1 PHE P1430 -24.371 -4.065 -41.744 0.00 0.00 O

ATOM 1431 OX1 PHE P1431 -25.898 -2.017 -41.279 0.00 0.00 O

ATOM 1432 OX1 PHE P1432 -25.883 0.223 -39.977 0.00 0.00 O

ATOM 1433 OX1 PHE P1433 -24.475 1.163 -41.966 0.00 0.00 O

ATOM 1434 SI1 PHE P1434 -23.099 -3.578 -42.576 0.00 0.00 S

ATOM 1435 OX1 PHE P1435 -20.183 -5.878 -41.775 0.00 0.00 O

ATOM 1436 OX1 PHE P1436 -22.284 -4.936 -42.731 0.00 0.00 O

ATOM 1437 OX1 PHE P1437 -22.211 -2.471 -41.805 0.00 0.00 O

ATOM 1438 OX1 PHE P1438 -20.632 -1.605 -43.664 0.00 0.00 O

ATOM 1439 SI1 PHE P1439 -19.445 -6.431 -43.079 0.00 0.00 S

ATOM 1440 OX1 PHE P1440 -17.091 -8.759 -44.055 0.00 0.00 O

ATOM 1441 OX1 PHE P1441 -19.465 -7.980 -43.280 0.00 0.00 O

ATOM 1442 OX1 PHE P1442 -18.594 -5.585 -44.127 0.00 0.00 O

ATOM 1443 SI1 PHE P1443 -24.463 0.785 -40.423 0.00 0.00 S

ATOM 1444 SI1 PHE P1444 -23.373 3.385 -40.132 0.00 0.00 S

ATOM 1445 OX1 PHE P1445 -24.352 2.204 -39.696 0.00 0.00 O

ATOM 1446 OX1 PHE P1446 -23.243 -0.210 -40.129 0.00 0.00 O

ATOM 1447 SI1 PHE P1447 -20.736 -1.874 -42.103 0.00 0.00 S

ATOM 1448 SI1 PHE P1448 -22.618 -0.717 -38.744 0.00 0.00 S

ATOM 1449 SI1 PHE P1449 -19.918 0.943 -41.376 0.00 0.00 S

ATOM 1450 OX1 PHE P1450 -20.657 -0.484 -41.311 0.00 0.00 O

ATOM 1451 OX1 PHE P1451 -19.548 -2.876 -41.667 0.00 0.00 O

ATOM 1452 OX1 PHE P1452 -19.824 -3.794 -39.152 0.00 0.00 O

ATOM 1453 OX1 PHE P1453 -21.670 -1.953 -39.110 0.00 0.00 O

ATOM 1454 OX1 PHE P1454 -21.766 0.441 -38.046 0.00 0.00 O

ATOM 1455 OX1 PHE P1455 -20.327 1.615 -39.981 0.00 0.00 O

ATOM 1456 SI1 PHE P1456 -17.263 -4.748 -43.887 0.00 0.00 S

ATOM 1457 SI1 PHE P1457 -18.828 -3.352 -40.297 0.00 0.00 S

ATOM 1458 SI1 PHE P1458 -16.713 -1.975 -43.210 0.00 0.00 S

ATOM 1459 OX1 PHE P1459 -17.597 -3.255 -43.487 0.00 0.00 O

ATOM 1460 OX1 PHE P1460 -15.886 -5.492 -43.557 0.00 0.00 O

ATOM 1461 OX1 PHE P1461 -16.398 -6.551 -41.216 0.00 0.00 O

ATOM 1462 OX1 PHE P1462 -17.920 -4.594 -40.730 0.00 0.00 O

ATOM 1463 OX1 PHE P1463 -17.899 -2.230 -39.702 0.00 0.00 O

ATOM 1464 OX1 PHE P1464 -16.068 -1.989 -41.753 0.00 0.00 O

ATOM 1465 SI1 PHE P1465 -15.267 -5.968 -42.164 0.00 0.00 S

ATOM 1466 OX1 PHE P1466 -12.262 -8.231 -41.629 0.00 0.00 O

ATOM 1467 OX1 PHE P1467 -14.425 -7.296 -42.440 0.00 0.00 O

ATOM 1468 OX1 PHE P1468 -14.393 -4.813 -41.465 0.00 0.00 O

ATOM 1469 OX1 PHE P1469 -13.116 -3.780 -43.480 0.00 0.00 O

ATOM 1470 SI1 PHE P1470 -11.593 -8.602 -43.031 0.00 0.00 S

ATOM 1471 OX1 PHE P1471 -9.348 -10.916 -44.216 0.00 0.00 O

ATOM 1472 OX1 PHE P1472 -11.667 -10.120 -43.368 0.00 0.00 O

ATOM 1473 OX1 PHE P1473 -10.618 -7.723 -43.944 0.00 0.00 O

ATOM 1474 SI1 PHE P1474 -20.373 1.110 -38.471 0.00 0.00 S

ATOM 1475 SI1 PHE P1475 -19.124 3.593 -37.942 0.00 0.00 S

ATOM 1476 OX1 PHE P1476 -20.132 2.410 -37.556 0.00 0.00 O

ATOM 1477 OX1 PHE P1477 -19.157 0.108 -38.276 0.00 0.00 O

ATOM 1478 SI1 PHE P1478 -16.495 -1.719 -40.229 0.00 0.00 S

ATOM 1479 SI1 PHE P1479 -15.467 0.944 -39.947 0.00 0.00 S

ATOM 1480 OX1 PHE P1480 -16.593 -0.167 -39.867 0.00 0.00 O

ATOM 1481 OX1 PHE P1481 -15.282 -2.575 -39.663 0.00 0.00 O

ATOM 1482 OX1 PHE P1482 -15.712 -3.308 -37.216 0.00 0.00 O

ATOM 1483 OX1 PHE P1483 -14.736 0.973 -38.535 0.00 0.00 O

ATOM 1484 SI1 PHE P1484 -13.010 -4.136 -41.937 0.00 0.00 S

ATOM 1485 SI1 PHE P1485 -14.604 -2.961 -38.287 0.00 0.00 S

ATOM 1486 SI1 PHE P1486 -12.087 -1.373 -41.197 0.00 0.00 S

ATOM 1487 OX1 PHE P1487 -12.840 -2.788 -41.094 0.00 0.00 O

ATOM 1488 OX1 PHE P1488 -11.791 -5.140 -41.679 0.00 0.00 O

ATOM 1489 OX1 PHE P1489 -11.965 -6.083 -39.148 0.00 0.00 O

ATOM 1490 OX1 PHE P1490 -13.742 -4.222 -38.774 0.00 0.00 O

ATOM 1491 OX1 PHE P1491 -13.723 -1.771 -37.695 0.00 0.00 O

ATOM 1492 OX1 PHE P1492 -12.466 -0.680 -39.803 0.00 0.00 O

ATOM 1493 SI1 PHE P1493 -9.277 -6.915 -43.633 0.00 0.00 S

ATOM 1494 SI1 PHE P1494 -11.034 -5.575 -40.330 0.00 0.00 S

ATOM 1495 SI1 PHE P1495 -8.705 -4.132 -43.055 0.00 0.00 S

ATOM 1496 OX1 PHE P1496 -9.598 -5.398 -43.346 0.00 0.00 O

ATOM 1497 OX1 PHE P1497 -7.899 -7.620 -43.230 0.00 0.00 O

ATOM 1498 OX1 PHE P1498 -8.249 -8.548 -40.760 0.00 0.00 O

ATOM 1499 OX1 PHE P1499 -10.118 -6.825 -40.720 0.00 0.00 O

ATOM 1500 OX1 PHE P1500 -10.172 -4.346 -39.841 0.00 0.00 O

ATOM 1501 OX1 PHE P1501 -8.074 -4.177 -41.591 0.00 0.00 O

ATOM 1502 SI1 PHE P1502 -7.213 -7.979 -41.829 0.00 0.00 S

ATOM 1503 OX1 PHE P1503 -4.214 -10.351 -41.700 0.00 0.00 O

ATOM 1504 OX1 PHE P1504 -6.398 -9.300 -42.195 0.00 0.00 O

ATOM 1505 OX1 PHE P1505 -6.266 -6.823 -41.207 0.00 0.00 O

ATOM 1506 OX1 PHE P1506 -4.735 -6.260 -43.237 0.00 0.00 O

ATOM 1507 SI1 PHE P1507 -3.701 -10.639 -43.203 0.00 0.00 S

ATOM 1508 OX1 PHE P1508 -0.988 -13.082 -43.326 0.00 0.00 O

ATOM 1509 OX1 PHE P1509 -3.485 -12.192 -43.541 0.00 0.00 O

ATOM 1510 OX1 PHE P1510 -2.690 -9.582 -43.850 0.00 0.00 O

ATOM 1511 SI1 PHE P1511 -15.481 0.949 -37.119 0.00 0.00 S

ATOM 1512 SI1 PHE P1512 -12.385 -1.124 -38.274 0.00 0.00 S

ATOM 1513 SI1 PHE P1513 -11.118 1.419 -37.924 0.00 0.00 S

ATOM 1514 OX1 PHE P1514 -12.079 0.226 -37.449 0.00 0.00 O

ATOM 1515 OX1 PHE P1515 -11.247 -2.215 -38.117 0.00 0.00 O

ATOM 1516 OX1 PHE P1516 -10.374 2.037 -36.668 0.00 0.00 O

ATOM 1517 SI1 PHE P1517 -8.658 -3.947 -40.118 0.00 0.00 S

ATOM 1518 SI1 PHE P1518 -10.263 -2.391 -36.889 0.00 0.00 S

ATOM 1519 SI1 PHE P1519 -7.524 -1.319 -39.697 0.00 0.00 S

ATOM 1520 OX1 PHE P1520 -8.695 -2.393 -39.729 0.00 0.00 O

ATOM 1521 OX1 PHE P1521 -7.782 -4.946 -39.243 0.00 0.00 O

ATOM 1522 OX1 PHE P1522 -7.110 -4.289 -36.871 0.00 0.00 O

ATOM 1523 OX1 PHE P1523 -9.662 -3.856 -37.059 0.00 0.00 O

ATOM 1524 OX1 PHE P1524 -9.335 -1.120 -37.074 0.00 0.00 O

ATOM 1525 OX1 PHE P1525 -6.992 -1.258 -38.178 0.00 0.00 O

ATOM 1526 SI1 PHE P1526 -4.782 -6.361 -41.652 0.00 0.00 S

ATOM 1527 SI1 PHE P1527 -6.550 -4.878 -38.235 0.00 0.00 S

ATOM 1528 SI1 PHE P1528 -3.698 -3.568 -41.408 0.00 0.00 S

ATOM 1529 OX1 PHE P1529 -4.371 -4.970 -41.004 0.00 0.00 O

ATOM 1530 OX1 PHE P1530 -3.674 -7.377 -41.110 0.00 0.00 O

ATOM 1531 OX1 PHE P1531 -3.964 -8.032 -38.491 0.00 0.00 O

ATOM 1532 OX1 PHE P1532 -6.202 -6.450 -38.177 0.00 0.00 O

ATOM 1533 OX1 PHE P1533 -5.460 -3.927 -38.889 0.00 0.00 O

ATOM 1534 OX1 PHE P1534 -3.533 -2.812 -40.020 0.00 0.00 O

ATOM 1535 SI1 PHE P1535 -1.331 -8.997 -43.243 0.00 0.00 S

ATOM 1536 SI1 PHE P1536 -2.970 -7.626 -39.678 0.00 0.00 S

ATOM 1537 SI1 PHE P1537 -0.704 -6.240 -42.704 0.00 0.00 S

ATOM 1538 OX1 PHE P1538 -1.372 -7.648 -42.427 0.00 0.00 O

ATOM 1539 OX1 PHE P1539 -0.092 -9.937 -42.929 0.00 0.00 O

ATOM 1540 OX1 PHE P1540 -0.334 -10.717 -40.330 0.00 0.00 O

ATOM 1541 OX1 PHE P1541 -2.048 -8.878 -40.028 0.00 0.00 O

ATOM 1542 OX1 PHE P1542 -2.015 -6.428 -39.237 0.00 0.00 O

ATOM 1543 OX1 PHE P1543 -0.787 -5.436 -41.344 0.00 0.00 O

ATOM 1544 SI1 PHE P1544 0.633 -10.243 -41.535 0.00 0.00 S

ATOM 1545 OX1 PHE P1545 3.692 -12.483 -41.468 0.00 0.00 O

ATOM 1546 OX1 PHE P1546 1.411 -11.582 -41.932 0.00 0.00 O

ATOM 1547 OX1 PHE P1547 1.616 -9.082 -40.996 0.00 0.00 O

ATOM 1548 OX1 PHE P1548 3.015 -8.288 -43.050 0.00 0.00 O

ATOM 1549 SI1 PHE P1549 4.263 -12.790 -42.941 0.00 0.00 S

ATOM 1550 OX1 PHE P1550 6.998 -15.206 -42.961 0.00 0.00 O

ATOM 1551 OX1 PHE P1551 4.494 -14.355 -43.193 0.00 0.00 O

ATOM 1552 OX1 PHE P1552 5.327 -11.757 -43.562 0.00 0.00 O

ATOM 1553 SI1 PHE P1553 -7.810 -0.756 -36.891 0.00 0.00 S

ATOM 1554 SI1 PHE P1554 -6.905 1.924 -36.247 0.00 0.00 S

ATOM 1555 OX1 PHE P1555 -7.901 0.855 -36.878 0.00 0.00 O

ATOM 1556 OX1 PHE P1556 -7.372 -1.460 -35.513 0.00 0.00 O

ATOM 1557 SI1 PHE P1557 -4.095 -3.155 -38.570 0.00 0.00 S

ATOM 1558 SI1 PHE P1558 -5.957 -1.736 -34.819 0.00 0.00 S

ATOM 1559 SI1 PHE P1559 -3.162 -0.612 -37.745 0.00 0.00 S

ATOM 1560 OX1 PHE P1560 -4.316 -1.700 -37.935 0.00 0.00 O

ATOM 1561 OX1 PHE P1561 -2.890 -4.008 -37.958 0.00 0.00 O

ATOM 1562 OX1 PHE P1562 -3.393 -4.925 -35.614 0.00 0.00 O

ATOM 1563 OX1 PHE P1563 -5.078 -2.910 -35.452 0.00 0.00 O

ATOM 1564 OX1 PHE P1564 -5.121 -0.419 -34.966 0.00 0.00 O

ATOM 1565 OX1 PHE P1565 -2.755 -0.641 -36.194 0.00 0.00 O

ATOM 1566 SI1 PHE P1566 -0.649 -5.808 -39.821 0.00 0.00 S

ATOM 1567 SI1 PHE P1567 -2.247 -4.356 -36.536 0.00 0.00 S

ATOM 1568 SI1 PHE P1568 0.348 -3.126 -39.383 0.00 0.00 S

ATOM 1569 OX1 PHE P1569 -0.556 -4.392 -39.102 0.00 0.00 O

ATOM 1570 OX1 PHE P1570 0.676 -6.674 -39.521 0.00 0.00 O

ATOM 1571 OX1 PHE P1571 0.379 -7.537 -37.074 0.00 0.00 O

ATOM 1572 OX1 PHE P1572 -1.268 -5.554 -36.880 0.00 0.00 O

ATOM 1573 OX1 PHE P1573 -1.334 -3.173 -35.948 0.00 0.00 O

ATOM 1574 OX1 PHE P1574 0.430 -2.371 -37.957 0.00 0.00 O

ATOM 1575 SI1 PHE P1575 3.049 -8.513 -41.480 0.00 0.00 S

ATOM 1576 SI1 PHE P1576 1.428 -7.035 -38.138 0.00 0.00 S

ATOM 1577 SI1 PHE P1577 4.057 -5.762 -40.915 0.00 0.00 S

ATOM 1578 OX1 PHE P1578 3.296 -7.147 -40.699 0.00 0.00 O

ATOM 1579 OX1 PHE P1579 4.256 -9.477 -41.072 0.00 0.00 O

ATOM 1580 OX1 PHE P1580 4.022 -10.239 -38.483 0.00 0.00 O

ATOM 1581 OX1 PHE P1581 2.371 -8.289 -38.426 0.00 0.00 O

ATOM 1582 OX1 PHE P1582 2.308 -5.835 -37.555 0.00 0.00 O

ATOM 1583 OX1 PHE P1583 3.958 -4.988 -39.532 0.00 0.00 O

ATOM 1584 SI1 PHE P1584 6.712 -11.215 -42.958 0.00 0.00 S

ATOM 1585 SI1 PHE P1585 4.980 -9.804 -39.668 0.00 0.00 S

ATOM 1586 SI1 PHE P1586 7.264 -8.389 -42.467 0.00 0.00 S

ATOM 1587 OX1 PHE P1587 6.758 -9.856 -42.150 0.00 0.00 O

ATOM 1588 OX1 PHE P1588 7.864 -12.222 -42.533 0.00 0.00 O

ATOM 1589 OX1 PHE P1589 7.716 -12.803 -39.844 0.00 0.00 O

ATOM 1590 OX1 PHE P1590 5.803 -11.129 -40.007 0.00 0.00 O

ATOM 1591 OX1 PHE P1591 5.905 -8.623 -39.102 0.00 0.00 O

ATOM 1592 OX1 PHE P1592 7.322 -7.608 -41.091 0.00 0.00 O

ATOM 1593 SI1 PHE P1593 8.618 -12.417 -41.128 0.00 0.00 S

ATOM 1594 OX1 PHE P1594 11.701 -14.594 -41.063 0.00 0.00 O

ATOM 1595 OX1 PHE P1595 9.400 -13.762 -41.483 0.00 0.00 O

ATOM 1596 OX1 PHE P1596 9.571 -11.204 -40.675 0.00 0.00 O

ATOM 1597 OX1 PHE P1597 10.884 -10.378 -42.768 0.00 0.00 O

ATOM 1598 SI1 PHE P1598 12.273 -14.921 -42.531 0.00 0.00 S

ATOM 1599 OX1 PHE P1599 15.002 -17.323 -42.324 0.00 0.00 O

ATOM 1600 OX1 PHE P1600 12.523 -16.486 -42.749 0.00 0.00 O

ATOM 1601 OX1 PHE P1601 13.323 -13.894 -43.181 0.00 0.00 O

ATOM 1602 SI1 PHE P1602 -3.562 -0.175 -34.886 0.00 0.00 S

ATOM 1603 SI1 PHE P1603 0.145 -2.672 -36.384 0.00 0.00 S

ATOM 1604 SI1 PHE P1604 1.191 0.017 -36.130 0.00 0.00 S

ATOM 1605 OX1 PHE P1605 0.279 -1.226 -35.664 0.00 0.00 O

ATOM 1606 OX1 PHE P1606 1.263 -3.712 -35.866 0.00 0.00 O

ATOM 1607 OX1 PHE P1607 1.872 0.717 -34.877 0.00 0.00 O

ATOM 1608 SI1 PHE P1608 3.749 -5.271 -37.983 0.00 0.00 S

ATOM 1609 SI1 PHE P1609 2.359 -3.516 -34.704 0.00 0.00 S

ATOM 1610 SI1 PHE P1610 4.835 -2.614 -37.708 0.00 0.00 S

ATOM 1611 OX1 PHE P1611 3.774 -3.771 -37.425 0.00 0.00 O

ATOM 1612 OX1 PHE P1612 4.965 -6.199 -37.512 0.00 0.00 O

ATOM 1613 OX1 PHE P1613 5.009 -6.727 -34.817 0.00 0.00 O

ATOM 1614 OX1 PHE P1614 3.540 -4.582 -34.773 0.00 0.00 O

ATOM 1615 OX1 PHE P1615 3.068 -2.127 -34.987 0.00 0.00 O

ATOM 1616 OX1 PHE P1616 5.421 -2.262 -36.261 0.00 0.00 O

ATOM 1617 SI1 PHE P1617 7.300 -7.973 -39.559 0.00 0.00 S

ATOM 1618 SI1 PHE P1618 5.803 -6.455 -36.164 0.00 0.00 S

ATOM 1619 SI1 PHE P1619 8.275 -5.279 -39.200 0.00 0.00 S

ATOM 1620 OX1 PHE P1620 7.360 -6.537 -38.855 0.00 0.00 O

ATOM 1621 OX1 PHE P1621 8.594 -8.848 -39.196 0.00 0.00 O

ATOM 1622 OX1 PHE P1622 8.361 -9.644 -36.676 0.00 0.00 O

ATOM 1623 OX1 PHE P1623 6.576 -7.790 -36.560 0.00 0.00 O

ATOM 1624 OX1 PHE P1624 6.763 -5.233 -35.772 0.00 0.00 O

ATOM 1625 OX1 PHE P1625 8.380 -4.430 -37.841 0.00 0.00 O

ATOM 1626 SI1 PHE P1626 10.971 -10.610 -41.201 0.00 0.00 S

ATOM 1627 SI1 PHE P1627 9.341 -9.166 -37.807 0.00 0.00 S

ATOM 1628 SI1 PHE P1628 11.980 -7.877 -40.595 0.00 0.00 S

ATOM 1629 OX1 PHE P1629 11.178 -9.243 -40.412 0.00 0.00 O

ATOM 1630 OX1 PHE P1630 12.200 -11.560 -40.828 0.00 0.00 O

ATOM 1631 OX1 PHE P1631 11.988 -12.282 -38.210 0.00 0.00 O

ATOM 1632 OX1 PHE P1632 10.284 -10.413 -38.108 0.00 0.00 O

ATOM 1633 OX1 PHE P1633 10.186 -7.937 -37.260 0.00 0.00 O

ATOM 1634 OX1 PHE P1634 11.836 -7.083 -39.234 0.00 0.00 O

ATOM 1635 SI1 PHE P1635 14.746 -13.394 -42.636 0.00 0.00 S

ATOM 1636 SI1 PHE P1636 12.928 -11.859 -39.423 0.00 0.00 S

ATOM 1637 SI1 PHE P1637 15.269 -10.574 -42.222 0.00 0.00 S

ATOM 1638 OX1 PHE P1638 14.913 -12.056 -41.822 0.00 0.00 O

ATOM 1639 OX1 PHE P1639 15.878 -14.409 -42.199 0.00 0.00 O

ATOM 1640 OX1 PHE P1640 15.637 -14.870 -39.519 0.00 0.00 O

ATOM 1641 OX1 PHE P1641 13.732 -13.207 -39.713 0.00 0.00 O

ATOM 1642 OX1 PHE P1642 13.865 -10.649 -38.938 0.00 0.00 O

ATOM 1643 OX1 PHE P1643 15.657 -9.852 -40.872 0.00 0.00 O

ATOM 1644 SI1 PHE P1644 16.599 -14.529 -40.772 0.00 0.00 S

ATOM 1645 OX1 PHE P1645 19.643 -16.731 -40.317 0.00 0.00 O

ATOM 1646 OX1 PHE P1646 17.398 -15.878 -41.055 0.00 0.00 O

ATOM 1647 OX1 PHE P1647 17.530 -13.287 -40.336 0.00 0.00 O

ATOM 1648 OX1 PHE P1648 18.914 -12.590 -42.426 0.00 0.00 O

ATOM 1649 SI1 PHE P1649 20.289 -17.124 -41.712 0.00 0.00 S

ATOM 1650 OX1 PHE P1650 22.541 -19.628 -42.694 0.00 0.00 O

ATOM 1651 OX1 PHE P1651 20.251 -18.667 -41.975 0.00 0.00 O

ATOM 1652 OX1 PHE P1652 21.095 -16.146 -42.687 0.00 0.00 O

ATOM 1653 SI1 PHE P1653 4.597 -1.685 -35.008 0.00 0.00 S

ATOM 1654 SI1 PHE P1654 5.676 0.842 -34.537 0.00 0.00 S

ATOM 1655 OX1 PHE P1655 4.531 -0.095 -35.089 0.00 0.00 O

ATOM 1656 OX1 PHE P1656 5.297 -2.172 -33.664 0.00 0.00 O

ATOM 1657 SI1 PHE P1657 8.191 -4.691 -36.261 0.00 0.00 S

ATOM 1658 SI1 PHE P1658 9.208 -1.990 -35.989 0.00 0.00 S

ATOM 1659 OX1 PHE P1659 8.256 -3.219 -35.580 0.00 0.00 O

ATOM 1660 OX1 PHE P1660 9.392 -5.645 -35.773 0.00 0.00 O

ATOM 1661 OX1 PHE P1661 9.851 -1.315 -34.706 0.00 0.00 O

ATOM 1662 SI1 PHE P1662 11.620 -7.402 -37.703 0.00 0.00 S

ATOM 1663 SI1 PHE P1663 10.287 -5.552 -34.442 0.00 0.00 S

ATOM 1664 SI1 PHE P1664 12.745 -4.751 -37.386 0.00 0.00 S

ATOM 1665 OX1 PHE P1665 11.718 -5.930 -37.086 0.00 0.00 O

ATOM 1666 OX1 PHE P1666 12.752 -8.438 -37.273 0.00 0.00 O

ATOM 1667 OX1 PHE P1667 13.100 -8.653 -34.567 0.00 0.00 O

ATOM 1668 OX1 PHE P1668 11.518 -6.578 -34.427 0.00 0.00 O

ATOM 1669 OX1 PHE P1669 10.979 -4.119 -34.487 0.00 0.00 O

ATOM 1670 OX1 PHE P1670 13.283 -4.320 -35.946 0.00 0.00 O

ATOM 1671 SI1 PHE P1671 15.318 -10.082 -39.338 0.00 0.00 S

ATOM 1672 SI1 PHE P1672 13.738 -8.503 -36.021 0.00 0.00 S

ATOM 1673 SI1 PHE P1673 16.417 -7.457 -38.896 0.00 0.00 S

ATOM 1674 OX1 PHE P1674 15.298 -8.582 -38.781 0.00 0.00 O

ATOM 1675 OX1 PHE P1675 16.562 -10.969 -38.841 0.00 0.00 O

ATOM 1676 OX1 PHE P1676 16.427 -11.606 -36.269 0.00 0.00 O

ATOM 1677 OX1 PHE P1677 14.631 -9.766 -36.366 0.00 0.00 O

ATOM 1678 OX1 PHE P1678 14.576 -7.159 -36.108 0.00 0.00 O

ATOM 1679 OX1 PHE P1679 16.934 -7.288 -37.376 0.00 0.00 O

ATOM 1680 SI1 PHE P1680 18.946 -12.709 -40.843 0.00 0.00 S

ATOM 1681 SI1 PHE P1681 17.358 -11.220 -37.468 0.00 0.00 S

ATOM 1682 SI1 PHE P1682 19.948 -9.922 -40.463 0.00 0.00 S

ATOM 1683 OX1 PHE P1683 19.146 -11.277 -40.178 0.00 0.00 O

ATOM 1684 OX1 PHE P1684 20.161 -13.628 -40.355 0.00 0.00 O

ATOM 1685 OX1 PHE P1685 19.866 -14.425 -37.800 0.00 0.00 O

ATOM 1686 OX1 PHE P1686 18.203 -12.541 -37.740 0.00 0.00 O

ATOM 1687 OX1 PHE P1687 18.265 -9.990 -37.017 0.00 0.00 O

ATOM 1688 OX1 PHE P1688 19.838 -9.144 -39.076 0.00 0.00 O

ATOM 1689 SI1 PHE P1689 22.499 -15.411 -42.482 0.00 0.00 S

ATOM 1690 SI1 PHE P1690 20.866 -13.941 -38.935 0.00 0.00 S

ATOM 1691 SI1 PHE P1691 23.105 -12.659 -41.859 0.00 0.00 S

ATOM 1692 OX1 PHE P1692 22.461 -14.082 -41.635 0.00 0.00 O

ATOM 1693 OX1 PHE P1693 23.791 -16.290 -42.191 0.00 0.00 O

ATOM 1694 OX1 PHE P1694 23.376 -17.083 -39.666 0.00 0.00 O

ATOM 1695 OX1 PHE P1695 21.776 -15.184 -39.323 0.00 0.00 O

ATOM 1696 OX1 PHE P1696 21.774 -12.751 -38.381 0.00 0.00 O

ATOM 1697 OX1 PHE P1697 23.050 -11.889 -40.482 0.00 0.00 O

ATOM 1698 SI1 PHE P1698 24.432 -16.602 -40.759 0.00 0.00 S

ATOM 1699 OX1 PHE P1699 27.408 -18.926 -40.285 0.00 0.00 O

ATOM 1700 OX1 PHE P1700 25.246 -17.957 -40.955 0.00 0.00 O

ATOM 1701 OX1 PHE P1701 25.327 -15.392 -40.182 0.00 0.00 O

ATOM 1702 OX1 PHE P1702 26.694 -14.599 -42.252 0.00 0.00 O

ATOM 1703 SI1 PHE P1703 27.811 -19.475 -41.737 0.00 0.00 S

ATOM 1704 OX1 PHE P1704 30.410 -21.525 -42.372 0.00 0.00 O

ATOM 1705 OX1 PHE P1705 27.921 -21.058 -41.812 0.00 0.00 O

ATOM 1706 OX1 PHE P1706 28.842 -18.640 -42.632 0.00 0.00 O

ATOM 1707 SI1 PHE P1707 12.510 -3.692 -34.690 0.00 0.00 S

ATOM 1708 SI1 PHE P1708 13.640 -1.169 -34.268 0.00 0.00 S

ATOM 1709 OX1 PHE P1709 12.494 -2.107 -34.824 0.00 0.00 O

ATOM 1710 OX1 PHE P1710 13.300 -4.159 -33.389 0.00 0.00 O

ATOM 1711 SI1 PHE P1711 16.125 -6.788 -36.067 0.00 0.00 S

ATOM 1712 SI1 PHE P1712 17.071 -4.119 -35.809 0.00 0.00 S

ATOM 1713 OX1 PHE P1713 15.953 -5.189 -36.133 0.00 0.00 O

ATOM 1714 OX1 PHE P1714 16.776 -7.355 -34.690 0.00 0.00 O

ATOM 1715 OX1 PHE P1715 17.250 -4.079 -34.232 0.00 0.00 O

ATOM 1716 SI1 PHE P1716 19.690 -9.471 -37.520 0.00 0.00 S

ATOM 1717 SI1 PHE P1717 18.264 -7.422 -34.048 0.00 0.00 S

ATOM 1718 SI1 PHE P1718 20.717 -6.749 -37.299 0.00 0.00 S

ATOM 1719 OX1 PHE P1719 19.813 -8.001 -36.868 0.00 0.00 O

ATOM 1720 OX1 PHE P1720 20.834 -10.498 -37.042 0.00 0.00 O

ATOM 1721 OX1 PHE P1721 21.022 -10.541 -34.335 0.00 0.00 O

ATOM 1722 OX1 PHE P1722 19.249 -8.628 -34.425 0.00 0.00 O

ATOM 1723 OX1 PHE P1723 19.135 -6.143 -34.439 0.00 0.00 O

ATOM 1724 OX1 PHE P1724 21.296 -5.982 -36.013 0.00 0.00 O

ATOM 1725 SI1 PHE P1725 23.148 -12.143 -38.934 0.00 0.00 S

ATOM 1726 SI1 PHE P1726 21.771 -10.408 -35.741 0.00 0.00 S

ATOM 1727 SI1 PHE P1727 24.256 -9.492 -38.857 0.00 0.00 S

ATOM 1728 OX1 PHE P1728 23.201 -10.620 -38.467 0.00 0.00 O

ATOM 1729 OX1 PHE P1729 24.457 -12.984 -38.528 0.00 0.00 O

ATOM 1730 OX1 PHE P1730 24.536 -13.549 -35.840 0.00 0.00 O

ATOM 1731 OX1 PHE P1731 22.997 -11.435 -35.755 0.00 0.00 O

ATOM 1732 OX1 PHE P1732 22.458 -8.988 -35.916 0.00 0.00 O

ATOM 1733 OX1 PHE P1733 24.800 -9.120 -37.396 0.00 0.00 O

ATOM 1734 SI1 PHE P1734 26.739 -14.797 -40.678 0.00 0.00 S

ATOM 1735 SI1 PHE P1735 25.316 -13.210 -37.178 0.00 0.00 S

ATOM 1736 SI1 PHE P1736 27.785 -12.020 -40.152 0.00 0.00 S

ATOM 1737 OX1 PHE P1737 26.965 -13.387 -39.942 0.00 0.00 O

ATOM 1738 OX1 PHE P1738 27.911 -15.819 -40.309 0.00 0.00 O

ATOM 1739 OX1 PHE P1739 27.876 -16.437 -37.663 0.00 0.00 O

ATOM 1740 OX1 PHE P1740 26.210 -14.461 -37.576 0.00 0.00 O

ATOM 1741 OX1 PHE P1741 26.209 -11.972 -36.711 0.00 0.00 O

ATOM 1742 OX1 PHE P1742 27.561 -11.154 -38.828 0.00 0.00 O

ATOM 1743 SI1 PHE P1743 30.214 -17.929 -42.215 0.00 0.00 S

ATOM 1744 SI1 PHE P1744 28.713 -16.089 -38.945 0.00 0.00 S

ATOM 1745 SI1 PHE P1745 31.043 -15.179 -41.639 0.00 0.00 S

ATOM 1746 OX1 PHE P1746 30.061 -16.401 -41.856 0.00 0.00 O

ATOM 1747 OX1 PHE P1747 31.490 -18.760 -41.779 0.00 0.00 O

ATOM 1748 OX1 PHE P1748 31.465 -19.110 -39.084 0.00 0.00 O

ATOM 1749 OX1 PHE P1749 29.601 -17.388 -39.216 0.00 0.00 O

ATOM 1750 OX1 PHE P1750 29.530 -14.773 -38.635 0.00 0.00 O

ATOM 1751 OX1 PHE P1751 31.678 -15.027 -40.164 0.00 0.00 O

ATOM 1752 SI1 PHE P1752 32.312 -18.829 -40.417 0.00 0.00 S

ATOM 1753 OX1 PHE P1753 35.331 -21.103 -40.187 0.00 0.00 O

ATOM 1754 OX1 PHE P1754 33.054 -20.228 -40.671 0.00 0.00 O

ATOM 1755 OX1 PHE P1755 33.236 -17.548 -40.121 0.00 0.00 O

ATOM 1756 OX1 PHE P1756 34.493 -17.056 -42.338 0.00 0.00 O

ATOM 1757 SI1 PHE P1757 35.838 -21.734 -41.566 0.00 0.00 S

ATOM 1758 OX1 PHE P1758 37.966 -24.140 -42.668 0.00 0.00 O

ATOM 1759 OX1 PHE P1759 35.773 -23.299 -41.628 0.00 0.00 O

ATOM 1760 OX1 PHE P1760 36.663 -20.995 -42.720 0.00 0.00 O

ATOM 1761 SI1 PHE P1761 20.734 -6.032 -34.509 0.00 0.00 S

ATOM 1762 SI1 PHE P1762 23.982 -8.554 -36.119 0.00 0.00 S

ATOM 1763 SI1 PHE P1763 25.048 -6.021 -35.733 0.00 0.00 S

ATOM 1764 OX1 PHE P1764 23.887 -6.970 -36.218 0.00 0.00 O

ATOM 1765 OX1 PHE P1765 24.776 -9.017 -34.821 0.00 0.00 O

ATOM 1766 SI1 PHE P1766 27.589 -11.393 -37.261 0.00 0.00 S

ATOM 1767 SI1 PHE P1767 28.790 -8.832 -37.139 0.00 0.00 S

ATOM 1768 OX1 PHE P1768 27.770 -9.939 -36.598 0.00 0.00 O

ATOM 1769 OX1 PHE P1769 28.851 -12.323 -36.950 0.00 0.00 O

ATOM 1770 OX1 PHE P1770 29.616 -8.230 -35.931 0.00 0.00 O

ATOM 1771 SI1 PHE P1771 31.077 -14.479 -38.773 0.00 0.00 S

ATOM 1772 SI1 PHE P1772 29.637 -12.468 -35.570 0.00 0.00 S

ATOM 1773 SI1 PHE P1773 32.135 -11.683 -38.767 0.00 0.00 S

ATOM 1774 OX1 PHE P1774 31.055 -12.864 -38.644 0.00 0.00 O

ATOM 1775 OX1 PHE P1775 31.716 -15.252 -37.523 0.00 0.00 O

ATOM 1776 OX1 PHE P1776 33.138 -15.516 -35.431 0.00 0.00 O

ATOM 1777 OX1 PHE P1777 30.249 -13.921 -35.390 0.00 0.00 O

ATOM 1778 OX1 PHE P1778 30.743 -11.350 -35.323 0.00 0.00 O

ATOM 1779 OX1 PHE P1779 32.478 -10.936 -37.396 0.00 0.00 O

ATOM 1780 SI1 PHE P1780 34.599 -16.986 -40.756 0.00 0.00 S

ATOM 1781 SI1 PHE P1781 33.206 -15.485 -37.012 0.00 0.00 S

ATOM 1782 SI1 PHE P1782 35.851 -14.342 -40.160 0.00 0.00 S

ATOM 1783 OX1 PHE P1783 34.685 -15.428 -40.367 0.00 0.00 O

ATOM 1784 OX1 PHE P1784 35.846 -17.912 -40.361 0.00 0.00 O

ATOM 1785 OX1 PHE P1785 35.775 -18.720 -37.837 0.00 0.00 O

ATOM 1786 OX1 PHE P1786 33.941 -16.800 -37.585 0.00 0.00 O

ATOM 1787 OX1 PHE P1787 33.913 -14.140 -37.408 0.00 0.00 O

ATOM 1788 OX1 PHE P1788 36.216 -14.313 -38.595 0.00 0.00 O

ATOM 1789 SI1 PHE P1789 37.994 -20.114 -42.672 0.00 0.00 S

ATOM 1790 SI1 PHE P1790 36.694 -18.299 -39.055 0.00 0.00 S

ATOM 1791 SI1 PHE P1791 38.581 -17.332 -42.179 0.00 0.00 S

ATOM 1792 OX1 PHE P1792 37.695 -18.638 -42.209 0.00 0.00 O

ATOM 1793 OX1 PHE P1793 39.438 -20.753 -42.437 0.00 0.00 O

ATOM 1794 OX1 PHE P1794 39.212 -21.536 -39.988 0.00 0.00 O

ATOM 1795 OX1 PHE P1795 37.567 -19.548 -39.561 0.00 0.00 O

ATOM 1796 OX1 PHE P1796 37.631 -17.109 -38.599 0.00 0.00 O

ATOM 1797 OX1 PHE P1797 39.320 -17.089 -40.761 0.00 0.00 O

ATOM 1798 SI1 PHE P1798 40.221 -20.957 -41.064 0.00 0.00 S

ATOM 1799 OX1 PHE P1799 43.438 -23.545 -40.745 0.00 0.00 O

ATOM 1800 OX1 PHE P1800 41.336 -22.070 -41.332 0.00 0.00 O

ATOM 1801 OX1 PHE P1801 40.906 -19.617 -40.510 0.00 0.00 O

ATOM 1802 OX1 PHE P1802 42.013 -18.596 -42.659 0.00 0.00 O

ATOM 1803 SI1 PHE P1803 43.851 -23.751 -42.272 0.00 0.00 S

ATOM 1804 OX1 PHE P1804 46.564 -26.782 -42.880 0.00 0.00 O

ATOM 1805 OX1 PHE P1805 45.080 -24.710 -42.588 0.00 0.00 O

ATOM 1806 OX1 PHE P1806 43.935 -22.411 -43.120 0.00 0.00 O

ATOM 1807 SI1 PHE P1807 32.251 -11.261 -35.848 0.00 0.00 S

ATOM 1808 SI1 PHE P1808 35.416 -13.698 -37.348 0.00 0.00 S

ATOM 1809 SI1 PHE P1809 36.565 -11.139 -37.549 0.00 0.00 S

ATOM 1810 OX1 PHE P1810 35.321 -12.107 -37.439 0.00 0.00 O

ATOM 1811 SI1 PHE P1811 38.993 -16.607 -39.247 0.00 0.00 S

ATOM 1812 SI1 PHE P1812 40.064 -13.944 -39.505 0.00 0.00 S

ATOM 1813 OX1 PHE P1813 38.962 -15.012 -39.071 0.00 0.00 O

ATOM 1814 OX1 PHE P1814 40.184 -17.180 -38.367 0.00 0.00 O

ATOM 1815 SI1 PHE P1815 42.195 -18.883 -41.109 0.00 0.00 S

ATOM 1816 SI1 PHE P1816 43.607 -16.409 -40.656 0.00 0.00 S

ATOM 1817 OX1 PHE P1817 42.511 -17.531 -40.319 0.00 0.00 O

ATOM 1818 OX1 PHE P1818 43.431 -19.850 -40.909 0.00 0.00 O

ATOM 1819 SI1 PHE P1819 45.284 -21.571 -43.164 0.00 0.00 S

ATOM 1820 SI1 PHE P1820 46.936 -19.968 -41.495 0.00 0.00 S

ATOM 1821 OX1 PHE P1821 45.754 -20.996 -41.756 0.00 0.00 O

ATOM 1822 OX1 PHE P1822 46.381 -21.996 -44.231 0.00 0.00 O

ATOM 1823 SI1 PHE P1823 -36.425 -8.492 -45.088 0.00 0.00 S

ATOM 1824 OX1 PHE P1824 -36.020 -12.611 -45.274 0.00 0.00 O

ATOM 1825 OX1 PHE P1825 -36.473 -9.964 -45.681 0.00 0.00 O

ATOM 1826 OX1 PHE P1826 -35.696 -7.364 -45.935 0.00 0.00 O

ATOM 1827 SI1 PHE P1827 -34.876 -6.172 -45.276 0.00 0.00 S

ATOM 1828 SI1 PHE P1828 -35.172 -3.867 -43.649 0.00 0.00 S

ATOM 1829 OX1 PHE P1829 -35.674 -5.256 -44.248 0.00 0.00 O

ATOM 1830 OX1 PHE P1830 -33.388 -6.491 -44.821 0.00 0.00 O

ATOM 1831 SI1 PHE P1831 -32.900 -7.442 -43.637 0.00 0.00 S

ATOM 1832 OX1 PHE P1832 -30.037 -9.748 -44.327 0.00 0.00 O

ATOM 1833 OX1 PHE P1833 -32.428 -8.830 -44.278 0.00 0.00 O

ATOM 1834 OX1 PHE P1834 -31.846 -6.569 -42.818 0.00 0.00 O

ATOM 1835 OX1 PHE P1835 -30.498 -5.477 -44.779 0.00 0.00 O

ATOM 1836 SI1 PHE P1836 -28.765 -10.692 -44.158 0.00 0.00 S

ATOM 1837 OX1 PHE P1837 -26.742 -13.435 -44.110 0.00 0.00 O

ATOM 1838 OX1 PHE P1838 -29.044 -12.242 -43.929 0.00 0.00 O

ATOM 1839 OX1 PHE P1839 -27.498 -10.269 -45.021 0.00 0.00 O

ATOM 1840 SI1 PHE P1840 -30.463 -5.909 -43.250 0.00 0.00 S

ATOM 1841 SI1 PHE P1841 -29.617 -3.184 -42.429 0.00 0.00 S

ATOM 1842 OX1 PHE P1842 -30.321 -4.618 -42.326 0.00 0.00 O

ATOM 1843 OX1 PHE P1843 -29.321 -6.980 -42.965 0.00 0.00 O

ATOM 1844 SI1 PHE P1844 -26.459 -9.131 -44.624 0.00 0.00 S

ATOM 1845 SI1 PHE P1845 -28.892 -7.570 -41.541 0.00 0.00 S

ATOM 1846 SI1 PHE P1846 -26.262 -6.348 -43.822 0.00 0.00 S

ATOM 1847 OX1 PHE P1847 -27.009 -7.667 -44.325 0.00 0.00 O

ATOM 1848 OX1 PHE P1848 -25.132 -9.660 -43.946 0.00 0.00 O

ATOM 1849 OX1 PHE P1849 -26.163 -10.764 -41.748 0.00 0.00 O

ATOM 1850 OX1 PHE P1850 -27.873 -8.753 -41.847 0.00 0.00 O

ATOM 1851 OX1 PHE P1851 -28.145 -6.447 -40.711 0.00 0.00 O

ATOM 1852 OX1 PHE P1852 -25.896 -6.396 -42.254 0.00 0.00 O

ATOM 1853 SI1 PHE P1853 -24.843 -10.270 -42.505 0.00 0.00 S

ATOM 1854 OX1 PHE P1854 -21.823 -12.802 -43.060 0.00 0.00 O

ATOM 1855 OX1 PHE P1855 -23.867 -11.452 -42.941 0.00 0.00 O

ATOM 1856 OX1 PHE P1856 -24.045 -9.178 -41.667 0.00 0.00 O

ATOM 1857 OX1 PHE P1857 -22.806 -8.059 -43.685 0.00 0.00 O

ATOM 1858 SI1 PHE P1858 -21.248 -13.197 -44.500 0.00 0.00 S

ATOM 1859 OX1 PHE P1859 -18.653 -15.538 -43.878 0.00 0.00 O

ATOM 1860 OX1 PHE P1860 -21.069 -14.775 -44.604 0.00 0.00 O

ATOM 1861 OX1 PHE P1861 -20.054 -12.299 -45.074 0.00 0.00 O

ATOM 1862 SI1 PHE P1862 -26.634 -5.972 -40.879 0.00 0.00 S

ATOM 1863 SI1 PHE P1863 -25.390 -3.409 -40.709 0.00 0.00 S

ATOM 1864 OX1 PHE P1864 -26.647 -4.387 -40.691 0.00 0.00 O

ATOM 1865 OX1 PHE P1865 -25.803 -6.640 -39.703 0.00 0.00 O

ATOM 1866 SI1 PHE P1866 -22.685 -8.498 -42.162 0.00 0.00 S

ATOM 1867 SI1 PHE P1867 -21.753 -5.810 -41.503 0.00 0.00 S

ATOM 1868 OX1 PHE P1868 -22.534 -7.184 -41.291 0.00 0.00 O

ATOM 1869 OX1 PHE P1869 -21.480 -9.543 -42.032 0.00 0.00 O

ATOM 1870 OX1 PHE P1870 -21.950 -10.606 -39.708 0.00 0.00 O

ATOM 1871 OX1 PHE P1871 -23.283 -6.422 -38.155 0.00 0.00 O

ATOM 1872 OX1 PHE P1872 -22.103 -5.044 -40.146 0.00 0.00 O

ATOM 1873 SI1 PHE P1873 -18.827 -11.606 -44.313 0.00 0.00 S

ATOM 1874 SI1 PHE P1874 -20.819 -10.184 -40.724 0.00 0.00 S

ATOM 1875 SI1 PHE P1875 -18.308 -9.035 -43.074 0.00 0.00 S

ATOM 1876 OX1 PHE P1876 -19.186 -10.257 -43.575 0.00 0.00 O

ATOM 1877 OX1 PHE P1877 -17.519 -12.415 -43.924 0.00 0.00 O

ATOM 1878 OX1 PHE P1878 -18.147 -13.299 -41.509 0.00 0.00 O

ATOM 1879 OX1 PHE P1879 -19.947 -11.431 -41.228 0.00 0.00 O

ATOM 1880 OX1 PHE P1880 -19.937 -9.086 -39.987 0.00 0.00 O

ATOM 1881 OX1 PHE P1881 -17.764 -9.151 -41.563 0.00 0.00 O

ATOM 1882 SI1 PHE P1882 -16.977 -12.812 -42.482 0.00 0.00 S

ATOM 1883 OX1 PHE P1883 -14.011 -15.046 -41.903 0.00 0.00 O

ATOM 1884 OX1 PHE P1884 -16.168 -14.156 -42.755 0.00 0.00 O

ATOM 1885 OX1 PHE P1885 -16.082 -11.689 -41.746 0.00 0.00 O

ATOM 1886 OX1 PHE P1886 -14.667 -10.646 -43.636 0.00 0.00 O

ATOM 1887 SI1 PHE P1887 -13.289 -15.495 -43.258 0.00 0.00 S

ATOM 1888 OX1 PHE P1888 -11.215 -17.998 -44.435 0.00 0.00 O

ATOM 1889 OX1 PHE P1889 -13.467 -17.008 -43.582 0.00 0.00 O

ATOM 1890 OX1 PHE P1890 -12.167 -14.747 -44.116 0.00 0.00 O

ATOM 1891 SI1 PHE P1891 -22.077 -5.500 -38.613 0.00 0.00 S

ATOM 1892 SI1 PHE P1892 -20.875 -3.017 -38.226 0.00 0.00 S

ATOM 1893 OX1 PHE P1893 -21.851 -4.182 -37.729 0.00 0.00 O

ATOM 1894 OX1 PHE P1894 -20.964 -6.595 -38.331 0.00 0.00 O

ATOM 1895 SI1 PHE P1895 -18.409 -8.672 -40.167 0.00 0.00 S

ATOM 1896 SI1 PHE P1896 -17.361 -5.963 -40.106 0.00 0.00 S

ATOM 1897 OX1 PHE P1897 -18.497 -7.076 -40.053 0.00 0.00 O

ATOM 1898 OX1 PHE P1898 -17.736 -9.392 -38.907 0.00 0.00 O

ATOM 1899 OX1 PHE P1899 -17.000 -10.380 -36.867 0.00 0.00 O

ATOM 1900 OX1 PHE P1900 -16.637 -5.902 -38.688 0.00 0.00 O

ATOM 1901 SI1 PHE P1901 -14.657 -11.015 -42.093 0.00 0.00 S

ATOM 1902 SI1 PHE P1902 -16.356 -9.828 -38.214 0.00 0.00 S

ATOM 1903 SI1 PHE P1903 -13.827 -8.263 -41.311 0.00 0.00 S

ATOM 1904 OX1 PHE P1904 -14.587 -9.673 -41.228 0.00 0.00 O

ATOM 1905 OX1 PHE P1905 -13.394 -11.964 -41.766 0.00 0.00 O

ATOM 1906 OX1 PHE P1906 -13.782 -12.945 -39.381 0.00 0.00 O

ATOM 1907 OX1 PHE P1907 -15.618 -11.019 -38.999 0.00 0.00 O

ATOM 1908 OX1 PHE P1908 -15.344 -8.678 -37.744 0.00 0.00 O

ATOM 1909 OX1 PHE P1909 -14.150 -7.596 -39.893 0.00 0.00 O

ATOM 1910 SI1 PHE P1910 -10.857 -13.933 -43.714 0.00 0.00 S

ATOM 1911 SI1 PHE P1911 -12.673 -12.445 -40.400 0.00 0.00 S

ATOM 1912 SI1 PHE P1912 -10.476 -11.110 -43.115 0.00 0.00 S

ATOM 1913 OX1 PHE P1913 -11.256 -12.462 -43.323 0.00 0.00 O

ATOM 1914 OX1 PHE P1914 -9.490 -14.637 -43.278 0.00 0.00 O

ATOM 1915 OX1 PHE P1915 -9.949 -15.625 -40.908 0.00 0.00 O

ATOM 1916 OX1 PHE P1916 -11.687 -13.669 -40.757 0.00 0.00 O

ATOM 1917 OX1 PHE P1917 -11.771 -11.294 -39.761 0.00 0.00 O

ATOM 1918 OX1 PHE P1918 -9.824 -10.928 -41.670 0.00 0.00 O

ATOM 1919 SI1 PHE P1919 -8.845 -14.969 -41.853 0.00 0.00 S

ATOM 1920 OX1 PHE P1920 -5.815 -17.480 -42.230 0.00 0.00 O

ATOM 1921 OX1 PHE P1921 -7.738 -16.045 -42.194 0.00 0.00 O

ATOM 1922 OX1 PHE P1922 -8.054 -13.724 -41.233 0.00 0.00 O

ATOM 1923 OX1 PHE P1923 -6.764 -12.807 -43.307 0.00 0.00 O

ATOM 1924 SI1 PHE P1924 -5.229 -17.651 -43.703 0.00 0.00 S

ATOM 1925 OX1 PHE P1925 -2.658 -20.378 -43.222 0.00 0.00 O

ATOM 1926 OX1 PHE P1926 -4.887 -19.195 -43.961 0.00 0.00 O

ATOM 1927 OX1 PHE P1927 -4.215 -16.507 -44.203 0.00 0.00 O

ATOM 1928 SI1 PHE P1928 -17.354 -5.907 -37.258 0.00 0.00 S

ATOM 1929 SI1 PHE P1929 -14.014 -8.022 -38.355 0.00 0.00 S

ATOM 1930 SI1 PHE P1930 -12.939 -5.407 -38.077 0.00 0.00 S

ATOM 1931 OX1 PHE P1931 -13.871 -6.613 -37.594 0.00 0.00 O

ATOM 1932 OX1 PHE P1932 -12.688 -8.916 -38.122 0.00 0.00 O

ATOM 1933 OX1 PHE P1933 -12.158 -4.855 -36.821 0.00 0.00 O

ATOM 1934 SI1 PHE P1934 -10.309 -10.753 -40.149 0.00 0.00 S

ATOM 1935 SI1 PHE P1935 -11.754 -9.030 -36.810 0.00 0.00 S

ATOM 1936 SI1 PHE P1936 -9.404 -7.956 -39.849 0.00 0.00 S

ATOM 1937 OX1 PHE P1937 -10.350 -9.227 -39.615 0.00 0.00 O

ATOM 1938 OX1 PHE P1938 -9.118 -11.656 -39.577 0.00 0.00 O

ATOM 1939 OX1 PHE P1939 -9.198 -12.346 -36.949 0.00 0.00 O

ATOM 1940 OX1 PHE P1940 -10.669 -10.211 -36.884 0.00 0.00 O

ATOM 1941 OX1 PHE P1941 -10.873 -7.698 -36.588 0.00 0.00 O

ATOM 1942 OX1 PHE P1942 -8.954 -7.227 -38.495 0.00 0.00 O

ATOM 1943 SI1 PHE P1943 -6.669 -13.070 -41.743 0.00 0.00 S

ATOM 1944 SI1 PHE P1944 -8.334 -11.956 -38.219 0.00 0.00 S

ATOM 1945 SI1 PHE P1945 -5.725 -10.317 -41.169 0.00 0.00 S

ATOM 1946 OX1 PHE P1946 -6.533 -11.682 -40.984 0.00 0.00 O

ATOM 1947 OX1 PHE P1947 -5.414 -14.026 -41.415 0.00 0.00 O

ATOM 1948 OX1 PHE P1948 -5.842 -15.124 -39.085 0.00 0.00 O

ATOM 1949 OX1 PHE P1949 -7.343 -13.120 -38.635 0.00 0.00 O

ATOM 1950 OX1 PHE P1950 -7.451 -10.728 -37.748 0.00 0.00 O

ATOM 1951 OX1 PHE P1951 -5.906 -9.644 -39.731 0.00 0.00 O

ATOM 1952 SI1 PHE P1952 -2.911 -15.891 -43.494 0.00 0.00 S

ATOM 1953 SI1 PHE P1953 -4.737 -14.535 -40.036 0.00 0.00 S

ATOM 1954 SI1 PHE P1954 -2.459 -13.110 -42.731 0.00 0.00 S

ATOM 1955 OX1 PHE P1955 -3.013 -14.592 -42.589 0.00 0.00 O

ATOM 1956 OX1 PHE P1956 -1.671 -16.819 -43.143 0.00 0.00 O

ATOM 1957 OX1 PHE P1957 -2.098 -17.666 -40.613 0.00 0.00 O

ATOM 1958 OX1 PHE P1958 -3.786 -15.775 -40.383 0.00 0.00 O

ATOM 1959 OX1 PHE P1959 -3.864 -13.405 -39.330 0.00 0.00 O

ATOM 1960 OX1 PHE P1960 -2.461 -12.397 -41.315 0.00 0.00 O

ATOM 1961 SI1 PHE P1961 -1.042 -17.162 -41.709 0.00 0.00 S

ATOM 1962 OX1 PHE P1962 1.967 -19.401 -41.457 0.00 0.00 O

ATOM 1963 OX1 PHE P1963 -0.236 -18.495 -42.040 0.00 0.00 O

ATOM 1964 OX1 PHE P1964 -0.145 -15.982 -41.069 0.00 0.00 O

ATOM 1965 OX1 PHE P1965 1.294 -15.116 -43.053 0.00 0.00 O

ATOM 1966 SI1 PHE P1966 2.610 -19.646 -42.917 0.00 0.00 S

ATOM 1967 OX1 PHE P1967 5.274 -22.165 -42.697 0.00 0.00 O

ATOM 1968 OX1 PHE P1968 2.866 -21.194 -43.227 0.00 0.00 O

ATOM 1969 OX1 PHE P1969 3.651 -18.575 -43.508 0.00 0.00 O

ATOM 1970 SI1 PHE P1970 -9.324 -7.406 -36.944 0.00 0.00 S

ATOM 1971 SI1 PHE P1971 -8.537 -4.592 -36.221 0.00 0.00 S

ATOM 1972 OX1 PHE P1972 -8.854 -6.146 -36.069 0.00 0.00 O

ATOM 1973 OX1 PHE P1973 -8.417 -8.578 -36.346 0.00 0.00 O

ATOM 1974 SI1 PHE P1974 -6.039 -10.159 -38.223 0.00 0.00 S

ATOM 1975 SI1 PHE P1975 -7.921 -8.700 -34.830 0.00 0.00 S

ATOM 1976 SI1 PHE P1976 -5.150 -7.482 -37.543 0.00 0.00 S

ATOM 1977 OX1 PHE P1977 -5.961 -8.833 -37.348 0.00 0.00 O

ATOM 1978 OX1 PHE P1978 -4.858 -11.179 -37.866 0.00 0.00 O

ATOM 1979 OX1 PHE P1979 -4.914 -11.549 -35.224 0.00 0.00 O

ATOM 1980 OX1 PHE P1980 -7.220 -10.075 -34.481 0.00 0.00 O

ATOM 1981 OX1 PHE P1981 -7.069 -7.401 -34.441 0.00 0.00 O

ATOM 1982 OX1 PHE P1982 -4.753 -7.257 -36.010 0.00 0.00 O

ATOM 1983 SI1 PHE P1983 -2.464 -12.773 -39.787 0.00 0.00 S

ATOM 1984 SI1 PHE P1984 -4.021 -11.273 -36.505 0.00 0.00 S

ATOM 1985 SI1 PHE P1985 -1.397 -10.122 -39.272 0.00 0.00 S

ATOM 1986 OX1 PHE P1986 -2.429 -11.323 -39.115 0.00 0.00 O

ATOM 1987 OX1 PHE P1987 -1.191 -13.687 -39.449 0.00 0.00 O

ATOM 1988 OX1 PHE P1988 -1.316 -14.373 -36.846 0.00 0.00 O

ATOM 1989 OX1 PHE P1989 -2.825 -12.305 -36.616 0.00 0.00 O

ATOM 1990 OX1 PHE P1990 -3.270 -9.887 -36.363 0.00 0.00 O

ATOM 1991 OX1 PHE P1991 -0.910 -9.924 -37.739 0.00 0.00 O

ATOM 1992 SI1 PHE P1992 1.275 -15.339 -41.481 0.00 0.00 S

ATOM 1993 SI1 PHE P1993 -0.409 -13.987 -38.078 0.00 0.00 S

ATOM 1994 SI1 PHE P1994 2.183 -12.548 -40.934 0.00 0.00 S

ATOM 1995 OX1 PHE P1995 1.415 -13.940 -40.728 0.00 0.00 O

ATOM 1996 OX1 PHE P1996 2.487 -16.285 -41.031 0.00 0.00 O

ATOM 1997 OX1 PHE P1997 2.191 -17.149 -38.460 0.00 0.00 O

ATOM 1998 OX1 PHE P1998 0.535 -15.240 -38.367 0.00 0.00 O

ATOM 1999 OX1 PHE P1999 0.443 -12.741 -37.563 0.00 0.00 O

ATOM 2000 OX1 PHE P2000 2.023 -11.800 -39.538 0.00 0.00 O

ATOM 2001 SI1 PHE P2001 5.030 -18.013 -42.911 0.00 0.00 S

ATOM 2002 SI1 PHE P2002 3.168 -16.669 -39.617 0.00 0.00 S

ATOM 2003 SI1 PHE P2003 5.536 -15.211 -42.342 0.00 0.00 S

ATOM 2004 OX1 PHE P2004 5.024 -16.681 -42.062 0.00 0.00 O

ATOM 2005 OX1 PHE P2005 6.224 -18.985 -42.521 0.00 0.00 O

ATOM 2006 OX1 PHE P2006 5.853 -19.678 -39.918 0.00 0.00 O

ATOM 2007 OX1 PHE P2007 3.957 -18.007 -39.984 0.00 0.00 O

ATOM 2008 OX1 PHE P2008 4.122 -15.525 -39.013 0.00 0.00 O

ATOM 2009 OX1 PHE P2009 5.540 -14.452 -40.958 0.00 0.00 O

ATOM 2010 SI1 PHE P2010 6.888 -19.246 -41.084 0.00 0.00 S

ATOM 2011 OX1 PHE P2011 9.909 -21.444 -40.569 0.00 0.00 O

ATOM 2012 OX1 PHE P2012 7.678 -20.598 -41.387 0.00 0.00 O

ATOM 2013 OX1 PHE P2013 7.849 -18.069 -40.541 0.00 0.00 O

ATOM 2014 OX1 PHE P2014 9.321 -17.326 -42.554 0.00 0.00 O

ATOM 2015 SI1 PHE P2015 10.611 -21.735 -41.966 0.00 0.00 S

ATOM 2016 OX1 PHE P2016 12.787 -24.267 -43.096 0.00 0.00 O

ATOM 2017 OX1 PHE P2017 10.526 -23.246 -42.371 0.00 0.00 O

ATOM 2018 OX1 PHE P2018 11.537 -20.722 -42.792 0.00 0.00 O

ATOM 2019 SI1 PHE P2019 -5.570 -6.871 -34.672 0.00 0.00 S

ATOM 2020 SI1 PHE P2020 -4.518 -4.230 -34.743 0.00 0.00 S

ATOM 2021 OX1 PHE P2021 -5.706 -5.284 -34.641 0.00 0.00 O

ATOM 2022 SI1 PHE P2022 -1.708 -9.554 -36.363 0.00 0.00 S

ATOM 2023 SI1 PHE P2023 -0.726 -6.851 -36.161 0.00 0.00 S

ATOM 2024 OX1 PHE P2024 -1.850 -7.969 -36.174 0.00 0.00 O

ATOM 2025 OX1 PHE P2025 -1.079 -10.356 -35.101 0.00 0.00 O

ATOM 2026 OX1 PHE P2026 -0.214 -6.496 -34.702 0.00 0.00 O

ATOM 2027 SI1 PHE P2027 1.859 -12.137 -37.995 0.00 0.00 S

ATOM 2028 SI1 PHE P2028 0.398 -10.408 -34.466 0.00 0.00 S

ATOM 2029 SI1 PHE P2029 2.943 -9.514 -37.568 0.00 0.00 S

ATOM 2030 OX1 PHE P2030 1.864 -10.667 -37.357 0.00 0.00 O

ATOM 2031 OX1 PHE P2031 3.098 -13.047 -37.546 0.00 0.00 O

ATOM 2032 OX1 PHE P2032 2.979 -13.700 -34.910 0.00 0.00 O

ATOM 2033 OX1 PHE P2033 1.425 -11.570 -34.868 0.00 0.00 O

ATOM 2034 OX1 PHE P2034 1.084 -9.057 -34.907 0.00 0.00 O

ATOM 2035 OX1 PHE P2035 3.467 -9.193 -36.090 0.00 0.00 O

ATOM 2036 SI1 PHE P2036 5.529 -14.865 -39.441 0.00 0.00 S

ATOM 2037 SI1 PHE P2037 3.871 -13.334 -36.166 0.00 0.00 S

ATOM 2038 SI1 PHE P2038 6.483 -12.174 -39.027 0.00 0.00 S

ATOM 2039 OX1 PHE P2039 5.580 -13.444 -38.715 0.00 0.00 O

ATOM 2040 OX1 PHE P2040 6.834 -15.727 -39.070 0.00 0.00 O

ATOM 2041 OX1 PHE P2041 6.560 -16.476 -36.548 0.00 0.00 O

ATOM 2042 OX1 PHE P2042 4.741 -14.617 -36.525 0.00 0.00 O

ATOM 2043 OX1 PHE P2043 4.806 -12.124 -35.692 0.00 0.00 O

ATOM 2044 OX1 PHE P2044 6.587 -11.351 -37.653 0.00 0.00 O

ATOM 2045 SI1 PHE P2045 9.296 -17.496 -40.976 0.00 0.00 S

ATOM 2046 SI1 PHE P2046 7.574 -16.055 -37.673 0.00 0.00 S

ATOM 2047 SI1 PHE P2047 10.208 -14.696 -40.489 0.00 0.00 S

ATOM 2048 OX1 PHE P2048 9.454 -16.085 -40.257 0.00 0.00 O

ATOM 2049 OX1 PHE P2049 10.521 -18.408 -40.475 0.00 0.00 O

ATOM 2050 OX1 PHE P2050 10.105 -19.262 -37.994 0.00 0.00 O

ATOM 2051 OX1 PHE P2051 8.423 -17.373 -37.955 0.00 0.00 O

ATOM 2052 OX1 PHE P2052 8.484 -14.871 -37.109 0.00 0.00 O

ATOM 2053 OX1 PHE P2053 10.070 -13.962 -39.085 0.00 0.00 O

ATOM 2054 SI1 PHE P2054 12.967 -20.087 -42.458 0.00 0.00 S

ATOM 2055 SI1 PHE P2055 11.184 -18.739 -39.035 0.00 0.00 S

ATOM 2056 SI1 PHE P2056 13.503 -17.319 -41.806 0.00 0.00 S

ATOM 2057 OX1 PHE P2057 12.966 -18.783 -41.572 0.00 0.00 O

ATOM 2058 OX1 PHE P2058 14.223 -21.025 -42.194 0.00 0.00 O

ATOM 2059 OX1 PHE P2059 13.819 -21.878 -39.683 0.00 0.00 O

ATOM 2060 OX1 PHE P2060 12.186 -19.940 -39.368 0.00 0.00 O

ATOM 2061 OX1 PHE P2061 12.033 -17.531 -38.415 0.00 0.00 O

ATOM 2062 OX1 PHE P2062 13.405 -16.554 -40.424 0.00 0.00 O

ATOM 2063 SI1 PHE P2063 14.881 -21.396 -40.783 0.00 0.00 S

ATOM 2064 OX1 PHE P2064 17.883 -23.682 -40.267 0.00 0.00 O

ATOM 2065 OX1 PHE P2065 15.702 -22.735 -41.078 0.00 0.00 O

ATOM 2066 OX1 PHE P2066 15.790 -20.222 -40.159 0.00 0.00 O

ATOM 2067 OX1 PHE P2067 17.186 -19.326 -42.159 0.00 0.00 O

ATOM 2068 SI1 PHE P2068 18.560 -24.181 -41.616 0.00 0.00 S

ATOM 2069 OX1 PHE P2069 20.640 -26.694 -42.624 0.00 0.00 O

ATOM 2070 OX1 PHE P2070 18.376 -25.710 -41.866 0.00 0.00 O

ATOM 2071 OX1 PHE P2071 19.645 -23.423 -42.511 0.00 0.00 O

ATOM 2072 SI1 PHE P2072 2.603 -8.609 -34.869 0.00 0.00 S

ATOM 2073 SI1 PHE P2073 3.639 -6.098 -34.330 0.00 0.00 S

ATOM 2074 OX1 PHE P2074 2.526 -7.023 -34.958 0.00 0.00 O

ATOM 2075 OX1 PHE P2075 3.257 -9.071 -33.493 0.00 0.00 O

ATOM 2076 SI1 PHE P2076 6.281 -11.628 -36.092 0.00 0.00 S

ATOM 2077 SI1 PHE P2077 7.294 -8.939 -35.737 0.00 0.00 S

ATOM 2078 OX1 PHE P2078 6.344 -10.179 -35.364 0.00 0.00 O

ATOM 2079 OX1 PHE P2079 7.430 -12.632 -35.579 0.00 0.00 O

ATOM 2080 OX1 PHE P2080 7.937 -8.291 -34.439 0.00 0.00 O

ATOM 2081 SI1 PHE P2081 9.918 -14.298 -37.534 0.00 0.00 S

ATOM 2082 SI1 PHE P2082 8.387 -12.483 -34.298 0.00 0.00 S

ATOM 2083 SI1 PHE P2083 10.921 -11.596 -37.247 0.00 0.00 S

ATOM 2084 OX1 PHE P2084 9.907 -12.798 -36.965 0.00 0.00 O

ATOM 2085 OX1 PHE P2085 11.143 -15.210 -37.007 0.00 0.00 O

ATOM 2086 OX1 PHE P2086 11.172 -15.556 -34.277 0.00 0.00 O

ATOM 2087 OX1 PHE P2087 9.596 -13.519 -34.282 0.00 0.00 O

ATOM 2088 OX1 PHE P2088 9.100 -11.069 -34.436 0.00 0.00 O

ATOM 2089 OX1 PHE P2089 11.462 -11.212 -35.790 0.00 0.00 O

ATOM 2090 SI1 PHE P2090 13.435 -16.904 -38.888 0.00 0.00 S

ATOM 2091 SI1 PHE P2091 11.979 -15.268 -35.622 0.00 0.00 S

ATOM 2092 SI1 PHE P2092 14.438 -14.194 -38.688 0.00 0.00 S

ATOM 2093 OX1 PHE P2093 13.564 -15.451 -38.234 0.00 0.00 O

ATOM 2094 OX1 PHE P2094 14.685 -17.846 -38.535 0.00 0.00 O

ATOM 2095 OX1 PHE P2095 14.636 -18.485 -35.867 0.00 0.00 O

ATOM 2096 OX1 PHE P2096 13.158 -16.342 -35.703 0.00 0.00 O

ATOM 2097 OX1 PHE P2097 12.763 -13.901 -35.390 0.00 0.00 O

ATOM 2098 OX1 PHE P2098 14.595 -13.266 -37.388 0.00 0.00 O

ATOM 2099 SI1 PHE P2099 17.202 -19.578 -40.592 0.00 0.00 S

ATOM 2100 SI1 PHE P2100 15.474 -18.195 -37.172 0.00 0.00 S

ATOM 2101 SI1 PHE P2101 18.093 -16.801 -39.973 0.00 0.00 S

ATOM 2102 OX1 PHE P2102 17.326 -18.196 -39.809 0.00 0.00 O

ATOM 2103 OX1 PHE P2103 18.437 -20.515 -40.191 0.00 0.00 O

ATOM 2104 OX1 PHE P2104 18.114 -21.346 -37.667 0.00 0.00 O

ATOM 2105 OX1 PHE P2105 16.307 -19.506 -37.551 0.00 0.00 O

ATOM 2106 OX1 PHE P2106 16.378 -17.008 -36.622 0.00 0.00 O

ATOM 2107 OX1 PHE P2107 17.832 -16.055 -38.610 0.00 0.00 O

ATOM 2108 SI1 PHE P2108 20.918 -22.549 -42.117 0.00 0.00 S

ATOM 2109 SI1 PHE P2109 19.140 -20.909 -38.794 0.00 0.00 S

ATOM 2110 SI1 PHE P2110 21.365 -19.725 -41.631 0.00 0.00 S

ATOM 2111 OX1 PHE P2111 20.524 -21.047 -41.833 0.00 0.00 O

ATOM 2112 OX1 PHE P2112 22.270 -23.221 -41.599 0.00 0.00 O

ATOM 2113 OX1 PHE P2113 21.719 -24.098 -39.193 0.00 0.00 O

ATOM 2114 OX1 PHE P2114 20.009 -22.190 -39.165 0.00 0.00 O

ATOM 2115 OX1 PHE P2115 20.097 -19.784 -38.207 0.00 0.00 O

ATOM 2116 OX1 PHE P2116 21.995 -19.608 -40.167 0.00 0.00 O

ATOM 2117 SI1 PHE P2117 22.845 -23.514 -40.137 0.00 0.00 S

ATOM 2118 OX1 PHE P2118 25.903 -25.886 -39.880 0.00 0.00 O

ATOM 2119 OX1 PHE P2119 23.838 -24.719 -40.369 0.00 0.00 O

ATOM 2120 OX1 PHE P2120 23.643 -22.270 -39.518 0.00 0.00 O

ATOM 2121 OX1 PHE P2121 24.867 -21.465 -41.652 0.00 0.00 O

ATOM 2122 SI1 PHE P2122 26.548 -26.183 -41.295 0.00 0.00 S

ATOM 2123 OX1 PHE P2123 29.056 -28.688 -41.870 0.00 0.00 O

ATOM 2124 OX1 PHE P2124 26.688 -27.735 -41.497 0.00 0.00 O

ATOM 2125 OX1 PHE P2125 27.443 -25.125 -42.108 0.00 0.00 O

ATOM 2126 SI1 PHE P2126 10.636 -10.637 -34.532 0.00 0.00 S

ATOM 2127 SI1 PHE P2127 11.710 -8.103 -34.024 0.00 0.00 S

ATOM 2128 OX1 PHE P2128 10.582 -9.048 -34.604 0.00 0.00 O

ATOM 2129 OX1 PHE P2129 11.355 -11.122 -33.197 0.00 0.00 O

ATOM 2130 SI1 PHE P2130 14.240 -13.447 -35.824 0.00 0.00 S

ATOM 2131 SI1 PHE P2131 15.329 -10.836 -35.437 0.00 0.00 S

ATOM 2132 OX1 PHE P2132 14.326 -12.018 -35.085 0.00 0.00 O

ATOM 2133 OX1 PHE P2133 15.369 -14.451 -35.302 0.00 0.00 O

ATOM 2134 OX1 PHE P2134 15.906 -10.145 -34.127 0.00 0.00 O

ATOM 2135 SI1 PHE P2135 17.781 -16.426 -37.081 0.00 0.00 S

ATOM 2136 SI1 PHE P2136 16.114 -14.404 -33.897 0.00 0.00 S

ATOM 2137 SI1 PHE P2137 18.798 -13.722 -36.841 0.00 0.00 S

ATOM 2138 OX1 PHE P2138 17.825 -14.940 -36.471 0.00 0.00 O

ATOM 2139 OX1 PHE P2139 18.994 -17.418 -36.699 0.00 0.00 O

ATOM 2140 OX1 PHE P2140 19.263 -17.345 -34.046 0.00 0.00 O

ATOM 2141 OX1 PHE P2141 16.857 -15.752 -33.519 0.00 0.00 O

ATOM 2142 OX1 PHE P2142 17.032 -13.099 -33.822 0.00 0.00 O

ATOM 2143 OX1 PHE P2143 19.338 -13.307 -35.396 0.00 0.00 O

ATOM 2144 SI1 PHE P2144 21.535 -19.256 -38.667 0.00 0.00 S

ATOM 2145 SI1 PHE P2145 19.995 -17.324 -35.452 0.00 0.00 S

ATOM 2146 SI1 PHE P2146 22.387 -16.455 -38.591 0.00 0.00 S

ATOM 2147 OX1 PHE P2147 21.359 -17.667 -38.433 0.00 0.00 O

ATOM 2148 OX1 PHE P2148 22.791 -19.994 -37.970 0.00 0.00 O

ATOM 2149 OX1 PHE P2149 22.776 -20.401 -35.295 0.00 0.00 O

ATOM 2150 OX1 PHE P2150 21.191 -18.376 -35.437 0.00 0.00 O

ATOM 2151 OX1 PHE P2151 20.686 -15.922 -35.651 0.00 0.00 O

ATOM 2152 OX1 PHE P2152 22.969 -16.135 -37.132 0.00 0.00 O

ATOM 2153 SI1 PHE P2153 25.007 -21.628 -40.078 0.00 0.00 S

ATOM 2154 SI1 PHE P2154 23.617 -20.031 -36.591 0.00 0.00 S

ATOM 2155 SI1 PHE P2155 25.903 -18.808 -39.773 0.00 0.00 S

ATOM 2156 OX1 PHE P2156 25.144 -20.176 -39.430 0.00 0.00 O

ATOM 2157 OX1 PHE P2157 26.264 -22.579 -39.745 0.00 0.00 O

ATOM 2158 OX1 PHE P2158 26.247 -23.286 -37.101 0.00 0.00 O

ATOM 2159 OX1 PHE P2159 24.857 -21.030 -36.759 0.00 0.00 O

ATOM 2160 OX1 PHE P2160 24.307 -18.640 -36.286 0.00 0.00 O

ATOM 2161 OX1 PHE P2161 25.696 -17.890 -38.473 0.00 0.00 O

ATOM 2162 SI1 PHE P2162 28.907 -24.533 -41.826 0.00 0.00 S

ATOM 2163 SI1 PHE P2163 27.090 -22.927 -38.401 0.00 0.00 S

ATOM 2164 SI1 PHE P2164 29.258 -21.724 -41.296 0.00 0.00 S

ATOM 2165 OX1 PHE P2165 29.110 -23.247 -40.934 0.00 0.00 O

ATOM 2166 OX1 PHE P2166 30.131 -25.500 -41.562 0.00 0.00 O

ATOM 2167 OX1 PHE P2167 29.625 -26.259 -39.108 0.00 0.00 O

ATOM 2168 OX1 PHE P2168 28.005 -24.184 -38.814 0.00 0.00 O

ATOM 2169 OX1 PHE P2169 27.968 -21.683 -37.940 0.00 0.00 O

ATOM 2170 OX1 PHE P2170 29.653 -20.936 -39.980 0.00 0.00 O

ATOM 2171 SI1 PHE P2171 30.761 -25.797 -40.124 0.00 0.00 S

ATOM 2172 OX1 PHE P2172 33.867 -28.132 -39.910 0.00 0.00 O

ATOM 2173 OX1 PHE P2173 31.713 -27.021 -40.426 0.00 0.00 O

ATOM 2174 OX1 PHE P2174 31.607 -24.590 -39.476 0.00 0.00 O

ATOM 2175 OX1 PHE P2175 32.815 -23.684 -41.552 0.00 0.00 O

ATOM 2176 SI1 PHE P2176 34.262 -28.758 -41.317 0.00 0.00 S

ATOM 2177 OX1 PHE P2177 36.601 -30.946 -41.855 0.00 0.00 O

ATOM 2178 OX1 PHE P2178 34.249 -30.334 -41.240 0.00 0.00 O

ATOM 2179 OX1 PHE P2179 35.391 -28.094 -42.241 0.00 0.00 O

ATOM 2180 SI1 PHE P2180 18.565 -12.715 -34.113 0.00 0.00 S

ATOM 2181 SI1 PHE P2181 19.579 -10.071 -33.836 0.00 0.00 S

ATOM 2182 OX1 PHE P2182 18.482 -11.132 -34.253 0.00 0.00 O

ATOM 2183 SI1 PHE P2183 22.207 -15.518 -35.849 0.00 0.00 S

ATOM 2184 SI1 PHE P2184 23.167 -12.954 -35.312 0.00 0.00 S

ATOM 2185 OX1 PHE P2185 22.088 -13.932 -35.915 0.00 0.00 O

ATOM 2186 OX1 PHE P2186 23.002 -15.977 -34.555 0.00 0.00 O

ATOM 2187 SI1 PHE P2187 25.672 -18.090 -36.891 0.00 0.00 S

ATOM 2188 SI1 PHE P2188 26.916 -15.594 -36.723 0.00 0.00 S

ATOM 2189 OX1 PHE P2189 25.849 -16.652 -36.216 0.00 0.00 O

ATOM 2190 OX1 PHE P2190 26.912 -19.035 -36.557 0.00 0.00 O

ATOM 2191 OX1 PHE P2191 27.702 -15.016 -35.484 0.00 0.00 O

ATOM 2192 SI1 PHE P2192 29.382 -21.128 -38.426 0.00 0.00 S

ATOM 2193 SI1 PHE P2193 27.646 -19.173 -35.157 0.00 0.00 S

ATOM 2194 SI1 PHE P2194 30.315 -18.413 -38.222 0.00 0.00 S

ATOM 2195 OX1 PHE P2195 29.419 -19.660 -37.797 0.00 0.00 O

ATOM 2196 OX1 PHE P2196 30.609 -22.053 -37.981 0.00 0.00 O

ATOM 2197 OX1 PHE P2197 28.240 -20.628 -34.950 0.00 0.00 O

ATOM 2198 OX1 PHE P2198 28.718 -18.015 -34.905 0.00 0.00 O

ATOM 2199 OX1 PHE P2199 30.685 -17.657 -36.859 0.00 0.00 O

ATOM 2200 SI1 PHE P2200 32.981 -23.938 -39.992 0.00 0.00 S

ATOM 2201 SI1 PHE P2201 31.313 -22.219 -36.560 0.00 0.00 S

ATOM 2202 SI1 PHE P2202 33.841 -21.114 -39.610 0.00 0.00 S

ATOM 2203 OX1 PHE P2203 33.156 -22.526 -39.262 0.00 0.00 O

ATOM 2204 OX1 PHE P2204 34.230 -24.925 -39.731 0.00 0.00 O

ATOM 2205 OX1 PHE P2205 34.231 -25.559 -37.144 0.00 0.00 O

ATOM 2206 OX1 PHE P2206 32.107 -23.587 -36.456 0.00 0.00 O

ATOM 2207 OX1 PHE P2207 32.233 -20.977 -36.156 0.00 0.00 O

ATOM 2208 OX1 PHE P2208 33.687 -20.259 -38.280 0.00 0.00 O

ATOM 2209 SI1 PHE P2209 36.642 -27.182 -41.836 0.00 0.00 S

ATOM 2210 SI1 PHE P2210 35.112 -25.308 -38.432 0.00 0.00 S

ATOM 2211 SI1 PHE P2211 36.981 -24.295 -41.434 0.00 0.00 S

ATOM 2212 OX1 PHE P2212 36.217 -25.686 -41.509 0.00 0.00 O

ATOM 2213 OX1 PHE P2213 38.067 -27.817 -41.473 0.00 0.00 O

ATOM 2214 OX1 PHE P2214 38.045 -28.300 -38.896 0.00 0.00 O

ATOM 2215 OX1 PHE P2215 35.995 -26.599 -38.824 0.00 0.00 O

ATOM 2216 OX1 PHE P2216 36.025 -24.086 -37.994 0.00 0.00 O

ATOM 2217 OX1 PHE P2217 37.776 -24.050 -40.061 0.00 0.00 O

ATOM 2218 SI1 PHE P2218 38.928 -27.783 -40.122 0.00 0.00 S

ATOM 2219 OX1 PHE P2219 41.823 -30.768 -40.583 0.00 0.00 O

ATOM 2220 OX1 PHE P2220 40.190 -28.755 -40.299 0.00 0.00 O

ATOM 2221 OX1 PHE P2221 39.541 -26.337 -39.802 0.00 0.00 O

ATOM 2222 OX1 PHE P2222 40.386 -25.344 -42.087 0.00 0.00 O

ATOM 2223 SI1 PHE P2223 42.304 -30.648 -42.095 0.00 0.00 S

ATOM 2224 OX1 PHE P2224 45.255 -33.123 -43.480 0.00 0.00 O

ATOM 2225 OX1 PHE P2225 43.173 -31.815 -42.744 0.00 0.00 O

ATOM 2226 OX1 PHE P2226 42.402 -29.204 -42.750 0.00 0.00 O

ATOM 2227 SI1 PHE P2227 30.263 -17.927 -35.324 0.00 0.00 S

ATOM 2228 SI1 PHE P2228 33.652 -20.478 -36.709 0.00 0.00 S

ATOM 2229 SI1 PHE P2229 34.853 -17.894 -36.822 0.00 0.00 S

ATOM 2230 OX1 PHE P2230 33.938 -19.010 -36.111 0.00 0.00 O

ATOM 2231 SI1 PHE P2231 37.383 -23.516 -38.590 0.00 0.00 S

ATOM 2232 SI1 PHE P2232 38.306 -20.807 -38.900 0.00 0.00 S

ATOM 2233 OX1 PHE P2233 37.229 -21.921 -38.512 0.00 0.00 O

ATOM 2234 OX1 PHE P2234 38.594 -23.954 -37.662 0.00 0.00 O

ATOM 2235 SI1 PHE P2235 40.736 -25.580 -40.558 0.00 0.00 S

ATOM 2236 SI1 PHE P2236 42.022 -23.007 -40.231 0.00 0.00 S

ATOM 2237 OX1 PHE P2237 41.034 -24.197 -39.813 0.00 0.00 O

ATOM 2238 OX1 PHE P2238 42.043 -26.478 -40.470 0.00 0.00 O

ATOM 2239 SI1 PHE P2239 43.793 -28.534 -43.114 0.00 0.00 S

ATOM 2240 SI1 PHE P2240 45.199 -26.254 -42.262 0.00 0.00 S

ATOM 2241 OX1 PHE P2241 43.952 -26.969 -42.940 0.00 0.00 O

ATOM 2242 OX1 PHE P2242 44.780 -29.273 -44.114 0.00 0.00 O

ATOM 2243 SI1 PHE P2243 -36.669 -11.253 -44.771 0.00 0.00 S

ATOM 2244 OX1 PHE P2244 -31.517 -16.724 -42.512 0.00 0.00 O

ATOM 2245 OX1 PHE P2245 -33.554 -11.183 -46.255 0.00 0.00 O

ATOM 2246 SI1 PHE P2246 -30.981 -17.455 -43.815 0.00 0.00 S

ATOM 2247 OX1 PHE P2247 -28.515 -19.837 -43.556 0.00 0.00 O

ATOM 2248 OX1 PHE P2248 -30.979 -19.048 -43.845 0.00 0.00 O

ATOM 2249 OX1 PHE P2249 -30.123 -16.603 -44.849 0.00 0.00 O

ATOM 2250 SI1 PHE P2250 -32.313 -11.946 -45.626 0.00 0.00 S

ATOM 2251 SI1 PHE P2251 -31.488 -9.990 -43.709 0.00 0.00 S

ATOM 2252 OX1 PHE P2252 -32.016 -11.428 -44.153 0.00 0.00 O

ATOM 2253 SI1 PHE P2253 -28.781 -15.819 -44.506 0.00 0.00 S

ATOM 2254 SI1 PHE P2254 -28.098 -13.355 -43.285 0.00 0.00 S

ATOM 2255 OX1 PHE P2255 -28.901 -14.721 -43.363 0.00 0.00 O

ATOM 2256 OX1 PHE P2256 -27.430 -16.664 -44.476 0.00 0.00 O

ATOM 2257 OX1 PHE P2257 -27.786 -17.524 -42.046 0.00 0.00 O

ATOM 2258 OX1 PHE P2258 -27.715 -13.170 -41.744 0.00 0.00 O

ATOM 2259 SI1 PHE P2259 -26.698 -17.189 -43.158 0.00 0.00 S

ATOM 2260 OX1 PHE P2260 -23.586 -19.395 -43.279 0.00 0.00 O

ATOM 2261 OX1 PHE P2261 -25.941 -18.524 -43.598 0.00 0.00 O

ATOM 2262 OX1 PHE P2262 -25.752 -16.066 -42.511 0.00 0.00 O

ATOM 2263 OX1 PHE P2263 -24.098 -15.657 -44.486 0.00 0.00 O

ATOM 2264 SI1 PHE P2264 -22.969 -19.796 -44.701 0.00 0.00 S

ATOM 2265 OX1 PHE P2265 -20.329 -22.146 -44.131 0.00 0.00 O

ATOM 2266 OX1 PHE P2266 -22.747 -21.371 -44.876 0.00 0.00 O

ATOM 2267 OX1 PHE P2267 -21.857 -18.798 -45.267 0.00 0.00 O

ATOM 2268 SI1 PHE P2268 -28.561 -12.500 -40.570 0.00 0.00 S

ATOM 2269 SI1 PHE P2269 -27.294 -9.934 -40.960 0.00 0.00 S

ATOM 2270 SI1 PHE P2270 -24.297 -15.535 -42.915 0.00 0.00 S

ATOM 2271 SI1 PHE P2271 -23.261 -12.802 -42.354 0.00 0.00 S

ATOM 2272 OX1 PHE P2272 -24.321 -13.969 -42.611 0.00 0.00 O

ATOM 2273 OX1 PHE P2273 -23.159 -16.451 -42.257 0.00 0.00 O

ATOM 2274 OX1 PHE P2274 -23.736 -17.280 -39.793 0.00 0.00 O

ATOM 2275 OX1 PHE P2275 -22.955 -12.881 -40.792 0.00 0.00 O

ATOM 2276 SI1 PHE P2276 -20.566 -18.252 -44.502 0.00 0.00 S

ATOM 2277 SI1 PHE P2277 -22.591 -16.902 -40.822 0.00 0.00 S

ATOM 2278 SI1 PHE P2278 -20.201 -15.585 -43.542 0.00 0.00 S

ATOM 2279 OX1 PHE P2279 -20.738 -17.068 -43.471 0.00 0.00 O

ATOM 2280 OX1 PHE P2280 -19.377 -19.237 -44.152 0.00 0.00 O

ATOM 2281 OX1 PHE P2281 -20.039 -20.093 -41.704 0.00 0.00 O

ATOM 2282 OX1 PHE P2282 -21.743 -18.168 -41.309 0.00 0.00 O

ATOM 2283 OX1 PHE P2283 -21.688 -15.802 -40.097 0.00 0.00 O

ATOM 2284 OX1 PHE P2284 -20.470 -14.847 -42.157 0.00 0.00 O

ATOM 2285 SI1 PHE P2285 -18.867 -19.623 -42.689 0.00 0.00 S

ATOM 2286 OX1 PHE P2286 -15.883 -21.963 -42.287 0.00 0.00 O

ATOM 2287 OX1 PHE P2287 -18.041 -20.967 -42.922 0.00 0.00 O

ATOM 2288 OX1 PHE P2288 -17.993 -18.464 -41.994 0.00 0.00 O

ATOM 2289 OX1 PHE P2289 -16.503 -17.572 -43.922 0.00 0.00 O

ATOM 2290 SI1 PHE P2290 -15.320 -22.455 -43.722 0.00 0.00 S

ATOM 2291 OX1 PHE P2291 -12.652 -24.813 -43.517 0.00 0.00 O

ATOM 2292 OX1 PHE P2292 -15.166 -24.029 -43.893 0.00 0.00 O

ATOM 2293 OX1 PHE P2293 -14.221 -21.608 -44.505 0.00 0.00 O

ATOM 2294 SI1 PHE P2294 -24.007 -12.967 -39.596 0.00 0.00 S

ATOM 2295 SI1 PHE P2295 -20.320 -15.167 -40.612 0.00 0.00 S

ATOM 2296 SI1 PHE P2296 -19.152 -12.598 -40.483 0.00 0.00 S

ATOM 2297 OX1 PHE P2297 -20.130 -13.735 -39.927 0.00 0.00 O

ATOM 2298 OX1 PHE P2298 -19.047 -16.093 -40.332 0.00 0.00 O

ATOM 2299 OX1 PHE P2299 -18.341 -12.055 -39.244 0.00 0.00 O

ATOM 2300 SI1 PHE P2300 -16.545 -17.869 -42.362 0.00 0.00 S

ATOM 2301 SI1 PHE P2301 -18.380 -16.512 -38.936 0.00 0.00 S

ATOM 2302 SI1 PHE P2302 -15.587 -15.104 -41.617 0.00 0.00 S

ATOM 2303 OX1 PHE P2303 -16.376 -16.504 -41.536 0.00 0.00 O

ATOM 2304 OX1 PHE P2304 -15.413 -18.935 -41.973 0.00 0.00 O

ATOM 2305 OX1 PHE P2305 -15.676 -19.684 -39.379 0.00 0.00 O

ATOM 2306 OX1 PHE P2306 -17.466 -17.772 -39.316 0.00 0.00 O

ATOM 2307 OX1 PHE P2307 -17.516 -15.335 -38.280 0.00 0.00 O

ATOM 2308 OX1 PHE P2308 -15.957 -14.379 -40.238 0.00 0.00 O

ATOM 2309 SI1 PHE P2309 -12.938 -20.845 -43.950 0.00 0.00 S

ATOM 2310 SI1 PHE P2310 -14.705 -19.313 -40.578 0.00 0.00 S

ATOM 2311 SI1 PHE P2311 -12.357 -18.094 -43.335 0.00 0.00 S

ATOM 2312 OX1 PHE P2312 -13.265 -19.362 -43.550 0.00 0.00 O

ATOM 2313 OX1 PHE P2313 -11.611 -21.593 -43.494 0.00 0.00 O

ATOM 2314 OX1 PHE P2314 -11.990 -22.364 -40.961 0.00 0.00 O

ATOM 2315 OX1 PHE P2315 -13.852 -20.645 -40.870 0.00 0.00 O

ATOM 2316 OX1 PHE P2316 -13.841 -18.046 -40.181 0.00 0.00 O

ATOM 2317 OX1 PHE P2317 -11.701 -18.004 -41.883 0.00 0.00 O

ATOM 2318 SI1 PHE P2318 -10.947 -21.844 -42.066 0.00 0.00 S

ATOM 2319 OX1 PHE P2319 -7.904 -24.094 -41.884 0.00 0.00 O

ATOM 2320 OX1 PHE P2320 -10.135 -23.181 -42.384 0.00 0.00 O

ATOM 2321 OX1 PHE P2321 -10.011 -20.623 -41.537 0.00 0.00 O

ATOM 2322 OX1 PHE P2322 -8.415 -20.310 -43.561 0.00 0.00 O

ATOM 2323 SI1 PHE P2323 -7.357 -24.419 -43.371 0.00 0.00 S

ATOM 2324 OX1 PHE P2324 -4.625 -26.745 -43.404 0.00 0.00 O

ATOM 2325 OX1 PHE P2325 -7.155 -25.979 -43.675 0.00 0.00 O

ATOM 2326 OX1 PHE P2326 -6.297 -23.416 -44.033 0.00 0.00 O

ATOM 2327 SI1 PHE P2327 -16.075 -14.785 -38.700 0.00 0.00 S

ATOM 2328 SI1 PHE P2328 -14.795 -12.237 -38.361 0.00 0.00 S

ATOM 2329 OX1 PHE P2329 -15.786 -13.416 -37.895 0.00 0.00 O

ATOM 2330 OX1 PHE P2330 -15.015 -15.937 -38.388 0.00 0.00 O

ATOM 2331 OX1 PHE P2331 -13.995 -11.689 -37.105 0.00 0.00 O

ATOM 2332 SI1 PHE P2332 -12.317 -17.673 -40.443 0.00 0.00 S

ATOM 2333 SI1 PHE P2333 -13.952 -16.041 -37.207 0.00 0.00 S

ATOM 2334 SI1 PHE P2334 -11.138 -15.008 -40.040 0.00 0.00 S

ATOM 2335 OX1 PHE P2335 -12.301 -16.104 -40.093 0.00 0.00 O

ATOM 2336 OX1 PHE P2336 -11.569 -18.689 -39.497 0.00 0.00 O

ATOM 2337 OX1 PHE P2337 -10.748 -18.019 -37.164 0.00 0.00 O

ATOM 2338 OX1 PHE P2338 -13.339 -17.516 -37.298 0.00 0.00 O

ATOM 2339 OX1 PHE P2339 -13.047 -14.787 -37.533 0.00 0.00 O

ATOM 2340 OX1 PHE P2340 -10.665 -14.862 -38.516 0.00 0.00 O

ATOM 2341 SI1 PHE P2341 -8.539 -20.103 -41.992 0.00 0.00 S

ATOM 2342 SI1 PHE P2342 -10.307 -18.670 -38.552 0.00 0.00 S

ATOM 2343 SI1 PHE P2343 -7.247 -17.409 -41.544 0.00 0.00 S

ATOM 2344 OX1 PHE P2344 -8.406 -18.510 -41.791 0.00 0.00 O

ATOM 2345 OX1 PHE P2345 -7.361 -20.996 -41.366 0.00 0.00 O

ATOM 2346 OX1 PHE P2346 -7.706 -21.750 -38.834 0.00 0.00 O

ATOM 2347 OX1 PHE P2347 -9.958 -20.232 -38.514 0.00 0.00 O

ATOM 2348 OX1 PHE P2348 -9.160 -17.870 -39.243 0.00 0.00 O

ATOM 2349 OX1 PHE P2349 -6.876 -17.449 -39.979 0.00 0.00 O

ATOM 2350 SI1 PHE P2350 -4.928 -22.809 -43.464 0.00 0.00 S

ATOM 2351 SI1 PHE P2351 -6.669 -21.326 -39.952 0.00 0.00 S

ATOM 2352 SI1 PHE P2352 -4.179 -20.125 -42.863 0.00 0.00 S

ATOM 2353 OX1 PHE P2353 -5.005 -21.456 -42.660 0.00 0.00 O

ATOM 2354 OX1 PHE P2354 -3.652 -23.701 -43.130 0.00 0.00 O

ATOM 2355 OX1 PHE P2355 -4.061 -24.469 -40.596 0.00 0.00 O

ATOM 2356 OX1 PHE P2356 -5.818 -22.633 -40.321 0.00 0.00 O

ATOM 2357 OX1 PHE P2357 -5.693 -20.190 -39.413 0.00 0.00 O

ATOM 2358 OX1 PHE P2358 -4.270 -19.346 -41.489 0.00 0.00 O

ATOM 2359 SI1 PHE P2359 -2.977 -24.009 -41.708 0.00 0.00 S

ATOM 2360 OX1 PHE P2360 0.053 -26.200 -41.135 0.00 0.00 O

ATOM 2361 OX1 PHE P2361 -2.193 -25.372 -42.018 0.00 0.00 O

ATOM 2362 OX1 PHE P2362 -1.999 -22.854 -41.122 0.00 0.00 O

ATOM 2363 OX1 PHE P2363 -0.449 -22.121 -43.092 0.00 0.00 O

ATOM 2364 SI1 PHE P2364 0.868 -26.572 -42.456 0.00 0.00 S

ATOM 2365 OX1 PHE P2365 3.140 -29.008 -43.584 0.00 0.00 O

ATOM 2366 OX1 PHE P2366 0.839 -28.108 -42.788 0.00 0.00 O

ATOM 2367 OX1 PHE P2367 1.768 -25.572 -43.334 0.00 0.00 O

ATOM 2368 SI1 PHE P2368 -11.556 -14.332 -37.302 0.00 0.00 S

ATOM 2369 SI1 PHE P2369 -10.617 -11.775 -36.542 0.00 0.00 S

ATOM 2370 OX1 PHE P2370 -11.618 -12.732 -37.313 0.00 0.00 O

ATOM 2371 OX1 PHE P2371 -11.034 -14.860 -35.901 0.00 0.00 O

ATOM 2372 SI1 PHE P2372 -7.807 -17.286 -38.688 0.00 0.00 S

ATOM 2373 SI1 PHE P2373 -6.978 -14.577 -38.122 0.00 0.00 S

ATOM 2374 OX1 PHE P2374 -8.073 -15.724 -38.355 0.00 0.00 O

ATOM 2375 OX1 PHE P2375 -7.245 -18.166 -37.453 0.00 0.00 O

ATOM 2376 OX1 PHE P2376 -6.156 -18.034 -35.078 0.00 0.00 O

ATOM 2377 OX1 PHE P2377 -6.396 -14.540 -36.643 0.00 0.00 O

ATOM 2378 SI1 PHE P2378 -4.274 -19.664 -39.941 0.00 0.00 S

ATOM 2379 SI1 PHE P2379 -5.863 -18.102 -36.636 0.00 0.00 S

ATOM 2380 SI1 PHE P2380 -3.154 -17.024 -39.596 0.00 0.00 S

ATOM 2381 OX1 PHE P2381 -4.218 -18.182 -39.365 0.00 0.00 O

ATOM 2382 OX1 PHE P2382 -3.031 -20.601 -39.535 0.00 0.00 O

ATOM 2383 OX1 PHE P2383 -3.217 -21.333 -36.935 0.00 0.00 O

ATOM 2384 OX1 PHE P2384 -4.757 -19.216 -36.896 0.00 0.00 O

ATOM 2385 OX1 PHE P2385 -5.117 -16.794 -37.087 0.00 0.00 O
[truncated: 69,487 more chars]
